# Encapsulation of greenhouse gases in clathrate hydrates with insights into structure, energetics, chemical interactions, and environmental implications

**DOI:** 10.1038/s41598-025-08202-z

**Published:** 2025-07-05

**Authors:** Arun Ramamurthy, Giridhar Baburao, Abburi Jahnavi, Gopi Ragupathy

**Affiliations:** Department of Chemistry, School of Advanced Sciences, Vellore Institute of Technology, Vellore, 632014 India

**Keywords:** Environmental chemistry, Physical chemistry, Theoretical chemistry, Environmental sciences

## Abstract

The encapsulation of greenhouse gases (GHGs), such as CCl$_4$, CF$_2$Cl$_2$, CH$_3$Br, CH$_3$Cl, CH$_4$, CO, CO$_2$, H$_2$S, CH$_3$F, N$_2$O, NF$_3$, O$_3$, CF$_4$, SF$_6$, and SO$_2$, within 5$^{12}$, 5$^{12}$6$^2$, and 5$^{12}$6$^4$ clathrate hydrate cages is being investigated in our study using Density Functional Theory (DFT). The smaller cages introduce steric constraints, leading to bond distortions and significant vibrational blue-shifts, while larger cages offer greater flexibility, resulting in red-shifts or minimal vibrational alterations. Intermediate-sized cages provide a nuanced balance between spatial limitations and structural stabilization. In the $5^{12}$ cage, fewer guests exhibit red-shifts, whereas the larger $5^{12}6^2$ and $5^{12}6^4$ cages show more frequent red-shifts due to enhanced host–guest interactions. Natural Bonding Orbital (NBO) analysis reveals systematic changes in orbital contributions, occupancies, and anti-bonding interactions upon encapsulation, highlighting enhanced bond stabilization and a reduction in environmental reactivity. Atoms in Molecules (AIM) analysis further corroborates that encapsulated molecules exhibit strong bonding, remaining securely trapped and exhibiting minimal reactivity. Energy Decomposition Analysis (EDA) indicates that while smaller cages amplify interaction energies, they can also introduce substantial steric strain. In contrast, Non-Covalent Interaction (NCI) analysis highlights that the stability of hydrate cages is enhanced by stronger H-bonds and weaker van der Waals interactions as cage size increases. This underscores the potential of clathrate hydrates for encapsulating GHGs.

## Introduction

Clathrate hydrates (CHs), also referred to as gas hydrates, are solid, crystalline structures resembling ice, where water molecules form a lattice framework through intricate hydrogen bonding interactions^1,2^. This lattice creates cavities that can trap GHGs. Notably, there is no chemical bonding between the water molecules and the enclosed guests^1,3^. They naturally occur in specific regions, such as oceanic sediments and permafrost areas across the globe^4,5^. These cages have the ability to encapsulate large quantities of gas molecules, showcasing their efficiency in natural gas storage^6^. The amount of gas contained in clathrates is estimated to be considerably greater than those from all other fossil fuel sources^7,8^. CHs have thus been considered to be a promising future energy source. The formation of CHs occurs under specific environmental conditions, typically involving low temperatures ($\le 300$ K) and high pressures ($\ge 38$ bar at 277 K)^9,10^. However, few studies suggest that these hydrates can form even at extremely low pressures ($\sim 10^{-10}$ mbar) and temperatures (10 K to 60 K), which reinforces their potential existence in the interstellar medium (ISM)^11–14^. Clathrates likely formed within the solar nebula, capturing volatile gases in icy solids^15,16^. Near the surfaces of Mars, carbon dioxide clathrates remain stable within the polar ice caps^17,18^. Carbon monoxide is abundant in the cosmos, found in gaseous nebulae, the interstellar medium, planetary atmospheres, and cometary ice, potentially in clathrate form^19^. The formation of these clathrate phases may play a crucial role in the development of nebulae, comets, and the outer planets of the solar system^20,21^.

Gas hydrates have gained attention due to their unique crystalline structures, which naturally form three distinct types: Structure-I (sI), Structure-II (sII), and Structure-H (sH)^22^. sI and sII are cubic structures, while sH is hexagonal. These structures vary based on cage size, type, and crystal arrangement. Structure-I consists of two small (5$^{12}$) and six larger (5$^{12}$6$^{2}$) cages per unit cell, with 46 water molecules, accommodating gas molecules of 4.0–5.5 Å in diameter^23^. Structure-II contains 16 (5$^{12}$) and 8 (5$^{12}$6$^{4}$) cages, with 136 water molecules per unit cell and is suitable for gases having a diameter of 6.0–7.0 Å^24^. Structure-H crystallizes in a P6/mmm arrangement, incorporating layers of (5$^{12}$), (4$^{3}$5$^{6}$6$^{3}$), and (5$^{12}$6$^{8}$) cages^25^. The 5$^{12}$ cages are common across all hydrates, while 5$^{12}$6$^{2}$ and 5$^{12}$6$^{4}$ are unique to sI and sII, respectively^26^. The X-ray diffraction study reveals that the CO clathrate hydrate adopts an sI structure with a lattice constant of 11.88 Å, and crystallization occurs at a pressure of 17.3 MPa and a temperature of around 243 K^27^. A recent computational study has demonstrated how CO is stabilized within specific molecular cages, 5$^{12}$, 5$^{12}$6$^{2}$, and 5$^{12}$6$^{4}$, through various types of interactions. The study concluded that vdW forces play a pivotal role in smaller cages, gradually diminishing in significance as the cage size increases^23^. Previous studies have demonstrated that the shape and size of clathrate hydrate cages exert a significant influence on the vibrational and encapsulated guest molecule’s electronic properties. Lower cages such as 5$^{12}$ induce blue-shifts in vibrational stretching modes as a result of spatial confinement and steric hindrance, and the larger cages have weaker confinement effects, leading to smaller frequency shifts. The nature of host–guest interaction, for example, hydrogen bonds and van der Waals forces, Dipole-induced interactions are crucial in the modulation of the vibrational signature, which is accessible by means of spectroscopy techniques such as infrared and Raman spectroscopy. In addition, entrapment within the polar environment of hydrogen-bonded water cages has been demonstrated to change guest species dipole moment by its influence on electron distribution, thereby influencing their dielectric and spectroscopic properties. There have also been experimental and theoretical investigations that have set up a direct structure–function correlation, where cage structure determines molecular orientation and accompanying thermodynamic and vibrational properties^28–30^.

The major GHGs present in the atmosphere include CH$_4$, CO$_2$, SO$_2$, N$_2$O, NH$_3$, H$_2$S, CCl$_4$, CH$_3$Br, CF$_2$Cl$_2$, and CH$_3$Cl. These gases are emitted from various sources, including motorized traffic, power plants, industries, agricultural and biological waste, and other human activities. These gases mainly result from fossil fuel combustion^31,32^. CH$_4$ released during natural gas and oil extraction, obstructs pipelines and production^33^. Carbon monoxide contributes to ground-level ozone, harming human health and vegetation^34^. N$_2$O is 300 times more potent than CO$_2$ in global warming and contributes to ozone depletion^35^. Halogenated carbons like CCl$_4$, CH$_3$Br, CF$_2$Cl$_2$, and CH$_3$Cl are long-lasting gases with significant ozone-depleting potential^36^. Excessive release of GHGs has severely impacted the climate, causing global warming and extreme weather^37^. Hence, the development of safe, effective, and environmentally friendly gas storage techniques is essential for reducing the greenhouse gas effect. Traditionally, gases like methane, carbon dioxide, and hydrogen are stored in compressed or liquefied forms^38,39^. Traditional gas storage methods, requiring high pressures or cryogenic temperatures, pose safety risks and high energy costs. As an alternative, adsorbent-based storage has gained attention^40^, utilizing materials like activated carbon^41^, carbon nanotubes^42^, graphene^43^, and metal-organic frameworks (MOFs)^44^ to adsorb gases. While these materials demonstrate excellent storage capacities, their high manufacturing costs hinder large-scale industrial use. Furthermore, fullerenes offer the potential to encapsulate small molecules due to their nearly spherical cavities^45^. Endohedral and open cage fullerenes have been used for the encapsulation of GHGs^46–49^. Yoshifumi Hashikawa et al. encapsulated CO$_2$ inside open-cage C$_{60}$ fullerenes^50^. X-ray diffraction, infrared spectroscopy, and computational studies revealed that confinement induced unique intramolecular interactions, restricting the motion of CO$_2$ and enabling it to act as both a Lewis acid and a Lewis base. Additionally, the trapped CO$_2$ altered the external properties of the fullerene by enhancing hydrogen bonding. Furthermore, the same group successfully encapsulated an NH$_3$ molecule in $\pi$-extended fullerenes^51^. Under mild conditions, CO was also encapsulated into an open-cage C$_{59}$ fullerene^52^. Bloodworth, S. et al. synthesized an endohedral C$_{60}$ fullerene encapsulating a single methane molecule^53^. Additionally several previous studies have also reported the encapsulation of molecular hydrogen and water inside fullerenes^54–59^. Another promising option is clathrate hydrate storage, offering a safer and more efficient solution for long-term gas containment^6^. Linga et al. emphasize hydrate formation as a promising method for trapping GHGs through experimental studies^6,60^. Recent experiments on CH$_4$ and CO$_2$ storage, conducted under isochoric and non-stirred conditions, demonstrate the potential for large-scale applications, making hydrates ideal for storage and transportation^2,61,62^. By utilizing clathrate hydrates, harmful emissions can be trapped, preventing their atmospheric release and mitigating climate impact. Clathrate hydrates are also a key gas storage reservoir on Earth^63^. Beyond gas storage, they hold promise in industrial applications such as gas separation^64,65^, where they selectively capture specific gases, and water desalination^66^, where they exclude salts during formation, offering a novel approach to freshwater production and sustainable environmental management^65,67^.

Quantum chemical studies have further advanced our understanding of clathrate hydrates, focusing on the molecular interactions that stabilize these structures^68,69^ particularly the stability of guest molecules encapsulated within the water cages of clathrate hydrates^70,71^. A recent study states that the stability of clathrate hydrate structures is primarily due to two interactions: hydrogen bonding between the water molecules forming the polyhedral cages and weak molecular forces between the trapped gas molecules and the surrounding water framework^23^. These weak molecular interactions ensure the stabilization of the crystalline lattice, enabling it to encapsulate a wide range of gases effectively^26^. These guest–host interactions significantly influence the behavior of both the guest and the clathrate hydrate framework. Guest molecules with higher basicity have been observed to form stronger hydrogen bonds with the water molecules in the clathrate framework, facilitating their formation even at low temperatures^72,73^. In contrast, guest molecules with lower basicity form weaker guest–host hydrogen bonds compared to the water–water hydrogen bonds within the clathrate structure. For such weakly basic guests, thermal excitation at higher temperatures is necessary to disrupt the water–water bonds of the framework, enabling guest-water hydrogen bond formation^31^.

Despite clearly defined structural and energetic properties of clathrate hydrates, the vibrational motion of the guest molecules under confinement is not well known. In particular, how changes in vibrational frequencies are connected to guest–host changes interactions and cage size is not systematic. The mechanistic origin of large blue or red-shifts in bond vibrations, which demonstrates the effects of confinement, remains insufficiently examined. Limited research connects these alterations to discernible spectroscopic indicators or anticipates their ecological relevance. An extensive vibrational analysis based on DFT that addresses this disparity is presently lacking. in the literature^23,68,70^.

We investigated the encapsulation of GHGs within 5$^{12}$, 5$^{12}$6$^{2}$, and 5$^{12}$6$^{4}$ cages. Through a computational approach, we analyzed minimal structural perturbations upon encapsulation, with stabilization primarily driven by vdW forces, hydrogen bonding, and electrostatic interactions. Vibrational frequency analysis was conducted to elucidate blue and red-shifts in key bond stretching regions. To further probe the nature of host–guest interactions, quantum topological analyses were performed. This research scientifically investigates the encapsulation of 15 greenhouse gases, among which lesser-studied halogenated compounds, in three forms of clathrate hydrate cages. Unlike prior studies focused mainly on CH$_4$, CO, CO$_2$, and H$_2$ clathrates, the current work expands the scope to diverse GHGs of atmospheric relevance. Using DFT, EDA, NBO, and AIM analyses, it disentangles the nature and the host–guest interaction strength. There is an important innovation in combining vibrational confinement effects with energy decomposition, providing anticipatory understanding regarding spectroscopic identification and the efficacy of gas retention.

## Results and discussion

### Structural characteristics and vibrational frequency analysis

#### Cage geometry and distortion energies

**Fig. 1:**
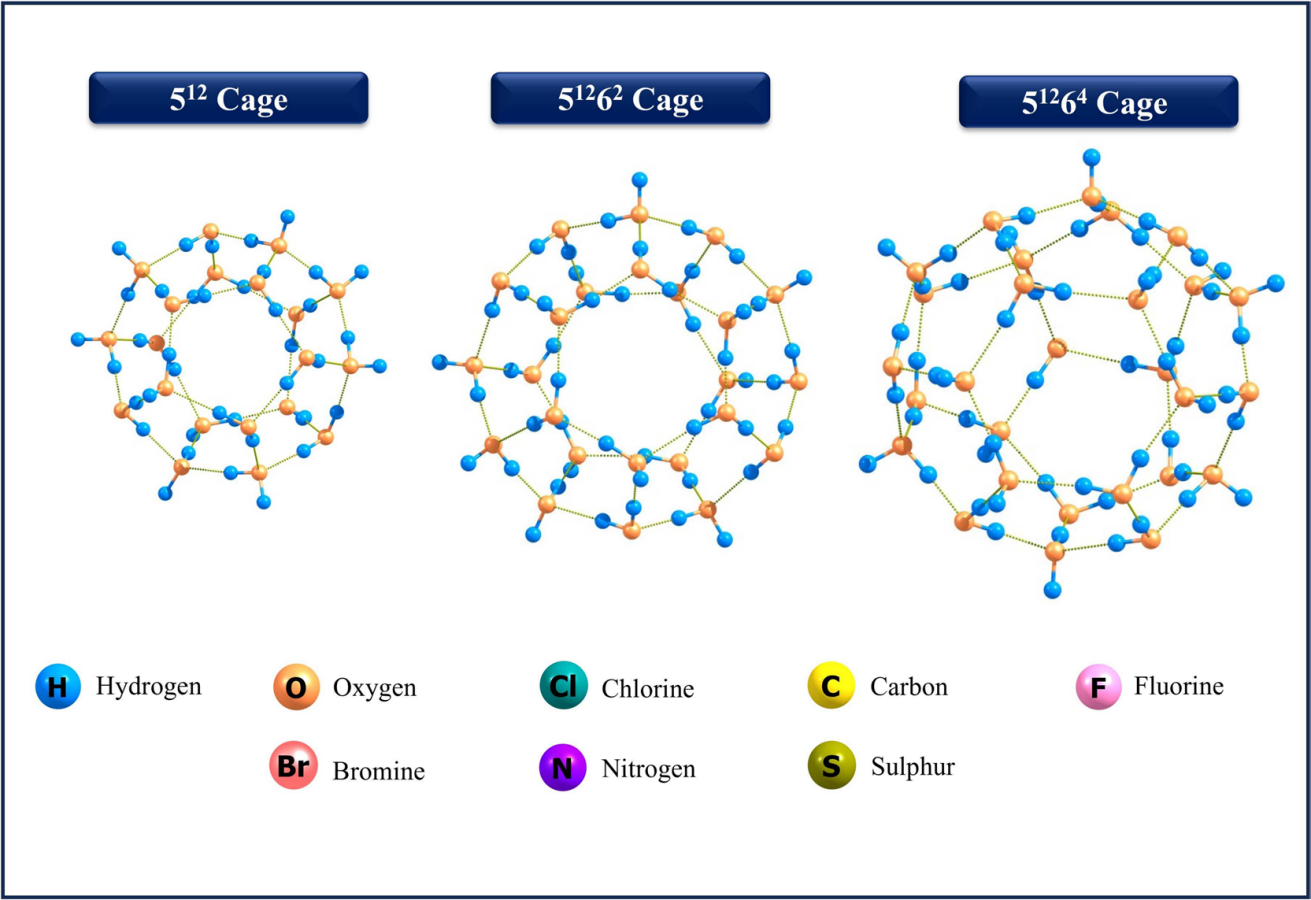
Optimized geometries of 5$^{12}$, 5$^{12}6^2$, and 5$^{12}6^4$ hydrate clathrate cages without encapsulated GHGs.

Studies investigate three distinct clathrate hydrate host structures $5^{12}$, $5^{12}6^2$, and $5^{12}6^4$ comprising 20, 24, and 28 water molecules, respectively, to explore the encapsulation of GHGs and compute relevant quantum chemical parameters (Fig. 1). The cavity diameters of the cages $5^{12}$, $5^{12}6^2$, and $5^{12}6^4$ are 7.76 Å, 8.72 Å, and 9.16 Å, respectively. Upon encapsulation of guest molecules, the host water framework undergoes anisotropic distortion, meaning the structural deformation is not uniform in all directions. This effect is especially pronounced for larger or asymmetrically shaped guest molecules, which interact more strongly with specific faces or directions of the cage, leading to non-uniform stretching or compression of O–O bond lengths within the cage structure. The distortion energy of clathrate hydrate cages becomes more negative with increasing cage size (5$^{12}$ < 5$^{12}$6$^2$ < 5$^{12}$6$^4$), indicating improved guest accommodation and reduced strain. Guest properties such as size, shape, polarity, and polarizability also influence distortion. Small, weakly interacting gases like CH$_4$ and CO fit efficiently with minimal perturbation, while larger polarizable molecules like SF$_6$ show improved energies in larger cages due to better dispersion and steric compatibility. Polar or hydrogen-bonding guests like H$_2$S and SO$_2$ exhibit moderate stabilization, balancing electrostatic interactions and structural strain (see supporting information, Table S11).

**Fig. 2:**
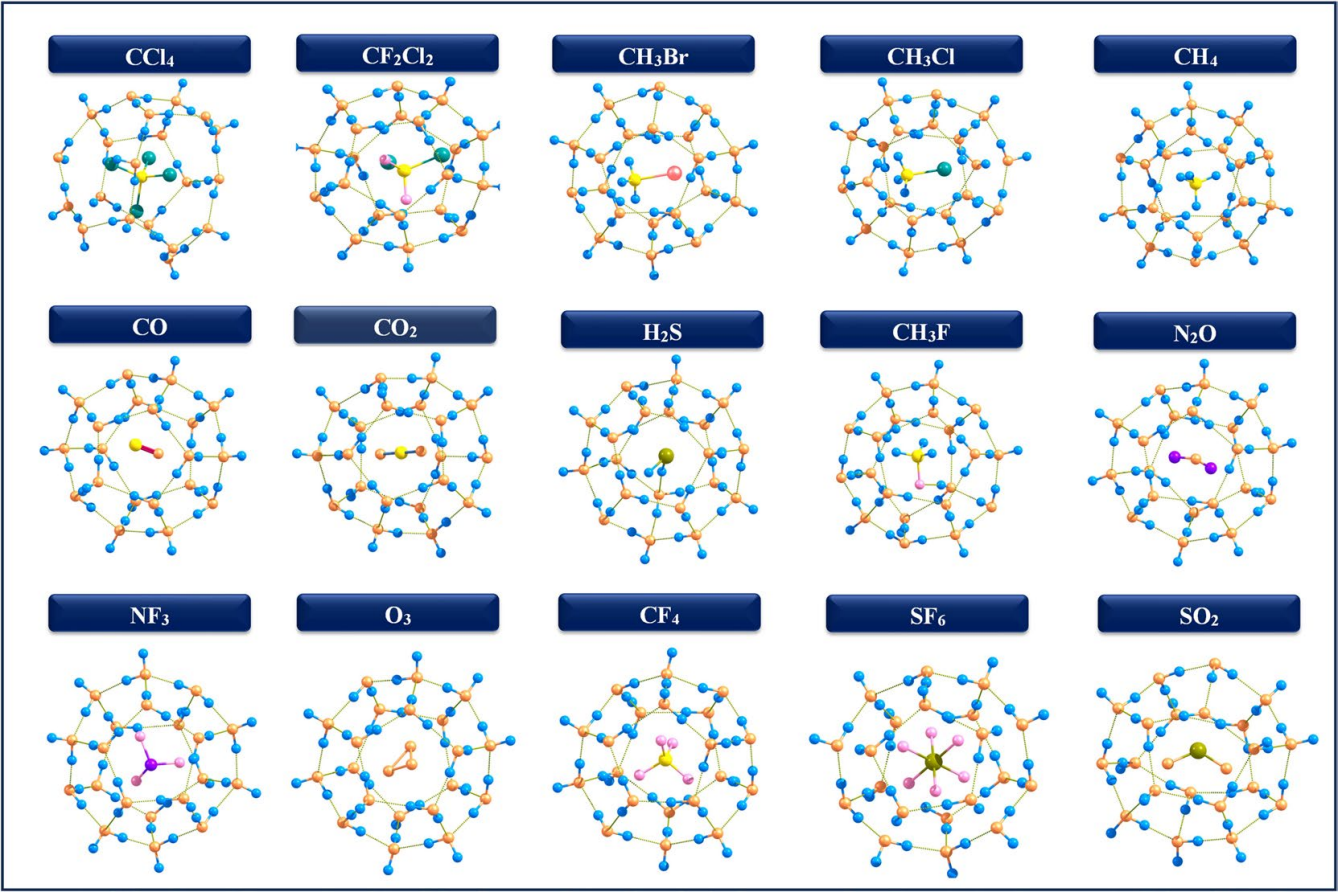
Optimized geometries of 5$^{12}$ clathrate hydrates encapsulated GHGs.

**Fig. 3:**
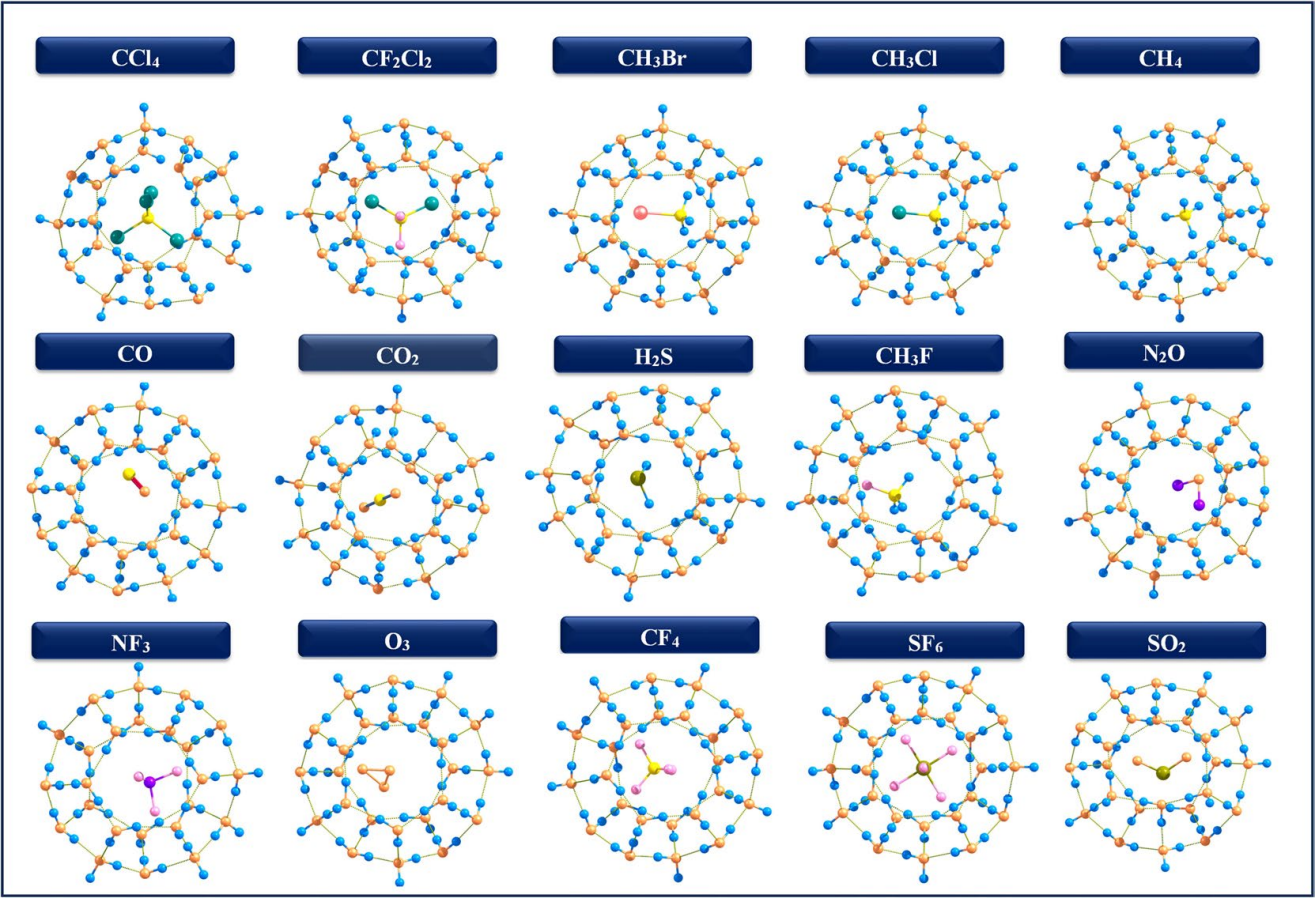
Optimized geometries of 5$^{12} 6^2$ clathrate hydrates encapsulated GHGs.

**Fig. 4:**
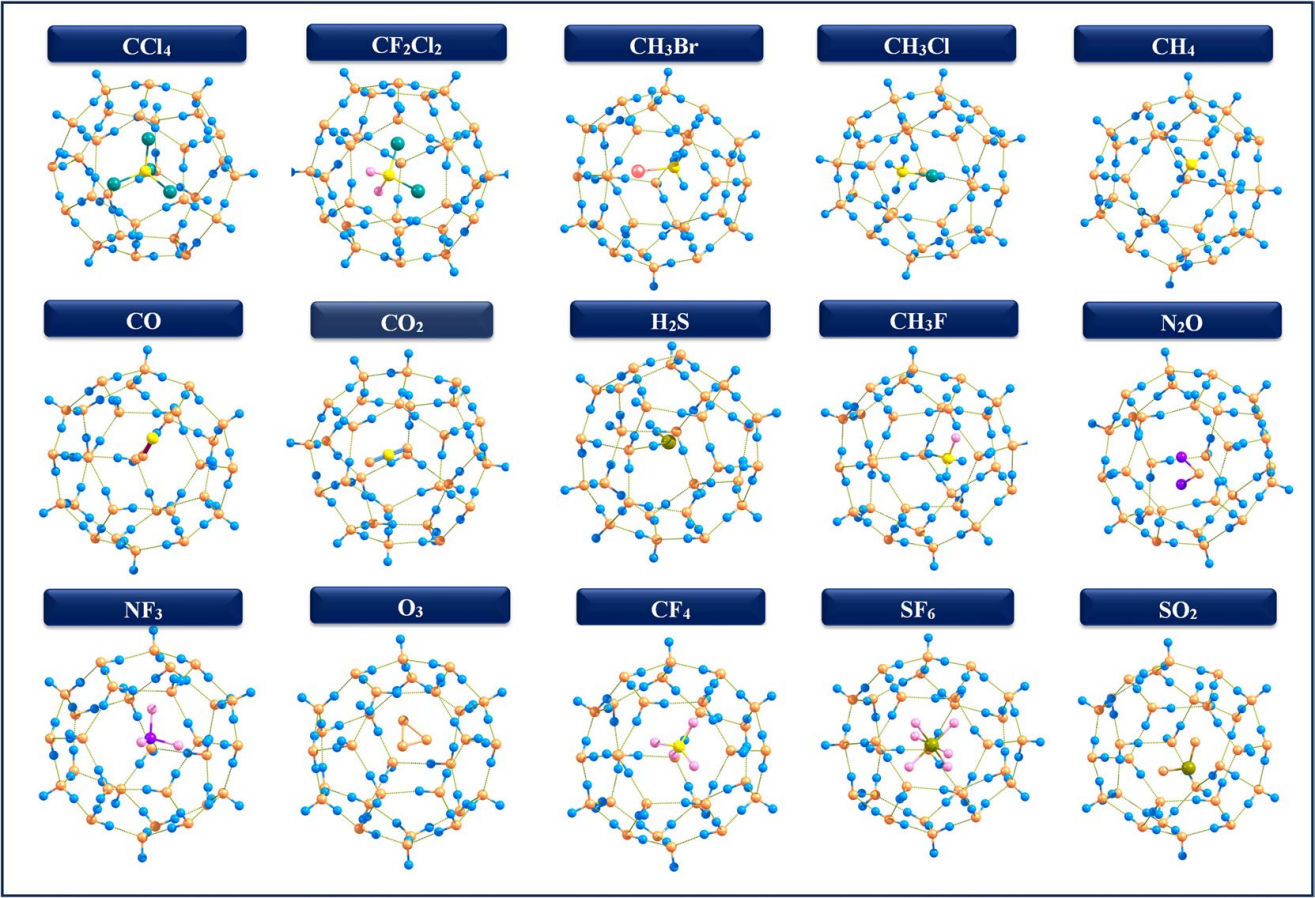
Optimized geometries of 5$^{12} 6^4$ clathrate hydrates encapsulated GHGs.

In the $5^{12}$ cage, the encapsulated molecule is centrally located, whereas larger molecules such as CCl$_4$, CF$_2$Cl$_2$, CH$_3$Br, CH$_3$Cl, and SF$_6$ are hosted in the $5^{12}6^2$, and $5^{12}6^4$ cages, with smaller molecules adopting off-centered positions in these larger structures (Figs. 2, 3 and 4) Specifically, CCl$_4$ assumes a bowl-shape within the $5^{12}$ cage due to the steric hindrance and high electronegativity of the chlorine atoms, coupled with the constrained size of the cage, which affects the molecular geometry within the clathrate. The data highlights the variations in the diameters of guest molecules Tables 1, 2 and 3 encapsulated within three types of hydrate clathrate cages $5^{12}$, $5^{12}6^2$, and $5^{12}6^4$ underscoring the influence of cage size and geometry on molecular fit. As noted in the introduction, the diameters of these molecules fall within the acceptable range, allowing all of them to fit comfortably within the clathrate structures. Despite adjustments during encapsulation, the overall cage structure remains largely intact with minimal distortion. Key geometric parameters, including bond length variations ($\Delta r$) and dipole moments ($\mu$) are summarized in Tables 1, 2 and 3. In the $5^{12}$ cage, some encapsulated molecules have a red-shift. In contrast, more guest molecules have a red-shift in the $5^{12}6^2$ and $5^{12}6^4$ cages. This is due to the larger cavity size, which promotes stronger host–guest interactions. These conditions promote bond elongation, resulting in a shift of vibrational frequencies to lower wavenumbers. These results show the significant influence of cage size on the vibrational and structural features of encapsulated species.

#### Dipole moment modulation

After the encapsulation of greenhouse gases their dipole moments show notable changes, reflecting altered electronic environments due to host–guest interactions. Nonpolar molecules like CCl$_4$, CF$_4$, CO$_2$, and SF$_6$, which originally have zero or negligible dipole moments, develop small induced dipoles (e.g., SF$_6$ rises to 0.17 D in $5^{12}6^{2}$), suggesting polarization by the cage’s electrostatic field. For slightly polar molecules like CF$_2$Cl$_2$ and NF$_3$, the dipole moment increases within the cage, peaking in intermediate-sized cavities due to optimal alignment and interaction with the water lattice. Strongly polar gases such as CH$_3$Cl, CH$_3$F, and SO$_2$ retain similar dipole values post-encapsulation, indicating minimal perturbation but slight reorientation inside the host. CO and N$_2$O show marginal dipole shifts, suggesting stable confinement without significant distortion. Remarkably, the largest cage ($5^{12}6^{4}$) often reduces induced dipole moments (e.g., CF$_2$Cl$_2$ drops from 0.24 D in $5^{12}6^{2}$ to 0.11 D), implying less confinement-induced polarization. Encapsulation within clathrate hydrates alters bond lengths due to steric constraints and weak molecular interactions.

The vibrational frequency analysis reveals structural changes due to confinement, affecting molecular vibrations. A blue-shift ($\Delta \nu > 0$) indicates bond strengthening and reduced bond length ($\Delta r < 0$), while a red-shift ($\Delta \nu < 0$) signifies bond weakening and increased bond length ($\Delta r > 0$)^74,75^. Encapsulation within clathrate hydrate cages alters symmetric stretching frequencies through host–guest interactions, confinement effects, and molecular structure. The discussion explores these trends, emphasizing chemical mechanisms and cage type influences. The Tables 1, 2 and 3 represent the change in stretching vibrational frequency ($\Delta \nu$). CCl$_4$ exhibits moderate blue-shifts in smaller cages (5.58 cm$^{-1}$ in 5$^{12}$, 23.06 cm$^{-1}$ in 5$^{12}$6$^2$) due to vdW stabilization, decreasing in the largest cage (8.05 cm$^{-1}$). CF$_2$Cl$_2$ shows contrasting trends: C–Cl bonds experience blue-shifts (26.13 cm$^{-1}$, 14.60 cm$^{-1}$, 3.40 cm$^{-1}$), while C–F bonds shift from a slight blue-shift (5.63 cm$^{-1}$) in 5$^{12}$ to red-shifts (− 0.02 cm$^{-1}$, − 1.21 cm$^{-1}$) in larger cages due to bond relaxation. CH$_3$Br and CH$_3$Cl exhibit strong blue-shifts in C–H bonds (43.68 cm$^{-1}$, 40.20 cm$^{-1}$) in small cages, decreasing in larger ones. Meanwhile, their C–X bonds (X=Cl, Br) show consistent red-shifts, indicating bond weakening due to polarization effects. Nonpolar molecules like CH$_4$ and CO$_2$ experience minimal shifts, reflecting weak host–guest interactions. CO undergoes red-shifts in 5$^{12}$ and 5$^{12}$6$^2$ cages (− 5.20 cm$^{-1}$, − 9.70 cm$^{-1}$, respectively) due to lone pair interactions but shifts slightly positive (0.47 cm$^{-1}$) in 5$^{12}$6$^4$. H$_2$S exhibits a dramatic blue-shift (622.10 cm$^{-1}$) in 5$^{12}$, decreasing in larger cages, highlighting strong hydrogen bonding. Large vibrational blue-shift observed in H$_2$S serve as quantitative indicators of strong host–guest interactions and spatial confinement within smaller cages. These shifts, coupled with favorable interaction energy values, underscore a direct relationship between cage-induced steric effects, vibrational modulation, and molecular stabilization^76^. CH$_3$F shows blue-shifts in C–H bonds and red-shifts in C–F bonds due to cage polarization. O$_3$ and SO$_2$ display strong blue-shifts in small cages, turning to red-shifts in larger ones, emphasizing steric and polarization effects. Symmetric molecules like CF$_4$ and SF$_6$ show small shifts, with blue-shifts in smaller cages transitioning to red-shifts in larger ones. Hence, smaller cages induce stabilization through vdW and polarization effects, while larger cages reduce these interactions. Polar molecules exhibit distinct blue- and red-shift trends, affecting their reactivity and environmental impact. Greenhouse gases encapsulated within clathrate hydrates were found to exhibit both red and blue-shifts in their hydrogen bond stretching frequencies. Specifically, GHGs such as CH$_3$F, CH$_3$Cl, and CH$_3$Br, which exhibit a blue-shift upon encapsulation, showed reduced IR activity compared to their monomeric forms. The corresponding IR intensity data are provided in the Supporting Information (Table S12). Furthermore, these GHGs encapsulated within clathrate cages were observed to have lower IR activity compared to their analogues encapsulated in endofullerenes^48,53^.

**Table 1:** Vibrational frequency shifts ($\Delta \nu$ in cm$^{-1}$), bond length changes ($\Delta r$ in mÅ), interaction energies ($\Delta E_{\text {int}}$ in kcal/mol), dipole moments of pure guest molecules ($\mu$ in Debye), and diameter (*d* in Å) for GHGs within the 5$^{12}$ cage.

Molecule	Bond	$\Delta \nu$ (cm$^{-1}$)	$\Delta r$ (mÅ)	$\Delta E_{\text {int}}$ (kcal/mol)	$\mu$ (Debye)	*d* (Å)
CCl$_4$	C–Cl	5.58	5.64	2.25	0.07	2.93
CF$_2$Cl$_2$	C–Cl	26.13	− 23.23	− 2.40	0.20	2.91
	C–F	5.63	16.62			
CH$_3$Br	C–H	43.68	− 5.29	− 31.27	1.98	2.50
	C–Br	− 2.19	5.94			
CH$_3$Cl	C–H	40.20	− 3.56	− 18.57	2.11	2.38
	C–Cl	− 16.97	3.82			
CH$_4$	C–H	2.79	− 0.75	− 14.93	0.01	1.79
CO$_2$	C–O	3.10	− 3.4	− 16.84	0.10	2.34
CO	C–O	− 5.20	0.90	− 12.4	0.06	1.14
H$_2$S	S–H	622.10	6.07	− 22.12	1.43	2.02
CH$_3$F	C–H	53.26	− 6.12	− 28.53	1.85	2.03
	C–F	− 74.20	32.40			
N$_2$O	O–N	5.96	− 2.06	− 15.06	1.11	1.77
NF$_3$	N–F	4.70	− 3.58	− 15.77	0.14	2.15
O$_3$	O–O	48.97	− 160.82	− 12.69	0.00	1.44
CF$_4$	C–F	8.05	− 7.39	− 16.85	0.08	2.17
SF$_6$	S–F	5.17	− 9.20	− 11.39	0.16	3.20
SO$_2$	S–O	6.20	8.97	− 31.76	1.98	2.49

**Table 2:** Vibrational frequency shifts ($\Delta \nu$ in cm$^{-1}$), bond length changes ($\Delta r$ in mÅ), interaction energies ($\Delta E_{\text {int}}$ in kcal/mol), dipole moments of pure guest molecules ($\mu$ in Debye), and diameter (*d* in Å) for GHGs within the 5$^{12}$6$^{2}$ Cage.

Molecule	Bond	$\Delta \nu$ (cm$^{-1}$)	$\Delta r$ (mÅ)	$\Delta E_{\text {int}}$ (kcal/mol)	$\mu$ (Debye)	*d* (Å)
CCl$_4$	C–Cl	23.06	− 20.34	− 8.05	0.06	2.89
CF$_2$Cl$_2$	C–Cl	14.60	− 10.37	− 20.14	0.24	2.92
	C–F	0.02	12.33			
CH$_3$Br	C–H	7.49	− 0.62	− 28.87	1.98	2.52
	C–Br	− 17.60	14.44			
CH$_3$Cl	C–H	15.86	− 0.66	− 20.91	2.13	2.39
	C–Cl	− 27.22	12.64			
CH$_4$	C–H	− 0.38	− 0.06	− 11.67	0.01	1.78
CO$_2$	C–O	0.89	2.44	− 15.98	0.09	2.34
CO	C–O	− 9.70	1.51	− 10.32	0.05	1.14
H$_2$S	S–H	323.31	13.31	− 20.14	1.42	1.99
CH$_3$F	C–H	29.96	− 3.24	− 17.98	1.80	2.04
	C–F	− 53.20	20.60			
N$_2$O	O–N	− 0.60	1.05	− 13.64	1.10	1.77
NF$_3$	N–F	− 1.32	8.30	− 14.71	0.12	2.15
O$_3$	O–O	− 20.55	4.10	− 10.86	0.00	1.44
CF$_4$	C–F	− 1.13	− 0.57	− 17.08	0.08	2.17
SF$_6$	S–F	− 8.46	3.97	− 25.76	0.17	3.21
SO$_2$	S–O	− 7.24	2.92	− 25.37	1.93	2.50

**Fig. 5:**
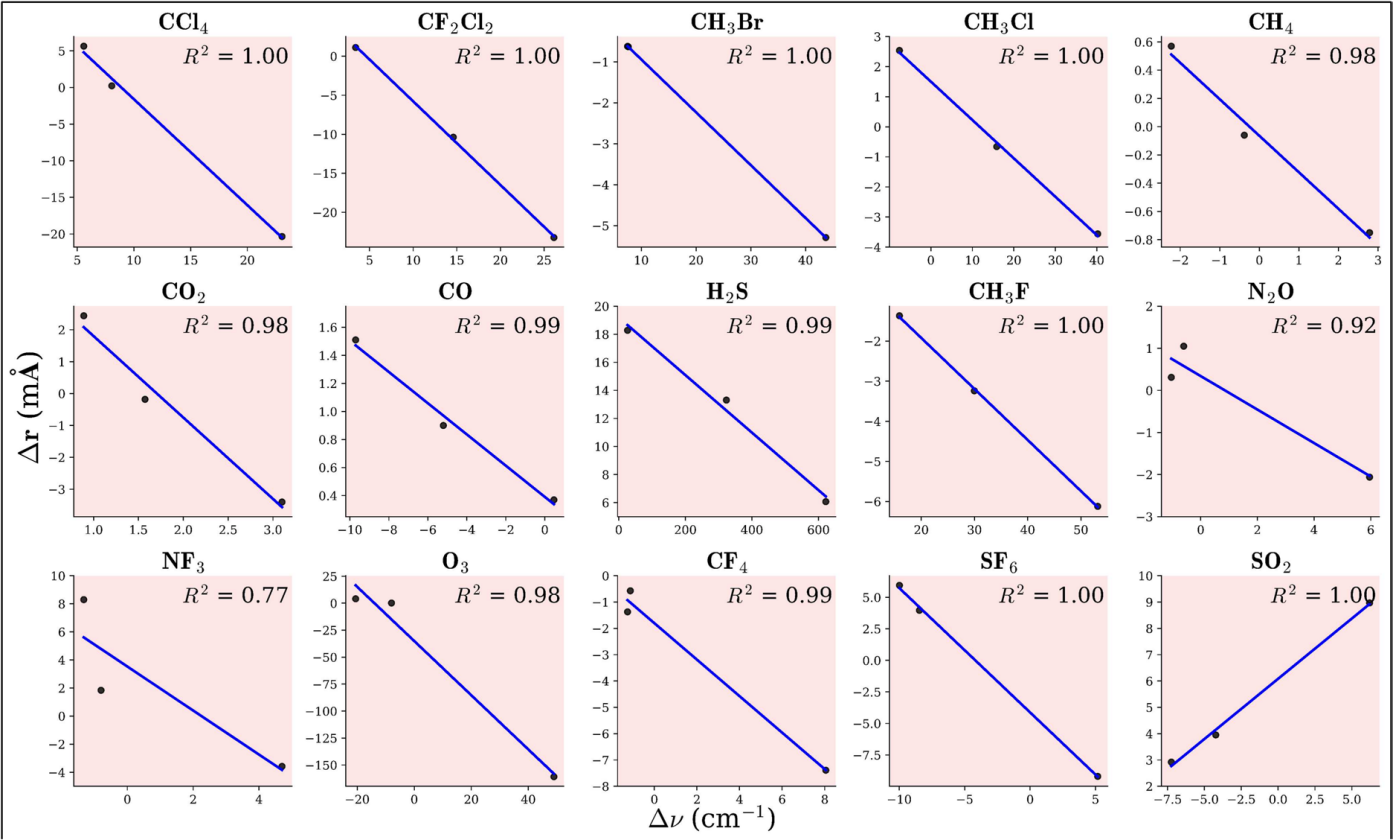
Plot for the relation between $\Delta \nu$ (in cm$^{-1}$) v/s $\Delta r$ (in mÅ) for the GHGs encapsulated within the 5$^{12}$, 5$^{12} 6^2$ and 5$^{12} 6^4$ clathrate hydrates. For CH$_3$F, CH$_3$Cl, and CH$_3$Br, the C–H bond is considered, whereas for CF$_2$Cl$_2$, the C–F bond is taken into consideration.

The inverse relationship between stretching frequency ($\nu$) and bond length (*r*) is evident as $\nu$ increases, *r* decreases, and vice versa. This trend aligns with observations, resulting in a linear plot of $\Delta \nu$ (in cm$^{-1}$) versus $\Delta r$ (in mÅ). The ($R^2$) values for all plots are found to be nearly equal to 1, confirming the linearity of the relationship. The observed $\Delta \nu$ − $\Delta r$ relationship, as illustrated in Fig. 5, helps predict how different guest molecules will behave within clathrate structures. The positive slope in SO$_2$ of the $\Delta \nu$-$\Delta r$ relationship results from its bent geometry, dipole moment, and unique interactions with clathrate cages, altering its bond force constant and vibrational behavior. The observed increase in total electron density with increasing bond length suggests an unusual bond strengthening mechanism. Additionally, a more positive Laplacian of electron density with increasing bond length, in comparison to other molecules, indicates that external polarization effects from the clathrate cage may be stabilizing the bond, leading to an increase in vibrational frequency. Furthermore, second-order perturbation energy from natural bond orbital analysis shows rising stabilization energy for charge transfer interactions (e.g., LP(O) $\rightarrow$ $\sigma ^*$(S–O)), counteracting bond weakening. This modifies the bond stabilization, increasing vibrational frequency with bond length. For CF$_4$, the slight deviation in the curve arises due to secondary interactions. A comparable total electron density in the 5$^{12}$6$^2$ and 5$^{12}$6$^4$ cages, along with an increase in antibonding occupancy, supports this observation. O$_3$ has a delocalized electronic structure with significant resonance effects, leading to a more flexible bond character. The distinct behavior of SO$_2$ can be attributed to its ability to engage in dipole-induced interactions within the clathrate cage, which modifies the electron density distribution and affects vibrational properties. In contrast, O$_3$ lacks a permanent dipole moment and is less influenced by such interactions, maintaining a conventional inverse $\Delta \nu$ − $\Delta r$ trend. Molecules with larger frequency shifts in smaller cages form more stable clathrates, whereas minimal shifts indicate weaker host–guest interactions. Clathrate cage size (5$^{12}$, 5$^{12}$6$^{2}$, 5$^{12}$6$^{4}$) modulates frequency shifts through confinement effects. Smaller 5$^{12}$ cages enhance steric and electrostatic interactions, amplifying shifts, as seen in CH$_3$F’s largest C–H blue-shift. Medium 5$^{12}$6$^{2}$ cages provide moderate shifts, such as CO’s red-shift, while larger 5$^{12}$6$^{4}$ cages allow greater molecular freedom, reducing interactions and shifts. Molecular properties influence frequency shifts: H$_2$S exhibits large blue-shifts due to strong hydrogen bonding, while CH$_3$X (X = Cl, Br, F) shows blue-shifts in C–H bonds and red-shifts in C–X bonds. Nonpolar molecules (CO, N$_2$O, SF$_6$) and symmetric CH$_4$ show minimal shifts due to weak host–guest interactions. Clathrate cage size ($5^{12}$, $5^{12}6^{2}$, $5^{12}6^{4}$) modulates frequency shifts through confinement effects.

**Table 3:** Vibrational frequency shifts ($\Delta \nu$ in cm$^{-1}$), bond length changes ($\Delta r$ in mÅ), interaction energies ($\Delta E_{\text {int}}$ in kcal/mol), dipole moments of pure guest molecules ($\mu$ in Debye), and diameter (*d* in Å) for GHGs within the 5$^{12}$6$^{4}$ Cage.

Molecule	Bond	$\Delta \nu$ (cm$^{-1}$)	$\Delta r$ (mÅ)	$\Delta E_{int}$ (kcal/mol)	$\mu$ (Debye)	d (Å)
CCl$_4$	C–Cl	8.05	0.23	− 25.41	0.04	2.92
CF$_2$Cl$_2$	C-Cl	3.40	1.12	− 21.94	0.11	2.94
	C–F	− 1.21	1.61			
CH$_3$Br	C–H	7.63	− 0.63	− 22.88	1.96	2.54
	C–Br	− 14.75	11.96			
CH$_3$Cl	C–H	− 7.59	2.54	− 16.04	2.10	2.38
	C–Cl	− 11.66	6.66			
CH$_4$	C–H	− 2.22	0.57	− 9.99	0.01	1.78
CO$_2$	C–O	1.57	− 0.18	− 10.74	0.03	2.34
CO	C–O	0.47	0.37	− 8.19	0.06	1.14
H$_2$S	S–H	26.21	18.28	− 15.33	1.42	1.98
CH$_3$F	C–H	15.89	− 1.37	− 13.53	1.77	2.03
	C–F	− 29.81	12.32			
N$_2$O	O–N	− 1.04	0.31	− 9.95	1.10	1.77
NF$_3$	N–F	− 0.80	1.84	− 11.98	0.10	2.15
O$_3$	O–O	− 7.98	0.19	− 8.28	0.00	1.44
CF$_4$	C–F	− 1.26	− 1.37	− 13.17	0.03	2.17
SF$_6$	S–F	− 9.99	5.95	− 22.72	0.06	3.20
SO$_2$	S–O	− 4.23	3.95	− 21.02	1.88	2.51

### Energetics of host–guest interaction in GHGs encapsulated hydrate clathrate

The energy stability of GHGs encapsulated in clathrate hydrates was evaluated through interaction energy ($\Delta E_{\text {int}}$) calculations, with the results presented in Tables 1, 2 and 3. This study is limited to the encapsulation of a single guest molecule, and the observed $\Delta E_{\text {int}}$ trends are influenced by the size, polarity, and polarizability of the molecules. The encapsulation process within clathrate cages not only modifies the interaction energy but also holds significant environmental implications by mitigating the detrimental effects of these guest molecules. since CCl$_4$ is not stable inside those cages, yet they experience considerable stabilization in the larger 5$^{12}$6$^4$ cage. Notably, CCl$_4$’s interaction energy becomes much more favorable, shifting from a weakly stabilizing 2.25 kcal/mol in the 5$^{12}$ cage to a strongly exothermic $-25.41$ kcal/mol in the 5$^{12}$6$^4$ cage, highlighting the enhanced dispersion-driven stabilization in larger cavities. Halogenated methanes like CH$_3$Br, CH$_3$Cl, and CH$_3$F continue to exhibit highly negative interaction energies across all cages, although CH$_3$Br shows slightly diminished binding with increasing cage size (from $-31.27$ to $-22.88$ kcal/mol), suggesting a saturation of stabilizing interactions in larger hosts. Methane (CH$_4$), while weakly polarizable, exhibits consistent but decreasing interaction strength (from $-14.93$ to $-9.99$ kcal/mol), reflecting limited dispersion-driven binding. CO$_2$ and CO also show weakened interactions in larger cages, with CO$_2$ dropping from $-16.84$ kcal/mol in 5$^{12}$ to $-10.74$ kcal/mol in 5$^{12}$6$^4$, and CO similarly declining from $-12.40$ to $-8.19$ kcal/mol, suggesting that smaller cages may facilitate closer contact and stronger interactions for these linear molecules. Sulfur-containing gases such as H$_2$S and SO$_2$ remain among the most strongly interacting species, especially in smaller cages (H$_2$S: $-22.12$ kcal/mol in 5$^{12}$; SO$_2$: $-31.76$ kcal/mol), although interaction strength declines with increasing cage size due to reduced confinement-enhanced binding. Among fluorinated gases, SF$_6$ and CF$_4$ show robust stabilization driven by dispersion forces. SF$_6$, in particular, reveals strong interaction in the 5$^{12}$6$^2$ cage ($-25.76$ kcal/mol) and slightly weaker but still substantial stabilization in 5$^{12}$6$^4$ ($-22.72$ kcal/mol). Overall, the inclusion of dispersion corrections accentuates the role of van der Waals forces in stabilizing guest–host complexes, particularly for large, polarizable guests, and reshapes the perceived encapsulation efficiencies across different hydrate cage types. Overall, encapsulation within clathrate hydrates leads to more substantial stabilization of guest molecules, especially in the 5$^{12}$6$^4$ cages, thereby reducing their mobility and reactivity and mitigating their environmental effects. The calculated interaction energies for encapsulated CH$_4$ and CO$_2$ align well with literature values, confirming the accuracy of our confinement models^76^. Additionally, reactivity indices such as the energy gap, chemical hardness, electronegativity, and electrophilicity quantitative measures of molecular stability are provided in the supporting information (Tables S14–S16 and Figs. S38–S42).

#### Nature of interaction

#### Natural bond orbital analysis (NBO)

NBO analysis is a pivotal technique for exploring inter- and intra-molecular interactions arising from chemical bonds within a molecule or compound. This method provides comprehensive insights into both occupied and unoccupied orbitals, enabling a nuanced understanding of bonding and anti-bonding characteristics. As presented in Tables S1–S4 of the supporting information, the analysis reveals the bonding and anti-bonding occupancies alongside their respective s and p orbital contributions of GHGs. The NBO analysis uncovers notable trends in the bonding and anti-bonding interactions of guest molecules encapsulated within hydrate clathrate cages. These encapsulations lead to systematic alterations in orbital contributions, occupancy patterns, and anti-bonding interactions, highlighting the influence of the distinct cage environment on the molecular interactions. For molecules such as CCl$_4$, CF$_2$Cl$_2$, and CH$_3$Cl, the C–Cl bonds show an increase in anti-bonding ($\sigma ^*$) occupancy, suggesting a slight weakening of the bond while retaining the overall bonding character. This trend may reduce their ozone-depleting potential. Similarly, the C–H bonds in CH$_3$Br, CH$_3$Cl, and CH$_4$ exhibit increased anti-bonding occupancy, which tends to decrease bond stability. In the case of CO$_2$, encapsulation leads to a redistribution of s- and p-character in C–O bonds, altering their bonding nature and potentially moderating their vibrational properties, which could mitigate their greenhouse effect. For molecules such as NF$_3$, increased anti-bonding interactions result in weaker bonds, and in SO$_2$, decreased s-character of the S–O bond compared to its monomer indicates a stronger interaction with the host cage. The occupancy data further show a significant decrease in the anti-bonding occupancy of the S–H bond in H$_2$S, suggesting a stronger hydrogen bonding interaction. In contrast, CO and O$_3$ hydrates exhibit occupancy values similar to their monomers, indicating weaker interactions. These observations suggest that SO$_2$ and H$_2$S are more easily trapped within the host cage, whereas the encapsulation of CO and O$_3$ is less favorable.

**Fig. 6:**
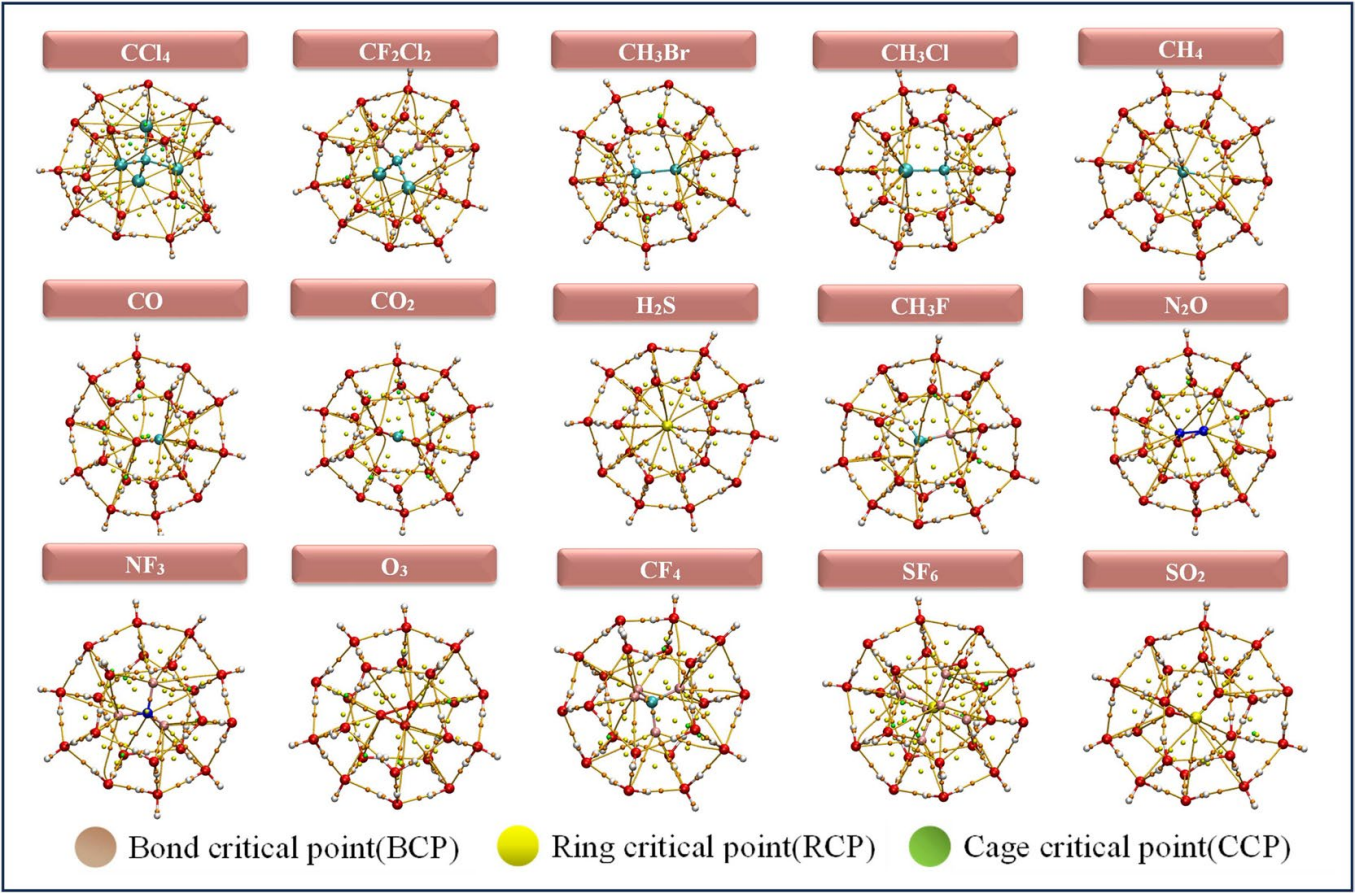
Visualization of QTAIM bond critical points for selected greenhouse gases encapsulated within the $5^{12}$ clathrate hydrate cages.

**Fig. 7:**
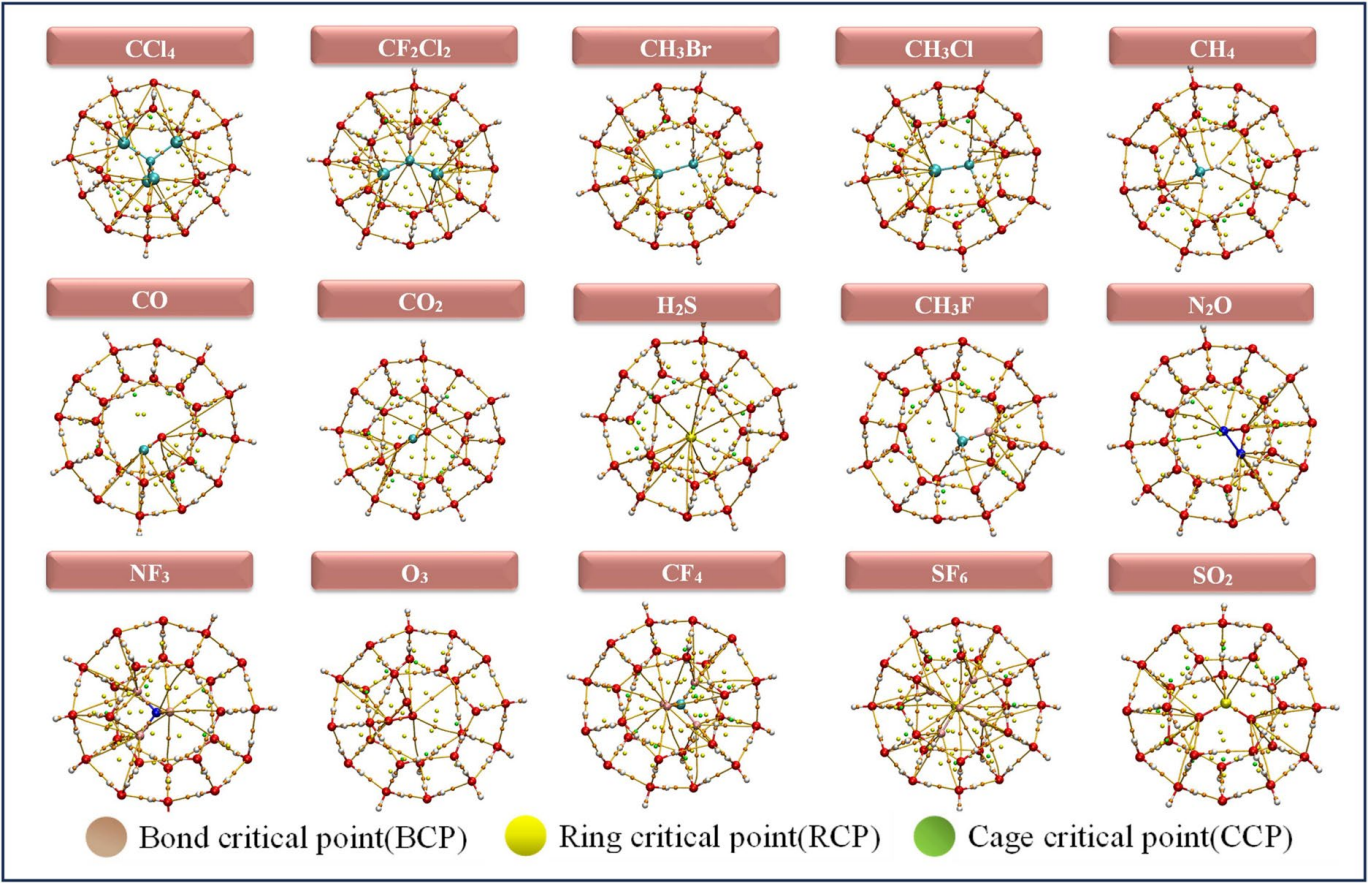
Visualization of QTAIM bond critical points for selected greenhouse gases encapsulated within the $5^{12}6^{2}$ clathrate hydrate cages.

#### Atoms in molecule analysis (AIM)

**Fig. 8:**
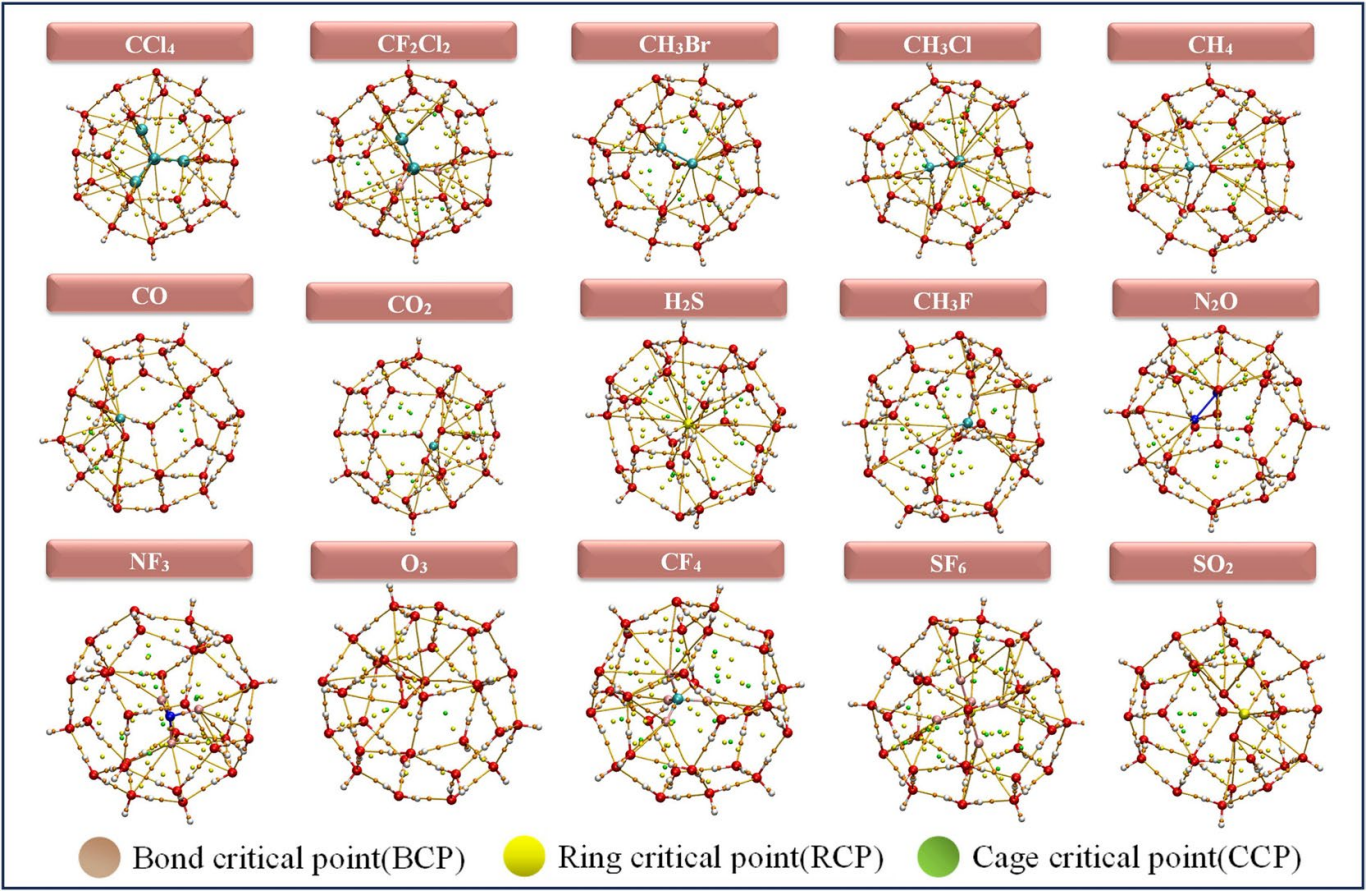
Visualization of QTAIM bond critical points for selected greenhouse gases encapsulated within the $5^{12}6^{4}$ clathrate hydrate cages.

The Quantum Theory of Atoms in Molecules (QTAIM), initially developed by Richard Bader, enables the study of topology analysis through the examination of electron density distribution^77^. This distribution represents the likelihood of electrons being found across the region influenced by the attractive field created by the nuclei. The QTAIM coordinates, assessed at the Bond Critical Point (BCP) the saddle point show the highest value of the distributed electron density along the Bond Path (BP) connecting atoms in guest molecules. BCPs are (3,–1) critical points located between two atoms forming a hydrogen bond. Ring Critical Points (RCPs), defined as (3,+1) critical points, confirm the presence of polygonal ring structures (e.g., 5- or 6-membered rings) within the clathrate hydrate framework formed through hydrogen bonding. Typically, the presence of 12, 14, and 16 RCPs in $5^{12}$, $5^{12}6^2$, and $5^{12}6^4$ clathrate hydrate cages, respectively, confirms the existence of 12, 14, and 16 distinct ring faces, reflecting the ideal polyhedral geometry of these cages. A (3,+3) critical points also known as Cage Critical Points (CCPs) or void critical points, indicate the presence of an internal cavity. The position and displacement of the CCP upon guest encapsulation provide insight into how the guest molecule is situated whether centered or off-center within the cage. Figures 6, 7 and 8 represents the BCPs, RCPs and CCPs generated from the QTAIM analysis of the complexes. According to Table S5 to S8 in the supporting information, the negative values of $\nabla ^2 \rho (\textbf{r})$ for all systems suggest the bonds are covalent. We employed QTAIM to analyze the chemical bonding characteristics of the GHGs after they were encapsulated within the hydrate clathrate cages. For all guest molecules and their bonds in the clathrate cages, ellipticity: $\epsilon < 0$, indicating stable bond interaction. Encapsulation within clathrate hydrates induces minor modifications in QTAIM parameters, predominantly preserving or slightly stabilizing covalent bonds, while non-covalent and mixed bonds exhibit minimal weakening or stabilization. The stability of bonds, as evidenced by low ellipticity values, ensures that the encapsulated molecules remain confined and less reactive. QTAIM analysis reveals that guest–host interactions in clathrate hydrates are primarily weak, non-covalent in nature, dominated by hydrogen bonding and dispersion forces (Table S9). Positive Laplacian values confirm closed-shell interactions, with stronger binding observed for polar molecules like H$_2$S and SO$_2$. Nonpolar guests such as CH$_4$ and CO exhibit weaker interactions, especially in larger cages due to a looser spatial fit. Overall, stabilization arises from O–H$\cdots$X (X = F, Cl, O, S, H) contacts, modulated by guest polarity and cage geometry.

**Fig. 9:**
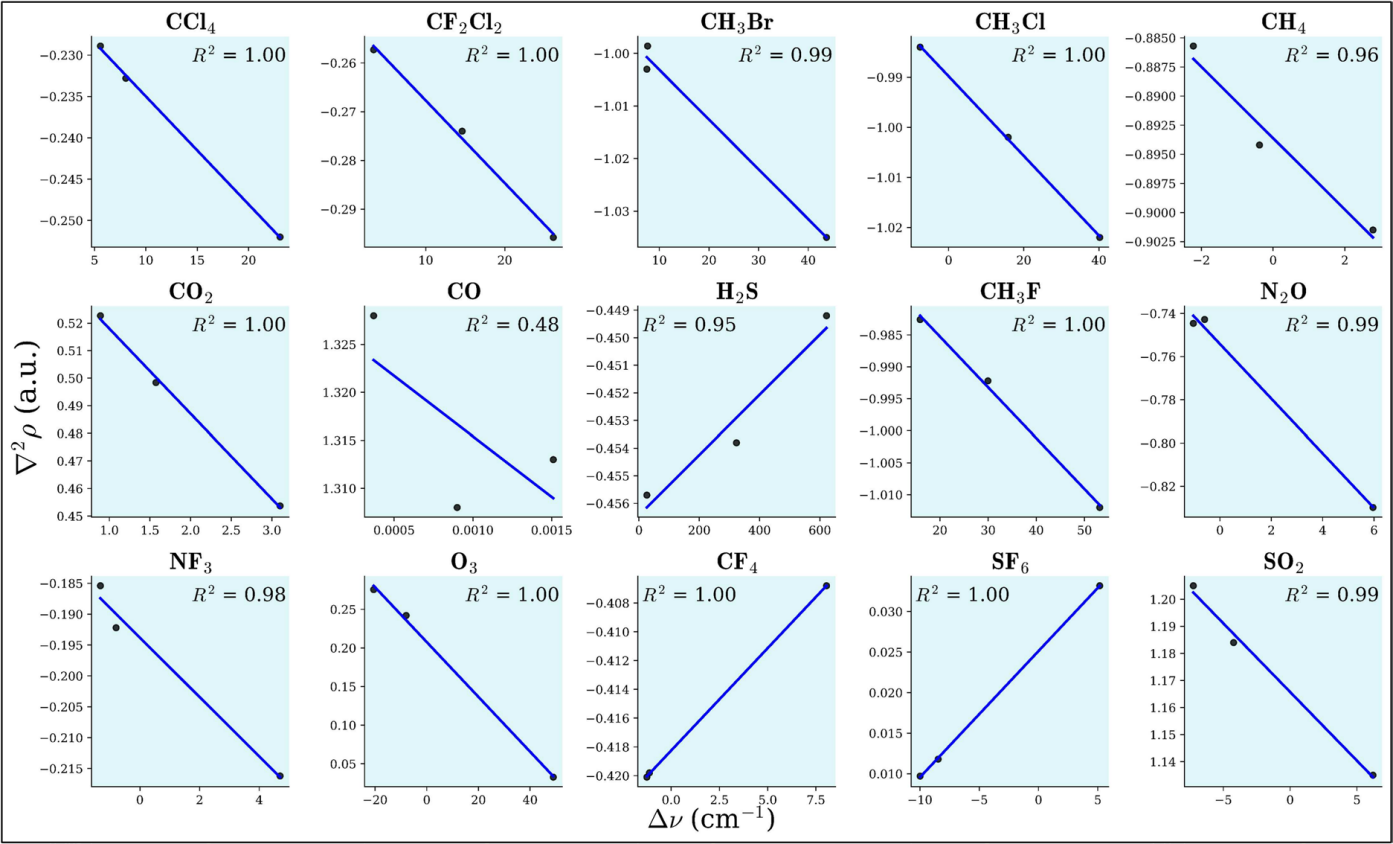
Plot for the relation between $\Delta \nu$ (in cm$^{-1}$) v/s $\nabla ^2\rho (r)$ (in a.u.) for the GHGs encapsulated in 5$^{12}$, 5$^{12}6^2$ and $5^{12}6^4$ clathrate cages. For CH$_3$F, CH$_3$Cl, and CH$_3$Br, the C–H bond is considered, whereas for CF$_2$Cl$_2$, the C–F bond is taken into consideration.

**Fig. 10:**
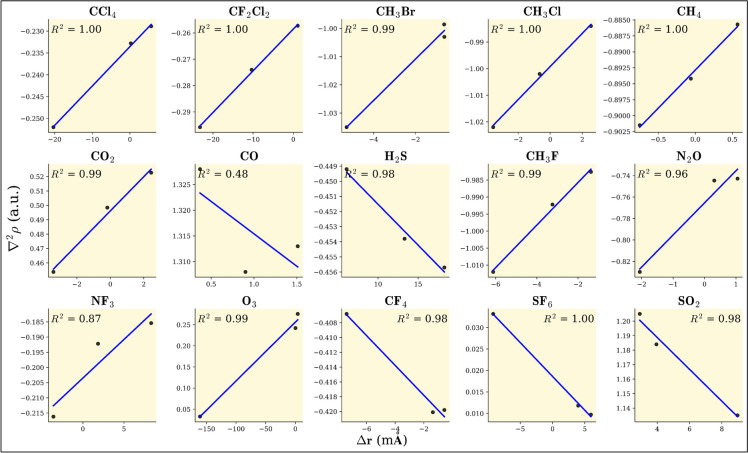
Plot for the relation between $\Delta r$ (in mÅ) v/s $\nabla ^2\rho (r)$ (in a.u.) for the GHGs encapsulated in 5$^{12}$, 5$^{12}6^2$ and $5^{12}6^4$ clathrate cages. For CH$_3$F, CH$_3$Cl, and CH$_3$Br, the C–H bond is considered, whereas for CF$_2$Cl$_2$, the C–F bond is taken into consideration.

The relationship between stretching vibrational frequency change and Laplacian electron density in clathrate hydrate cages reveals electronic structure and molecular interaction effects. As shown in Fig. 9 , most molecules exhibit an inverse linear trend (negative slope), where increasing $\nabla ^2 \rho (\textbf{r})$ weakens bond strength, dispersing electron density at the bond critical point. We observe a deviation for CO because oxygen exhibits a partial positive charge due to resonance effects, lone-pair donation from carbon, and the overall charge distribution within the molecule. Additionally, CO has a very small molecular size compared to other gases (from Tables 1, 2 and 3). In the smaller cage, CO fits well, allowing for stronger interactions. However, in the larger cage, the increased cavity size causes CO to become off-centered, which may explain this deviation. Additionally, NBO and AIM analyses reveal that the 5$^{12}$6$^{2}$ cage exhibits higher second-order perturbation energy and lower total electron density compared to the 5$^{12}$6$^{4}$ cage, further supporting this unusual behavior. Figure 10 illustrates guest-cage electronic interactions, where most molecules show a positive slope ($R^2 > 0.9$) indicating that increasing $\nabla ^2\rho (r)$ weakens bonds, leading to elongation. However, CF$_2$Cl$_2$, H$_2$S, CF$_4$, SF$_6$, and SO$_2$ exhibit bond contraction (negative slope), suggesting localized stabilization mechanisms. Moreover, higher electron density at the BCP for CH$_3$F, H$_2$S, and SO$_2$ suggests greater stabilization due to enhanced donor–acceptor interactions. In contrast, CO and O$_3$ clathrates exhibit lower electron density, indicating weaker hydrogen-bonding interactions.

#### Non-covalent interaction analysis and energy decomposition analysis

A crucial factor in stabilizing inclusion compounds like clathrate hydrates is the noncovalent interaction (NCI) between the host and guest molecules. We performed NCI analysis using the reduced density gradient (RDG) approach, which enables direct visualization of NCIs within a molecular system. This method provides specific criteria for characterizing NCIs, including H-bonding, steric interactions, and vdW interactions between the components of the molecular system. Figure S34 given in supporting information represents the RDG scatterplot and corresponding iso-surface map for the guest molecules encased within the three hydrate cages. The map reveals that the cage structures are stabilized by both hydrogen bonding (blue regions) and vdW (green regions) interactions. The less prominent inverted peak structures for $\text {sign}(\lambda _2) \rho (\vec {r}) \le -0.30 \, \text {a.u.}$ correspond to hydrogen bonding, which becomes more pronounced as the cage size increases, indicating an enhancement in hydrogen bonding interactions. Furthermore, the appearance of two distinct troughs (inverted peaks) on either side of $\text {sign}(\lambda _2) \rho (\vec {r}) = 0$ indicates that both the attractive and repulsive components of vdW interactions play a role in the stability of the cages. However, these troughs diminish in prominence with increasing cage size, reflecting the anticipated weakening of the vdW interactions between the guest molecule and the cage. The RDG plots indicate that in the case of the 5$^{12}$ cage, CHF$_3$, SO$_2$, and H$_2$S exhibit a larger H-bonding region, whereas CCl$_4$ and CF$_2$Cl$_2$ show comparatively less. This observation is further supported by AIM and NBO analyses.

**Fig. 11:**
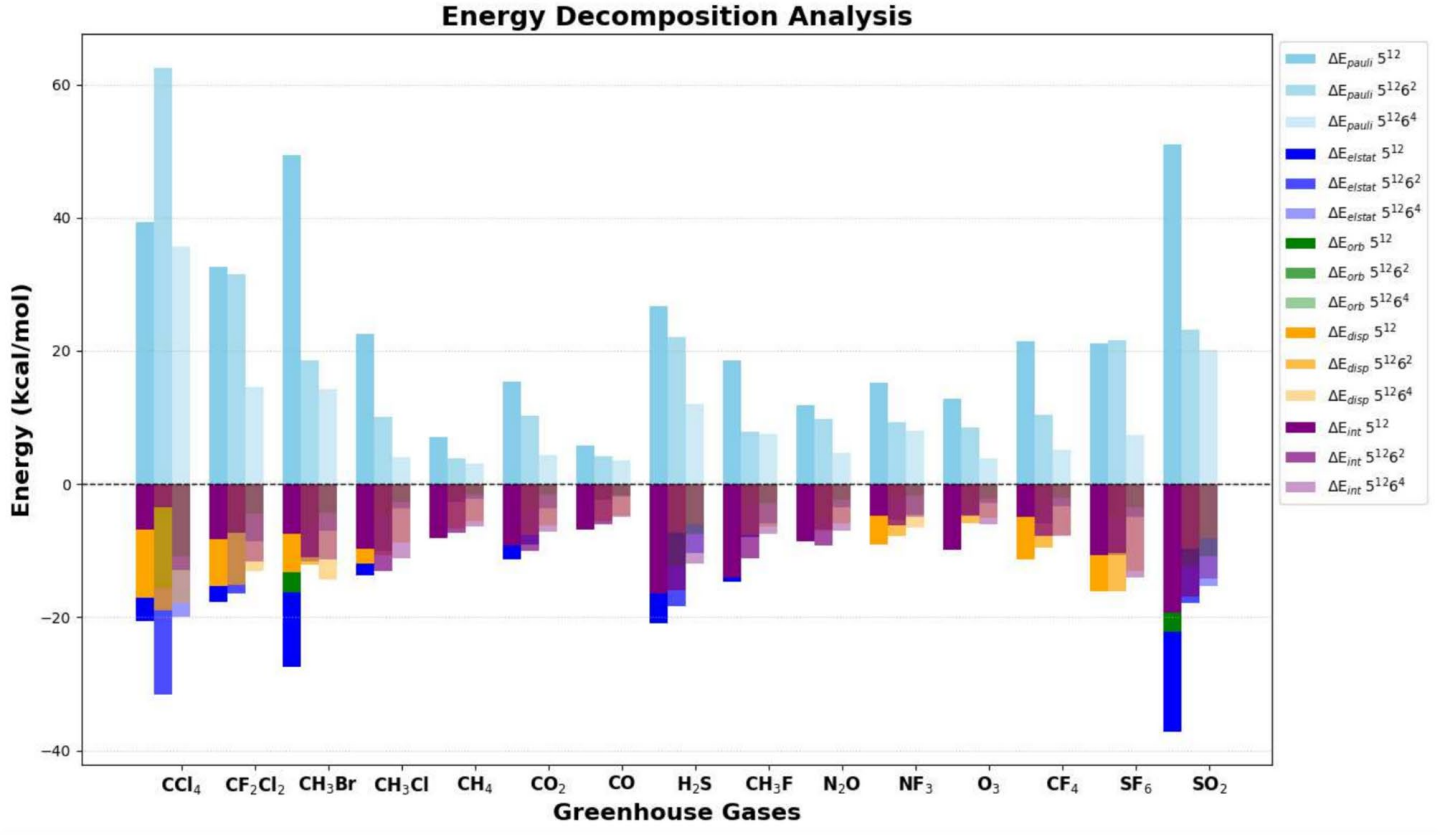
Bar plot for the energy decomposition analysis of GHGs encapsulated within $5^{12}$, $5^{12}6^2$ and $5^{12}6^4$ clathrate hydrates.

The energy decomposition analysis (EDA) results offer comprehensive insights into the interactions between greenhouse gas molecules and clathrate hydrate cages ($5^{12}$, $5^{12}6^2$, and $5^{12}6^4$) using the Amsterdam Density Functional (ADF) software. These interactions are divided into four fundamental energy components: Pauli repulsion ($\Delta E_{\text {pauli}}$), electrostatic interaction ($\Delta E_{\text {elstat}}$), orbital interaction ($\Delta E_{\text {orb}}$), and dispersion interaction ($\Delta E_{\text {disp}}$), culminating in the total interaction energy ($\Delta E_{\text {int}}$, as represented in Table 4 and Fig. 11). Pauli repulsion represents steric hindrance arising from electron cloud overlap, with higher values indicating stronger repulsion and subsequent system destabilization. The intermediate $5^{12}6^2$ cage exhibits the highest Pauli repulsion for most molecules, particularly CCl$_4$ (62.56 kcal/mol) and SO$_2$ (23.13 kcal/mol), suggesting spatial constraints within these cages. Electrostatic interaction reflects Coulombic attraction between host and guest molecules, where more negative values indicate stronger stabilization. Notably, SO$_2$ (− 37.11 kcal/mol in $5^{12}$) and H$_2$S (− 20.92 kcal/mol in $5^{12}$) exhibit significant electrostatic stabilization, which diminishes in larger cages due to reduced confinement effects. Orbital interaction accounts for charge transfer and polarization effects, with more negative values signifying stronger electronic stabilization through orbital overlap. For instance, SO$_2$ (− 22.22 kcal/mol in $5^{12}$) and CH$_3$Br (− 16.28 kcal/mol in $5^{12}$) display substantial orbital interactions. These interactions decrease in larger cages, underscoring the role of confinement in enhancing host–guest interactions. Dispersion interaction, which accounts for van der Waals forces, primarily stabilizes nonpolar and weakly polar molecules such as SF$_6$ (− 16.06 kcal/mol in $5^{12}$) and CCl$_4$ (− 17.06 kcal/mol in $5^{12}$). While dispersion effects remain relatively consistent across different cage sizes, they slightly diminish in the largest cages due to increased guest mobility. The total interaction energy ($\Delta E_{\text {int}}$) sums these contributions to determine the overall stability of guest molecules within clathrate hydrates. SO$_2$ (− 19.37 kcal/mol in $5^{12}$) and H$_2$S (− 16.36 kcal/mol in $5^{12}$) exhibit the most stable interactions. Molecular suitability for specific cages varies based on these interactions. Strongly interacting molecules, such as SO$_2$, CH$_3$Br, and H$_2$S, exhibit high electrostatic and orbital interactions, rendering them most stable in $5^{12}$ cages, although their stability decreases in larger cages. Conversely, weakly interacting molecules like CH$_4$, CO, and CO$_2$ display minimal interaction energy across all cages, indicating weak encapsulation and a necessity for additional stabilization factors such as pressure. Large, nonpolar molecules, including CCl$_4$, SF$_6$, and CF$_4$, benefit from dispersion forces and exhibit greater stability in larger cages ($5^{12}6^4$) due to reduced steric hindrance. Among the three clathrate cage types, the smallest ($5^{12}$) provides the strongest stabilization due to enhanced electrostatic and orbital interactions, making it ideal for small, polar molecules like SO$_2$ and H$_2$S, although steric repulsion may hinder the encapsulation of larger molecules. Overall, the intermediate $5^{12}6^2$ cage exhibits high Pauli repulsion, reducing guest–host compatibility and rendering it less favourable. The larger cage ($5^{12}6^4$) offers greater spatial accommodation, reducing steric repulsion but also weakening electrostatic and orbital interactions, making it more suitable for bulky, nonpolar molecules such as CCl$_4$ and SF$_6$. In conclusion, molecular stability within clathrate hydrates is dictated by a balance of steric effects, electrostatic interactions, orbital interactions, and dispersion forces. The interplay between these factors determines the feasibility of different guest molecules within specific clathrate cage structures.

**Table 4:** Energy decomposition analysis of GHGs encapsulated in $5^{12}$, $5^{12}6^2$, and $5^{12}6^4$ hydrate clathrates. All values are in kcal/mol.

Greenhouse	$\Delta E_{\text {Pauli}}$			$\Delta E_{\text {elstat}}$			$\Delta E_{\text {orb}}$			$\Delta E_{\text {disp}}$			$\Delta E_{\text {int}}$		
Gases	$5^{12}$	$5^{12}6^2$	$5^{12}6^4$	$5^{12}$	$5^{12}6^2$	$5^{12}6^4$	$5^{12}$	$5^{12}6^2$	$5^{12}6^4$	$5^{12}$	$5^{12}6^2$	$5^{12}6^4$	$5^{12}$	$5^{12}6^2$	$5^{12}6^4$
CCl$_4$	39.36	62.56	35.64	− 20.63	− 31.56	− 19.88	− 8.56	− 15.61	− 10.83	− 17.06	− 18.92	− 17.81	− 6.89	− 3.53	− 12.88
CF$_2$Cl$_2$	32.61	31.56	14.56	− 17.74	− 16.50	− 8.58	− 7.79	− 7.25	− 4.50	− 15.27	− 15.19	− 13.14	− 8.20	− 7.37	− 11.67
CH$_3$Br	49.40	18.58	14.26	− 27.48	− 11.65	− 6.99	− 16.28	− 5.87	− 4.26	− 13.16	− 12.03	− 14.30	− 7.53	− 10.98	− 11.30
CH$_3$Cl	22.50	10.09	3.96	− 13.64	− 10.09	− 3.64	− 6.67	− 4.40	− 2.72	− 11.91	− 10.65	− 8.69	− 9.72	− 13.05	− 11.09
CH$_4$	7.11	3.90	3.00	− 4.36	− 2.62	− 2.24	− 2.70	− 1.93	− 1.62	− 8.15	− 6.64	− 5.57	− 8.10	− 7.29	− 6.43
CO$_2$	15.37	10.28	4.31	− 11.27	− 9.02	− 3.59	− 4.42	− 3.62	− 1.64	− 8.84	− 7.65	− 6.21	− 9.16	− 10.00	− 7.14
CO	5.74	4.16	3.48	− 3.42	− 2.35	− 1.79	− 2.72	− 2.31	− 1.89	− 6.49	− 5.50	− 4.74	− 6.89	− 6.00	− 4.94
H$_2$S	26.66	22.02	12.00	− 20.92	− 18.38	− 10.32	− 13.94	− 12.29	− 7.52	− 8.15	− 7.29	− 6.09	− 16.36	− 15.94	− 11.94
CH$_3$F	18.58	7.88	7.59	− 14.59	− 8.03	− 5.83	− 8.84	− 3.48	− 2.84	− 9.15	− 7.58	− 6.36	− 14.00	− 11.20	− 7.45
N$_2$O	11.80	9.72	4.62	− 7.23	− 6.88	− 3.42	− 5.19	− 5.19	− 2.40	− 8.01	− 6.94	− 5.84	− 8.62	− 9.29	− 7.04
NF$_3$	15.27	9.31	7.94	− 8.00	− 5.41	− 4.55	− 2.94	− 2.24	− 1.80	− 9.06	− 7.79	− 6.53	− 4.74	− 6.13	− 4.93
O$_3$	12.76	8.57	3.87	− 7.46	− 4.55	− 2.82	− 7.78	− 2.90	− 2.13	− 7.44	− 5.84	− 5.04	− 9.91	− 4.72	− 6.11
CF$_4$	21.39	10.47	5.14	− 10.87	− 5.93	− 3.39	− 4.09	− 2.85	− 2.04	− 11.29	− 9.55	− 7.59	− 4.86	− 7.86	− 7.87
SF$_6$	21.13	21.57	7.43	− 10.79	− 10.75	− 4.91	− 4.90	− 5.12	− 3.54	− 16.06	− 16.09	− 13.05	− 10.62	− 10.38	− 14.08
SO$_2$	50.99	23.13	20.16	− 37.11	− 17.93	− 15.37	− 22.22	− 12.33	− 10.85	− 11.03	− 9.79	− 8.10	− 19.37	− 16.92	− 14.17

## Limitations and outlook

The current study utilizes gas-phase DFT, provides insightful theoretical predictions of guest–host interaction in clathrate hydrates but has some limitations that must be taken into account. The calculations are not comprehensive of lattice dynamics and external environmental conditions, both of which play a critical role in encapsulation behavior and vibrational characterization. Additionally, the rigid-cage approximation is not inclusive of host lattice flexibility and solvent influence, which can influence the landscape of interaction in real systems. Future work could involve periodic DFT calculations and ab initio molecular dynamics simulations to include solid-state and thermal influences, yielding an improved understanding of the stability of clathrates and gas sequestration under realistic conditions. Experimental verification and calibration based on spectroscopic or crystallographic evidence would render the outcome more beneficial to environmental applications.

## Conclusion

In conclusion, DFT studies on the encapsulation of GHGs within clathrate hydrate cages reveal minimal structural changes. However, confinement induces both attractive and repulsive vdW interactions, enhancing guest stability through hydrogen bonding. NCI analysis confirms that weak interactions become less prominent in larger cages, while smaller cages impose steric constraints, leading to bond distortions. Intermediate cages balance spatial constraints and asymmetry, influencing host–guest interactions. Vibrational shifts correlate with confinement effects, with smaller cages causing blue-shifts due to stronger interactions and larger cages leading to red-shifts or minimal changes. Based on EDA’s $\Delta E_{\text {int}}$ parameter, it is evident that among all the guest molecules, CH$_3$F, SO$_2$, H$_2$S, and CH$_3$Br exhibit high stability in 5$^{12}$ cages, whereas CF$_2$Cl$_2$ and CCl$_4$ are unstable in both 5$^{12}$ and 5$^{12}$6$^{2}$ cages. This observation is further supported by QTAIM, NBO, and NCI analyses. These topological analyses indicate that CH$_3$F, CH$_3$Br, H$_2$S, CH$_4$, CO, and O$_3$ are well-suited for smaller cages. Meanwhile, CO$_2$, CH$_3$Cl, N$_2$O, NF$_3$, and SO$_2$ are compatible with both small and intermediate cages, whereas CCl$_4$, CF$_2$Cl$_2$, and SF$_6$ exhibit stability in larger cages. This work highlights the potential of clathrate hydrates as agents for environmental remediation through the integration of structural, vibrational, and energetic studies. The correlations obtained between vibrational changes, interaction energies, and electron density variations demonstrate their ability to neutralize toxic gases via selective encapsulation. These findings offer a strong theoretical basis for environmental advantages such as CO$_2$ and CH$_4$ sequestration, control of ozone-depleting species like CF$_2$Cl$_2$ and CCl$_4$, and acid rain precursors such as SO$_2$ and H$_2$S. Such results provide predictive insights into the behavior of gases within clathrate systems and propose useful strategies for pollutant control. Experimental verification and extensive testing should be conducted to assess real-world effectiveness. The ability of clathrates to stabilize poisonous molecules through tailored encapsulation presents significant potential to reduce atmospheric reactivity, enhance sequestration, and decelerate climate change.

## Methods

We performed a computational investigation into the encapsulation and stability of clathrate hydrate cages and their inclusion complexes with GHGs, utilizing advanced calculations implemented through the Gaussian 16 software package (Fig. 1)^78^. Optimization of the clathrate hydrates was computationally demanding, often requiring hundreds of steps to achieve convergence, with the B3LYP/6-31G(d) method proving suitable for this purpose.The B3LYP functional has incorporated with Grimme’s D3(BJ) dispersion correction

Interaction energies ($\Delta E$) were calculated using the formula:

1 $$\begin{aligned} \Delta E = E_{AB} - (E_A + E_B), \end{aligned}$$

where $E_{AB}$ is the energy of the complex, and $E_A$ and $E_B$ are the energies of the individual monomers.

To quantify electron populations in bonding and anti-bonding orbitals, NBO analysis was performed. QTAIM analysis was employed to examine the bonding nature of the guest molecules, with key parameters such as electron density ($\rho (r)$), the Laplacian of electron density ($\nabla ^2\rho (r)$), and kinetic energy (*G*(*r*)) providing insight into the interactions, distinguishing covalent and non-covalent character^79,80^. AIM analysis was conducted using Multiwfn and Visual Molecular Dynamics (VMD) software^81,82^. Non-Covalent Interaction (NCI) analysis, based on Reduced Density Gradient (RDG) and eigenvalue analysis, revealed the details of weak and strong non-covalent interactions stabilizing the guest molecules in the hydrate clathrate cages. Electron Localized Function (ELF) analysis offered a three-dimensional representation of electron density localization at the guest–host interface.(Given in supporting information) The Energy Decomposition Analysis (EDA) was performed using the generalized gradient approximation:Perdew–Burke–Ernzerhof (GGA:PBE-D3) functional with a triple zeta plus polarization (TZP) basis set in the Amsterdam Density Functional (ADF) program package^83^.

## Supplementary Information

Supplementary Information 1.

## Data Availability

The datasets used or analysed during the current study are available from the supporting information.

## References

[1] Thomas, J. D. L., Thomas, M. G., McMullan, R. K., Hinds, J. P. & Leach, S. E. Thermodynamics and phase behavior of clathrate hydrates. *Nature* **409**, 539–542 (2001).11206553

[2] Hashimoto, H., Yamaguchi, T., Ozeki, H. & Muromachi, S. Structure-driven CO selectivity and gas capacity of ionic clathrate hydrates. *Sci. Rep.* **7**, 17216 (2017). 29222487 10.1038/s41598-017-17375-1 PMC5722917

[3] Buffett, B. A. Clathrate hydrates. *Annu. Rev. Earth Planet. Sci.* **28**, 477–507 (2000).

[4] Hesselbo, S. P. et al. Massive dissociation of gas hydrate during a Jurassic oceanic anoxic event. *Nature* **406**, 392–395 (2000). 10935632 10.1038/35019044

[5] Fehn, U., Snyder, G. & Egeberg, P. K. Dating of pore waters with 129i: Relevance for the origin of marine gas hydrates. *Science* **289**, 2332–2335 (2000). 11009415 10.1126/science.289.5488.2332

[6] Lan, X., Chen, J., Li, D., Zheng, J. & Linga, P. Gas storage via clathrate hydrates: Advances, challenges, and prospects. *Gas Sci. Eng.* **129**, 205388 (2024).

[7] Patt, A., Simon, J.-M., Picaud, S. & Salazar, J. M. A grand canonical Monte Carlo study of the N, CO, and mixed N-CO clathrate hydrates. *J. Phys. Chem. C* **122**, 18432–18444 (2018).

[8] Ota, M., Abe, Y., Watanabe, M., Smith, R. L. Jr. & Inomata, H. Methane recovery from methane hydrate using pressurized CO. *Fluid Phase Equilib.* **228**, 553–559 (2005).

[9] Kumar, A., Sakpal, T., Roy, S. & Kumar, R. Methane hydrate formation in a test sediment of sand and clay at various levels of water saturation. *Can. J. Chem.* **93**, 874–881 (2015).

[10] Davidson, D., Handa, Y., Ratcliffe, C., Tse, J. & Powell, B. The ability of small molecules to form clathrate hydrates of structure II. *Nature* **311**, 142–143 (1984).

[11] Ghosh, J., Vishwakarma, G., Kumar, R. & Pradeep, T. Formation and transformation of clathrate hydrates under interstellar conditions. *Acc. Chem. Res.* **56**, 2241–2252 (2023). 37531446 10.1021/acs.accounts.3c00317

[12] Ghosh, J. et al. Clathrate hydrates in interstellar environment. *Proc. Natl. Acad. Sci.* **116**, 1526–1531 (2019). 30630945 10.1073/pnas.1814293116 PMC6358667

[13] Ghosh, J., Bhuin, R. G., Vishwakarma, G. & Pradeep, T. Formation of cubic ice via clathrate hydrate, prepared in ultrahigh vacuum under cryogenic conditions. *J. Phys. Chem. Lett.* **11**, 26–32 (2019). 31804833 10.1021/acs.jpclett.9b03063

[14] Tychengulova, A. et al. Laboratory studies of the clathrate hydrate formation in the carbon dioxide-water mixtures at interstellar conditions. *ACS Omega* **10**, 1237–1248 (2024). 39829442 10.1021/acsomega.4c08342 PMC11740112

[15] Lunine, J. I. & Stevenson, D. J. Thermodynamics of clathrate hydrate at low and high pressures with application to the outer solar system. *Astrophys. J. Suppl. Ser.* **58**, 493–531 (1985).

[16] Schneeberger, A., Mousis, O., Aguichine, A. & Lunine, J. I. Evolution of the reservoirs of volatiles in the protosolar nebula. *Astron. Astrophys.* **670**, A28 (2023).

[17] Miller, S. L. & Smythe, W. D. Carbon dioxide clathrate in the Martian ice cap. *Science* **170**, 531–533 (1970). 17799706 10.1126/science.170.3957.531

[18] Buhler, P., Ingersoll, A., Piqueux, S., Ehlmann, B. & Hayne, P. Coevolution of Mars’s atmosphere and massive south polar CO ice deposit. *Nat. Astron.* **4**, 364–371 (2020).

[19] Dartois, E. Co clathrate hydrate: Near to mid-ir spectroscopic signatures. *Icarus* **212**, 950–956 (2011).

[20] Luspay-Kuti, A. et al. The presence of clathrates in comet 67p/churyumov-gerasimenko. *Sci. Adv.* **2**, e1501781 (2016). 27152351 10.1126/sciadv.1501781 PMC4846445

[21] Marboeuf, U., Thiabaud, A., Alibert, Y., Cabral, N. & Benz, W. From stellar nebula to planetesimals. *Astron. Astrophys.* **570**, A35 (2014).

[22] Makogon, Y. F. Natural gas hydrates-a promising source of energy. *J. Nat. Gas Sci. Eng.* **2**, 49–59 (2010).

[23] Rai, S. & Rai, D. Electric field influence on CO clathrate hydrates. *J. Phys. Chem. A* **128**, 9596–9605 (2024). 39442922 10.1021/acs.jpca.4c05074

[24] Aghajanloo, M., Yan, L., Berg, S., Voskov, D. & Farajzadeh, R. Impact of CO hydrates on injectivity during CO storage in depleted gas fields: A literature review. *Gas Sci. Eng.* **205**, 250 (2024).

[25] Fortes, A. D. & Choukroun, M. Phase behaviour of ices and hydrates. *Space Sci. Rev.* **153**, 185–218 (2010).

[26] Sloan, E. D. Jr. & Koh, C. A. *Clathrate Hydrates of Natural Gases* (CRC Press, 2007).

[27] Davidson, D. et al. A clathrate hydrate of carbon monoxide. *Nature* **328**, 418–419 (1987).

[28] Florusse, L. J. et al. Stable low-pressure hydrogen clusters stored in a binary clathrate hydrate. *Science* **306**, 469–471 (2004). 15486295 10.1126/science.1102076

[29] Lee, H. et al. Tuning clathrate hydrates for hydrogen storage. *Nature* **434**, 743–746 (2005). 15815624 10.1038/nature03457

[30] Loveday, J. et al. Stable methane hydrate above 2 gpa and the source of titan’s atmospheric methane. *Nature* **410**, 661–663 (2001). 11287946 10.1038/35070513

[31] Yoro, K. O. & Daramola, M. O. CO emission sources, greenhouse gases, and the global warming effect. In *Advances in Carbon Capture*, 3–28 (Elsevier, 2020).

[32] Yan, X., Li, Y., Zhao, J. & Wang, Z. Density functional theory study on CO adsorption by CE-promoted CaO in the presence of steam. *Energy Fuels* **34**, 6197–6208 (2020).

[33] Liu, W. et al. Assessment of hydrate blockage risk in long-distance natural gas transmission pipelines. *J. Nat. Gas Sci. Eng.* **60**, 256–270 (2018).

[34] Pitts, J. Jr. & Finlayson, B. Mechanisms of photochemical air pollution. *Angew. Chem. Int. Ed. Engl.* **14**, 1–15 (1975). 804281 10.1002/anie.197500011

[35] Hu, Z., Lee, J. W., Chandran, K., Kim, S. & Khanal, S. K. Nitrous oxide (NO) emission from aquaculture: A review. *Environ. Sci. Technol.* **46**, 6470–6480 (2012). 22594516 10.1021/es300110x

[36] Sherry, D., McCulloch, A., Liang, Q., Reimann, S. & Newman, P. A. Current sources of carbon tetrachloride (ccl) in our atmosphere. *Environ. Res. Lett.* **13**, 024004 (2018).

[37] Barbera, A. C., Vymazal, J., Maucieri, C. *et al.* Greenhouse gases formation and emission. In *Encyclopedia of Ecology*, 329–333 (Elsevier, 2018).

[38] Liu, Z., Yang, X., Jia, W., Li, H. & Yang, X. Justification of CO as the working fluid for a compressed gas energy storage system: A thermodynamic and economic study. *J. Energy Storage* **27**, 101132 (2020).

[39] Ogden, J., Jaffe, A. M., Scheitrum, D., McDonald, Z. & Miller, M. Natural gas as a bridge to hydrogen transportation fuel: Insights from the literature. *Energy Policy* **115**, 317–329 (2018).

[40] Jia, C. et al. Three birds with one stone approach to superior n/s co-doped microporous carbon for gas storage and water purification. *Chem. Eng. J.* **431**, 133231 (2022).

[41] Conte, G. et al. Copper-doped activated carbon from amorphous cellulose for hydrogen, methane and carbon dioxide storage. *Int. J. Hydrog. Energy* **47**, 18384–18395 (2022).

[42] Ullah Rather, S. Preparation, characterization and hydrogen storage studies of carbon nanotubes and their composites: A review. *Int. J. Hydrog. Energy* **45**, 4653–4672 (2020).

[43] Gargiulo, V., Policicchio, A., Lisi, L. & Alfe, M. CO capture and gas storage capacities enhancement of hkust-1 by hybridization with functionalized graphene-like materials. *Energy Fuels* **37**, 5291–5302 (2023). 37058617 10.1021/acs.energyfuels.2c04289 PMC10084447

[44] Connolly, B. M., Madden, D. G., Wheatley, A. E. & Fairen-Jimenez, D. Shaping the future of fuel: Monolithic metal-organic frameworks for high-density gas storage. *J. Am. Chem. Soc.* **142**, 8541–8549 (2020). 32294384 10.1021/jacs.0c00270

[45] Kroto, H. W., Heath, J. R., O’Brien, S. C., Curl, R. F. & Smalley, R. E. C60: Buckminsterfullerene. *Nature* **318**, 162–163 (1985).

[46] Hashikawa, Y. & Murata, Y. Water in fullerenes. *Bull. Chem. Soc. Jpn.* **96**, 943–967 (2023).

[47] Shi, L. & Gan, L. Open-cage fullerenes as tailor-made container for a single water molecule. *J. Phys. Org. Chem.* **26**, 766–772 (2013).

[48] Bloodworth, S. et al. Synthesis and properties of open fullerenes encapsulating ammonia and methane. *ChemPhysChem* **19**, 266–276 (2018). 29131544 10.1002/cphc.201701212 PMC5838534

[49] Vougioukalakis, G. C., Roubelakis, M. M. & Orfanopoulos, M. Open-cage fullerenes: Towards the construction of nanosized molecular containers. *Chem. Soc. Rev.* **39**, 817–844 (2010). 20111794 10.1039/b913766a

[50] Hashikawa, Y., Sadai, S. & Murata, Y. Molecular CO storage: State of a single-molecule gas. *ACS Phys. Chem. Au* **4**, 143–147 (2024). 38560749 10.1021/acsphyschemau.3c00068 PMC10979473

[51] Hashikawa, Y., Fujikawa, N. & Murata, Y. -extended fullerenes with a reactant inside. *J. Am. Chem. Soc.* **144**, 23292–23296 (2022). 36534086 10.1021/jacs.2c12259

[52] Shi, L. et al. Punching a carbon atom of C60 into its own cavity to form an endohedral complex CO@ C59O6 under mild conditions. *Chem. A Eur. J.* **19**, 16545–16549 (2013). 10.1002/chem.201303501 24281804

[53] Bloodworth, S. et al. First synthesis and characterization of CH@ C. *Angew. Chem. Int. Ed.* **58**, 5038–5043 (2019). 10.1002/anie.201900983 PMC6492075 30773760

[54] Komatsu, K., Murata, M. & Murata, Y. Encapsulation of molecular hydrogen in fullerene C by organic synthesis. *Science* **307**, 238–240 (2005). 15653499 10.1126/science.1106185

[55] Rubin, Y. et al. Insertion of helium and molecular hydrogen through the orifice of an open fullerene. *Angew. Chem.* **113**, 1591–1594 (2001). 10.1002/1521-3773(20010417)40:8<1543::AID-ANIE1543>3.0.CO;2-6 29712354

[56] Iwamatsu, S.-I. et al. A bowl-shaped fullerene encapsulates a water into the cage. *J. Am. Chem. Soc.* **126**, 2668–2669 (2004). 14995161 10.1021/ja038537a

[57] Xiao, Z. et al. Synthesis of [59] fullerenones through peroxide-mediated stepwise cleavage of fullerene skeleton bonds and x-ray structures of their water-encapsulated open-cage complexes. *J. Am. Chem. Soc.* **129**, 16149–16162 (2007). 18052066 10.1021/ja0763798

[58] Kurotobi, K. & Murata, Y. A single molecule of water encapsulated in fullerene C. *Science* **333**, 613–616 (2011). 21798946 10.1126/science.1206376

[59] Hashikawa, Y., Kizaki, K., Hirose, T. & Murata, Y. An orifice design: Water insertion into C. *RSC Adv.* **10**, 40406–40410 (2020). 35520847 10.1039/d0ra09067k PMC9057476

[60] Linga, P., Kumar, R. & Englezos, P. Gas hydrate formation from hydrogen/carbon dioxide and nitrogen/carbon dioxide gas mixtures. *Chem. Eng. Sci.* **62**, 4268–4276 (2007).

[61] Xiao, P. et al. Enhanced formation of methane hydrate from active ice with high gas uptake. *Nat. Commun.* **14**, 8068 (2023). 38057299 10.1038/s41467-023-43487-6 PMC10700304

[62] Prasad, P. S. & Sai Kiran, B. Clathrate hydrates of greenhouse gases in the presence of natural amino acids: Storage, transportation and separation applications. *Sci. Rep.* **8**, 8560 (2018). 29867219 10.1038/s41598-018-26916-1 PMC5986743

[63] Farrando-Perez, J. et al. Rapid and efficient hydrogen clathrate hydrate formation in confined nanospace. *Nat. Commun.* **13**, 5953 (2022). 36216832 10.1038/s41467-022-33674-2 PMC9550858

[64] Zhao, W., Wang, L., Bai, J., Francisco, J. S. & Zeng, X. C. Spontaneous formation of one-dimensional hydrogen gas hydrate in carbon nanotubes. *J. Am. Chem. Soc.* **136**, 10661–10668 (2014). 24885238 10.1021/ja5041539

[65] Zhao, W., Francisco, J. S. & Zeng, X. C. Co separation from H via hydrate formation in single-walled carbon nanotubes. *J. Phys. Chem. Lett.* **7**, 4911–4915 (2016). 27934039 10.1021/acs.jpclett.6b02443

[66] Kang, K. C., Linga, P., Park, K.-N., Choi, S.-J. & Lee, J. D. Seawater desalination by gas hydrate process and removal characteristics of dissolved ions (Na^+^, K^+^, Mg^2+^, Ca^2+^, B^3+^, Cl^-^, SO^4^^2-^). *Desalination* **353**, 84–90 (2014).

[67] Chesnokov, C. et al. Analytical model for Joule-Thomson cooling under heat exchange during CO storage. *Adv. Water Resour.*10.1016/j.advwatres.2024.104758 (2024).

[68] Ramya, K. & Venkatnathan, A. Stability and reactivity of methane clathrate hydrates: Insights from density functional theory. *J. Phys. Chem. A* **116**, 7742–7745 (2012). 22738177 10.1021/jp304229p

[69] Kumar, P. & Sathyamurthy, N. Theoretical studies of host-guest interaction in gas hydrates. *J. Phys. Chem. A* **115**, 14276–14281 (2011). 22044163 10.1021/jp2089565

[70] Liu, Y., Zhao, J., Li, F. & Chen, Z. Appropriate description of intermolecular interactions in the methane hydrates: An assessment of DFT methods. *J. Comput. Chem.* **34**, 121–131 (2013). 22949382 10.1002/jcc.23112

[71] Román-Pérez, G., Moaied, M., Soler, J. M. & Yndurain, F. Stability, adsorption, and diffusion of CH, CO, and H in clathrate hydrates. *Phys. Rev. Lett.* **105**, 145901 (2010). 21230845 10.1103/PhysRevLett.105.145901

[72] Alavi, S., Udachin, K. & Ripmeester, J. A. Effect of guest-host hydrogen bonding on the structures and properties of clathrate hydrates. *Chem. A Eur. J.* **16**, 1017–1025 (2010). 10.1002/chem.200902351 19946907

[73] Devlin, J. P. & Monreal, I. A. Clathrate-hydrate ultrafast nucleation and crystallization from supercooled aqueous nanodroplets. *Chem. Phys. Lett.* **492**, 1–8 (2010).

[74] Joseph, J. & Jemmis, E. D. Red-, blue-, or no-shift in hydrogen bonds: A unified explanation. *J. Am. Chem. Soc.* **129**, 4620–4632 (2007). 17375920 10.1021/ja067545z

[75] Baburao, G., Esakkimuthu, A. & Ragupathy, G. Comparative analysis of red and blue-shifting hydrogen bonds in 1: 1 haloform complexes. *Comput. Theor. Chem.* **1242**, 114935 (2024).

[76] Loveday, J. & Nelmes, R. High-pressure gas hydrates. *Phys. Chem. Chem. Phys.* **10**, 937–950 (2008). 18259632 10.1039/b704740a

[77] Bader, R. F. Atoms in molecules. *Acc. Chem. Res.* **18**, 9–15 (1985).

[78] Frisch, M. J. et al. *Gaussian 16 Revision C.01* (Gaussian Inc., Wallingford, 2016).

[79] Lendening, E., Reed, A., Carpenter, J. & Weinhold, F. *NBO, version 3.1* (Gaussian, Pittsburgh Inc., 2003).

[80] Gopi, R., Ramanathan, N. & Sundararajan, K. Acetonitrile-water hydrogen-bonded interaction: Matrix-isolation infrared and ab initio computation. *J. Mol. Struct.* **1094**, 118–129 (2015).

[81] Lu, T. A comprehensive electron wavefunction analysis toolbox for chemists, multiwfn. *J. Chem. Phys.* **161**, 082503 (2024). 39189657 10.1063/5.0216272

[82] Humphrey, W., Dalke, A. & Schulten, K. VMD: Visual molecular dynamics. *J. Mol. Graph.* **14**, 33–38 (1996). 8744570 10.1016/0263-7855(96)00018-5

[83] Baerends, E. J. *et al.* Adf 2024.1,scm, theoretical chemistry, vrije universiteit, amsterdam, the netherlands. ADF. Available online: http://www scm.com.

