# Supplementary material for: Encapsulation of greenhouse gases in clathrate hydrates with insights into structure, energetics, chemical interactions, and environmental implications
Source: Sci Rep. 2025 Jul 5;15:24090. doi: 10.1038/s41598-025-08202-z (PMC12228833; doi:10.1038/s41598-025-08202-z)
Supplement: Supplementary file 1 — Supplementary Information 1. [file 41598_2025_8202_MOESM1_ESM.pdf]

Supporting Information

Encapsulation of Greenhouse Gases in Clathrate Hydrates with Insights into Structure, Energetics, Chemical Interactions, and Environmental Implications

Arun Ramamurthy, Giridhar Baburao, Abburi Jahnavi and Gopi Ragupathy*

Department of Chemistry, School of Advanced Sciences, Vellore Institute of Technology, Vellore-632014, India

*E-mail: r.gopi@vit.ac.in

Content

**Tables**	**Page No.**
**Table S1.** Bonding and anti-bonding occupancies and their *s*- and *p*-orbital contribution of their Greenhouse gases.	**10**
**Table S2.** Bonding and anti-bonding occupancies and their s- and p-orbital contribution of their Greenhouse gases after encapsulated in 5^{12} hydrate clathrates.	**12**
**Table S3.** Bonding and anti-bonding occupancies and their *s*- and *p*-orbital contribution of their Greenhouse gases after encapsulated in $5^{12}6^{2}$ hydrate clathrates.	**14**
**Table S4.** Bonding and anti-bonding occupancies and their *s*- and *p*-orbital contribution of their Greenhouse gases after encapsulated in $5^{12}6^{4}$ hydrate clathrates.	**16**
**Table S5.** Topological parameters of Greenhouse gases from QTAIM analysis.	**18**
**Table S6.** Topological parameters of Greenhouse gases within 5^{12} hydrate cages from QTAIM analysis.	**19**
**Table S7.** Topological parameters of Greenhouse gases within $5^{12}6^{2}$ hydrate cages from QTAIM analysis.	**20**
**Table S8.** Topological parameters of Greenhouse gases within $5^{12}6^{4}$ hydrate cages from QTAIM analysis.	**21**

Table S9. Electron density ($\rho(r)$) and Laplacian electron density ($\nabla^2\rho(r)$) of the bond critical point between guest and host molecules from the QTAIM analysis. **22**

Table S10. Bond angles of the guest Greenhouse gases encapsulated in 5^{12}, $5^{12}6^2$ and $5^{12}6^4$ hydrate clathrates. All the angles in degree (°). **23**

Table S11. Distortion energies of the 5^{12}, $5^{12}6^2$ and $5^{12}6^4$ hydrate clathrate cages for after the Greenhouse gases encapsulation computes at B3LYP-D3BJ/6-31G(d) level of theory and basis set. All the values in kcal/mol. **25**

Table S12. Vibrational symmetric stretching frequencies and their IR intensity of the Greenhouse gases guest molecule encapsulated in 5^{12}, $5^{12}6^2$ and $5^{12}6^4$ hydrate clathrate cages. **26**

Table S13. Total energies of the Greenhouse gases encapsulation in 5^{12}, $5^{12}6^2$ and $5^{12}6^4$ hydrate clathrate cages computes at B3LYP/6-31G(d) and B3LYP-D3BJ/6-31G(d) level of theory and basis sets. All the values in Hartree. **27**

Table S14. Global Chemical indices of Greenhouse gases within 5^{12} hydrate clathrates. All values in eV. **28**

Table S15. Global Chemical indices of Greenhouse gases within $5^{12}6^2$ hydrate clathrates. All values in eV. **29**

Table S16. Global Chemical indices of Greenhouse gases within $5^{12}6^4$ hydrate clathrates. All values in eV. **30**

Table S17. Cartesian coordinates for optimized geometries of the guest molecule monomers computes at B3LYP/6-31G(d) level of theory and basis set. **99**

Table S18. Cartesian coordinates for optimized geometries of the empty 5^{12}, $5^{12}6^{2}$ and $5^{12}6^{4}$ clathrate hydrate monomers computes at B3LYP/6-31G(d) level of theory and basis set. **103**

Table S19. Cartesian coordinates for optimized geometries of the Greenhouse gases encapsulated within 5^{12} clathrate hydrate computes at B3LYP/6-31G(d) level of theory and basis set. **111**

Table S20. Cartesian coordinates for optimized geometries of the Greenhouse gases encapsulated within $5^{12}6^{2}$ clathrate hydrate computes at B3LYP/6-31G(d) level of theory and basis set. **146**

Table S21. Cartesian coordinates for optimized geometries of the Greenhouse gases encapsulated within $5^{12}6^{4}$ clathrate hydrate computes at B3LYP/6-31G(d) level of theory and basis set. **186**

Figures	Page No.
**Figure S1.** Mulliken charges of the Greenhouse gases guest molecule monomers before encapsulation in clathrate hydrate computes at B3LYP/6-31G(d) level of theory and basis set.	**31**
**Figure S2.** Mulliken charges of the empty 5^{12}, $5^{12}6^2$ and $5^{12}6^4$ clathrate hydrate monomers computes at B3LYP/6-31G(d) level of theory and basis set.	**33**
**Figure S3.** Mulliken charges of the CCl_4 molecule within 5^{12}, $5^{12}6^2$ and $5^{12}6^4$ clathrate hydrates computes at B3LYP/6-31G(d) level of theory and basis set.	**34**
**Figure S4.** Mulliken charges of the CF_2Cl_2 molecule within 5^{12}, $5^{12}6^2$ and $5^{12}6^4$ clathrate hydrates computes at B3LYP/6-31G(d) level of theory and basis set.	**35**
**Figure S5.** Mulliken charges of the CH_3Br molecule within 5^{12}, $5^{12}6^2$ and $5^{12}6^4$ clathrate hydrates computes at B3LYP/6-31G(d) level of theory and basis set.	**36**
**Figure S6.** Mulliken charges of the CH_3Cl molecule within 5^{12}, $5^{12}6^2$ and $5^{12}6^4$ clathrate hydrates computes at B3LYP/6-31G(d) level of theory and basis set.	**37**
**Figure S7.** Mulliken charges of the CH_4 molecule within 5^{12}, $5^{12}6^2$ and $5^{12}6^4$ clathrate hydrates computes at B3LYP/6-31G(d) level of theory and basis set.	**38**
**Figure S8.** Mulliken charges of the CO_2 molecule within 5^{12}, $5^{12}6^2$ and $5^{12}6^4$ clathrate hydrates computes at B3LYP/6-31G(d) level of theory and basis set.	**39**

Figure S9. Mulliken charges of the CO molecule within 5^{12}, $5^{12}6^2$ and $5^{12}6^4$ clathrate hydrates computes at B3LYP/6-31G(d) level of theory and basis set. **40**

Figure S10. Mulliken charges of the H_2S molecule within 5^{12}, $5^{12}6^2$ and $5^{12}6^4$ clathrate hydrates computes at B3LYP/6-31G(d) level of theory and basis set. **41**

Figure S11. Mulliken charges of the CH_3F molecule within 5^{12}, $5^{12}6^2$ and $5^{12}6^4$ clathrate hydrates computes at B3LYP/6-31G(d) level of theory and basis set. **42**

Figure S12. Mulliken charges of the N_2O molecule within 5^{12}, $5^{12}6^2$ and $5^{12}6^4$ clathrate hydrates computes at B3LYP/6-31G(d) level of theory and basis set. **43**

Figure S13. Mulliken charges of the NF_3 molecule within 5^{12}, $5^{12}6^2$ and $5^{12}6^4$ clathrate hydrates computes at B3LYP/6-31G(d) level of theory and basis set. **44**

Figure S14. Mulliken charges of the O_3 molecule within 5^{12}, $5^{12}6^2$ and $5^{12}6^4$ clathrate hydrates computes at B3LYP/6-31G(d) level of theory and basis set. **45**

Figure S15. Mulliken charges of the CF_4 molecule within 5^{12}, $5^{12}6^2$ and $5^{12}6^4$ clathrate hydrates computes at B3LYP/6-31G(d) level of theory and basis set. **46**

Figure S16. Mulliken charges of the SF_6 molecule within 5^{12}, $5^{12}6^2$ and $5^{12}6^4$ clathrate hydrates computes at B3LYP/6-31G(d) level of theory and basis set. **47**

Figure S17. Mulliken charges of the SO_2 molecule within 5^{12}, $5^{12}6^2$ and $5^{12}6^4$ clathrate hydrates computes at B3LYP/6-31G(d) level of theory and basis set. **48**

Figure S18. Frontier molecular orbital of the empty 5^{12}, $5^{12}6^2$ and $5^{12}6^4$ clathrate hydrates computes at B3LYP/6-31G(d) level of theory and basis set. **49**

Figure S19. Frontier molecular orbital of the CCl_4 molecule before and after encapsulation in 5^{12}, $5^{12}6^2$ and $5^{12}6^4$ clathrate hydrates computes at B3LYP/6-31G(d) level of theory and basis set. **51**

Figure S20. Frontier molecular orbital of the CF_2Cl_2 molecule before and after encapsulation in 5^{12}, $5^{12}6^2$ and $5^{12}6^4$ clathrate hydrates computes at B3LYP/6-31G(d) level of theory and basis set. **53**

Figure S21. Frontier molecular orbital of the CH_3Br molecule before and after encapsulation in 5^{12}, $5^{12}6^2$ and $5^{12}6^4$ clathrate hydrates computes at B3LYP/6-31G(d) level of theory and basis set. **55**

Figure S22. Frontier molecular orbital of the CH_3Cl molecule before and after encapsulation in 5^{12}, $5^{12}6^2$ and $5^{12}6^4$ clathrate hydrates computes at B3LYP/6-31G(d) level of theory and basis set. **57**

Figure S23. Frontier molecular orbital of the CH_4 molecule before and after encapsulation in 5^{12}, $5^{12}6^2$ and $5^{12}6^4$ clathrate hydrates computes at B3LYP/6-31G(d) level of theory and basis set. **59**

Figure S24. Frontier molecular orbital of the CO_2 molecule before and after encapsulation in 5^{12}, $5^{12}6^2$ and $5^{12}6^4$ clathrate hydrates computes at B3LYP/6-31G(d) level of theory and basis set. **61**

Figure S25. Frontier molecular orbital of the CO molecule before and after encapsulation in 5^{12}, $5^{12}6^2$ and $5^{12}6^4$ clathrate hydrates computes at B3LYP/6-31G(d) level of theory and basis set. **63**

Figure S26. Frontier molecular orbital of the H_2S molecule before and after encapsulation in 5^{12}, $5^{12}6^2$ and $5^{12}6^4$ clathrate hydrates computes at B3LYP/6-31G(d) level of theory and basis set. **65**

Figure S27. Frontier molecular orbital of the CH_3F molecule before and after encapsulation in 5^{12}, $5^{12}6^2$ and $5^{12}6^4$ clathrate hydrates computes at B3LYP/6-31G(d) level of theory and basis set. **67**

Figure S28. Frontier molecular orbital of the N_2O molecule before and after encapsulation in 5^{12}, $5^{12}6^2$ and $5^{12}6^4$ clathrate hydrates computes at B3LYP/6-31G(d) level of theory and basis set. **69**

Figure S29. Frontier molecular orbital of the NF_3 molecule before and after encapsulation in 5^{12}, $5^{12}6^2$ and $5^{12}6^4$ clathrate hydrates computes at B3LYP/6-31G(d) level of theory and basis set. **71**

Figure S30. Frontier molecular orbital of the O_3 molecule before and after encapsulation in 5^{12}, $5^{12}6^2$ and $5^{12}6^4$ clathrate hydrates computes at B3LYP/6-31G(d) level of theory and basis set. **73**

Figure S31. Frontier molecular orbital of the CF_4 molecule before and after encapsulation in 5^{12}, $5^{12}6^2$ and $5^{12}6^4$ clathrate hydrates computes at B3LYP/6-31G(d) level of theory and basis set. **75**

Figure S32. Frontier molecular orbital of the SF_6 molecule before and after encapsulation in 5^{12}, $5^{12}6^2$ and $5^{12}6^4$ clathrate hydrates computes at B3LYP/6-31G(d) level of theory and basis set. **77**

Figure S33. Frontier molecular orbital of the SO_2 molecule before and after encapsulation in 5^{12}, $5^{12}6^2$ and $5^{12}6^4$ clathrate hydrates computes at B3LYP/6-31G(d) level of theory and basis set. **79**

Figure S34. RDG plot and their iso-surface from Non-covalent Interaction (NCI) analysis. **81**

Figure S35. Electron Localized function (ELF) Mapping of Greenhouse gases encased within 5^{12} hydrate clathrates. **88**

Figure S36. Electron Localized Function (ELF) Mapping of Greenhouse gases encased within $5^{12}6^2$ hydrate clathrates. **89**

Figure S37. Electron Localized Function (ELF) Mapping of Greenhouse gases encased within $5^{12}6^4$ hydrate clathrates. 90

Figure S38. Bar plot for the energy gap among the Greenhouse gases encapsulation in 5^{12}, $5^{12}6^2$ and $5^{12}6^4$ clathrate hydrates. 91

Figure S39. Bar plot for the chemical potential among the Greenhouse gases encapsulation in 5^{12}, $5^{12}6^2$ and $5^{12}6^4$ clathrate hydrates. 92

Figure S40. Bar plot for the chemical hardness among the Greenhouse gases encapsulation in 5^{12}, $5^{12}6^2$ and $5^{12}6^4$ clathrate hydrates. 93

Figure S41. Bar plot for the electronegativity among the Greenhouse gases encapsulation in 5^{12}, $5^{12}6^2$ and $5^{12}6^4$ clathrate hydrates. 94

Figure S42. Bar plot for the electrophilicity index among the Greenhouse gases encapsulation in 5^{12}, $5^{12}6^2$ and $5^{12}6^4$ clathrate hydrates. 95

Figure S43. Plot for the relation between the $\Delta\nu$ (in cm^{-1}) and Δr (in Å) for GHGs such as CH_3Br, CH_3Cl, CF_2Cl_2 and CH_3F within the three hydrate clathrate cages of their bonds C-Br, C-Cl, C-F, and C-F respectively. 96

Figure S44. Plot for the relation between the $\Delta\nu$ (in cm^{-1}) and $\nabla^2 \rho(r)$ (in a.u.) for GHGs such as CH_3Br, CH_3Cl, CF_2Cl_2 and CH_3F within the three hydrate clathrate cages of their bonds C-Br, C-Cl, C-F, and C-F respectively. 97

Figure S45. Plot for the relation between the Δr (in Å) and $\nabla^2 \rho(r)$ (in a.u.) for GHGs such as CH_3Br, CH_3Cl, CF_2Cl_2 and CH_3F within the three hydrate clathrate cages of their bonds C-Br, C-Cl, C-F, and C-F respectively. 98

Table S1. Bonding and anti-bonding occupancies and their *s*- and *p*-orbital contribution of their Greenhouse gases

Guest Molecule	Bond	Occupancy		Contribution in %	
		σ (Bonding)	σ* (Anti Bonding)	*s*-orbital	*p*-orbital
CCl_4	C-Cl	1.98925	0.10883	25.00	74.82
				14.43	85.02
CF_2Cl_2	C-Cl	1.98678	0.12333	25.53	74.24
				14.41	85.07
	C-F	1.99549	0.12319	24.53	75.05
				29.78	70.13
CH_3Br	C-H	1.99824	0.00881	27.73	72.21
				100.0	-
	C-Br	1.99824	0.00177	16.81	83.08
				11.38	87.90
CH_3Cl	C-H	1.99757	0.01165	27.10	72.83
				100.0	-
	C-Cl	1.99820	0.00167	18.69	81.13
				15.22	84.33
CH_4	C-H	1.99926	0.00046	25.00	74.94
				100.0	-
CO_2	C-O	1.99891	0.37901	-	99.74
				-	99.62
CO	C-O	2.0000	0	-	99.57
				-	99.58
H_2S	S-H	1.99888	0.00094	15.47	83.78
				100.0	-
CH_3F	C-H	1.99859	0.01852	26.70	72.83
				100.0	-
	C-F	1.99927	0.00259	19.95	79.87
				26.16	73.68
	C-F	1.99825	0.00425	19.03	80.8
				25.81	74.05
N_2O	O-N	1.98936	0.01572	22.23	77.27
				14.20	85.58
NF_3	N-F	1.99187	0.09659	12.18	87.46
				14.50	85.34

O_3	O-O	1.98839	0.00897	6.680	92.98
				6.680	92.98
CF_4	C-F	1.99380	0.12620	25.00	74.65
				27.63	72.19
SF_6	S-F	1.88535	0.17710	16.67	50.00
				15.94	83.89
SO_2	S-O	1.99455	0.10899	16.18	81.93
				24.37	74.94

Table S2. Bonding and anti-bonding occupancies and their *s*- and *p*-orbital contribution of their Greenhouse gases after encapsulated in 5^{12} hydrate clathrates.

Guest Molecule	Bond	Occupancy		Contribution in %	
		σ (Bonding)	σ* (Anti Bonding)	*s*-orbital	*p*-orbital
CCl_4	C-Cl	1.9887	0.11261	24.73	75.08
				14.64	84.84
CF_2Cl_2	C-Cl	1.98679	0.12932	26.24	73.54
				14.57	84.89
	C-F	1.99490	0.13355	23.67	75.91
				29.51	70.41
CH_3Br	C-H	1.99648	0.01876	28.46	71.48
				100.0	-
	C-Br	1.99652	0.01706	14.59	85.28
				12.28	87.45
CH_3Cl	C-H	1.99625	0.01995	27.49	72.45
				100.0	-
	C-Cl	1.99713	0.01148	17.04	82.77
				16.12	83.49
CH_4	C-H	1.99796	0.00867	25.37	74.57
				100.0	-
CO_2	C-O	1.99815	0.36670	49.88	50.09
				37.32	62.25
CO	C-O	1.99806	0.00751	5.050	94.55
				9.750	89.76
H_2S	S-H	1.99763	0.05235	18.63	80.76
				100.0	-
	C-F	1.99927	0.00259	19.95	79.87
				26.16	73.68
CH_3F	C-H	1.99757	0.01983	27.26	72.66
				100.0	-
	C-F	1.99783	0.00685	17.60	82.24
				25.30	74.57
N_2O	O-N	1.98098	0.05114	19.40	80.10
				13.71	86.07

NF_3	N-F	1.99079	0.10352	11.39	88.25
				14.26	85.58
O_3	O-O	1.99493	0.06199	13.82	85.93
				22.03	77.81
CF_4	C-F	1.99343	0.12596	24.85	74.63
				27.64	72.27
SF_6	S-F	1.88446	0.18078	16.52	49.98
				16.24	83.60
SO_2	S-O	1.99251	0.11352	16.08	82.12
				26.00	73.42

Table S3. Bonding and anti-bonding occupancies and their *s*- and *p*-orbital contribution of their Greenhouse gases after encapsulated in $5^{12}6^2$ hydrate clathrates.

Guest Molecule	Bond	Occupancy		Contribution in %	
		σ (Bonding)	σ* (Anti Bonding)	*s*-orbital	*p*-orbital
CCl_4	C-Cl	1.98902	0.12283	24.65	75.17
				14.76	84.72
CF_2Cl_2	C-Cl	1.98642	0.12391	25.44	74.33
				14.82	84.67
	C-F	1.99486	0.13183	23.79	75.80
				29.41	70.50
CH_3Br	C-H	1.99727	0.01487	28.26	71.67
				100.0	-
	C-Br	1.99742	0.00835	15.30	84.58
				11.97	87.77
CH_3Cl	C-H	1.99658	0.01803	27.40	72.54
				100.0	-
	C-Cl	1.99776	0.00605	17.20	82.61
				15.74	83.86
CH_4	C-H	1.99818	0.00744	25.30	74.65
				100.0	-
CO_2	C-O	1.99795	0.02065	49.91	50.06
				37.10	62.47
CO	C-O	1.9979	0.00566	7.380	92.24
				13.69	85.80
H_2S	S-H	1.99795	0.04156	17.92	81.45
				100.0	-
CH_3F	C-H	1.99763	0.02193	27.27	72.65
				100.0	-
	C-F	1.99798	0.00469	18.59	81.24
				25.85	74.01
N_2O	O-N	1.98682	0.02711	20.41	79.09
				14.58	85.2
NF_3	N-F	1.99087	0.09799	11.90	87.74
				14.32	85.52
O_3	O-O	1.98785	0.01543	6.640	93.03
				6.410	93.25

CF_4	C-F	1.99355	0.12449	25.06	74.42
				27.55	72.36
SF_6	S-F	1.88369	0.18043	16.64	50.00
				15.95	83.88
SO_2	S-O	1.99235	0.11747	15.63	82.45
				25.10	74.32

Table S4. Bonding and anti-bonding occupancies and their *s*- and *p*-orbital contribution of their Greenhouse gases after encapsulated in $5^{12}6^4$ hydrate clathrates.

Guest Molecule	Bond	Occupancy		Contribution in %	
		**σ (Bonding)**	**σ* (Anti Bonding)**	***s*-orbital**	***p*-orbital**
CCl_4	C-Cl	1.989	0.11954	25.15	74.67
				14.37	85.09
CF_2Cl_2	C-Cl	1.98639	0.12583	25.97	73.80
				14.26	85.21
	C-F	1.99512	0.12469	24.39	75.19
				29.60	70.31
CH_3Br	C-H	1.9974	0.01328	27.83	72.10
				100.0	-
	C-Br	1.99766	0.01307	16.24	83.64
				11.46	88.25
CH_3Cl	C-H	1.99714	0.01440	26.93	73.00
				100.0	-
	C-Cl	1.99795	0.00359	17.98	81.84
				15.43	84.15
CH_4	C-H	1.99853	0.00618	25.35	74.60
				100.0	-
CO_2	C-O	1.99765	0.37329	0.750	98.99
				0.250	99.38
CO	C-O	1.99935	0.0047	0.010	99.57
				0.010	99.57
H_2S	S-H	1.9986	0.00997	15.95	83.33
				100.0	-
CH_3F	C-H	1.9982	0.01695	26.63	73.29
				100.0	-
	C-F	1.99825	0.00425	19.03	80.80
				25.81	74.05
N_2O	O-N	1.98182	0.04882	18.92	80.57
				14.04	85.74
NF_3	N-F	1.99107	0.09589	12.18	87.46
				14.40	85.43

O_3	O-O	1.98796	0.01031	6.680	92.98
				6.720	92.94
CF_4	C-F	1.99365	0.12382	25.11	74.52
				27.63	72.19
SF_6	S-F	1.88373	0.17904	16.77	50.00
				15.70	84.12
SO_2	S-O	1.9936	0.1126	16.10	82.04
				25.36	74.03

Table S5. Topological parameters of Greenhouse gases from QTAIM analysis.

Molecule	***Bond***	$\rho(r)$	***G(r)***	***V(r)***	$-G(r)/V(r)$	***H(r)***	$\nabla^2\rho(r)$	***ELF***	***LOL***	ε
CCl_4	C-Cl	0.1885	0.06301	-0.1823	0.34564	-0.1193	-0.2253	0.8886	0.7385	0
CF_2Cl_2	C-Cl	0.1944	0.06239	-0.1893	0.32958	-0.127	-0.2584	0.9002	0.7502	0.02153
	C-F	0.2856	0.3637	-0.7928	0.45875	-0.429	-0.2611	0.4886	0.4943	0.07251
CH_3Br	C-H	0.2786	0.03418	-0.314	0.10885	-0.2799	-0.9829	0.99	0.9089	0.01932
	C-Br	0.1427	0.04489	-0.1257	0.35712	-0.08082	-0.1437	0.8614	0.7138	0
CH_3Cl	C-H	0.2784	0.03363	-0.3123	0.10768	-0.2786	-0.9802	0.9903	0.9101	0.02658
	C-Cl	0.1741	0.05655	-0.1652	0.34231	-0.1086	-0.2085	0.8836	0.7337	0
CH_4	C-H	0.2686	0.03928	-0.2999	0.13098	-0.2606	-0.8854	0.9852	0.8909	0
CO_2	C-O	0.4421	0.8823	-1.641	0.53766	-0.7593	0.4916	0.4107	0.455	0
CO	C-O	0.4712	0.1108	-0.1878	0.58999	-0.7701	1.354	0.3531	0.4249	0
H_2S	S-H	0.2028	0.04193	-0.204	0.20554	-0.1621	-0.4807	0.9582	0.8274	0.09614
CH_3F	C-H	0.2784	0.03242	-0.3085	0.10509	-0.276	-0.9746	0.991	0.9131	0.04937
	C-F	0.2376	0.3263	-0.6528	0.49985	-0.3265	-0.7241	0.3914	0.445	0
N_2O	N-O	0.397	0.3421	-0.8705	0.39299	-0.5284	-0.745	0.7641	0.6428	0.32364
NF_3	N-F	0.3158	0.2265	-0.5036	0.44976	-0.277	-0.2018	0.775	0.6498	0.07923
O_3	O-O	0.2842	0.2415	-0.48	0.50313	-0.2385	0.01199	0.6807	0.5935	0.0403
CF_4	C-F	0.2954	0.352	-0.8082	0.43554	-0.4561	-0.416	0.5331	0.5165	0
SF_6	S-F	0.2074	0.245	-0.4804	0.50999	-0.2353	0.03895	0.4204	0.4599	0
SO_2	S-O	0.2746	0.6105	-0.9068	0.67325	-0.2962	1.256	0.2294	0.353	0.15083

Table S6. Topological parameters of Greenhouse gases within 5^{12} hydrate cages from QTAIM analysis.

Molecule	***Bond***	$\rho(r)$	***G(r)***	***V(r)***	$-G(r)/V(r)$	***H(r)***	$\nabla^2\rho(r)$	***ELF***	***LOL***	ε
CCl_4	C-Cl	0.1897	0.06381	-0.1848	0.34529	-0.121	-0.2289	0.8882	0.7381	0.00089
CF_2Cl_2	C-Cl	0.2056	0.06736	-0.2086	0.32291	-0.1413	-0.2958	0.9030	0.7532	0.02034
	C-F	0.2872	0.371	-0.8038	0.46156	-0.4327	-0.2601	0.4835	0.4917	0.09038
CH_3Br	C-H	0.2841	0.03316	-0.3251	0.1020	-0.2920	-1.0350	0.9912	0.9140	0.02307
	C-Br	0.1398	0.04415	-0.1223	0.3610	-0.07816	-0.1360	0.8570	0.7101	0.00446
CH_3Cl	C-H	0.2824	0.03231	-0.3201	0.10094	-0.2878	-1.0220	0.9914	0.9152	0.02815
	C-Cl	0.1703	0.05768	-0.1645	0.35064	-0.1068	-0.1966	0.8715	0.7226	0.00164
CH_4	C-H	0.2701	0.03831	-0.3020	0.12685	-0.2637	-0.9015	0.9862	0.8943	0.00118
CO_2	C-O	0.4401	0.8692	-1.6250	0.53489	-0.7558	0.4537	0.4143	0.4568	0.00385
CO	C-O	0.4704	1.0980	-1.8690	0.58748	-0.7711	1.3080	0.3561	0.4265	0.00082
H_2S	S-H	0.1968	0.0344	-0.1811	0.18995	-0.1467	-0.4492	0.9686	0.8475	0.06595
CH_3F	C-H	0.2836	0.0316	-0.3186	0.09909	-0.2870	-1.0220	0.9919	0.9175	0.05238
	C-F	0.2132	0.2931	-0.5723	0.51214	-0.2791	0.05616	0.3572	0.4271	0.01329
N_2O	N-O	0.4098	0.3522	-0.9118	-0.3863	-0.5596	-0.8298	0.7727	0.6483	0.29696
NF_3	N-F	0.3184	0.2283	-0.5107	0.44703	-0.2824	-0.2162	0.7770	0.6512	0.07749
O_3	O-O	0.2796	0.2393	-0.47063	-0.5087	-0.2312	0.03267	0.6728	0.5891	0.03845
CF_4	C-F	0.2970	0.3581	-0.8179	0.43783	-0.4598	-0.4068	0.5291	0.5145	0.00177
SF_6	S-F	0.2065	0.2421	-0.4760	0.50861	-0.2338	0.03318	0.4226	0.4610	0.00207
SO_2	S-O	0.2705	0.5785	-0.8732	0.66251	-0.2947	1.1350	0.2398	0.3597	0.13289

Table S7. Topological parameters of Greenhouse gases within $5^{12}6^2$ hydrate cages from QTAIM analysis.

Molecule	*Bond*	$\rho(r)$	*G(r)*	*V(r)*	$-G(r)/V(r)$	*H(r)*	$\nabla^2\rho(r)$	*ELF*	*LOL*	ε
CCl_4	C-Cl	0.1967	0.06697	-0.1969	0.34012	-0.1299	-0.2520	0.8906	0.7405	0.00414
CF_2Cl_2	C-Cl	0.1989	0.06433	-0.1971	0.32638	-0.1328	-0.2740	0.9015	0.7516	0.02141
	C-F	0.281	0.354	-0.7735	0.45766	-0.4195	-0.2650	0.4888	0.4944	0.08961
CH_3Br	C-H	0.2809	0.03375	-0.3184	0.1060	-0.2847	-1.003	0.9905	0.9111	0.02343
	C-Br	0.1378	0.04269	-0.1183	0.36086	-0.07563	-0.1332	0.8594	0.712	0.00076
CH_3Cl	C-H	0.28	0.03231	-0.3151	0.10254	-0.2828	-1.002	0.9912	0.9141	0.02947
	C-Cl	0.1673	0.05595	-0.1587	0.35255	-0.1028	-0.1873	0.8716	0.7227	0.00073
CH_4	C-H	0.2694	0.03862	-0.3008	0.12839	-0.2621	-0.8942	0.9858	0.8931	0.00241
CO_2	C-O	0.4452	0.8969	-1.663	0.53933	-0.7662	0.5227	0.4085	0.4538	0.00359
CO	C-O	0.4694	1.096	-1.864	0.58798	-0.768	1.313	0.3554	0.4261	0.00111
H_2S	S-H	0.1978	0.03524	-0.1839	0.19163	-0.1487	-0.4538	0.9676	0.8454	0.06567
CH_3F	C-H	0.28	0.03174	-0.3115	0.10189	-0.2797	-0.9922	0.9915	0.9155	0.05256
	C-F	0.2218	0.3032	-0.5989	0.50626	-0.2957	0.02979	0.3721	0.435	0.01023
N_2O	N-O	0.3966	0.3388	-0.8634	-0.3924	-0.5245	-0.7428	0.7669	0.6446	0.31623
NF_3	N-F	0.3127	0.2248	-0.4961	0.45313	-0.2712	-0.1854	0.7719	0.6479	0.07885
O_3	O-O	0.4299	0.4033	-0.8732	-0.4619	-0.4698	0.2533	0.7523	0.6354	0.06811
CF_4	C-F	0.298	0.3572	-0.8194	0.43593	-0.4621	-0.4198	0.5332	0.5166	0.00264
SF_6	S-F	0.2064	0.2372	-0.4714	0.50318	-0.2342	0.0118	0.4323	0.466	0.00077
SO_2	S-O	0.2738	0.5991	-0.8968	0.66804	-0.2977	1.205	0.2344	0.3562	0.14501

Table S8. Topological parameters of Greenhouse gases within $5^{12}6^4$ hydrate cages from QTAIM analysis.

Molecule	*Bond*	*ρ(r)*	*G(r)*	*V(r)*	$-G(r)/V(r)$	*H(r)*	$\nabla^2\rho(r)$	*ELF*	*LOL*	*ε*
CCl_4	C-Cl	0.1905	0.06393	-0.186	0.34371	-0.1221	-0.2328	0.8893	0.7392	0.00211
CF_2Cl_2	C-Cl	0.1942	0.06234	-0.189	0.32984	-0.1266	-0.2573	0.8999	0.75	0.02148
	C-F	0.2846	0.3589	-0.7861	0.45656	-0.4272	-0.2691	0.4925	0.4962	0.07735
CH_3Br	C-H	0.2799	0.03357	-0.3168	0.10597	-0.2832	-0.9986	0.9905	0.911	0.02186
	C-Br	0.1395	0.04325	-0.1206	0.35862	-0.07734	-0.1342	0.8612	0.7136	0.00107
CH_3Cl	C-H	0.2785	0.03316	-0.3123	0.10618	-0.2791	-0.984	0.9906	0.9113	0.02778
	C-Cl	0.1706	0.05607	-0.1616	0.34697	-0.1055	-0.1978	0.8784	0.7289	0.00343
CH_4	C-H	0.2687	0.03928	-0.3	0.13093	-0.2607	-0.8857	0.9852	0.891	0.00093
CO_2	C-O	0.4431	0.8866	-0.1648	5.37985	-0.762	0.4984	0.4103	0.4547	0.00081
CO	C-O	0.4711	1.104	-1.876	0.58849	-0.772	1.328	0.355	0.4259	0.00034
H_2S	S-H	0.1974	0.0337	-0.1813	0.18588	-0.1476	-0.4557	0.9701	0.8508	0.07076
CH_3F	C-H	0.2795	0.03246	-0.3105	0.10454	-0.2781	-0.9826	0.9911	0.9135	0.05124
	C-F	0.2282	0.3111	-0.6195	0.50218	-0.3083	0.01119	0.3823	0.4403	0.01283
N_2O	N-O	0.3971	0.3411	-0.8684	-0.3928	-0.5273	-0.7446	0.7653	0.6436	0.32207
NF_3	N-F	0.3142	0.2259	-0.4998	0.45198	-0.2739	-0.1922	0.7732	0.6486	0.07898
O_3	O-O	0.3896	0.3582	-0.7587	-0.4721	-0.4005	0.242	0.7351	0.6249	0.05935
CF_4	C-F	0.2967	0.354	-0.8131	0.43537	-0.4591	-0.4201	0.5339	0.517	0.00069
SF_6	S-F	0.2052	0.2345	-0.4667	0.50246	-0.2321	0.00971	0.4331	0.4664	0.00027
SO_2	S-O	0.2719	0.5909	-0.8857	0.66716	-0.2947	1.184	0.2352	0.3567	0.14091

Table S9. Electron density ($\rho(r)$) and Laplacian electron density ($\nabla^2\rho(r)$) of the bond critical point between guest and host molecules from the QTAIM analysis.

**Greenhouse gases**	**Bond**	**5^{12}**		**$5^{12}6^2$**		**$5^{12}6^4$**	
		$\rho(r)$	$\nabla^2\rho(r)$	$\rho(r)$	$\nabla^2\rho(r)$	$\rho(r)$	$\nabla^2\rho(r)$
CCl_4	H-O···Cl	0.0123	0.0437	0.0162	0.0582	0.006	0.0238
CF_2Cl_2	H-O···F	0.0126	0.0546	0.0081	0.038	0.006	0.0302
CH_3Br	H-O···H	0.0118	0.0363	0.0091	0.028	0.0095	0.0291
CH_3Cl	H-O···H	0.0095	0.0303	0.0086	0.0272	0.0072	0.0229
CH_4	H-O···H	0.0056	0.019	0.0067	0.0225	0.0043	0.0155
CO	H-O···O	0.0047	0.0174	0.0024	0.0134	0.0013	0.0083
CO_2	H-O···O	0.0138	0.0488	0.0053	0.0232	0.0042	0.0199
H_2S	H-O···H	0.0322	0.0778	0.0288	0.0719	0.0302	0.0766
CH_3F	H-O···H	0.0097	0.0304	0.0086	0.0274	0.0078	0.0242
N_2O	H-O···O	0.0055	0.0205	0.0036	0.017	0.0038	0.0178
NF_3	H-O···F	0.0078	0.0366	0.005	0.0271	0.0067	0.0318
O_3	H-O···O	0.0019	0.0115	0.0033	0.0165	0.0052	0.0225
CF_4	H-O···F	0.0091	0.0429	0.0054	0.0284	0.0058	0.0304
SF_6	H-O···F	0.0122	0.0483	0.0065	0.0309	0.0045	0.024
SO_2	H-O···S	0.0184	0.052	0.0287	0.0761	0.0276	0.0751

Table S10. Bond angles of the guest Greenhouse gases encapsulated in 5^{12}, $5^{12}6^2$ and $5^{12}6^4$ hydrate clathrates. All the angles in degree (°).

Greenhouse gases	Bond angle	5^{12}	$5^{12}6^2$	$5^{12}6^4$
CCl_4	∠Cl-C-Cl	110.1	108.8	108.9
CF_2Cl_2	∠Cl-C-Cl	111.4	111.5	111.2
	∠Cl-C-F	109.3	108.9	109.6
	∠F-C-F	108.0	107.7	108.4
CH_3Br	∠H-C-Br	105.8	106.6	108.2
	∠H-C-H	113.7	112.5	110.8
CH_3Cl	∠H-C-Cl	106.9	107.7	108.2
	∠H-C-H	104.7	110.6	111.0
CH_4	∠H-C-H	109.3	109.4	109.2
CO	-	-	-	-
CO_2	∠O-C-O	176.1	176.5	178.7
H_2S	∠H-S-H	95.6	93.9	93.6
CH_3F	∠H-C-F	107.7	108.7	109.2
	∠H-C-H	111.1	110.1	109.9
N_2O	∠N-O-N	86.3	86.7	86.4

NF_3	∠F-N-F	101.2	101.2	101.8
O_3	∠O-O-O	60.2	60.1	59.9
CF_4	∠F-C-F	110.1	109.8	109.6
SF_6	∠F-S-F	89.9	89.8	90.0
SO_2	∠O-S-O	115.6	116.5	117.4

Table S11. Distortion energies of the 5^{12}, $5^{12}6^{2}$ and $5^{12}6^{4}$ hydrate clathrate cages for after the Greenhouse gases encapsulation computes at B3LYP-D3BJ/6-31G(d) level of theory and basis set. All the values in kcal/mol.

**GHGs**	**5^{12}**	**$5^{12}6^{2}$**	**$5^{12}6^{4}$**
CCl_4	-11.95	-29.73	-47.61
CF_2Cl_2	-22.78	-40.95	-50.86
CH_3Br	-34.71	-42.89	-50.44
CH_3Cl	-33.86	-42.93	-50.68
CH_4	-36.70	-44.45	-51.62
CO_2	-35.38	-43.67	-51.29
CO	-36.84	-44.55	-51.80
H_2S	-32.53	-40.32	-50.21
CH_3F	-39.31	-43.50	-51.05
N_2O	-36.20	-43.75	-51.81
NF_3	-36.05	-44.66	-52.04
O_3	-36.79	-44.64	-51.97
CF_4	-34.70	-44.58	-51.97
SF_6	-28.46	-43.79	-52.29
SO_2	-29.37	-40.96	-49.10

Table S12. Vibrational symmetric stretching frequencies and their IR intensity of the Greenhouse gases guest molecule encapsulated in 5^{12}, $5^{12}6^2$ and $5^{12}6^4$ hydrate clathrate cages.

Molecule	*Bond*	Before Encapsulation		5^{12}		$5^{12}6^2$		$5^{12}6^4$	
		IR Frequency	IR Intensity	IR Frequency	IR Intensity	IR Frequency	IR Intensity	IR Frequency	IR Intensity
CCl_4	C-Cl	452.209	0.000	457.785	24.601	475.271	9.238	460.257	2.609
CF_2Cl_2	C-Cl	442.196	0.668	468.330	21.761	456.798	1.252	445.600	5.912
	C-F	657.294	18.394	662.920	272.509	657.310	39.007	656.082	26.634
CH_3Br	C-H	594.826	13.938	592.641	76.073	577.227	42.277	580.075	16.913
	C-Br	3105.778	16.056	3149.456	13.757	3113.267	240.507	3113.407	7.011
CH_3Cl	C-H	720.122	29.166	703.148	160.165	692.902	11.868	708.460	25.935
	C-Cl	3094.238	23.064	3134.441	28.581	3110.096	3.904	3086.652	6.136
CH_4	C-H	3051.325	0.000	3054.111	1.889	3050.946	27.980	3049.101	23.971
CO_2	C-O	1372.078	0.000	1375.179	1.324	1372.968	1.123	1373.652	0.035
CO	C-O	2209.012	67.959	2197.808	47.176	2193.308	47.370	2203.480	46.886
H_2S	S-H	2691.566	11.101	3313.671	1161.147	3014.871	2630.410	2717.778	1509.557
CH_3F	C-H	1093.491	91.900	1019.289	9.446	1040.288	14.739	1063.681	24.654
	C-F	3037.116	32.136	3090.274	16.402	3067.078	6.397	3053.001	57.656
N_2O	N-O	1405.670	25.161	1411.628	13.236	1405.071	15.363	1404.634	18.749
NF_3	N-F	644.166	2.752	648.863	13.181	642.841	1.586	643.366	4.724
O_3	O-O	1210.818	0.000	1189.348	0.485	1190.267	1.157	1202.836	0.094
CF_4	C-F	906.234	0.000	914.284	3.530	905.106	120.190	904.974	41.050
SF_6	S-F	730.536	0.000	735.701	35.891	722.073	2.220	720.550	3.135
SO_2	S-O	1141.349	27.654	1147.552	44.948	1134.104	30.434	1137.117	43.096

Table S13. Total energies of the Greenhouse gases encapsulation in 5^{12}, $5^{12}6^{2}$ and $5^{12}6^{4}$ hydrate clathrate cages computes at B3LYP/6-31G(d) and B3LYP-D3BJ/6-31G(d) level of theory and basis sets. All the values in Hartree.

Greenhouse gases	5^{12}		$5^{12}6^{2}$		$5^{12}6^{4}$	
	B3LYP/6-31G(d)	B3LYP-D3BJ/6-31G(d)	B3LYP/6-31G(d)	B3LYP-D3BJ/6-31G(d)	B3LYP/6-31G(d)	B3LYP-D3BJ/6-31G(d)
CCl_4	-3407.44	-3407.55	-3713.20	-3713.32	-4018.96	-4019.1
CF_2Cl_2	-2689.76	-2686.86	-2992.53	-2992.64	-3298.27	-3298.39
CH_3Br	-4140.26	-4140.36	-4446.01	-4446.11	-4751.74	-4751.84
CH_3Cl	-2028.74	-2028.83	-2334.49	-2334.59	-2640.22	-2640.33
CH_4	-1569.15	-1569.23	-1874.90	-1874.98	-2180.63	-2180.72
CO	-1641.94	-1717.3	-1947.68	-2023.05	-2253.42	-2328.79
CO_2	-1717.22	-1642.02	-2328.69	-1947.77	-2328.69	-2253.51
H_2S	-1928.03	-1928.11	-2539.50	-2233.86	-2539.50	-2539.6
CH_3F	-1668.39	-1668.47	-1974.12	-1974.21	-2279.85	-2279.94
N_2O	-1713.06	-1713.14	-2324.54	-2018.89	-2324.54	-2324.63
NF_3	-1882.71	-1882.78	-2188.45	-2188.54	-2494.18	-2494.28
O_3	-1754.00	-1754.07	-2059.74	-2059.82	-2365.47	-2365.56
CF_4	-1966.11	-1966.19	-2271.86	-2271.95	-2577.59	-2577.69
SF_6	-2525.72	-2525.82	-2831.49	-2831.6	-3137.22	-3137.34
SO_2	-2077.27	-2077.33	-2382.98	-2383.07	-2688.71	-2688.81

Table S14. Global Chemical indices of Greenhouse gases within 5^{12} hydrate clathrates. All values in eV.

Energy gap (E_g) $= E_{LUMO} - E_{HOMO}$ Chemical potential (μ) $= \frac{E_{HOMO}+E_{LUMO}}{2}$ Chemical Hardness (η) $= \frac{E_{LUMO}-E_{HOMO}}{2}$ Electronegativity (χ) $= \frac{-(E_{HOMO}+E_{LUMO})}{2}$ Electrophilicity (ω) $== \frac{-\eta^2}{2\chi}$

Molecules	HOMO	LUMO	E_g	μ	η	χ	ω
CCl_4	-7.04	-1.67	5.38	-4.36	2.69	4.36	0.02
CF_2Cl_2	-6.8	-0.24	6.56	-3.52	3.28	3.52	0.03
CH_3Br	-7.01	0.04	7.05	-3.49	3.53	3.49	0.03
CH_3Cl	-6.75	0.458	7.21	-3.15	3.6	3.15	0.03
CH_4	-6.7	0.237	6.94	-3.23	3.47	3.23	0.03
CO_2	-6.64	0.303	6.95	-3.17	3.47	3.17	0.03
CO	-6.7	-0.45	6.25	-3.58	3.13	3.58	0.02
H_2S	-5.92	0.466	6.38	-2.73	3.19	2.73	0.02
CH_3F	-6.94	0.502	7.44	-3.22	3.72	3.22	0.03
N_2O	-6.59	-3.86	2.72	-5.23	1.36	5.23	0.01
NF_3	-6.71	-0.02	6.69	-3.36	3.35	3.36	0.03
O_3	-6.71	-4.55	2.16	-5.63	1.08	5.63	0.00
CF_4	-6.71	0.312	7.03	-3.20	3.51	3.20	0.03
SF_6	-6.86	-2.24	4.61	-4.55	2.31	4.55	0.02
SO_2	-6.75	-1.73	5.03	-4.24	2.51	4.24	0.02

Table S15. Global Chemical indices of Greenhouse gases within $5^{12}6^{2}$ hydrate clathrates. All values in eV.

Molecules	HOMO	LUMO	E_g	μ	η	χ	ω
CCl_4	-6.93	-1.59	5.34	-4.26	2.67	4.26	0.02
CF_2Cl_2	-6.76	-0.53	6.23	-3.64	3.12	3.64	0.02
CH_3Br	-6.88	0.038	6.92	-3.42	3.46	3.42	0.03
CH_3Cl	-6.73	0.136	6.86	-3.29	3.43	3.29	0.03
CH_4	-6.72	0.135	6.85	-3.29	3.43	3.29	0.03
CO_2	-7.02	0.183	7.20	-3.42	3.6	3.42	0.03
CO	-7.13	-0.47	6.65	-3.8	3.33	3.8	0.03
H_2S	-6.65	0.189	6.84	-3.23	3.42	3.23	0.03
CH_3F	-6.99	0.014	7.00	-3.49	3.5	3.49	0.03
N_2O	-7.09	-2.65	4.44	-4.87	2.22	4.87	0.02
NF_3	-6.69	-0.05	6.64	-3.37	3.32	3.37	0.03
O_3	-7.04	-5.03	2.01	-6.03	1.01	6.03	0.00
CF_4	-7.35	0.258	7.61	-3.55	3.81	3.55	0.03
SF_6	-6.91	-2.58	4.33	-4.75	2.17	4.75	0.02
SO_2	-7.16	-2.41	4.75	-4.78	2.38	4.78	0.02

Table S16. Global Chemical indices of Greenhouse gases within $5^{12}6^4$ hydrate clathrates. All values in eV.

**Molecules**	**HOMO**	**LUMO**	E_g	μ	η	χ	ω
CCl_4	-6.91	-1.78	5.13	-4.35	2.57	4.35	0.02
CF_2Cl_2	-7.1	-0.60	6.50	-3.85	3.25	3.85	0.03
CH_3Br	-6.87	-0.12	6.75	-3.5	3.38	3.50	0.03
CH_3Cl	-6.89	0.17	7.06	-3.36	3.53	3.36	0.03
CH_4	-6.85	0.20	7.05	-3.33	3.53	3.33	0.03
CO_2	-6.79	0.24	7.03	-3.28	3.52	3.28	0.03
CO	-6.86	-1.02	5.84	-3.94	2.92	3.94	0.02
H_2S	-6.91	0.24	7.15	-3.33	3.57	3.33	0.03
CH_3F	-6.97	0.13	7.11	-3.42	3.55	3.42	0.03
N_2O	-6.75	-2.54	4.21	-4.64	2.11	4.64	0.01
NF_3	-7.41	-0.52	6.89	-3.96	3.45	3.96	0.03
O_3	-6.95	-4.70	2.25	-5.83	1.13	5.83	0.00
CF_4	-6.9	0.27	7.17	-3.31	3.58	3.31	0.03
SF_6	-6.89	-2.79	4.09	-4.84	2.05	4.84	0.01
SO_2	-7.28	-2.08	5.21	-4.68	2.60	4.68	0.02

Figure S1. Mulliken charges of the Greenhouse gases monomers before encapsulation in clathrate hydrate computes at B3LYP/6-31G(d) level of theory and basis set.

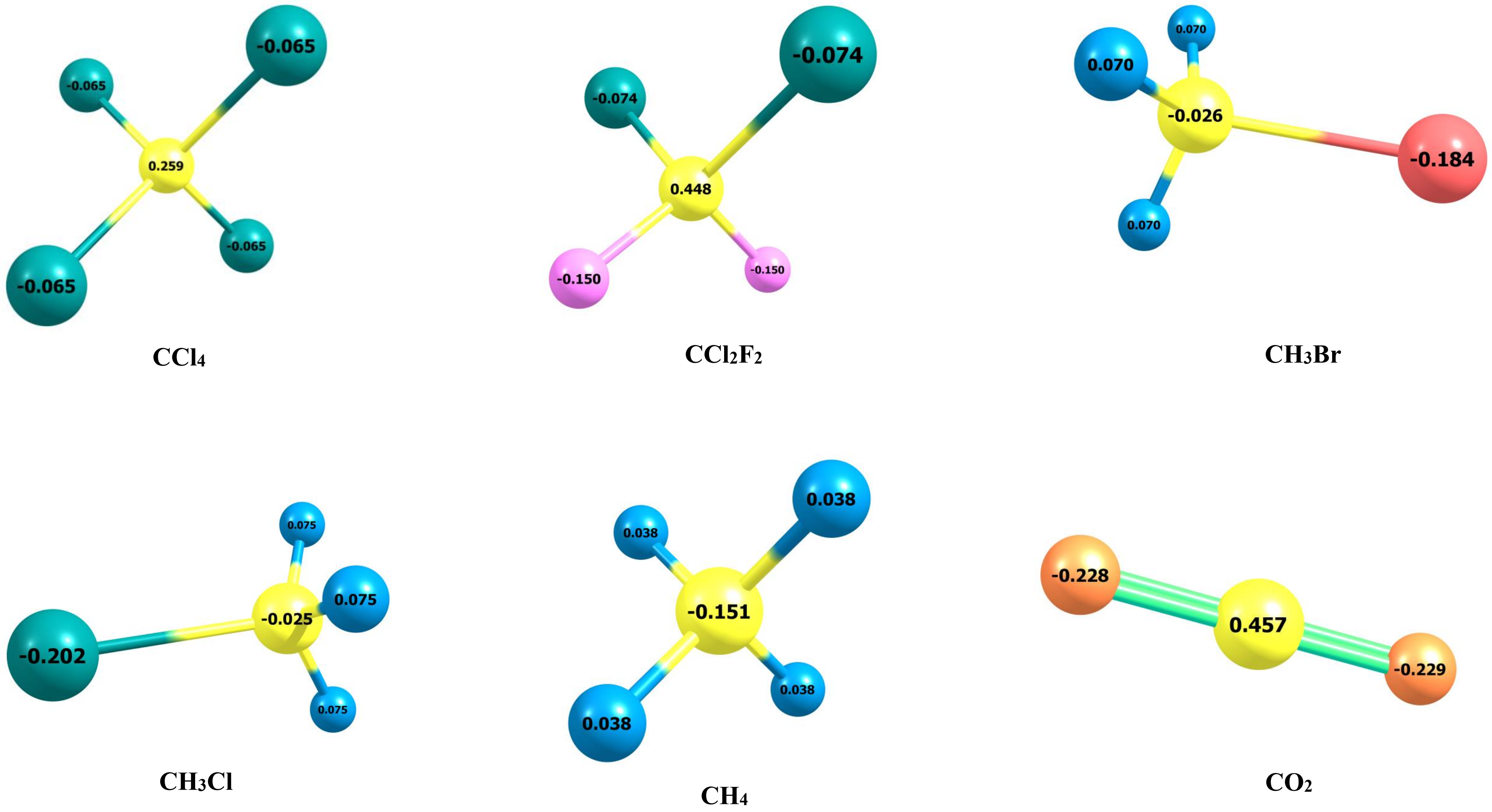

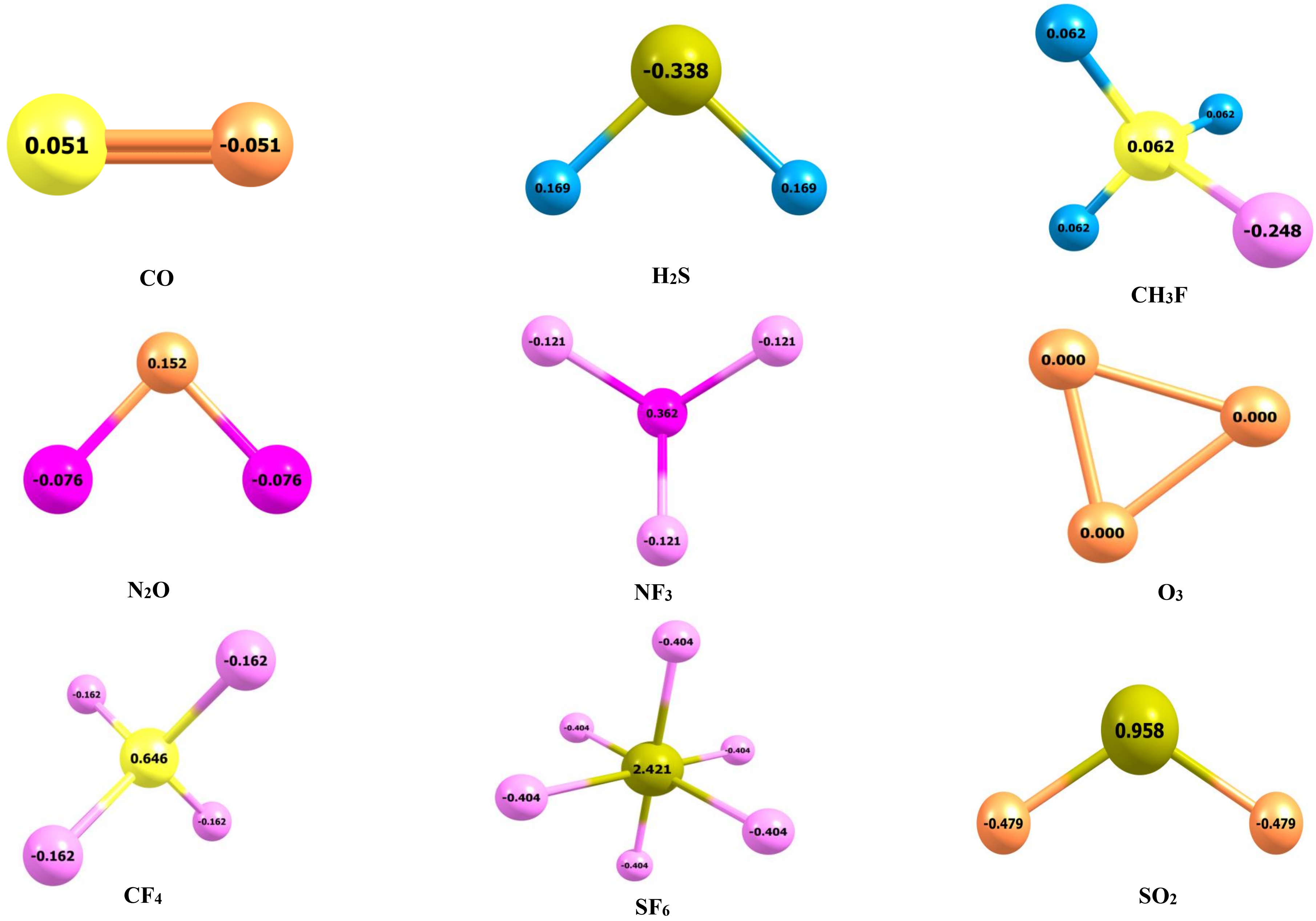
0.051
-0.051
CO
-0.338
0.169
0.169
H2S
0.062
0.062
0.062
0.062
-0.248
CH3F
0.152
-0.076
-0.076
N2O
-0.121
-0.121
0.362
-0.121
NF3
0.000
0.000
0.000
O3
-0.162
-0.162
0.646
-0.162
-0.162
CF4
-0.404
-0.404
-0.404
2.421
-0.404
-0.404
-0.404
SF6
0.958
-0.479
-0.479
SO2

S32

Figure S2. Mulliken charges of the empty 5^{12}, $5^{12}6^2$ and $5^{12}6^4$ clathrate hydrate monomers computes at B3LYP/6-31G(d) level of theory and basis set.

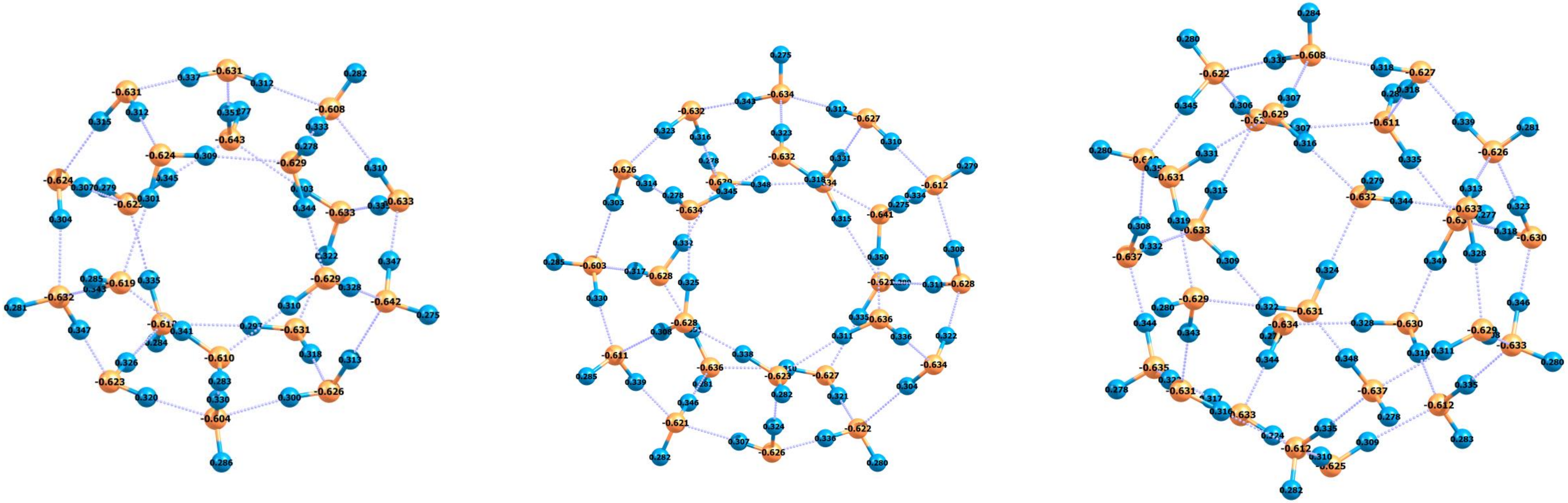

5^{12} **$5^{12}6^2$** **$5^{12}6^4$**

Figure S3. Mulliken charges of the CCl_4 molecule within 5^{12}, $5^{12}6^2$ and $5^{12}6^4$ clathrate hydrates computes at B3LYP/6-31G(d) level of theory and basis set.

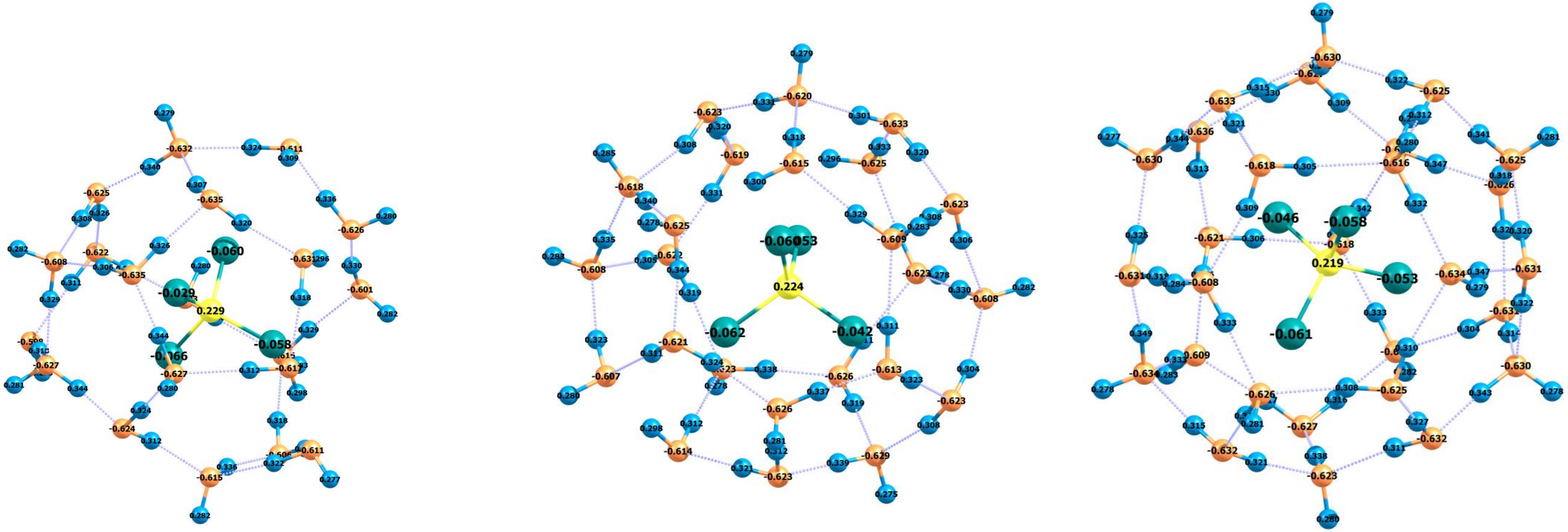

5^{12} **$5^{12}6^2$** **$5^{12}6^4$**

Figure S4. Mulliken charges of the CF_2Cl_2 molecule within 5^{12}, $5^{12}6^2$ and $5^{12}6^4$ clathrate hydrates computes at B3LYP/6-31G(d) level of theory and basis set.

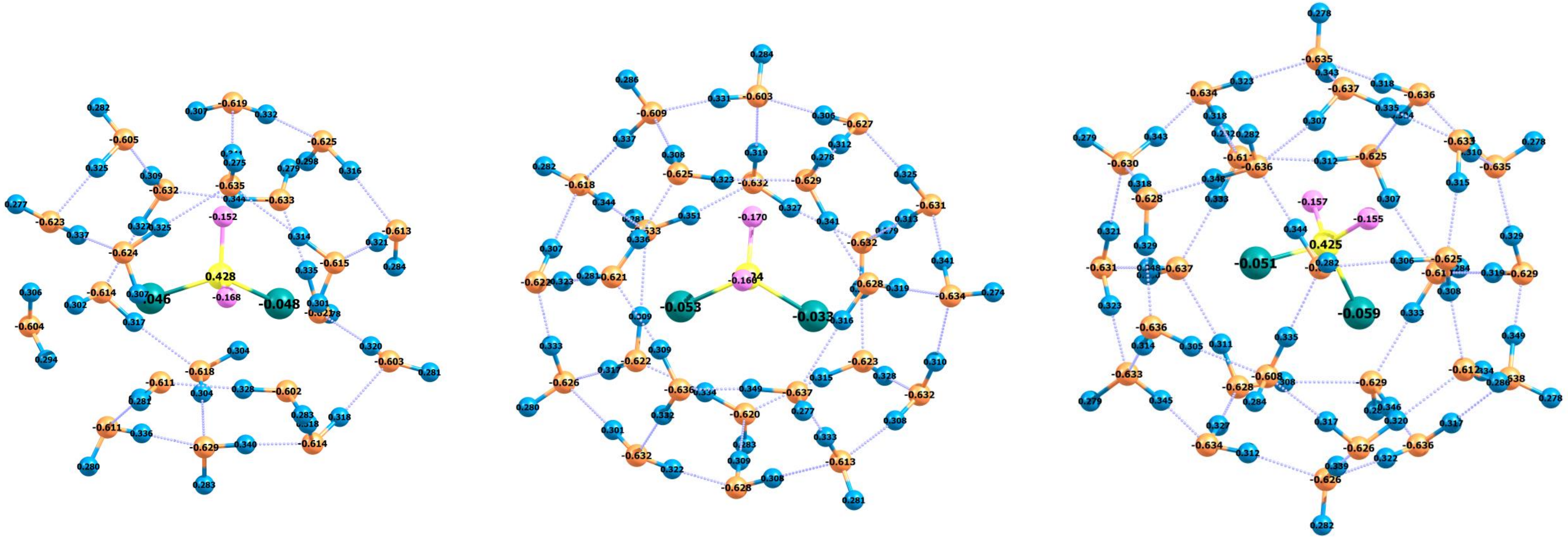

5^{12} **$5^{12}6^2$** **$5^{12}6^4$**

Figure S5. Mulliken charges of the CH_3Br molecule within 5^{12}, $5^{12}6^2$ and $5^{12}6^4$ clathrate hydrates computes at B3LYP/6-31G(d) level of theory and basis set.

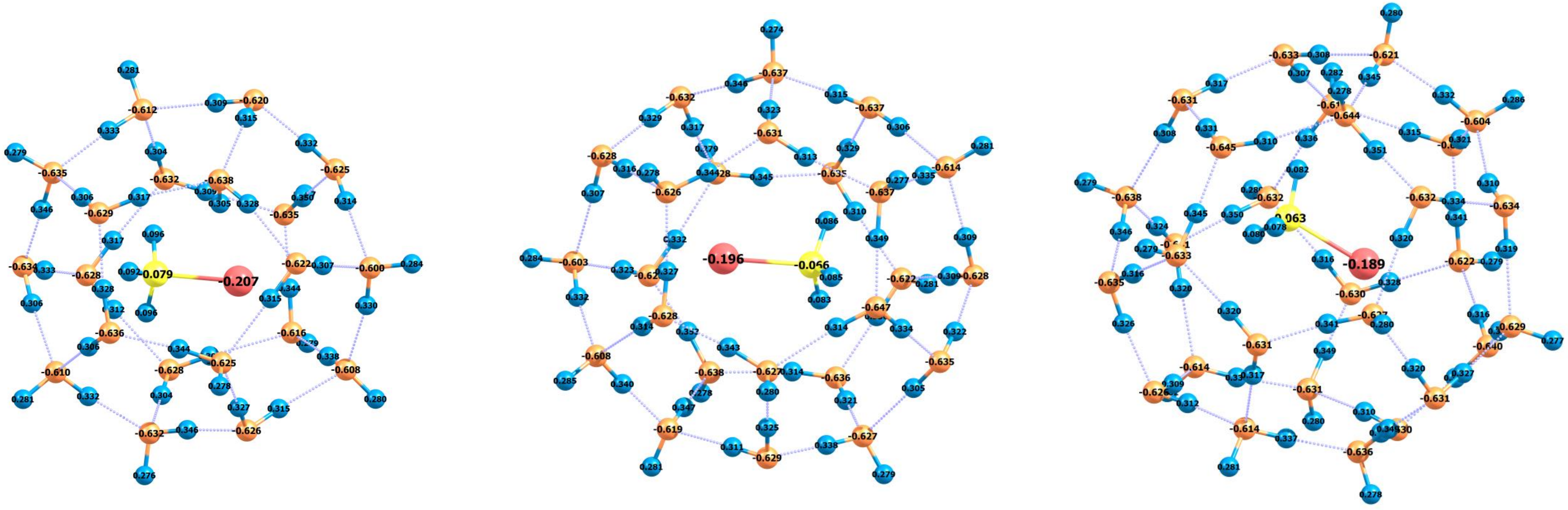

5^{12} **$5^{12}6^2$** **$5^{12}6^4$**

Figure S6. Mulliken charges of the CH_3Cl molecule within 5^{12}, $5^{12}6^2$ and $5^{12}6^4$ clathrate hydrates computes at B3LYP/6-31G(d) level of theory and basis set.

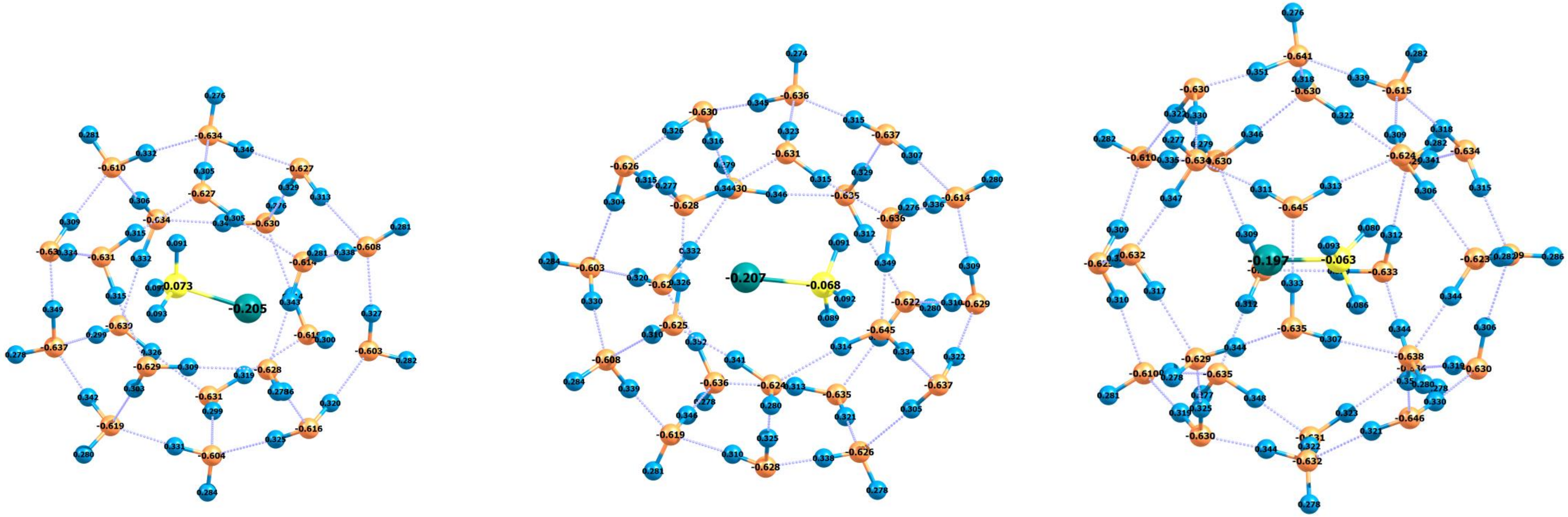

5^{12} **$5^{12}6^2$** **$5^{12}6^4$**

Figure S7. Mulliken charges of the CH_4 molecule within 5^{12}, $5^{12}6^2$ and $5^{12}6^4$ clathrate hydrates computes at B3LYP/6-31G(d) level of theory and basis set.

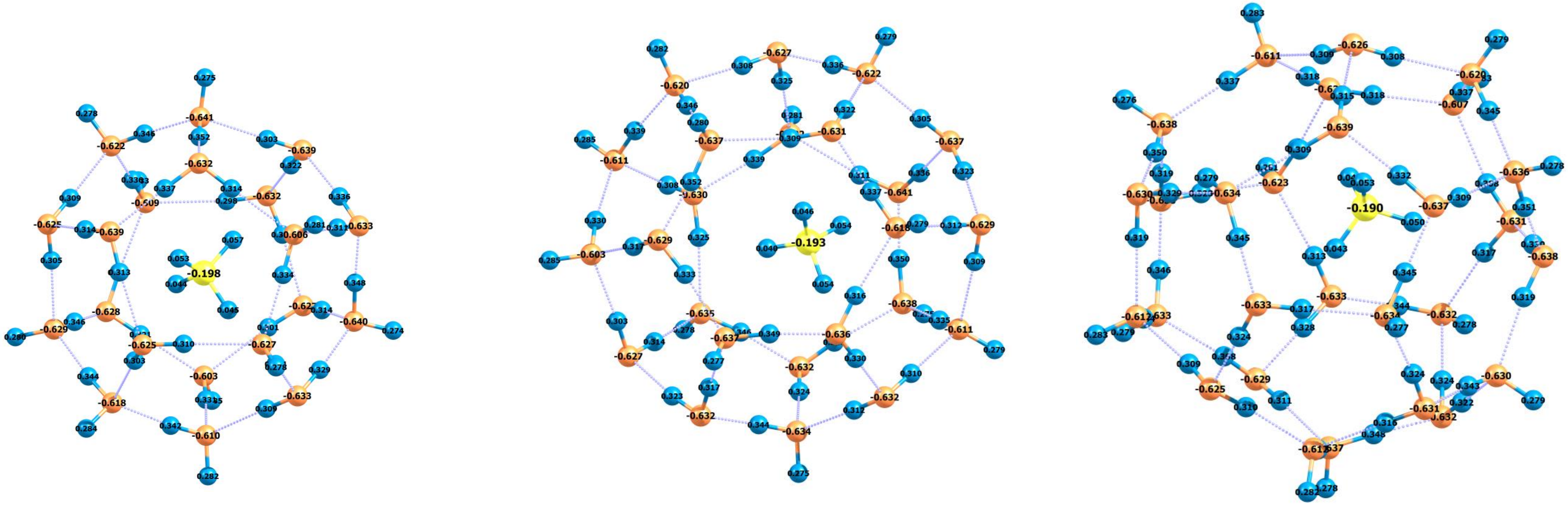

5^{12} **$5^{12}6^2$** **$5^{12}6^4$**

Figure S8. Mulliken charges of the CO_2 molecule within 5^{12}, $5^{12}6^2$ and $5^{12}6^4$ clathrate hydrates computes at B3LYP/6-31G(d) level of theory and basis set.

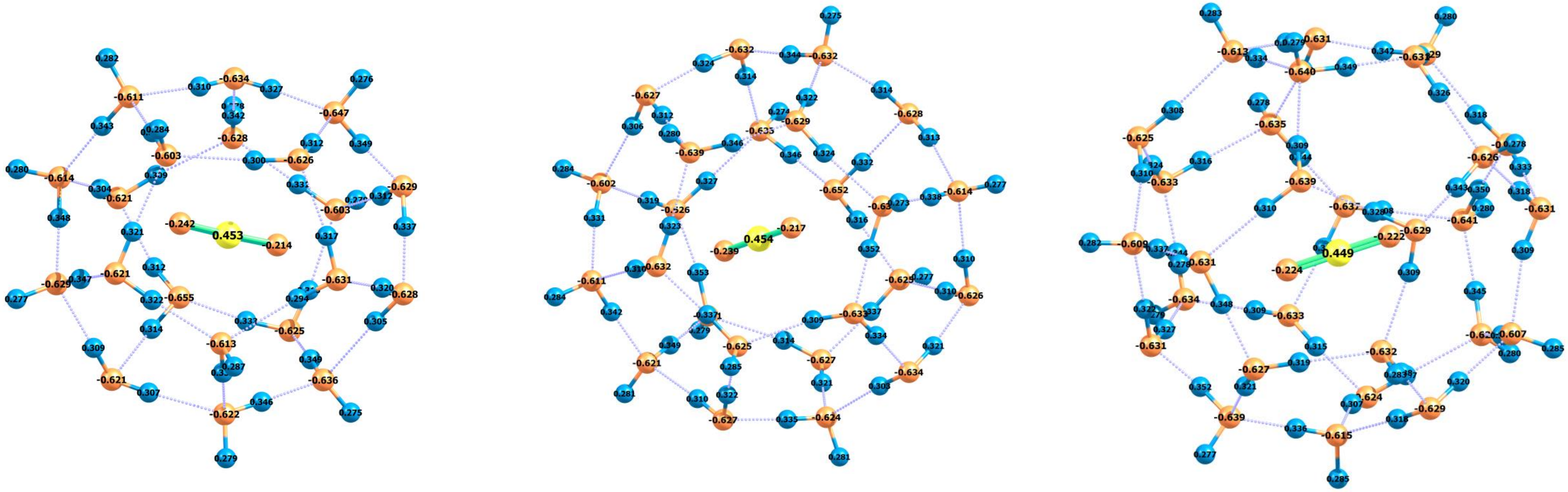

5^{12} **$5^{12}6^2$** **$5^{12}6^4$**

Figure S9. Mulliken charges of the CO molecule within 5^{12}, $5^{12}6^2$ and $5^{12}6^4$ clathrate hydrates computes at B3LYP/6-31G(d) level of theory and basis set.

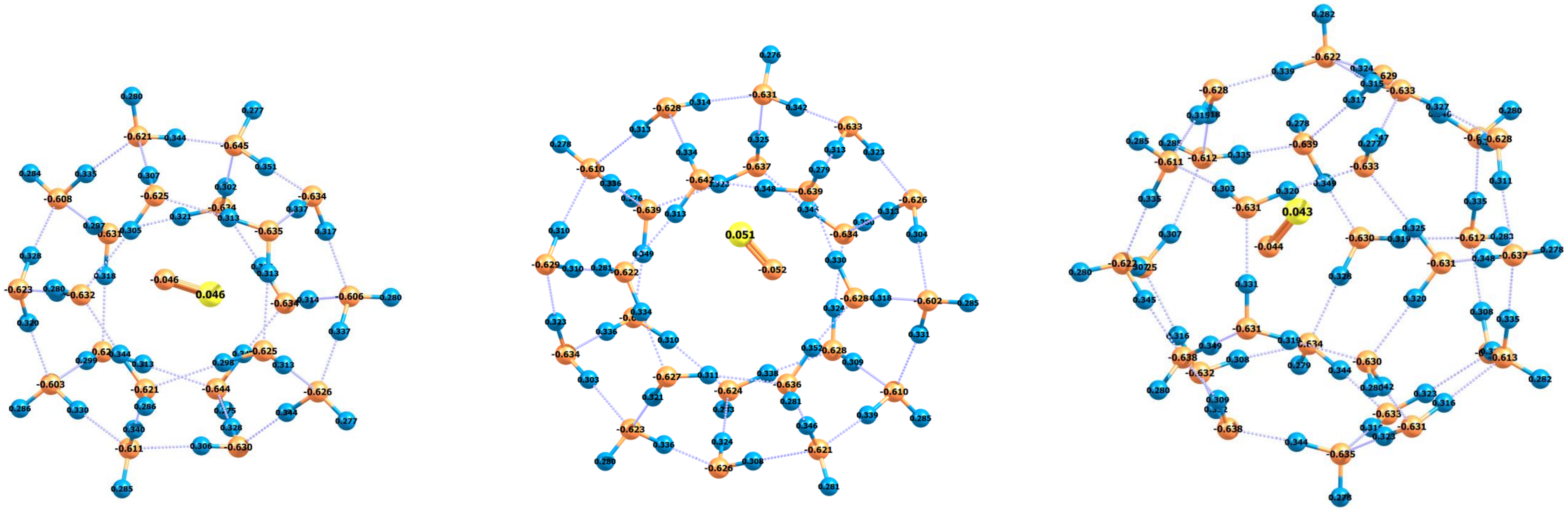

5^{12} **$5^{12}6^2$** **$5^{12}6^4$**

Figure S10. Mulliken charges of the H_2S molecule within 5^{12}, $5^{12}6^2$ and $5^{12}6^4$ clathrate hydrates computes at B3LYP/6-31G(d) level of theory and basis set.

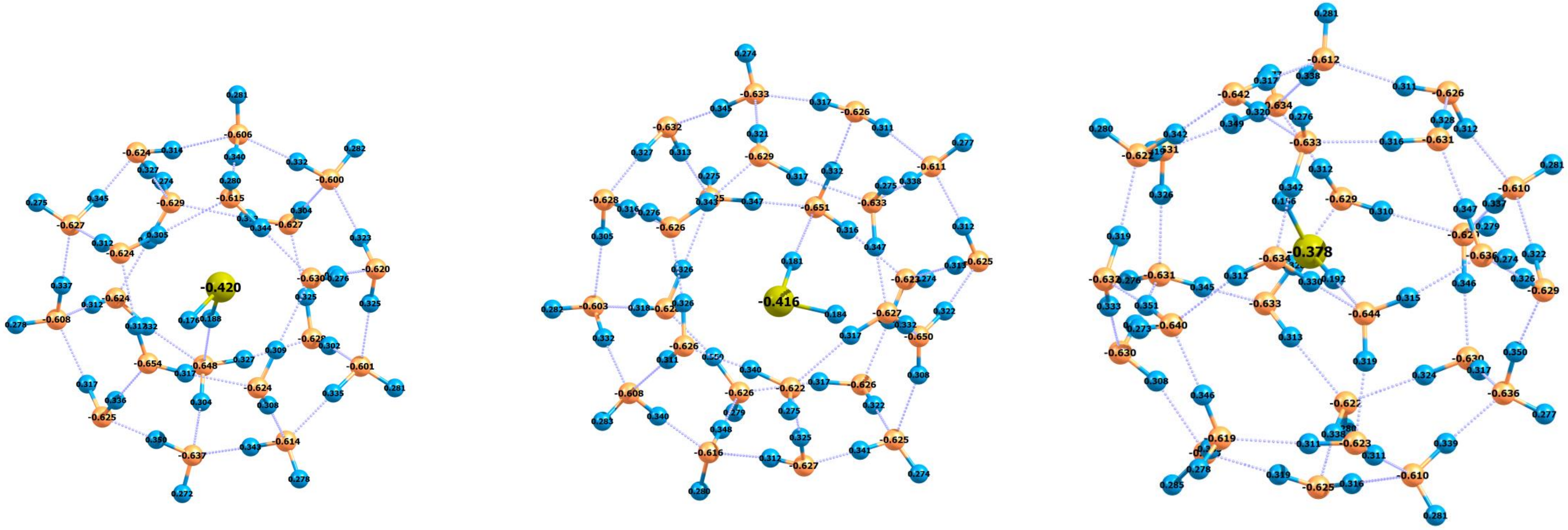

5^{12} **$5^{12}6^2$** **$5^{12}6^4$**

Figure S11. Mulliken charges of the CH_3F molecule within 5^{12}, $5^{12}6^2$ and $5^{12}6^4$ clathrate hydrates computes at B3LYP/6-31G(d) level of theory and basis set.

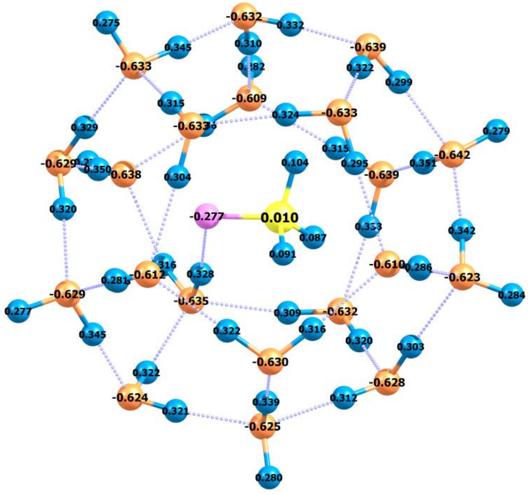

5^{12}

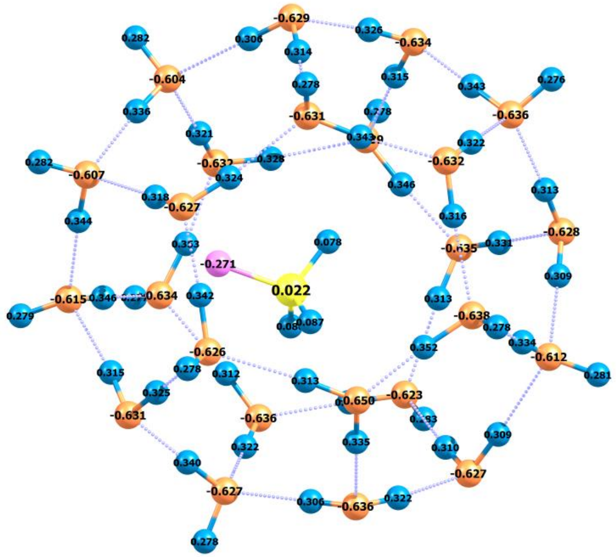

$5^{12}6^2$

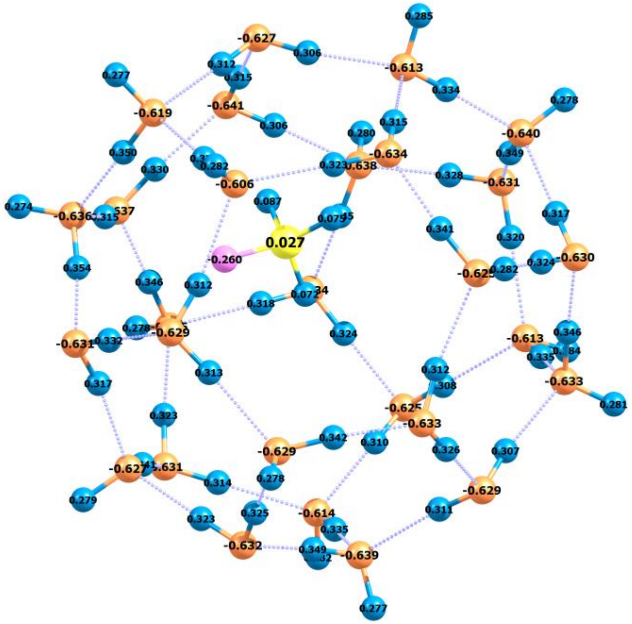

$5^{12}6^4$

Figure S12. Mulliken charges of the N_2O molecule within 5^{12}, $5^{12}6^2$ and $5^{12}6^4$ clathrate hydrates computes at B3LYP/6-31G(d) level of theory and basis set.

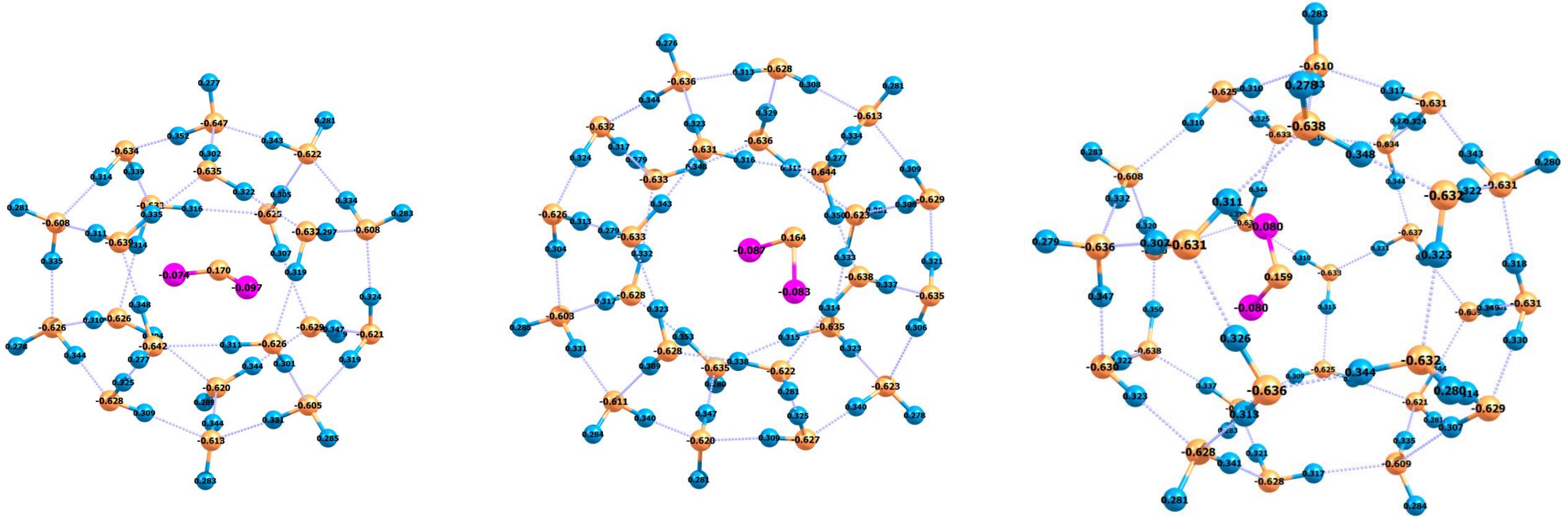

5^{12} **$5^{12}6^2$** **$5^{12}6^4$**

Figure S13. Mulliken charges of the NF_3 molecule within 5^{12}, $5^{12}6^2$ and $5^{12}6^4$ clathrate hydrates computes at B3LYP/6-31G(d) level of theory and basis set.

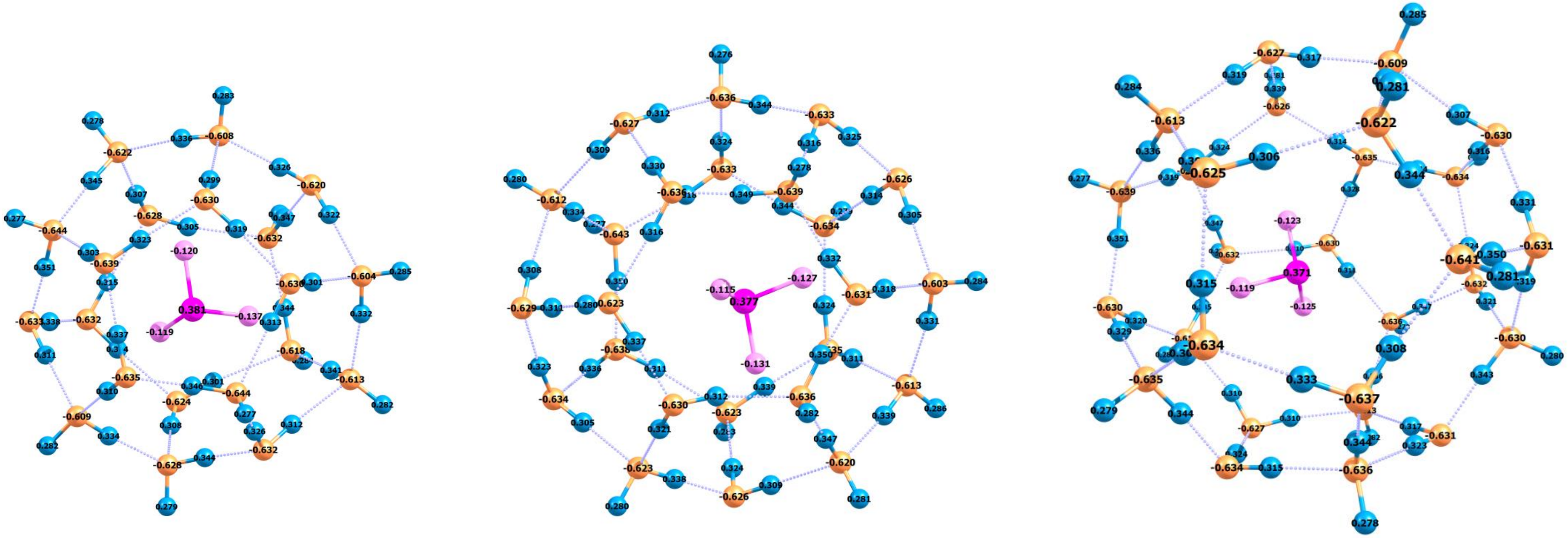

5^{12} **$5^{12}6^2$** **$5^{12}6^4$**

Figure S14. Mulliken charges of the O_3 molecule within 5^{12}, $5^{12}6^2$ and $5^{12}6^4$ clathrate hydrates computes at B3LYP/6-31G(d) level of theory and basis set.

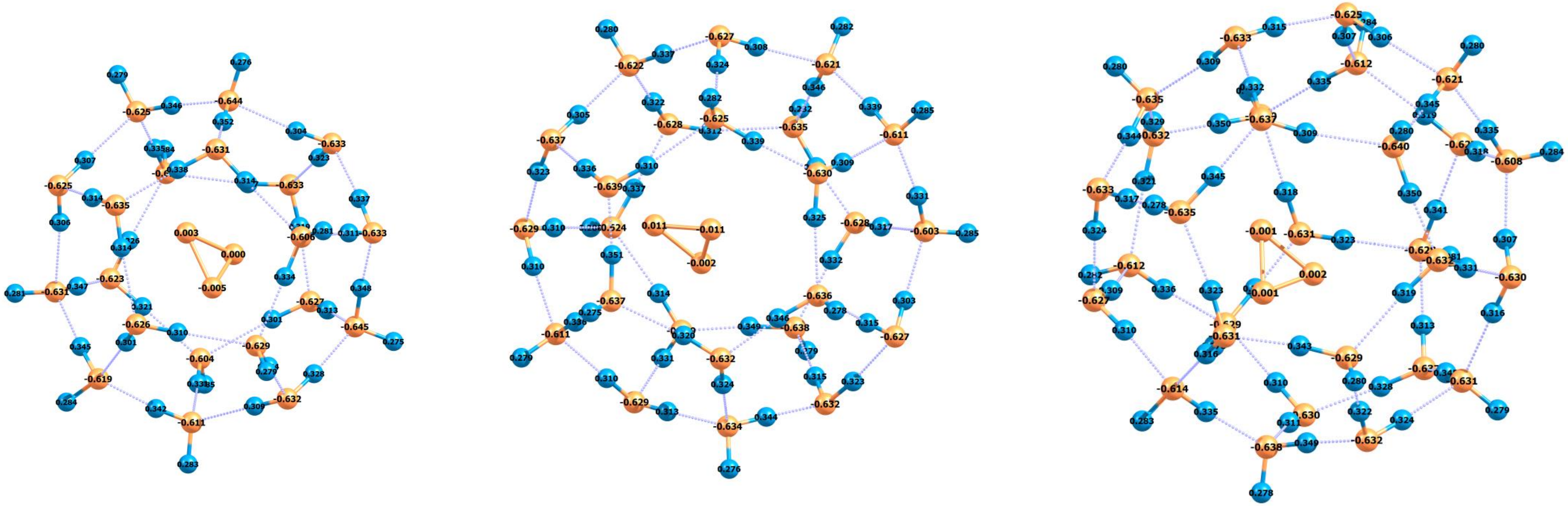

$\mathbf{5^{12}}$ $\mathbf{5^{12}6^2}$ $\mathbf{5^{12}6^4}$

Figure S15. Mulliken charges of the CF_4 molecule within 5^{12}, $5^{12}6^2$ and $5^{12}6^4$ clathrate hydrates computes at B3LYP/6-31G(d) level of theory and basis set.

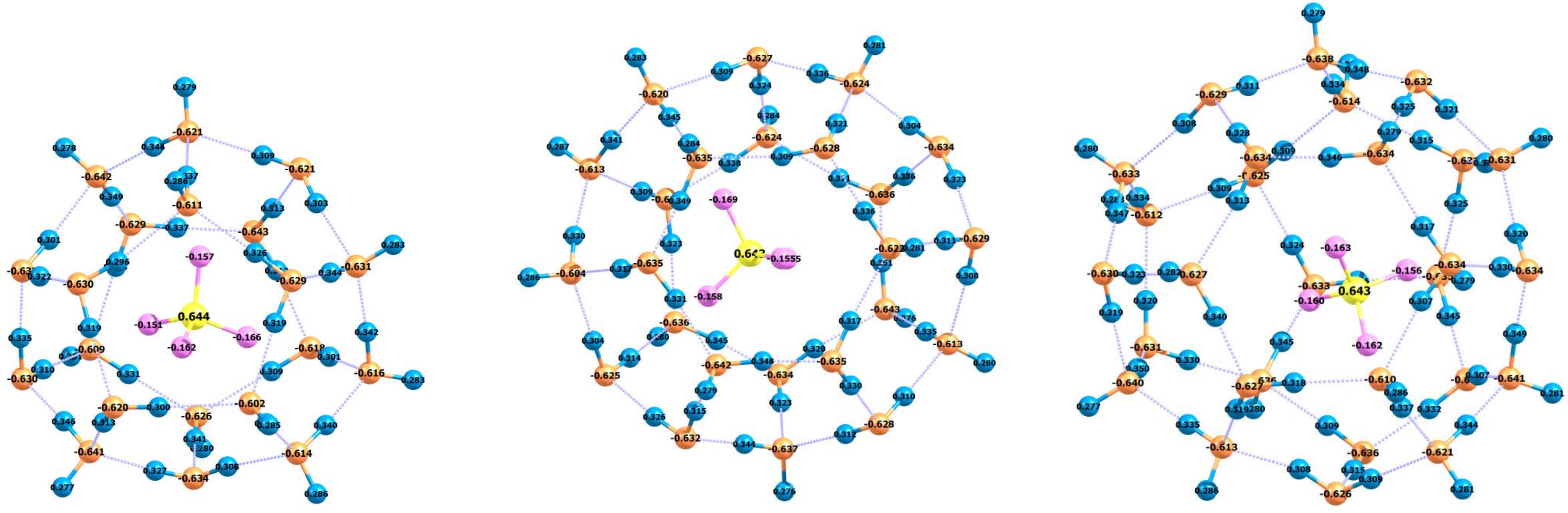

5^{12} **$5^{12}6^2$** **$5^{12}6^4$**

Figure S16. Mulliken charges of the SF_6 molecule within 5^{12}, $5^{12}6^2$ and $5^{12}6^4$ clathrate hydrates computes at B3LYP/6-31G(d) level of theory and basis set.

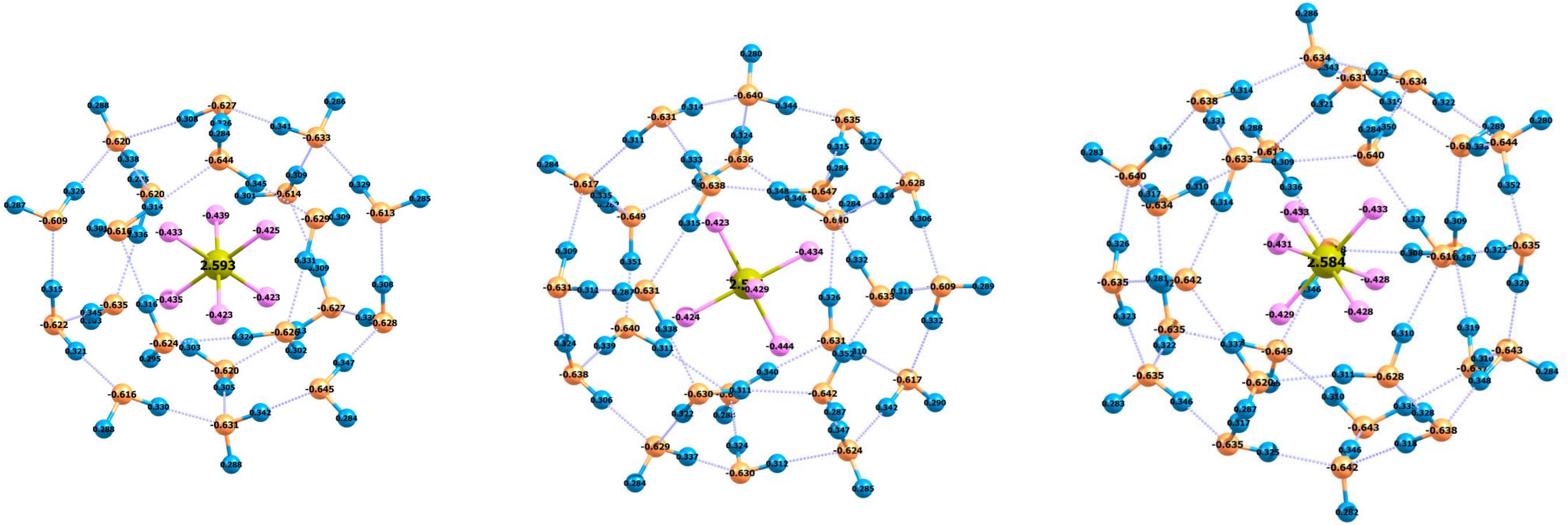

5^{12} **$5^{12}6^2$** **$5^{12}6^4$**

Figure S17. Mulliken charges of the SO_2 molecule within 5^{12}, $5^{12}6^2$ and $5^{12}6^4$ clathrate hydrates computes at B3LYP/6-31G(d) level of theory and basis set.

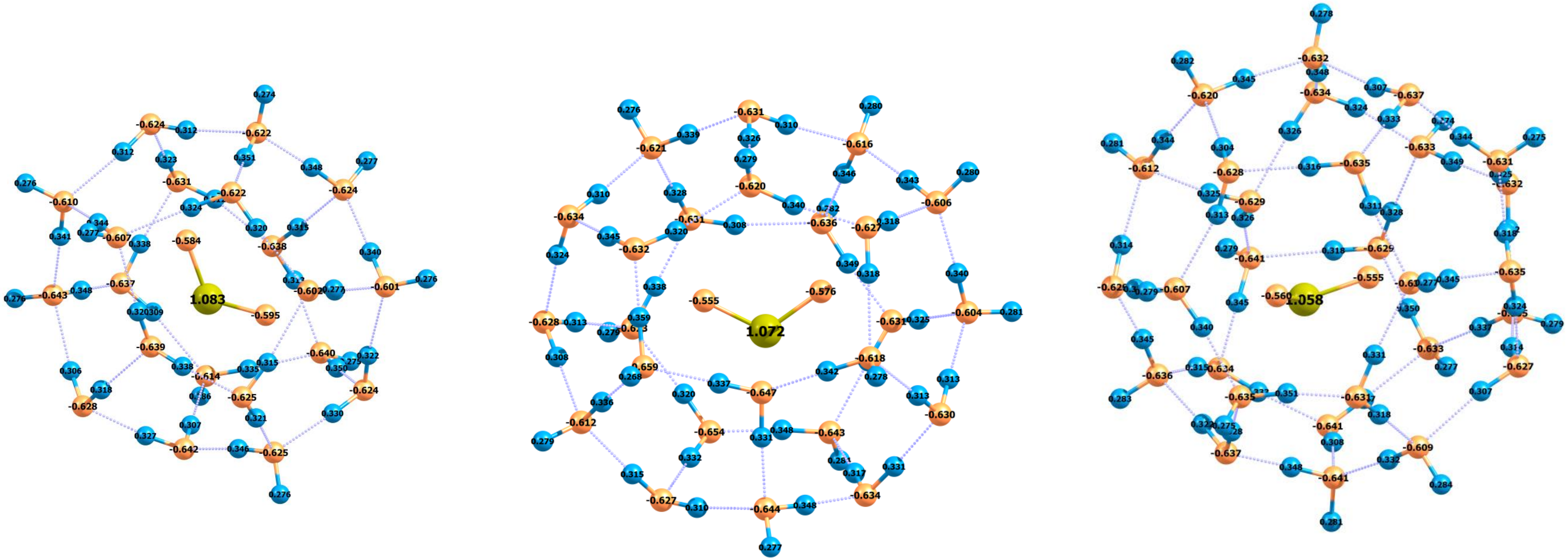

5^{12} **$5^{12}6^2$** **$5^{12}6^4$**

Figure S18. Frontier molecular orbital of the empty 5^{12}, $5^{12}6^{2}$ and $5^{12}6^{4}$ clathrate hydrates computes at B3LYP/6-31G(d) level of theory and basis set.

HOMO **LUMO**

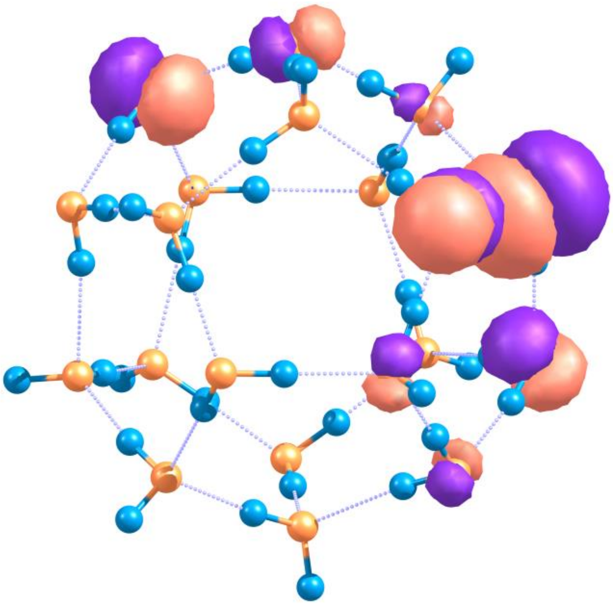

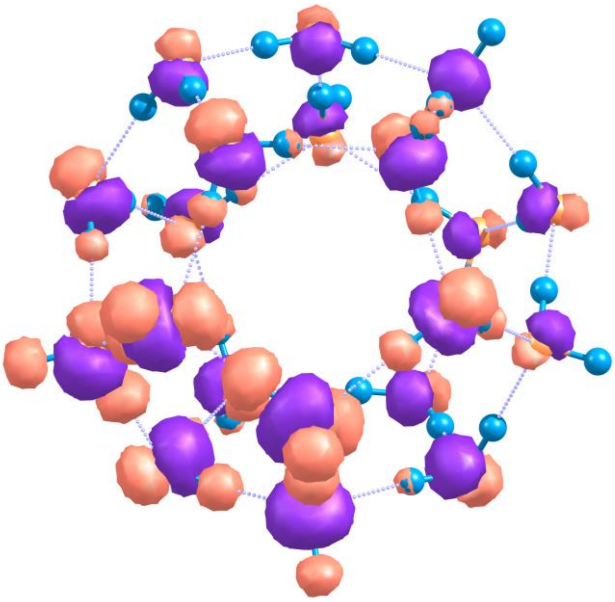

5^{12}

HOMO **LUMO**

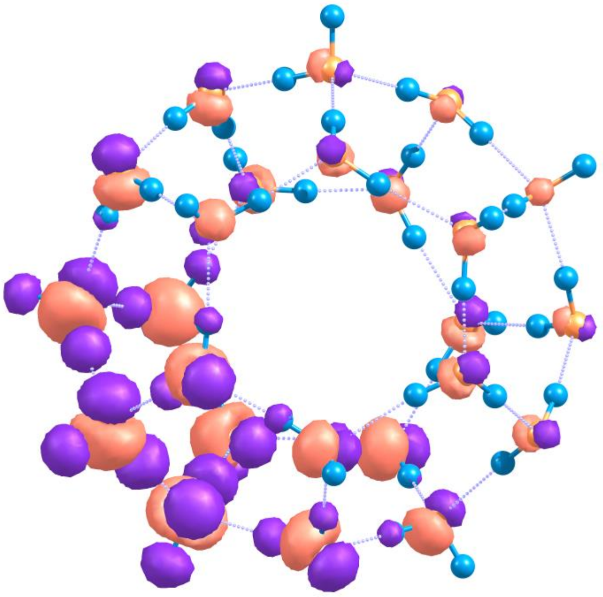

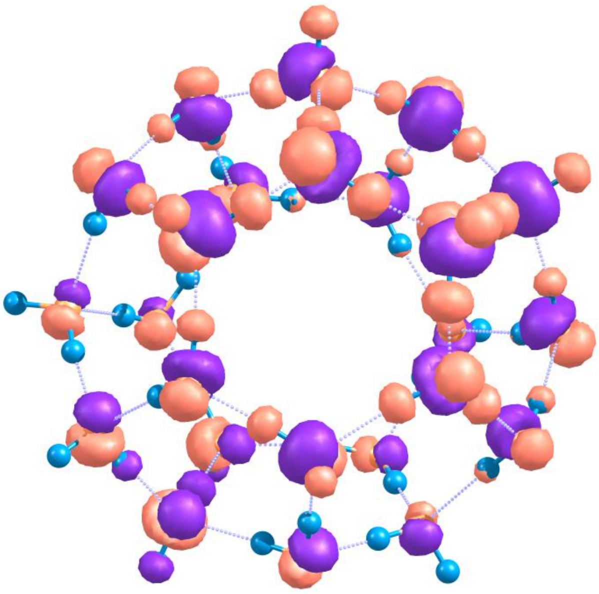

$5^{12}6^{2}$

HOMO

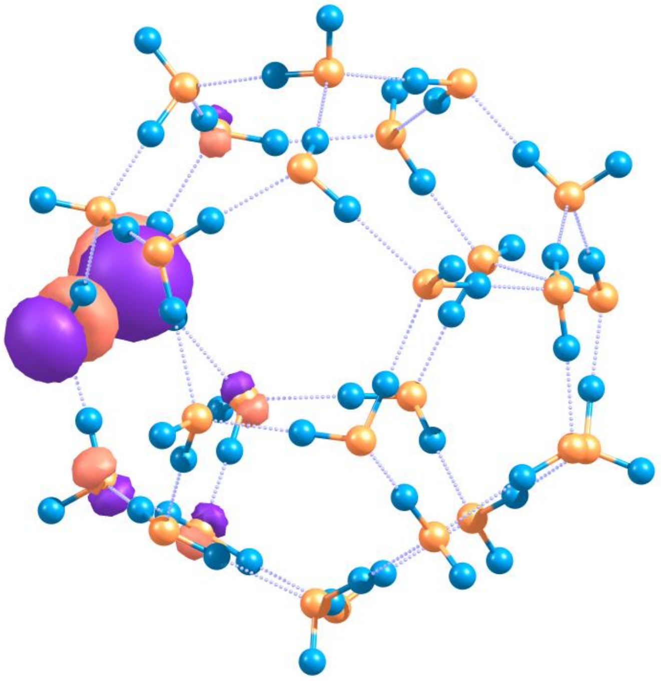

LUMO

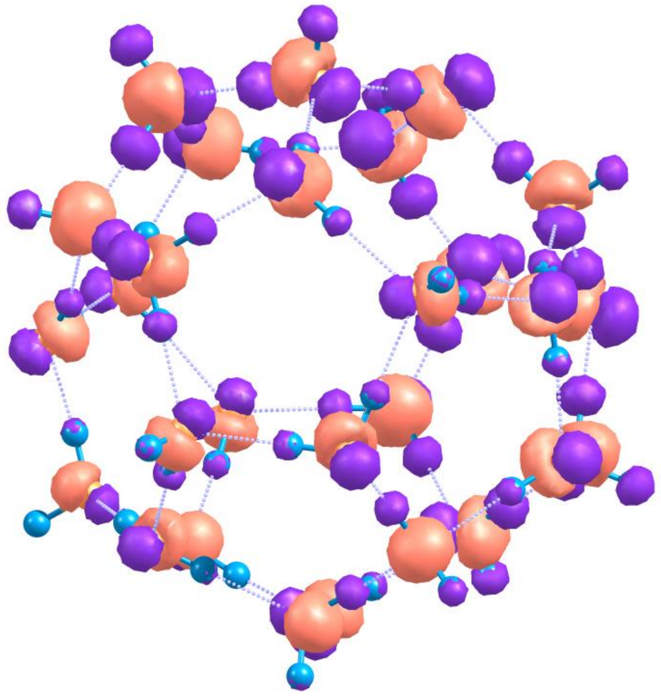

$5^{12}6^{4}$

Figure S19. Frontier molecular orbital of the CCl_4 molecule before and after encapsulation in 5^{12}, $5^{12}6^{2}$ and $5^{12}6^{4}$ clathrate hydrates computes at B3LYP/6-31G(d) level of theory and basis set.

Before Encapsulation

HOMO

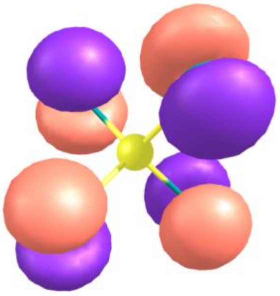

LUMO

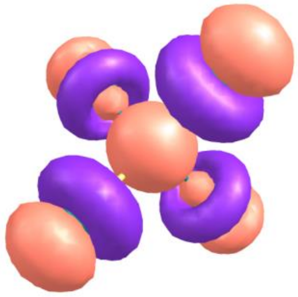

After Encapsulation

HOMO

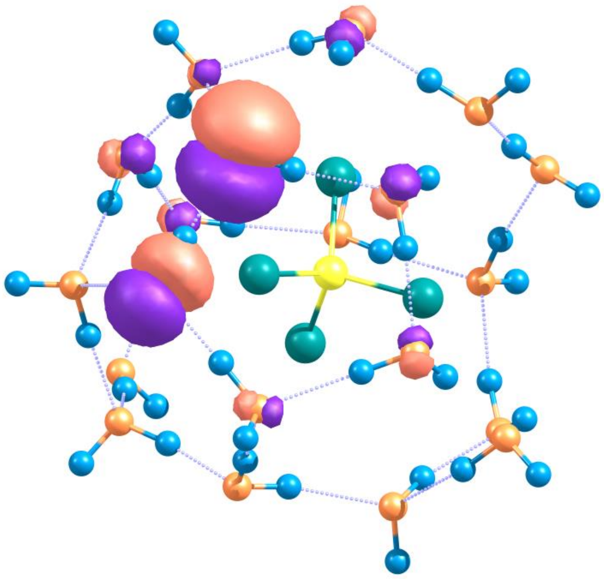

LUMO

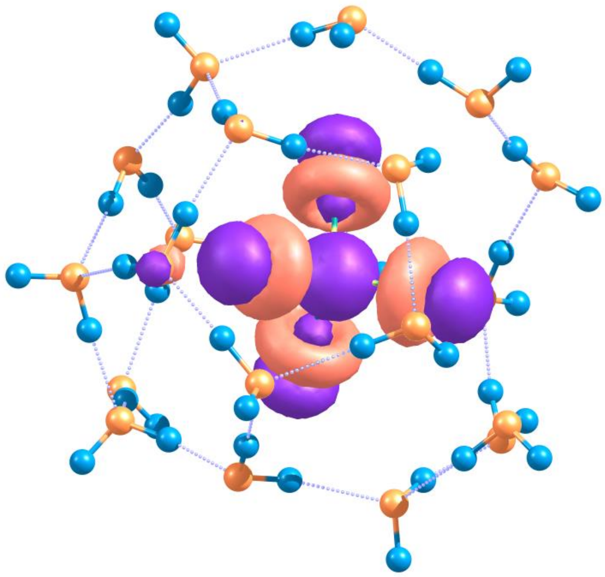

5^{12}

HOMO

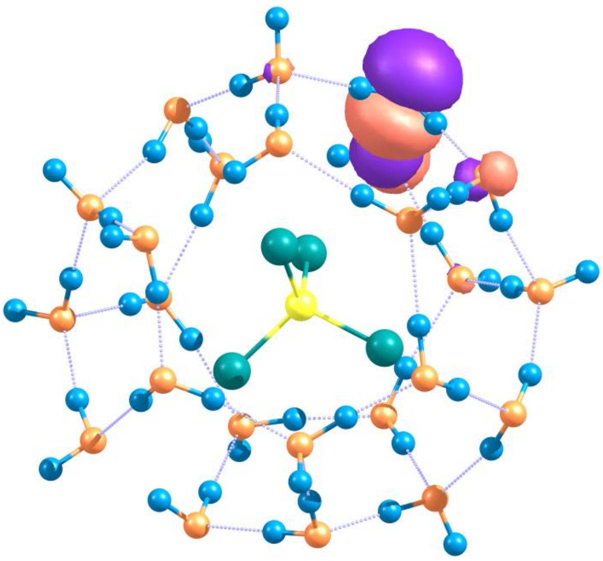

LUMO

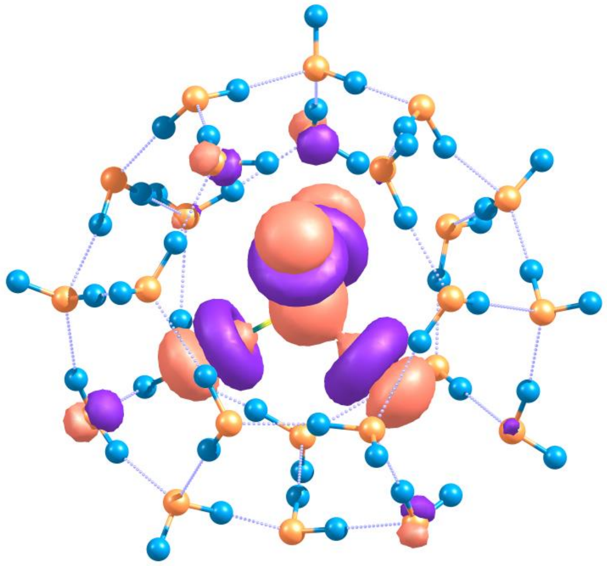

$5^{12}6^{2}$

HOMO

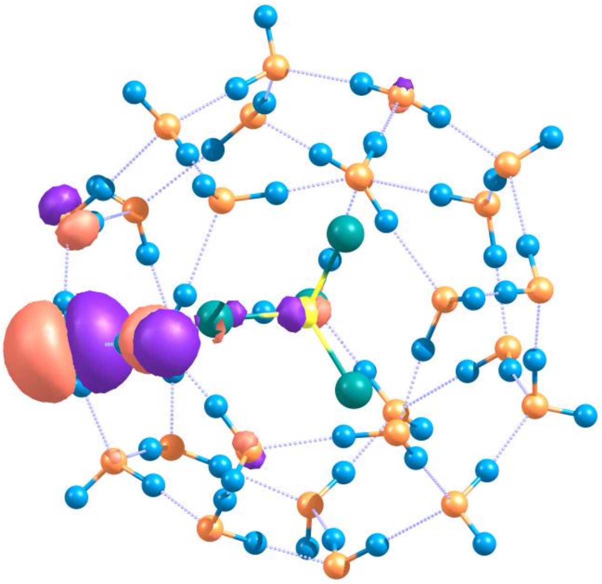

LUMO

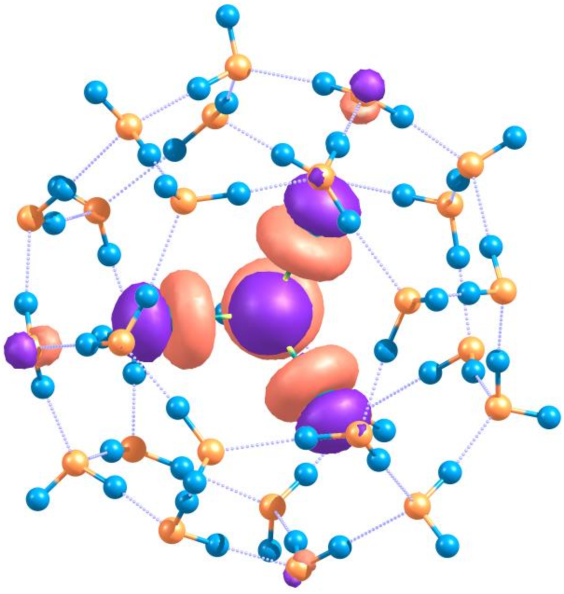

$5^{12}6^{4}$

Figure S20. Frontier molecular orbital of the CF_2Cl_2 molecule before and after encapsulation in 5^{12}, $5^{12}6^2$ and $5^{12}6^4$ clathrate hydrates computes at B3LYP/6-31G(d) level of theory and basis set.

Before Encapsulation

HOMO

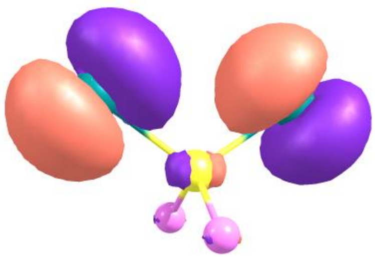

LUMO

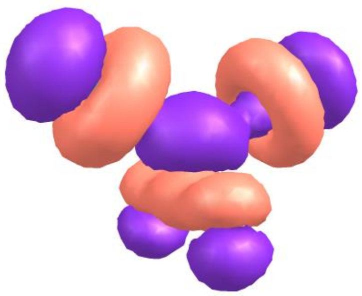

After Encapsulation

HOMO

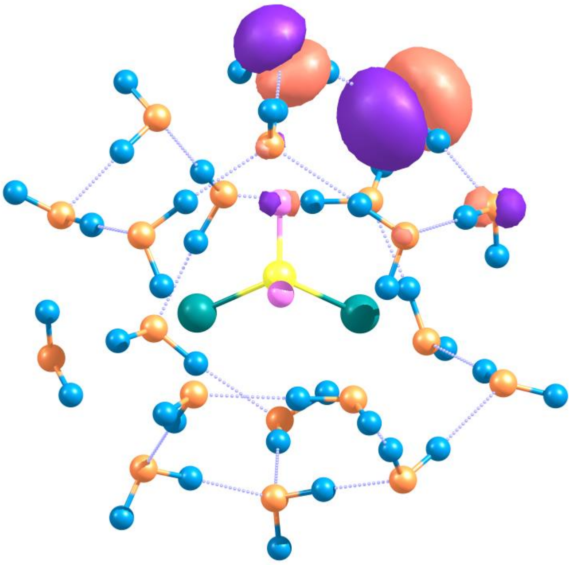

LUMO

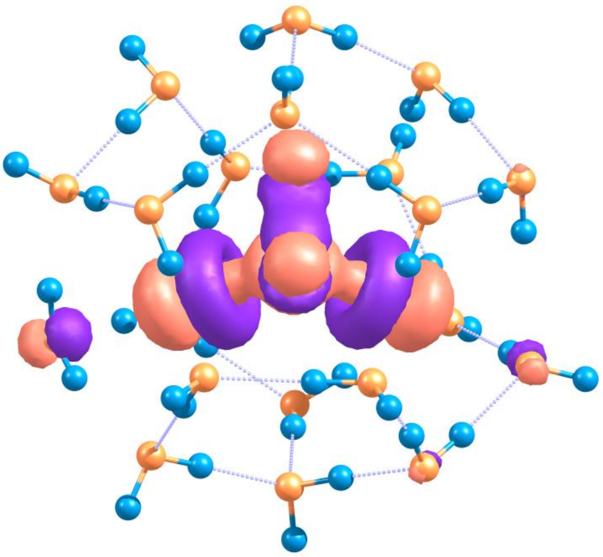

5^{12}

HOMO **LUMO**

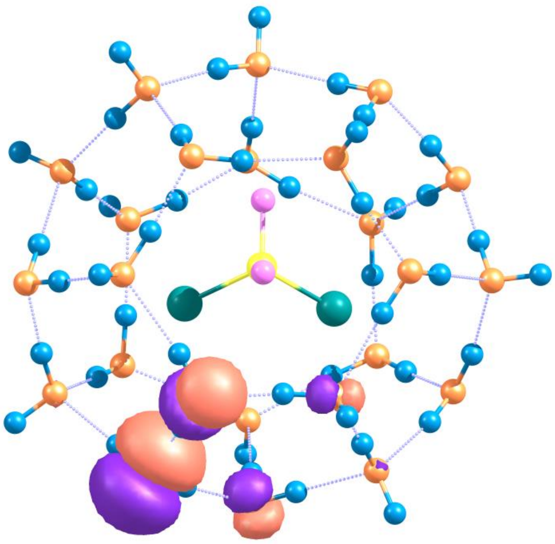

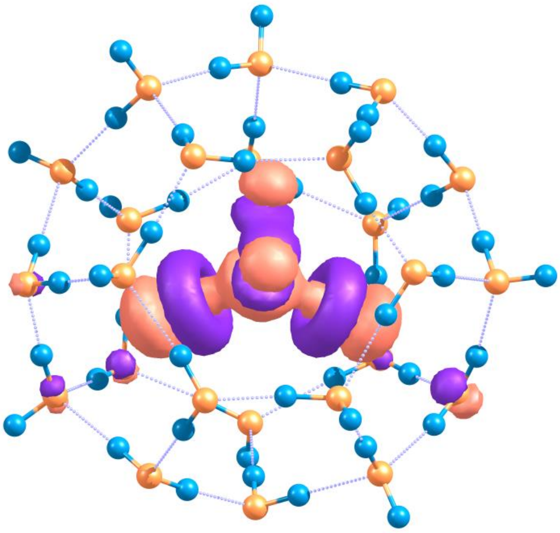

$5^{12}6^{2}$

HOMO **LUMO**

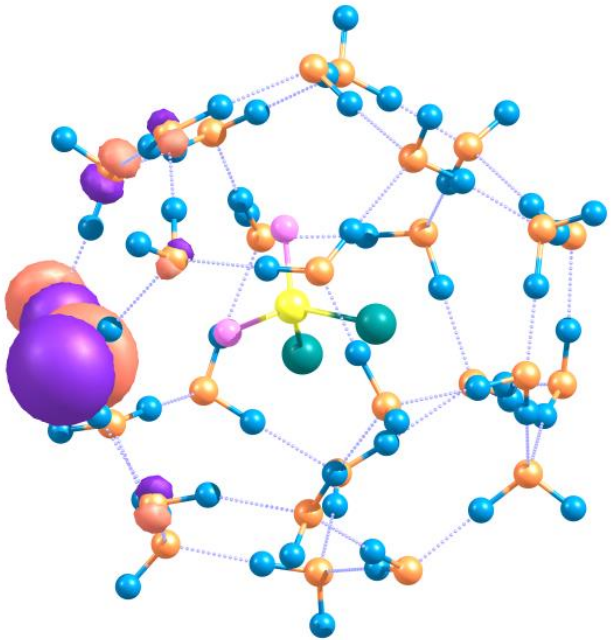

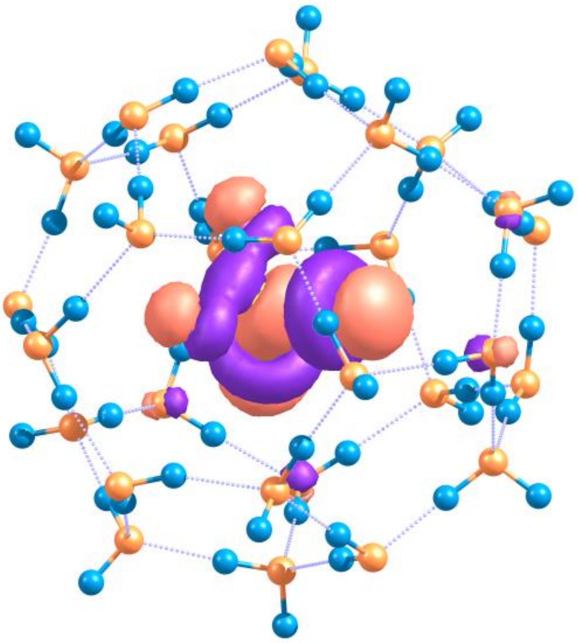

$5^{12}6^{4}$

Figure S21. Frontier molecular orbital of the CH_3Br molecule before and after encapsulation in 5^{12}, $5^{12}6^2$ and $5^{12}6^4$ clathrate hydrates computes at B3LYP/6-31G(d) level of theory and basis set.

Before Encapsulation

HOMO

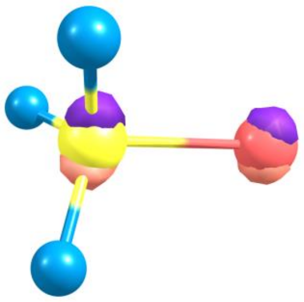

LUMO

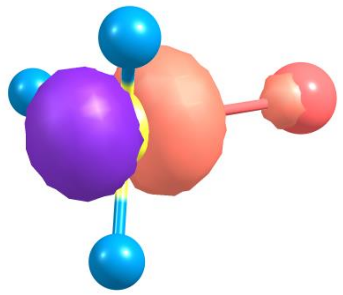

After Encapsulation

HOMO

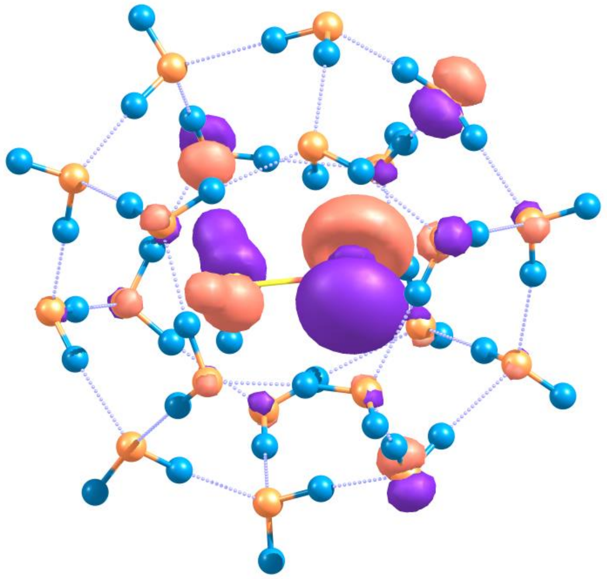

LUMO

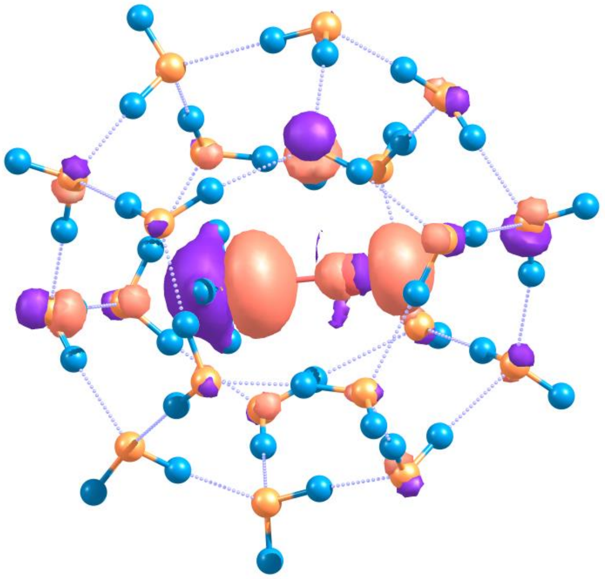

5^{12}

HOMO **LUMO**

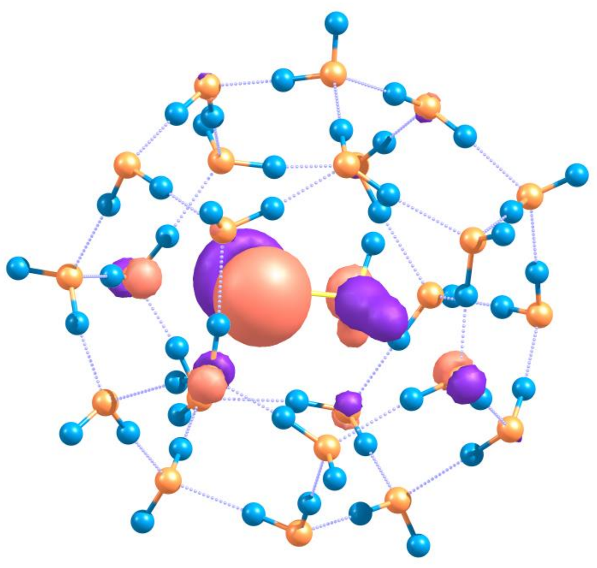

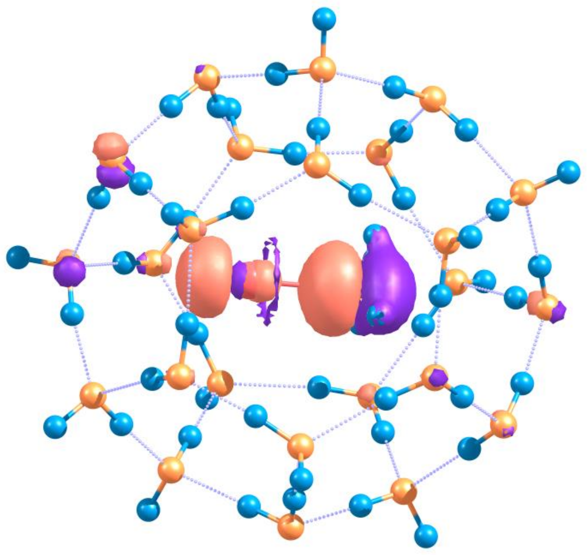

$5^{12}6^{2}$

HOMO **LUMO**

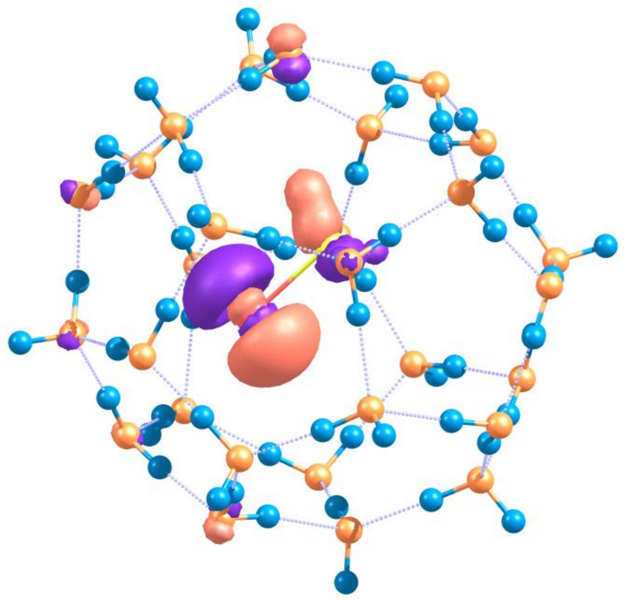

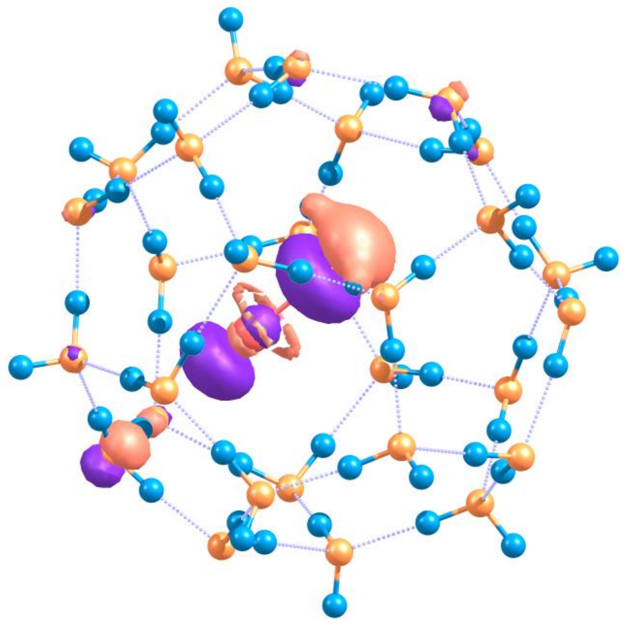

$5^{12}6^{4}$

Figure S22. Frontier molecular orbital of the CH_3Cl molecule before and after encapsulation in 5^{12}, $5^{12}6^2$ and $5^{12}6^4$ clathrate hydrates computes at B3LYP/6-31G(d) level of theory and basis set.

Before Encapsulation

HOMO

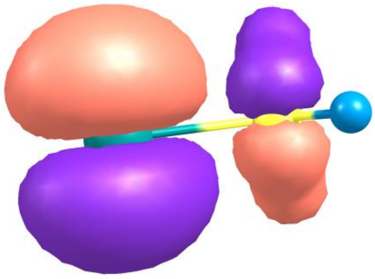

LUMO

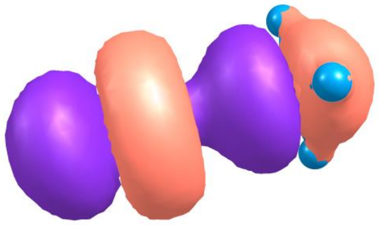

After Encapsulation

HOMO

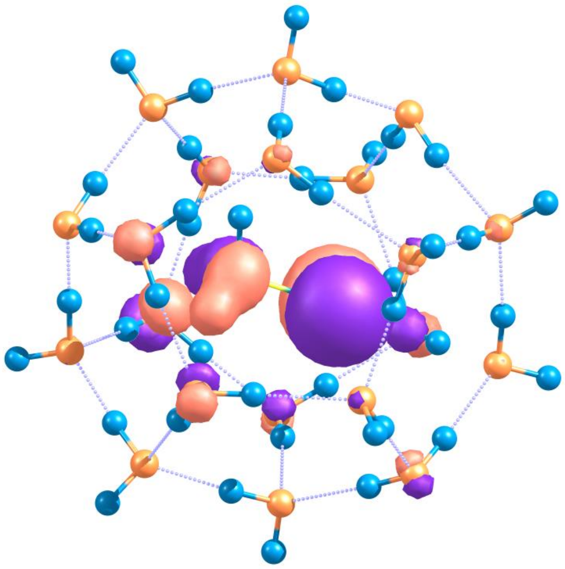

LUMO

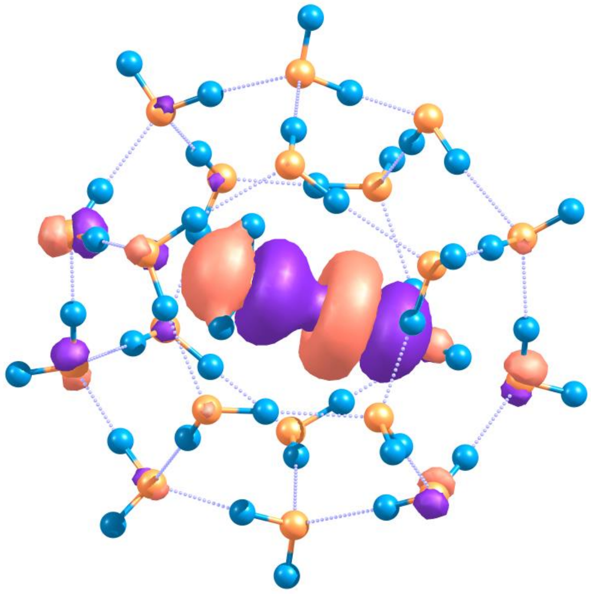

5^{12}

HOMO **LUMO**

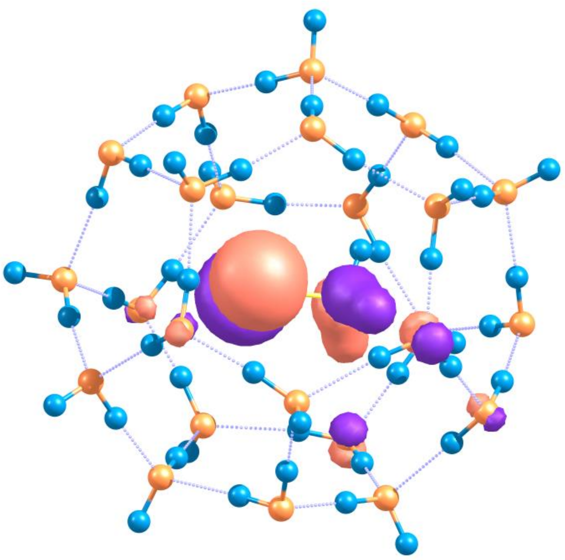

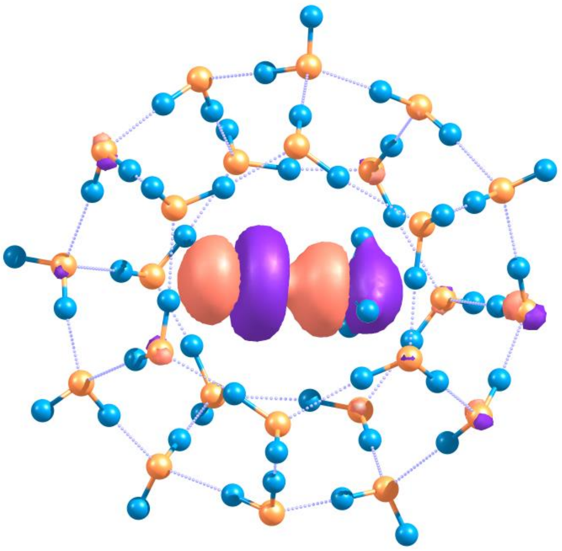

$5^{12}6^{2}$

HOMO **LUMO**

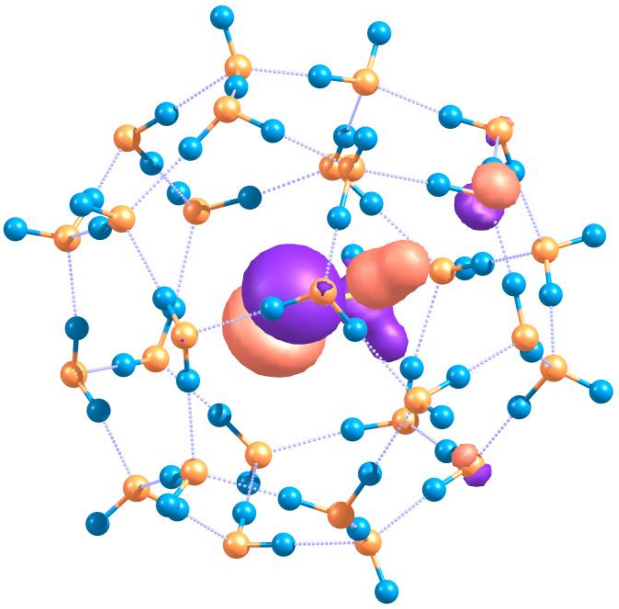

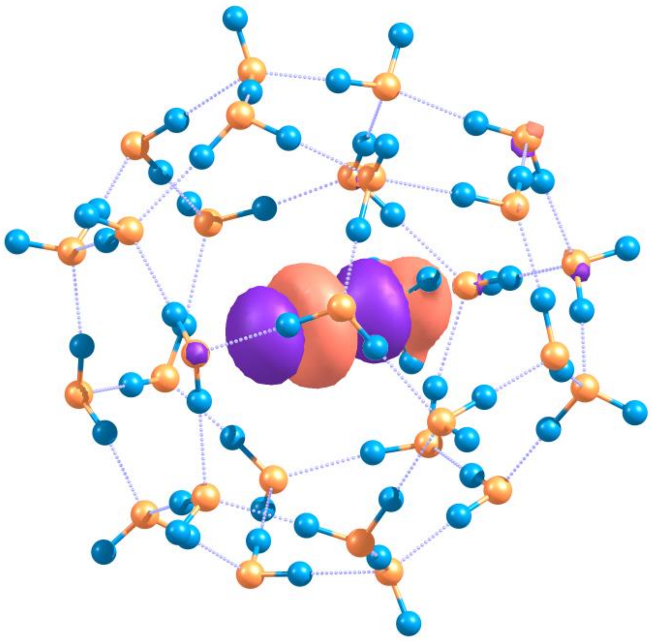

$5^{12}6^{4}$

Figure S23. Frontier molecular orbital of the CH_4 molecule before and after encapsulation in 5^{12}, $5^{12}6^2$ and $5^{12}6^4$ clathrate hydrates computes at B3LYP/6-31G(d) level of theory and basis set.

Before Encapsulation

HOMO

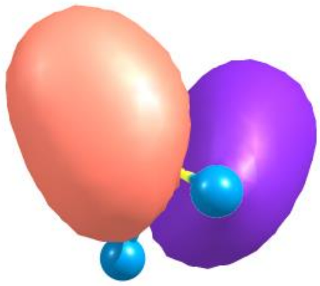

LUMO

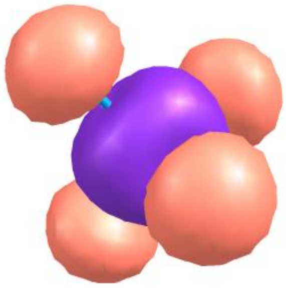

After Encapsulation

HOMO

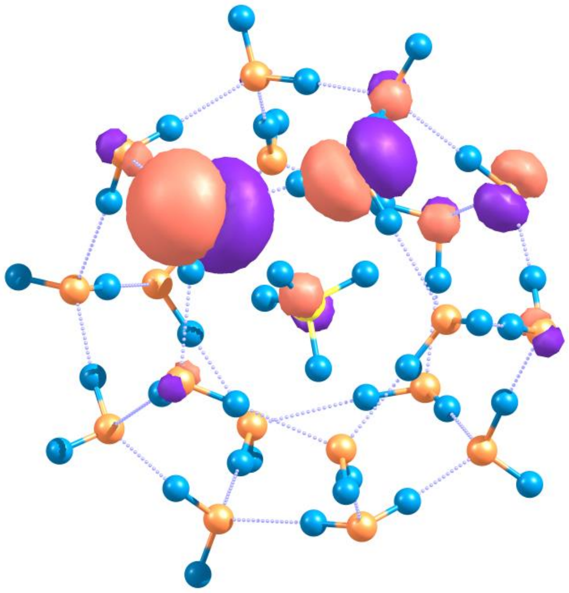

LUMO

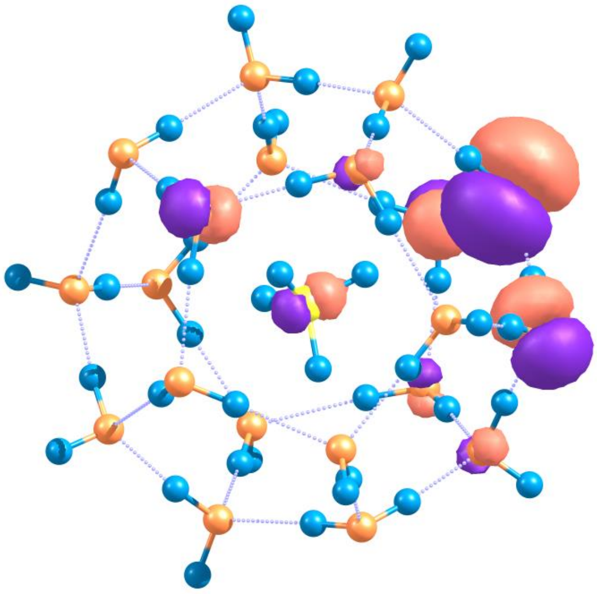

$\mathbf{5^{12}}$

HOMO | LUMO

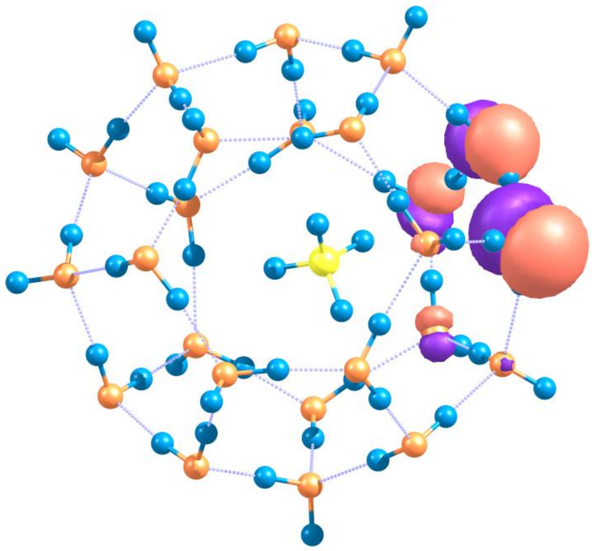

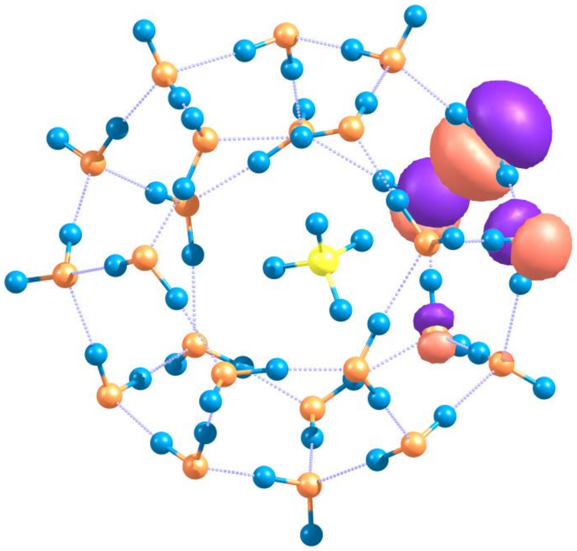

$5^{12}6^2$

HOMO | LUMO

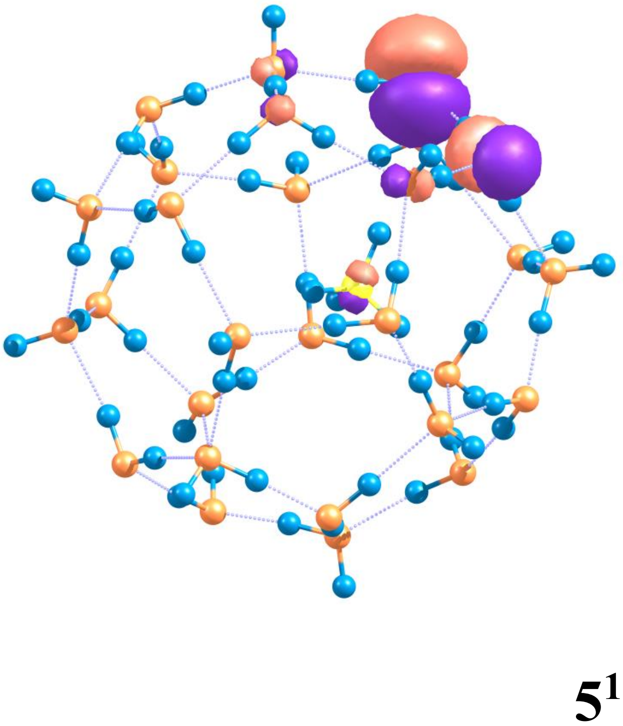

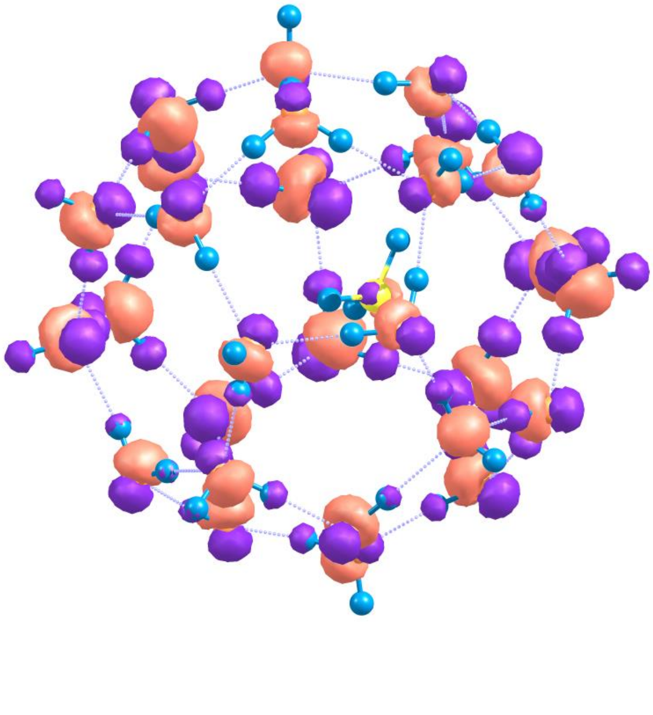

$5^{12}6^4$

Figure S24. Frontier molecular orbital of the CO_2 molecule before and after encapsulation in 5^{12}, $5^{12}6^2$ and $5^{12}6^4$ clathrate hydrates computes at B3LYP/6-31G(d) level of theory and basis set.

Before Encapsulation

HOMO

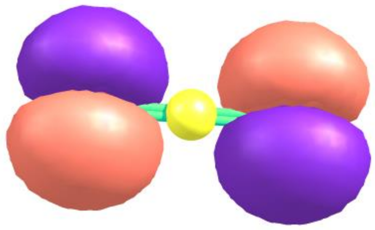

LUMO

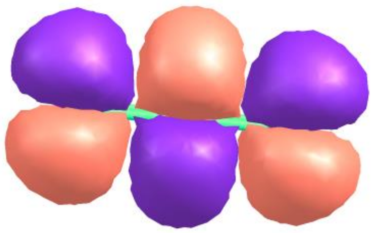

After Encapsulation

HOMO

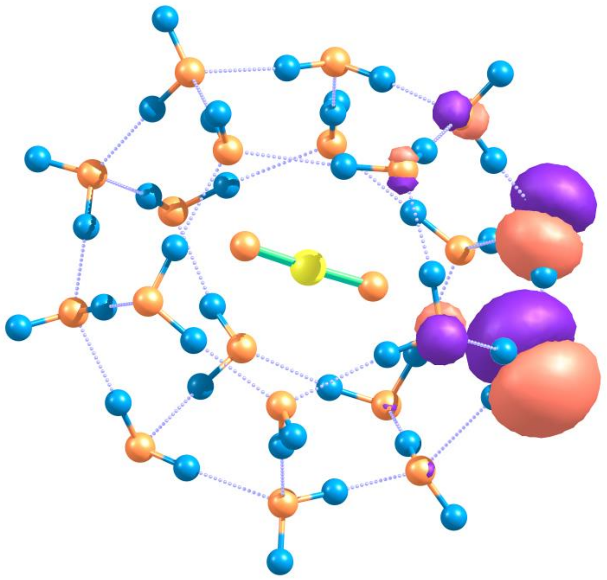

LUMO

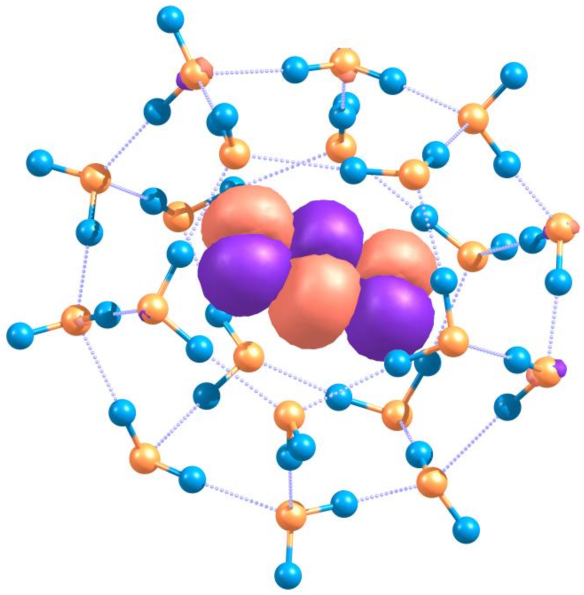

5^{12}

HOMO LUMO

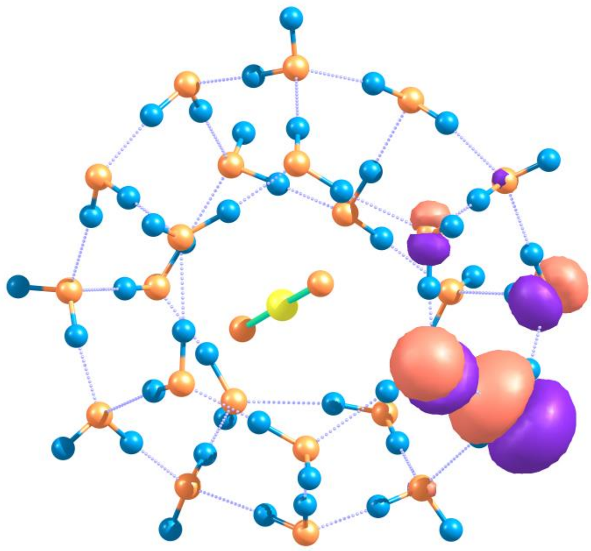

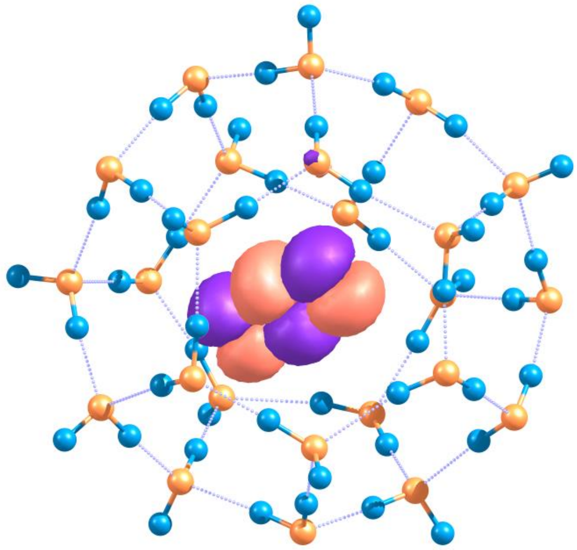

$5^{12}6^{2}$

HOMO LUMO

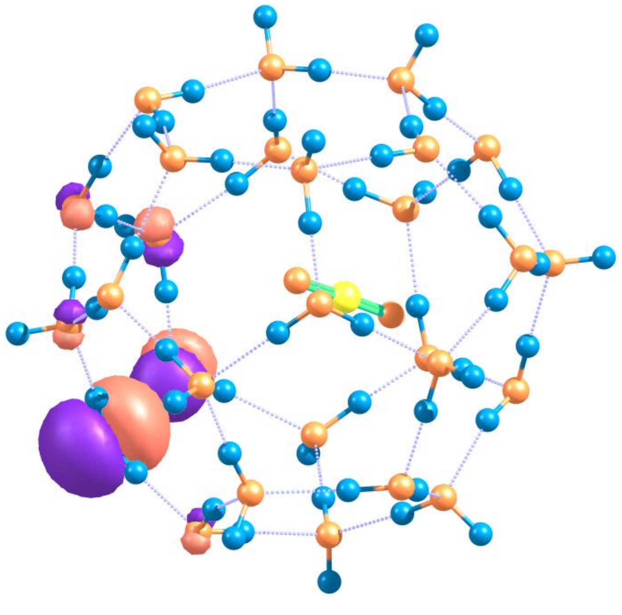

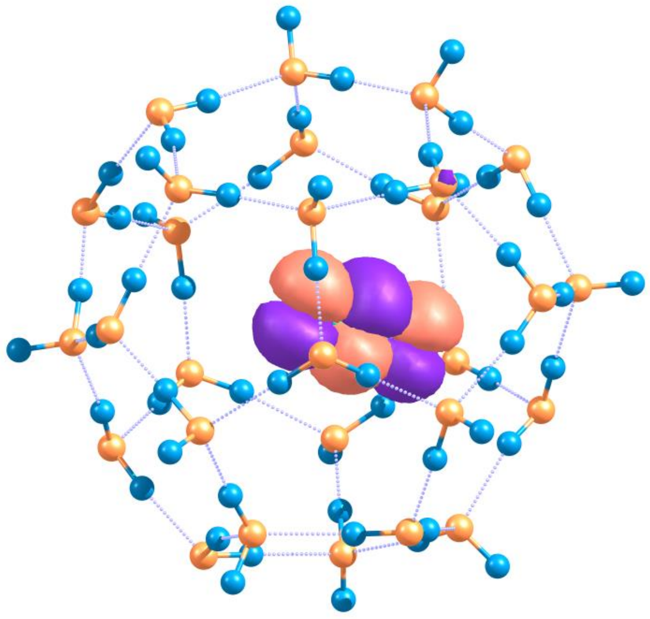

$5^{12}6^{4}$

Figure S25. Frontier molecular orbital of the CO molecule before and after encapsulation in 5^{12}, $5^{12}6^{2}$ and $5^{12}6^{4}$ clathrate hydrates computes at B3LYP/6-31G(d) level of theory and basis set.

Before Encapsulation

HOMO

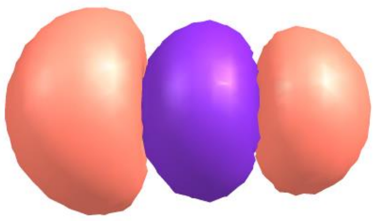

LUMO

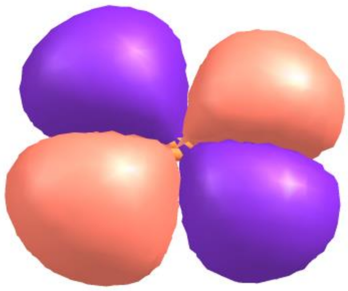

After Encapsulation

HOMO

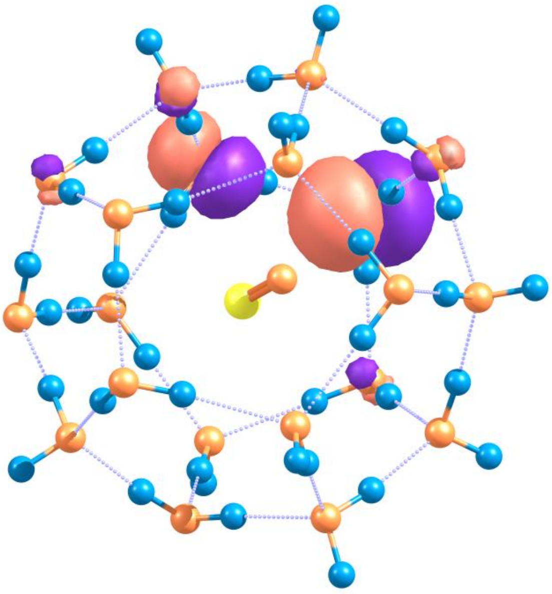

LUMO

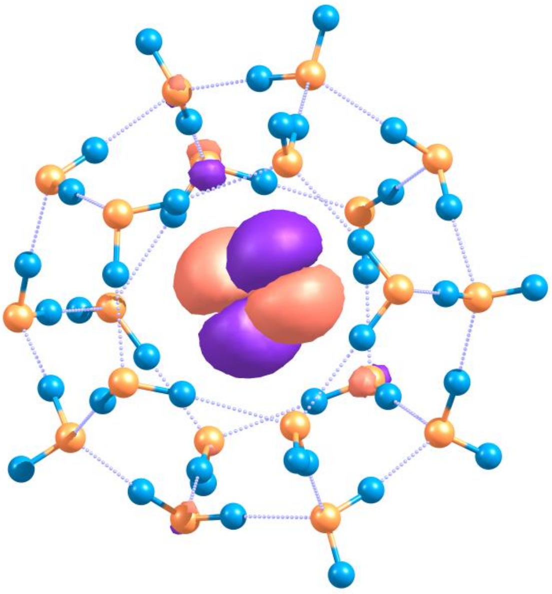

5^{12}

HOMO **LUMO**

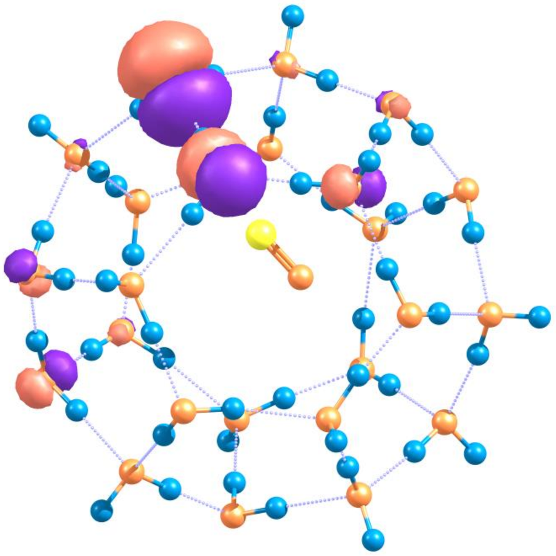

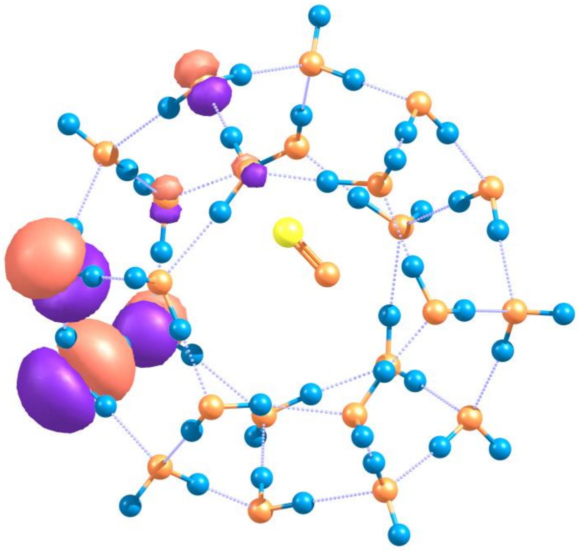

$5^{12}6^{2}$

HOMO **LUMO**

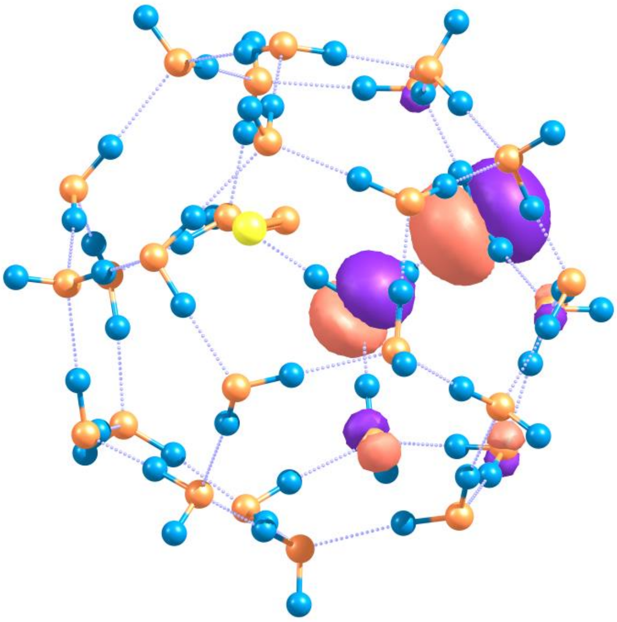

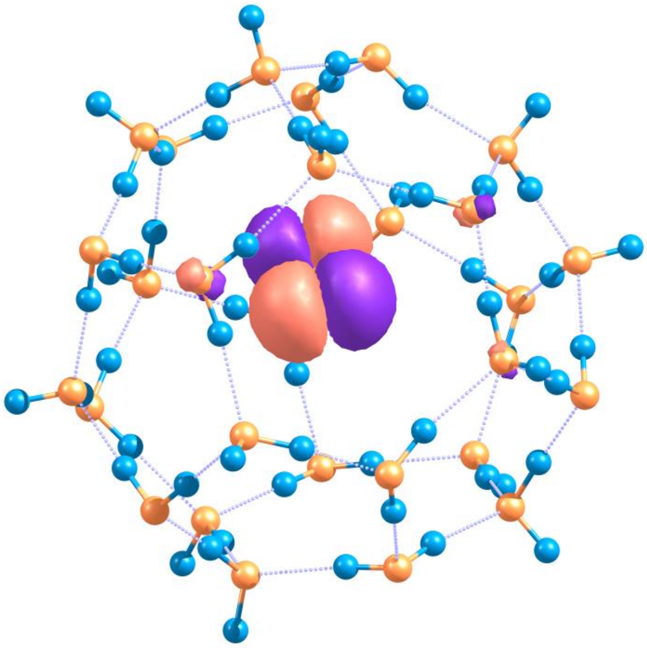

$5^{12}6^{4}$

Figure S26. Frontier molecular orbital of the H_2S molecule before and after encapsulation in 5^{12}, $5^{12}6^{2}$ and $5^{12}6^{4}$ clathrate hydrates computes at B3LYP/6-31G(d) level of theory and basis set.

Before Encapsulation

HOMO

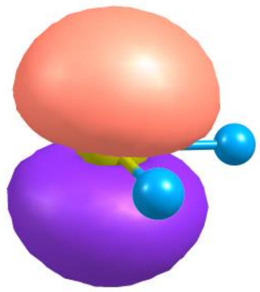

LUMO

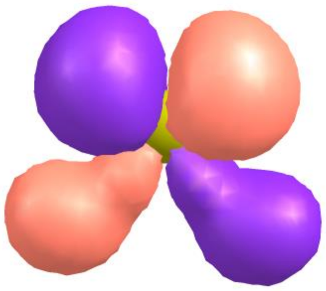

After Encapsulation

HOMO

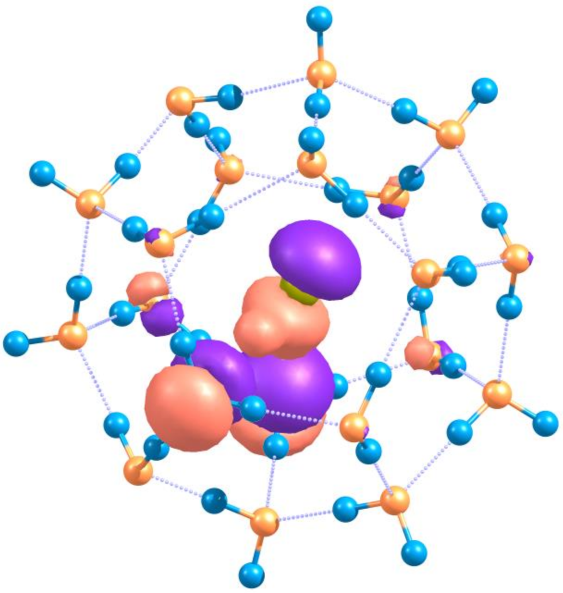

LUMO

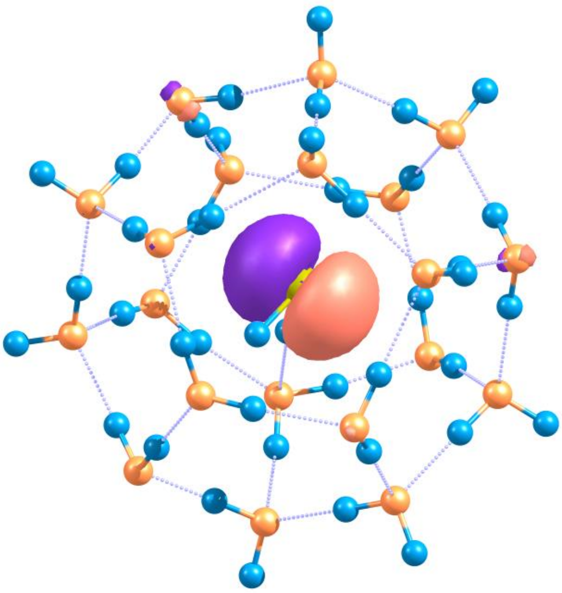

5^{12}

HOMO **LUMO**

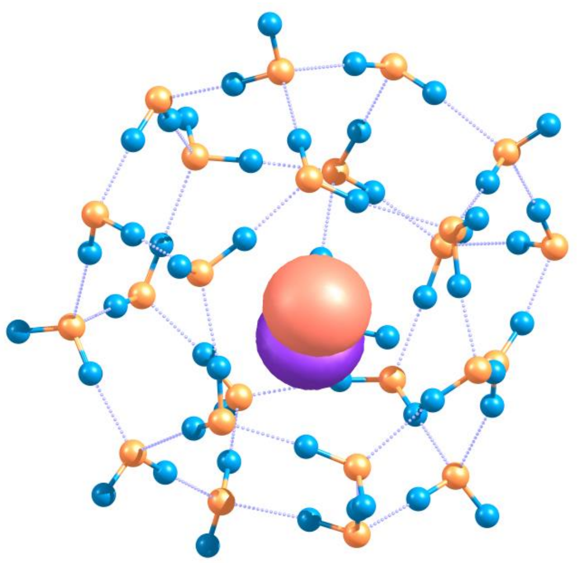

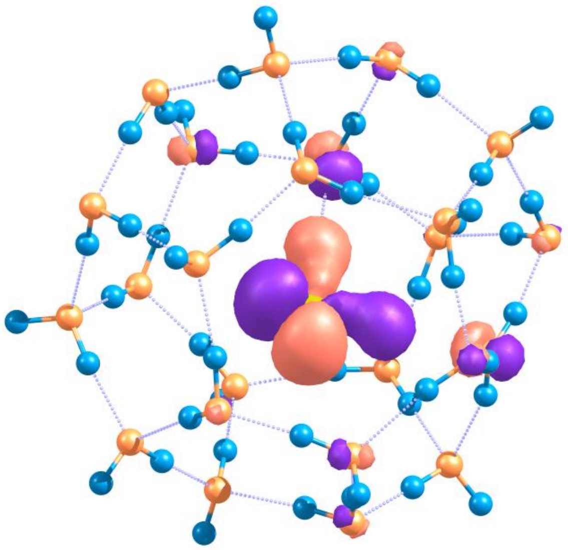

$5^{12}6^{2}$

HOMO **LUMO**

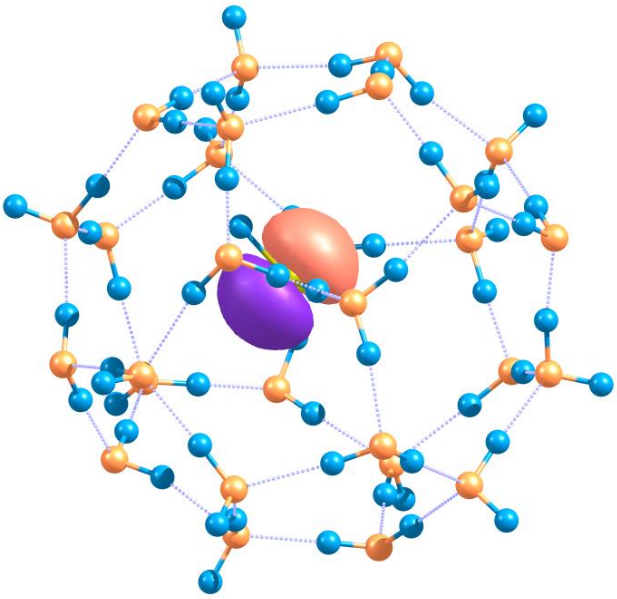

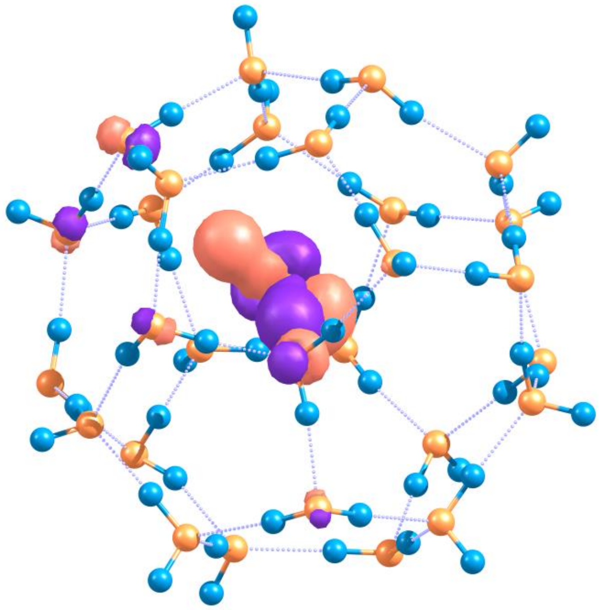

$5^{12}6^{4}$

Figure S27. Frontier molecular orbital of the CH_3F molecule before and after encapsulation in 5^{12}, $5^{12}6^2$ and $5^{12}6^4$ clathrate hydrates computes at B3LYP/6-31G(d) level of theory and basis set.

Before Encapsulation

HOMO

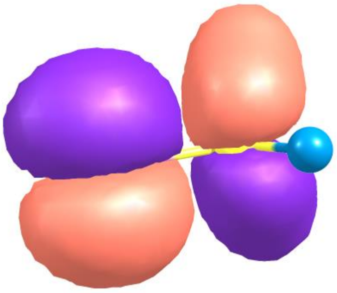

LUMO

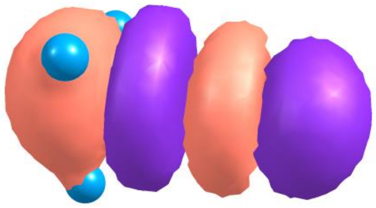

After Encapsulation

HOMO

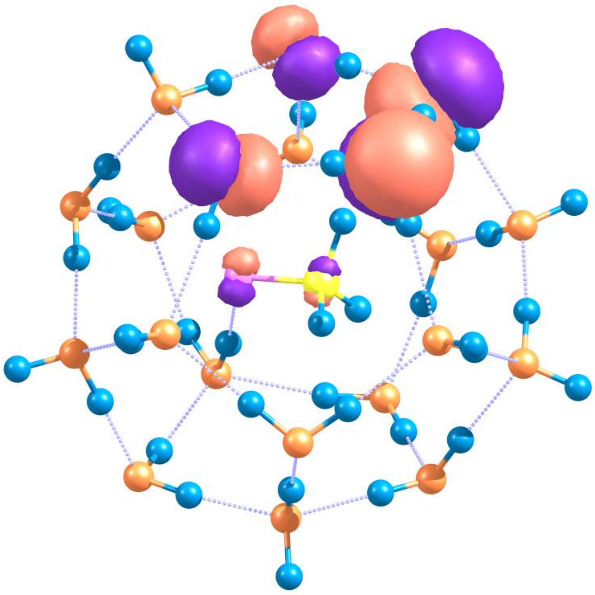

LUMO

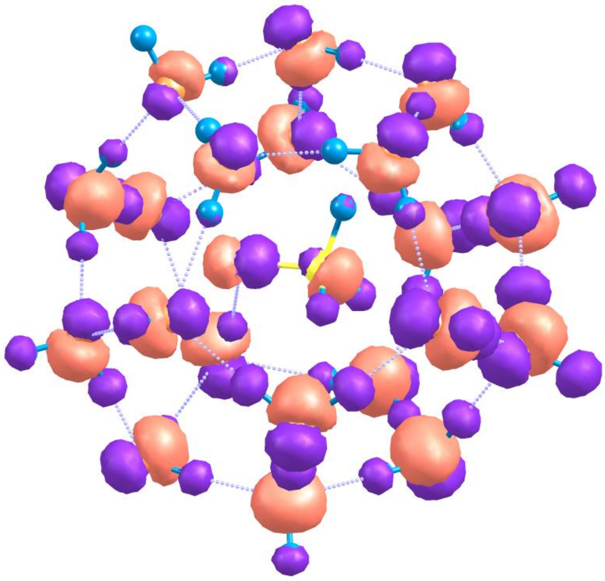

5^{12}

HOMO LUMO

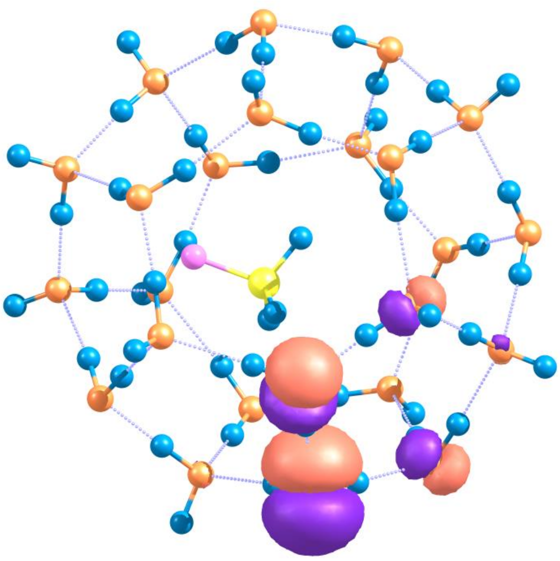

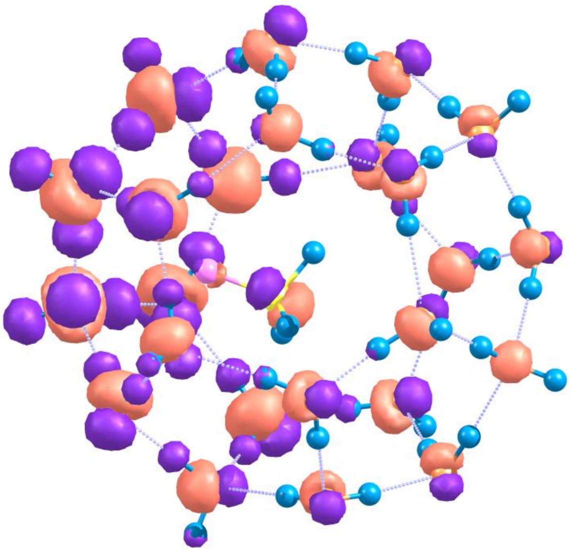

$5^{12}6^{2}$

HOMO LUMO

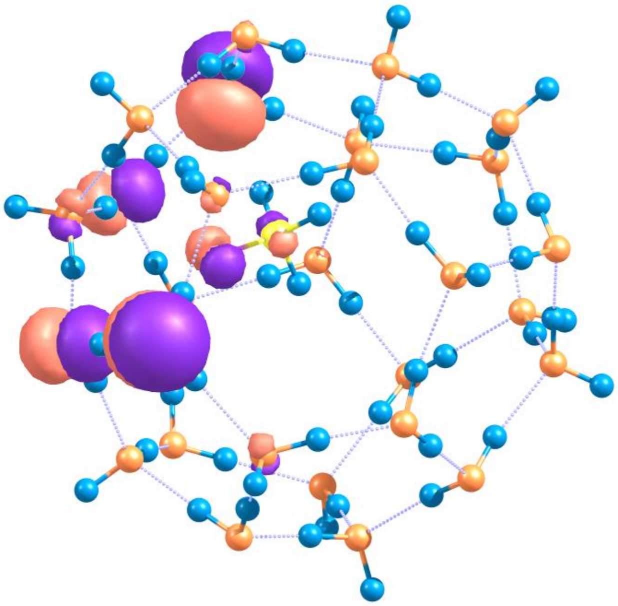

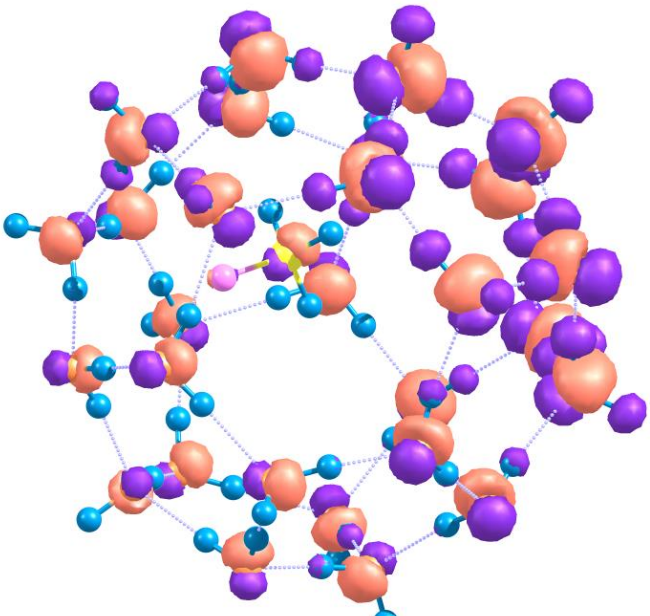

$5^{12}6^{4}$

Figure S28. Frontier molecular orbital of the N_2O molecule before and after encapsulation in 5^{12}, $5^{12}6^2$ and $5^{12}6^4$ clathrate hydrates computes at B3LYP/6-31G(d) level of theory and basis set.

Before Encapsulation

HOMO

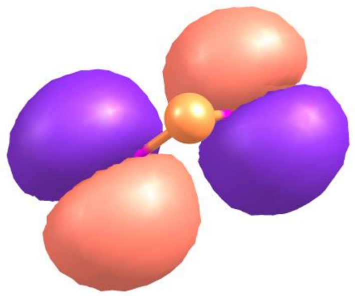

LUMO

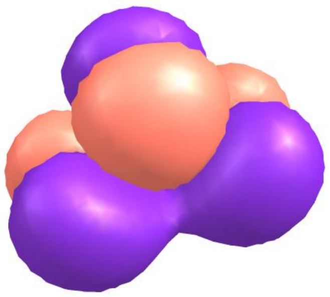

After Encapsulation

HOMO

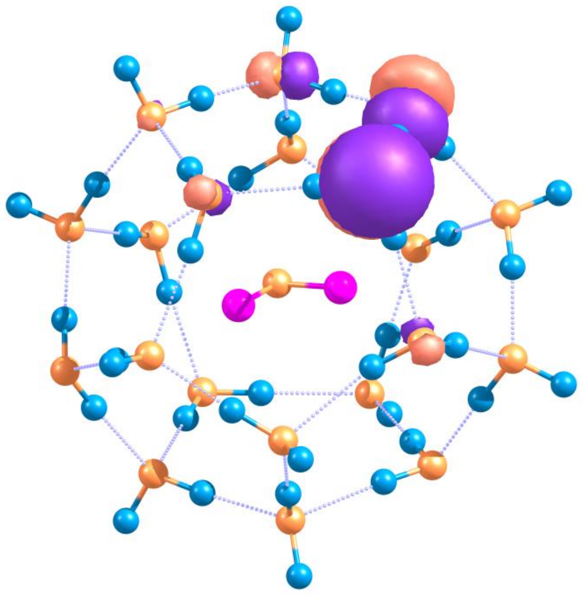

LUMO

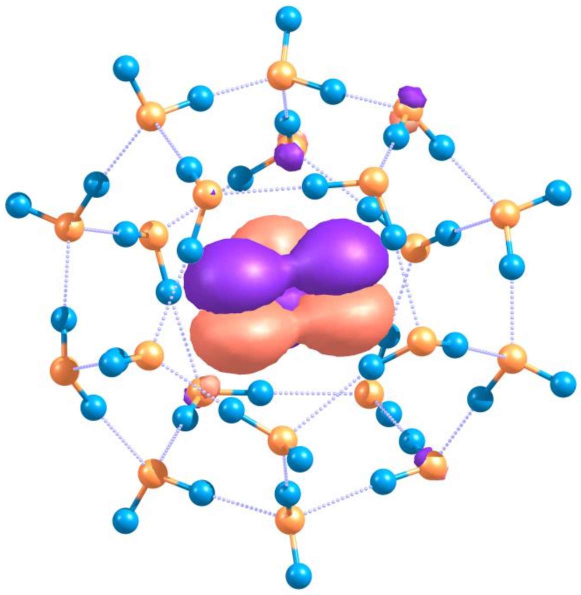

5^{12}

HOMO LUMO

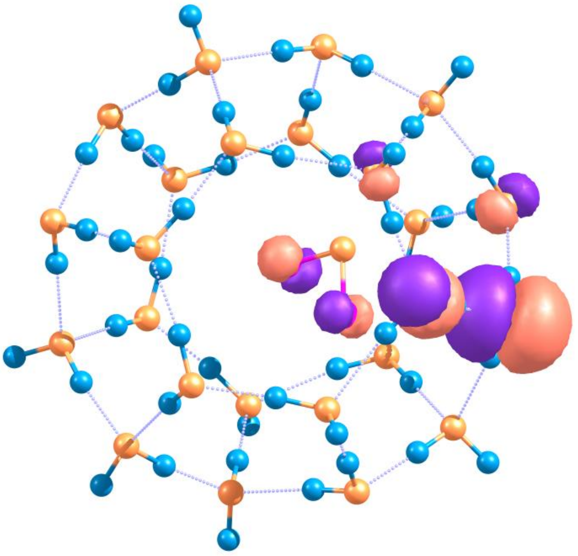

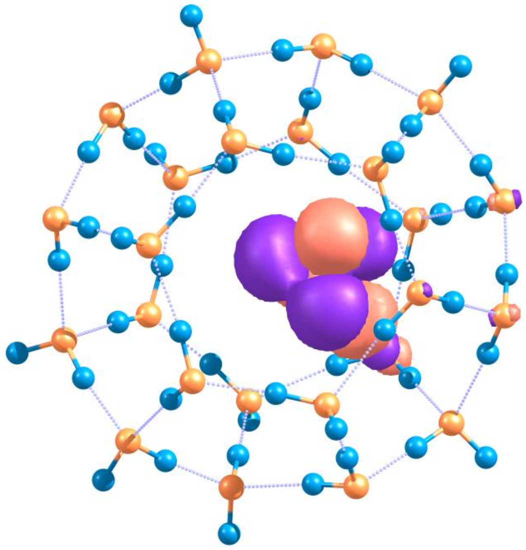

$5^{12}6^{2}$

HOMO LUMO

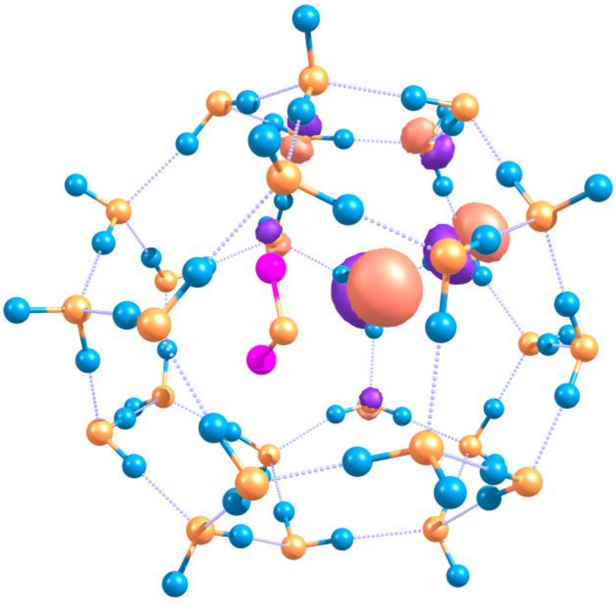

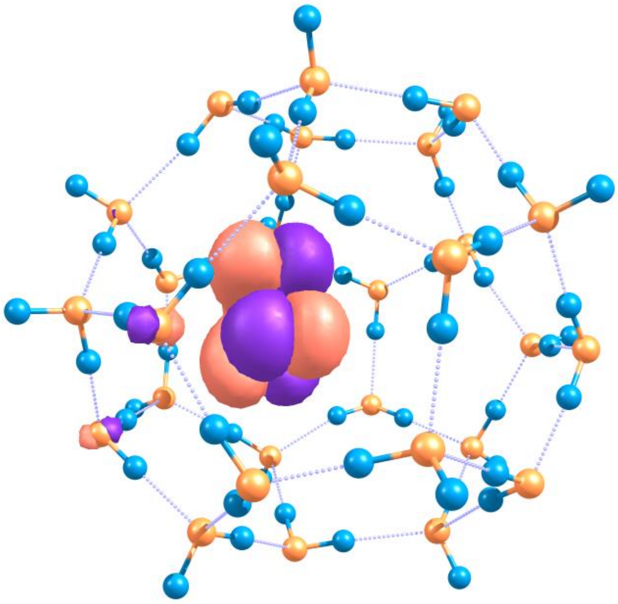

$5^{12}6^{4}$

Figure S29. Frontier molecular orbital of the NF_3 molecule before and after encapsulation in 5^{12}, $5^{12}6^2$ and $5^{12}6^4$ clathrate hydrates computes at B3LYP/6-31G(d) level of theory and basis set.

Before Encapsulation

HOMO

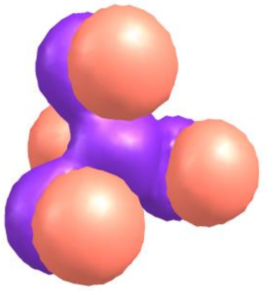

LUMO

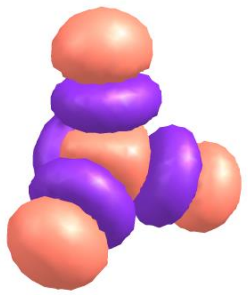

After Encapsulation

HOMO

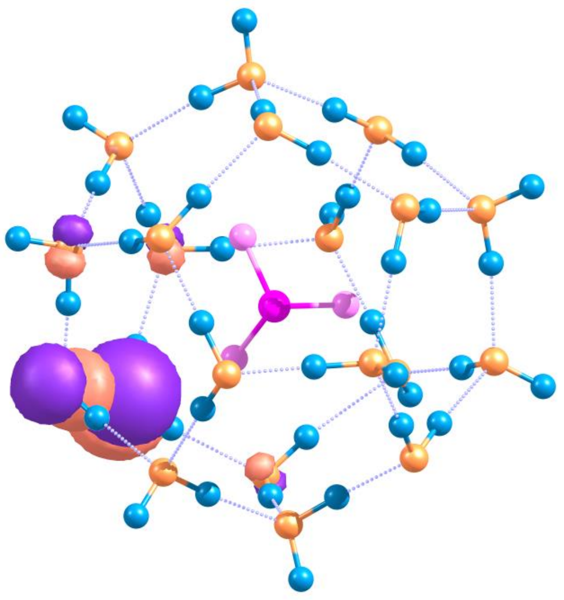

LUMO

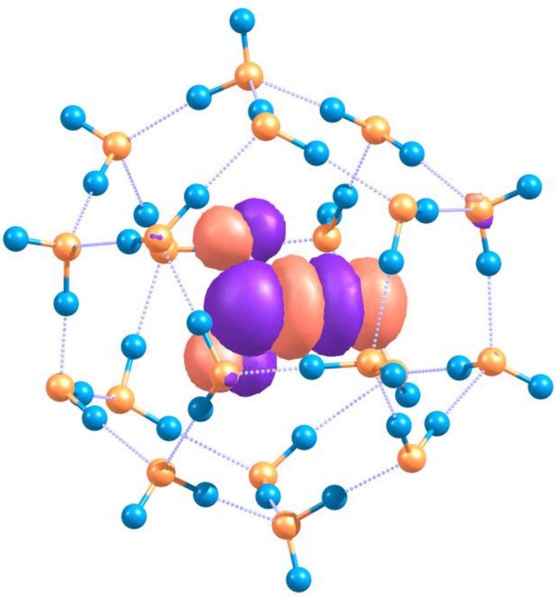

5^{12}

HOMO **LUMO**

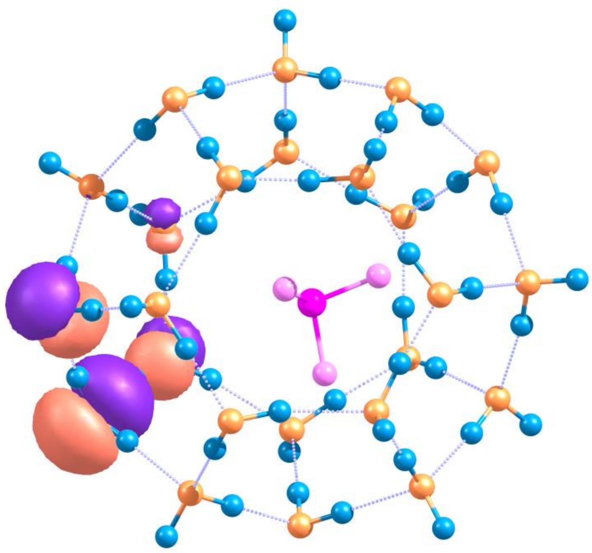

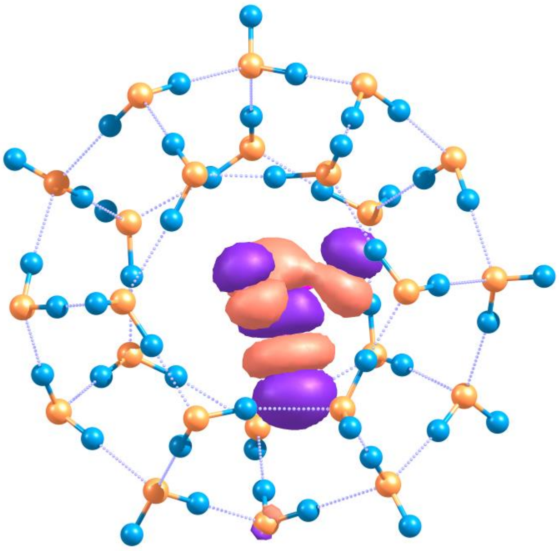

$5^{12}6^{2}$

HOMO **LUMO**

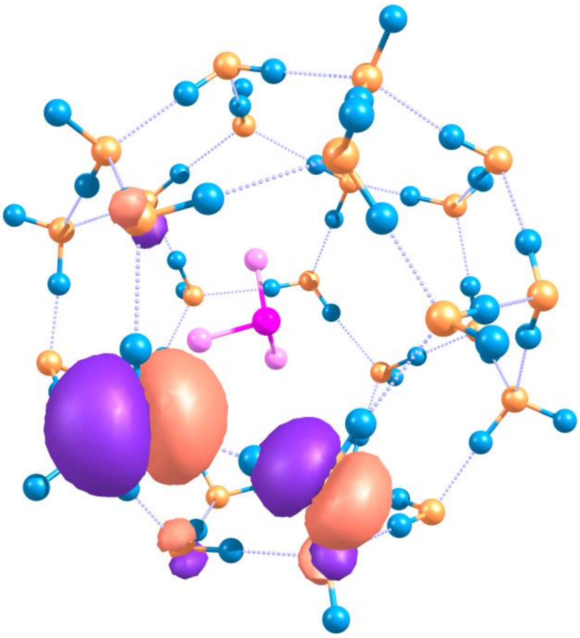

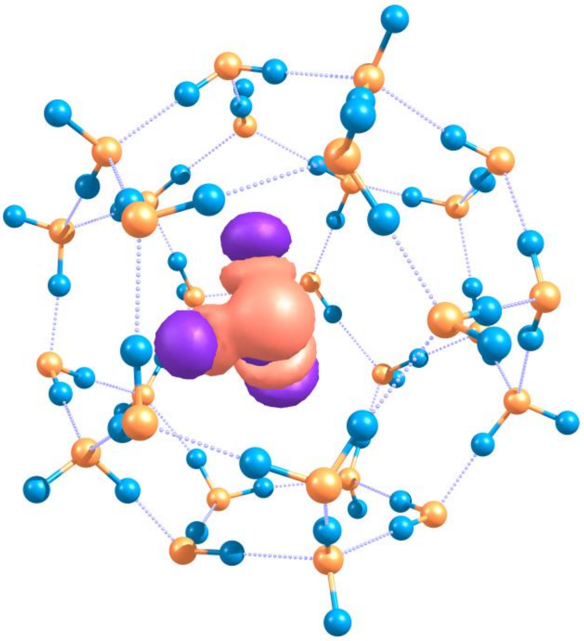

$5^{12}6^{4}$

Figure S30. Frontier molecular orbital of the O_3 molecule before and after encapsulation in 5^{12}, $5^{12}6^2$ and $5^{12}6^4$ clathrate hydrates computes at B3LYP/6-31G(d) level of theory and basis set.

Before Encapsulation

HOMO **LUMO**

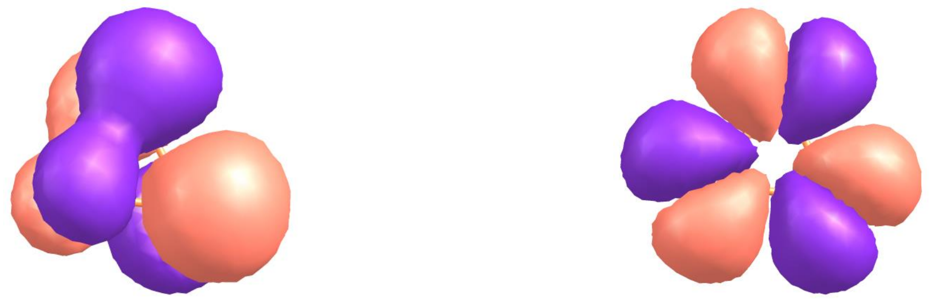

After Encapsulation

HOMO **LUMO**

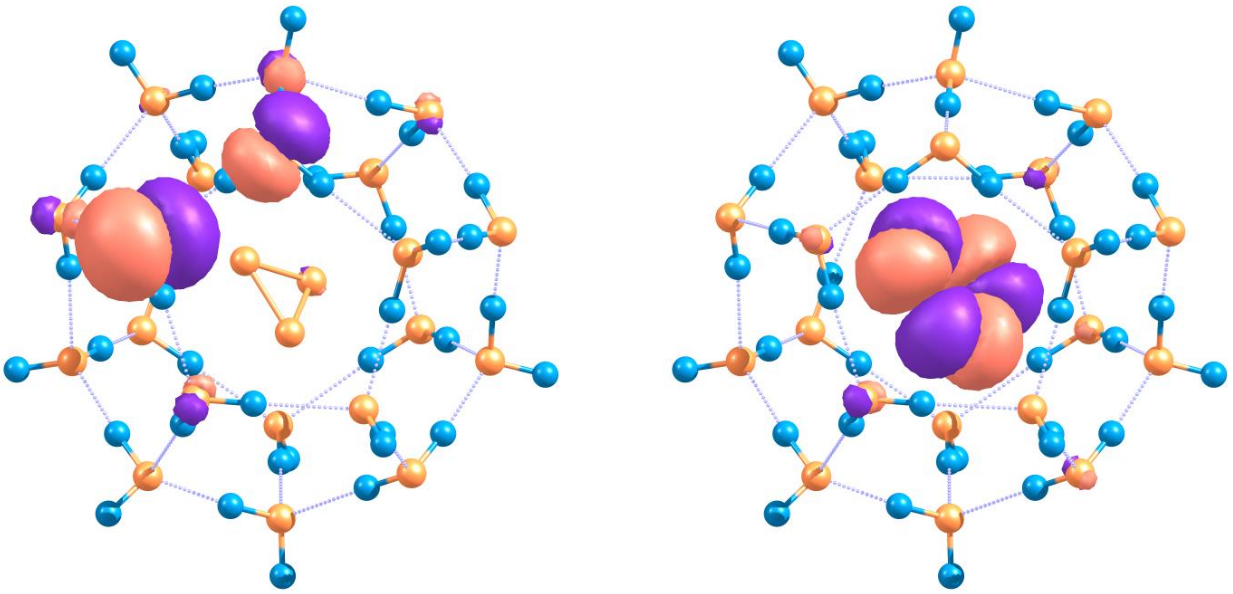

5^{12}

HOMO

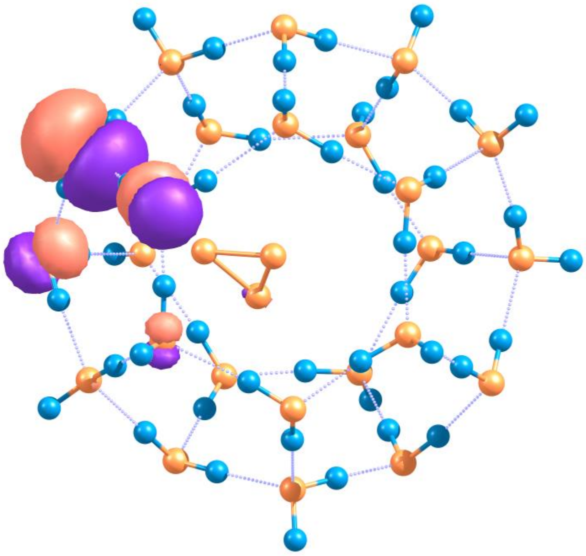

LUMO

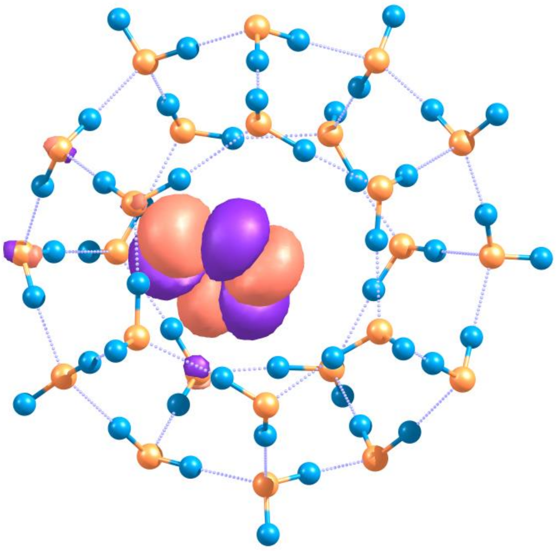

$5^{12}6^{2}$

HOMO

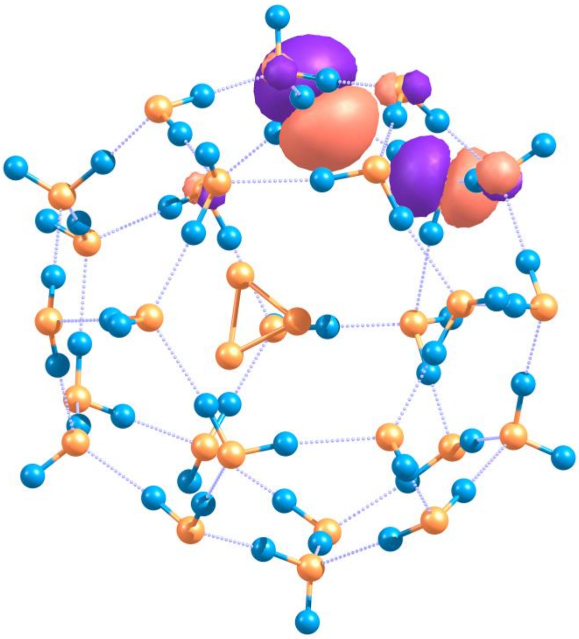

LUMO

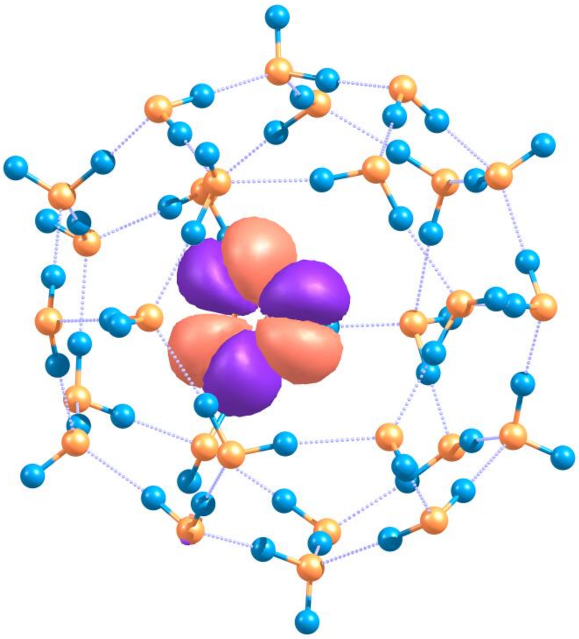

$5^{12}6^{4}$

Figure S31. Frontier molecular orbital of the CF_4 molecule before and after encapsulation in 5^{12}, $5^{12}6^{2}$ and $5^{12}6^{4}$ clathrate hydrates computes at B3LYP/6-31G(d) level of theory and basis set.

Before Encapsulation

HOMO **LUMO**

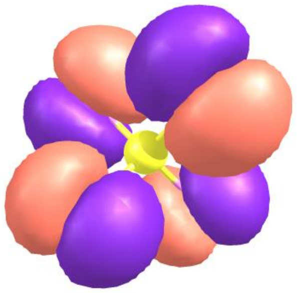

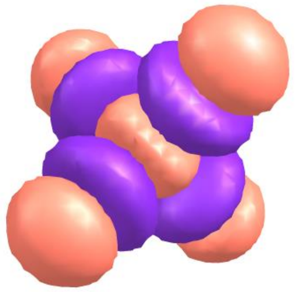

After Encapsulation

HOMO **LUMO**

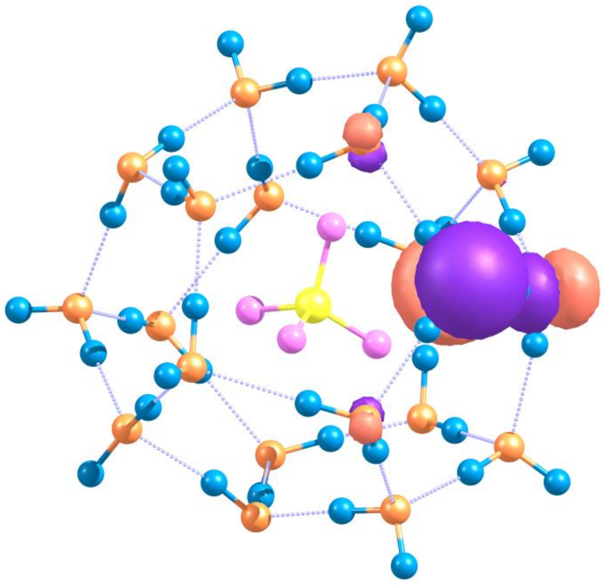

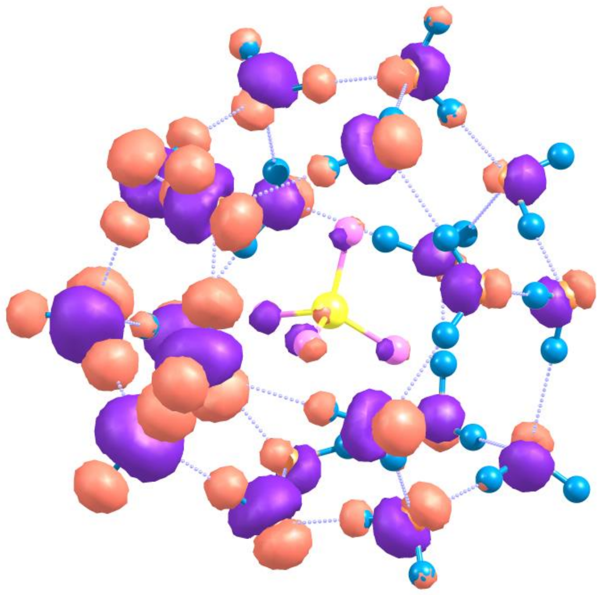

$\mathbf{5^{12}}$

HOMO **LUMO**

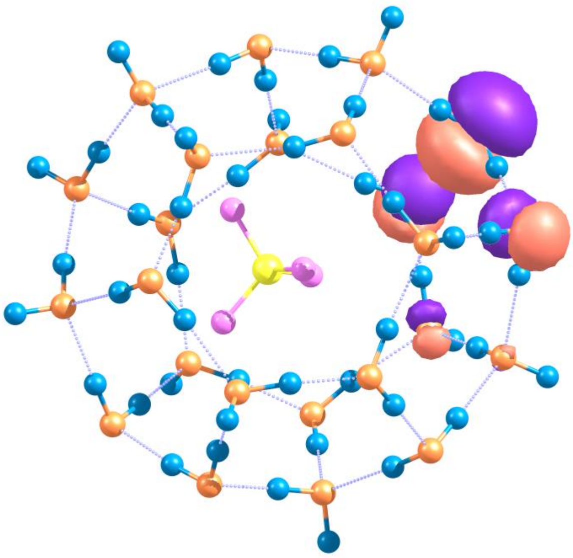

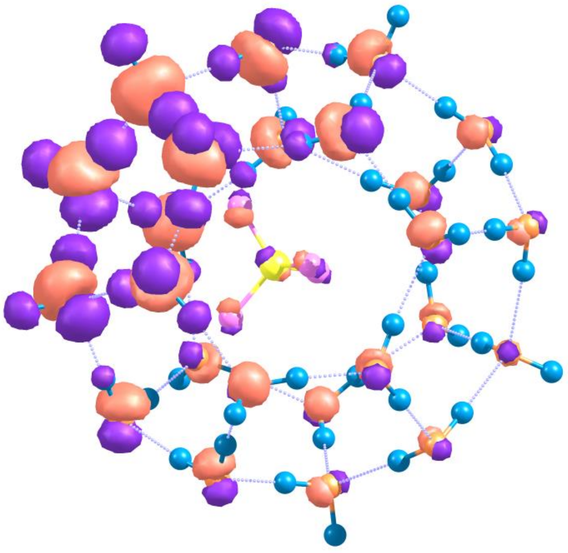

$5^{12}6^{2}$

HOMO **LUMO**

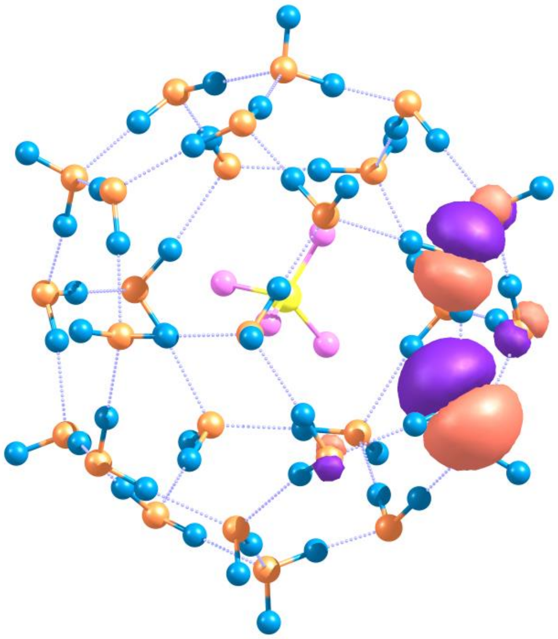

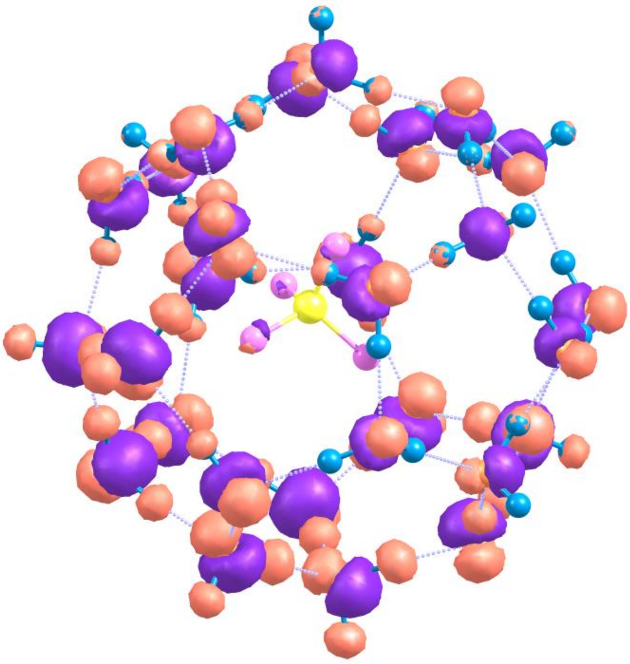

$5^{12}6^{4}$

Figure S32. Frontier molecular orbital of the SF_6 molecule before and after encapsulation in 5^{12}, $5^{12}6^2$ and $5^{12}6^4$ clathrate hydrates computes at B3LYP/6-31G(d) level of theory and basis set.

Before Encapsulation

HOMO **LUMO**

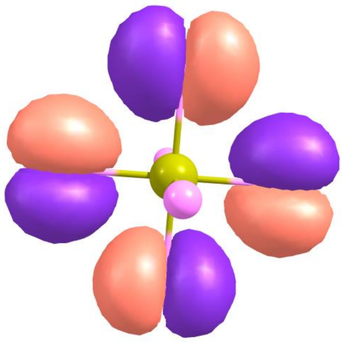

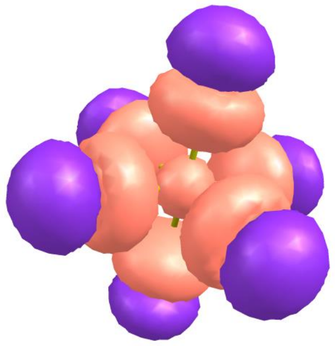

After Encapsulation

HOMO **LUMO**

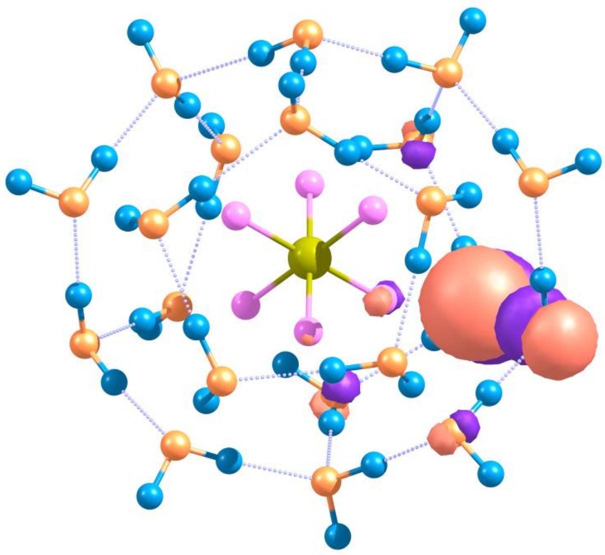

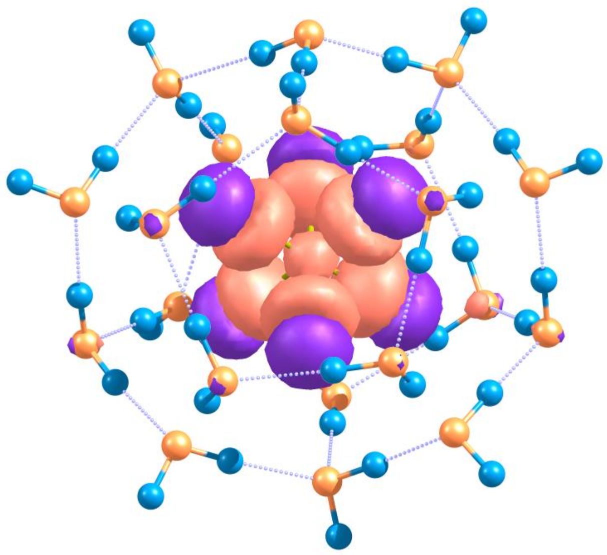

$\mathbf{5^{12}}$

HOMO **LUMO**

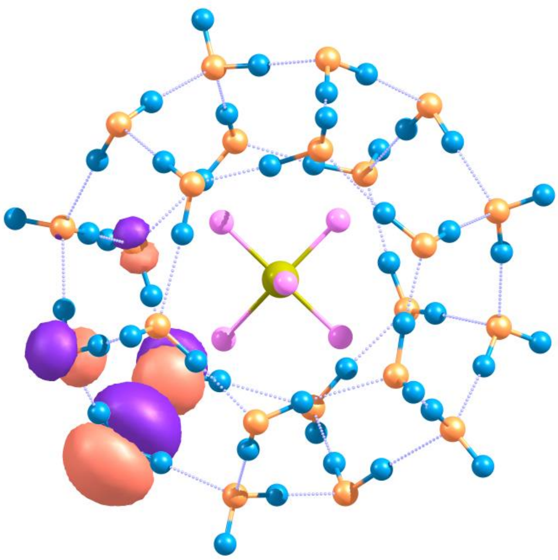

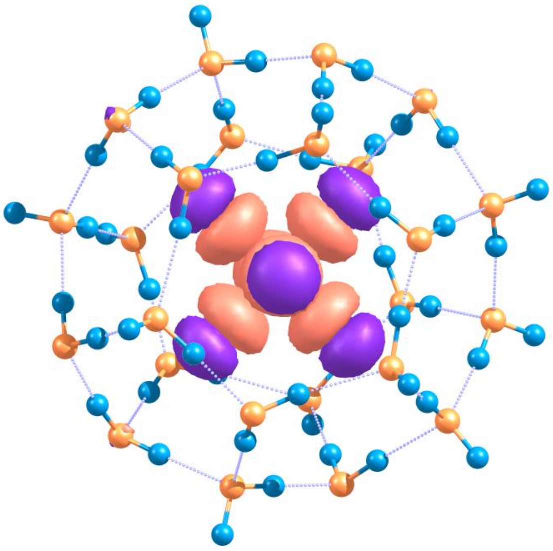

$5^{12}6^{2}$

HOMO **LUMO**

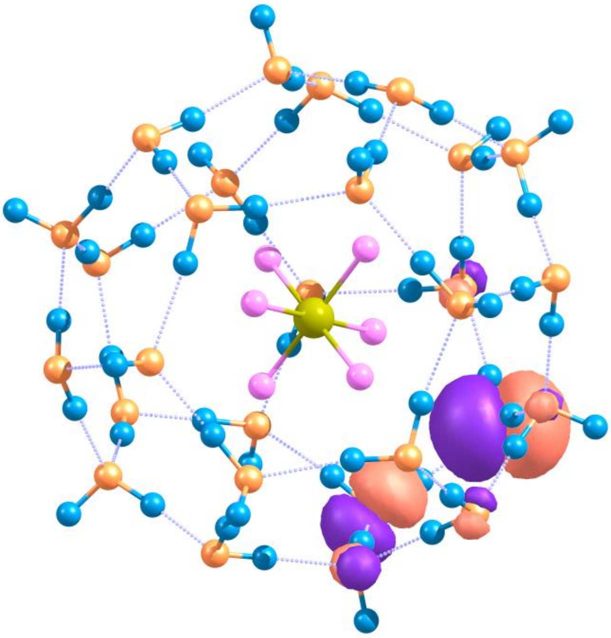

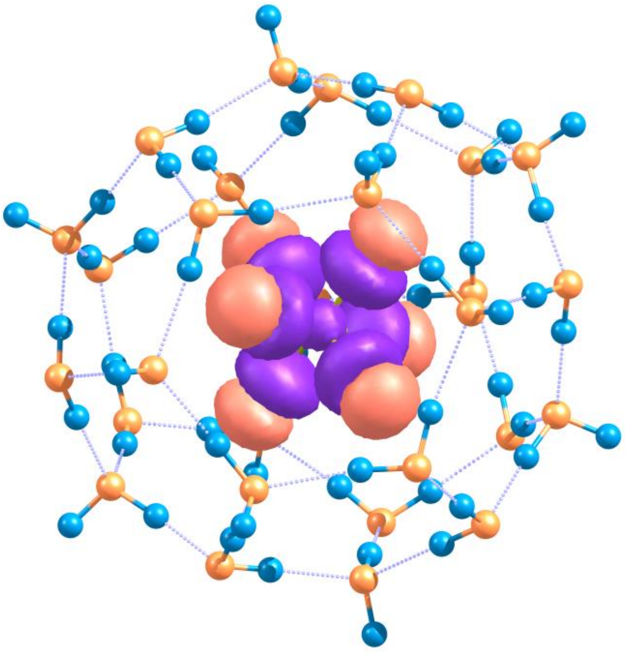

$5^{12}6^{4}$

Figure S33. Frontier molecular orbital of the SO_2 molecule before and after encapsulation in 5^{12}, $5^{12}6^2$ and $5^{12}6^4$ clathrate hydrates computes at B3LYP/6-31G(d) level of theory and basis set.

Before Encapsulation

HOMO

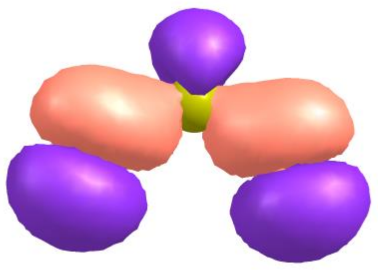

LUMO

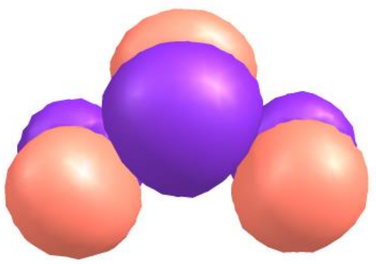

After Encapsulation

HOMO

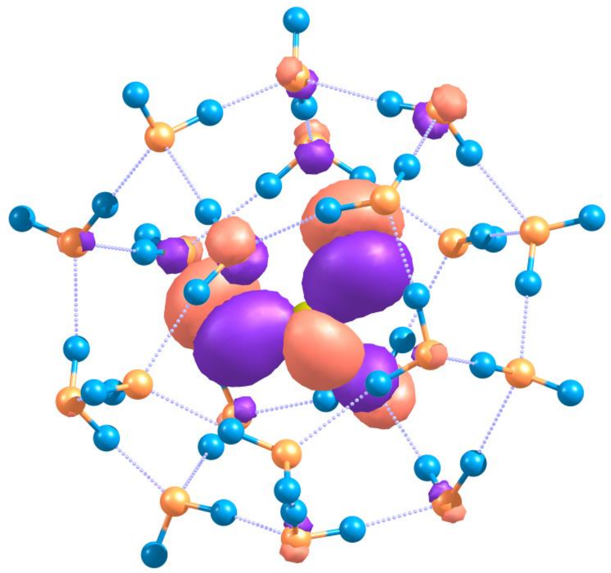

LUMO

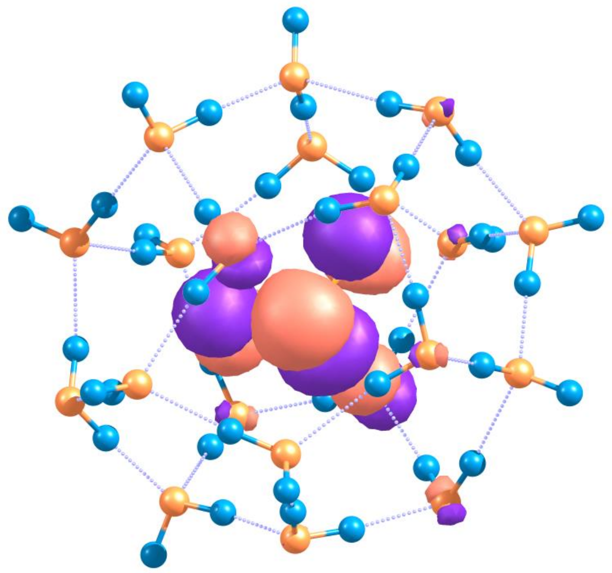

5^{12}

HOMO **LUMO**

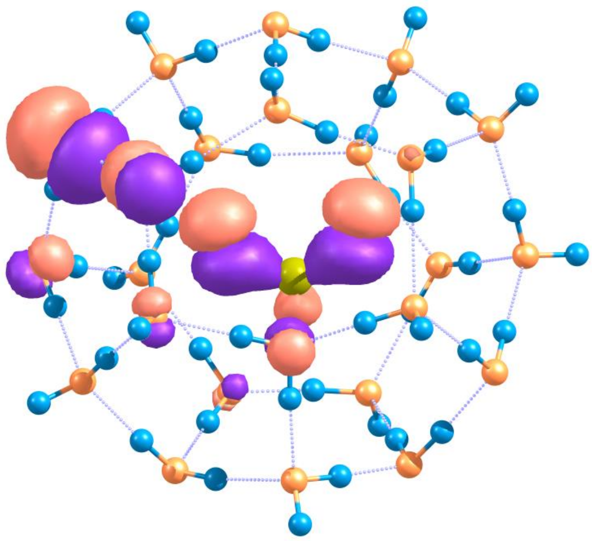

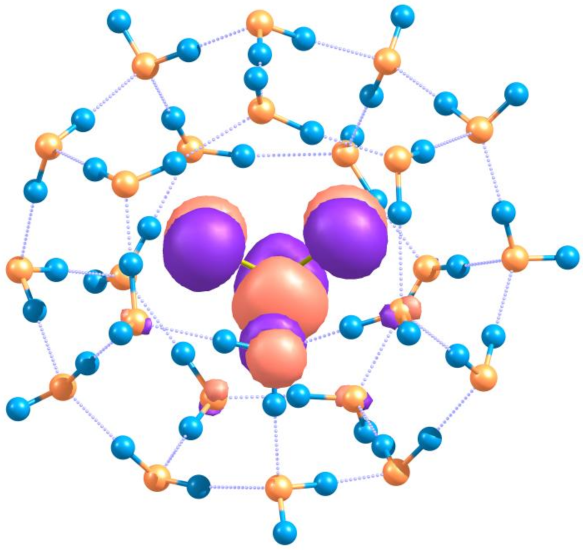

$5^{12}6^{2}$

HOMO **LUMO**

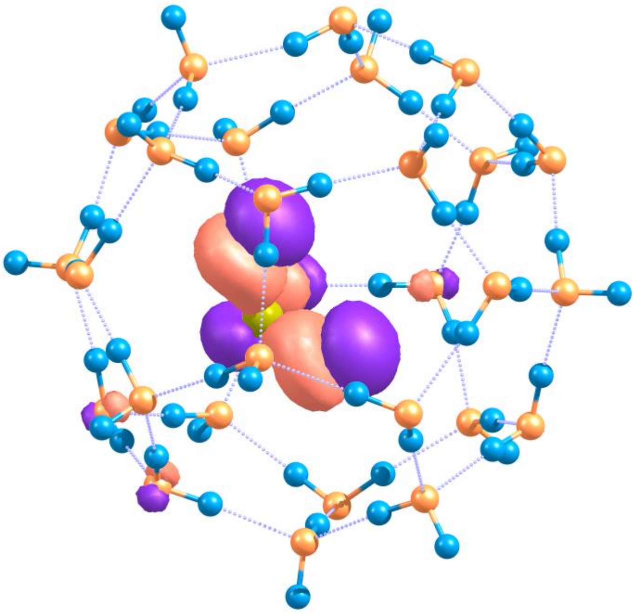

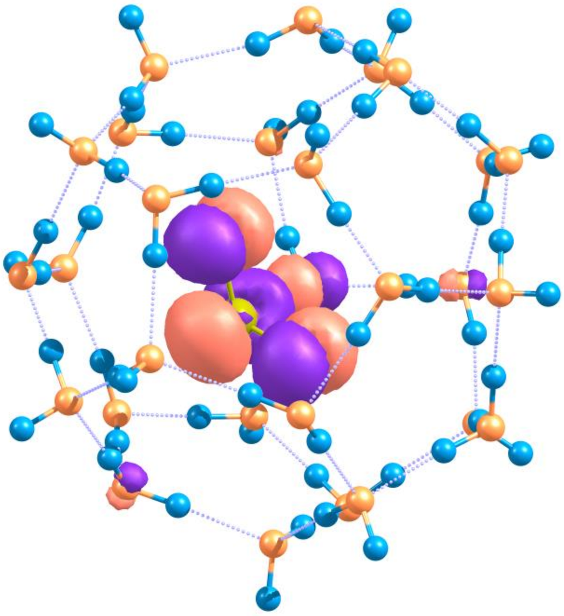

$5^{12}6^{4}$

Figure S34. RDG plot and their iso-surface from Non-covalent Interaction (NCI) analysis.

	5^{12}	$5^{12}6^{2}$	$5^{12}6^{4}$
CCl_4	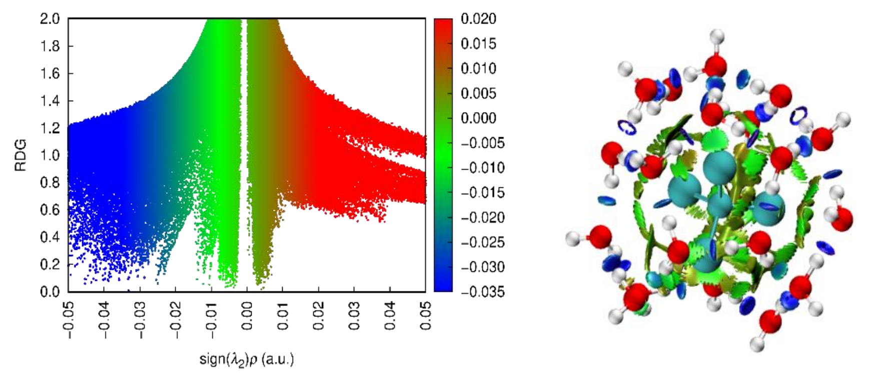	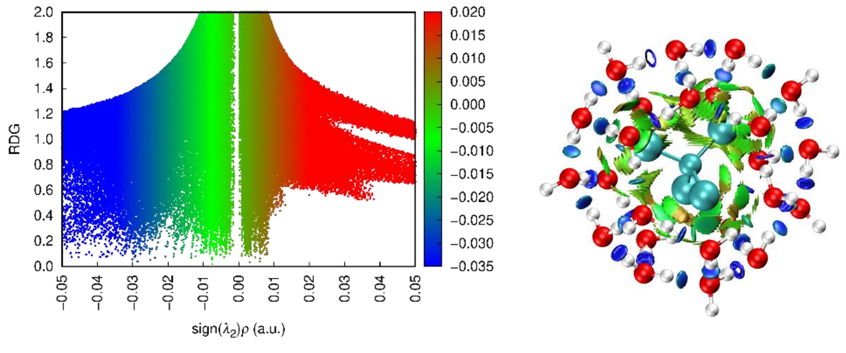	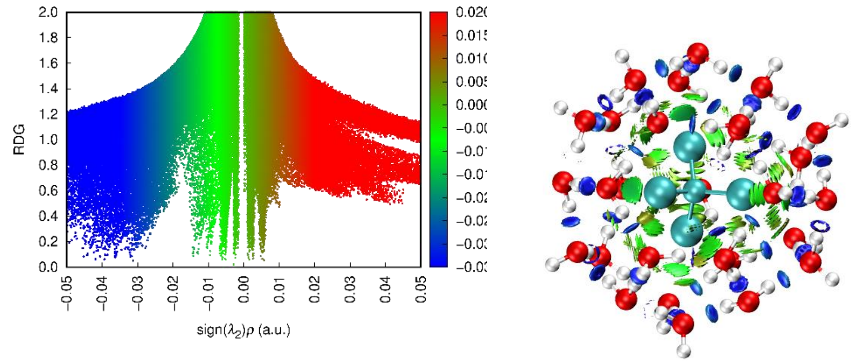
CF_2Cl_2	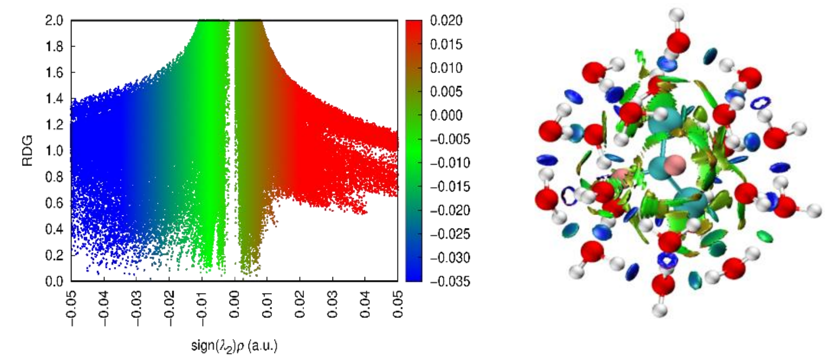	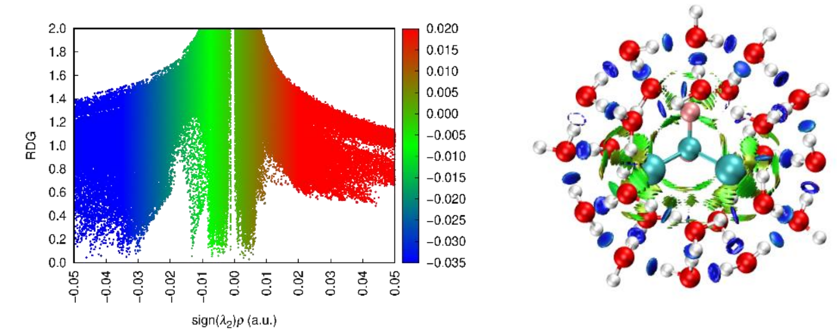	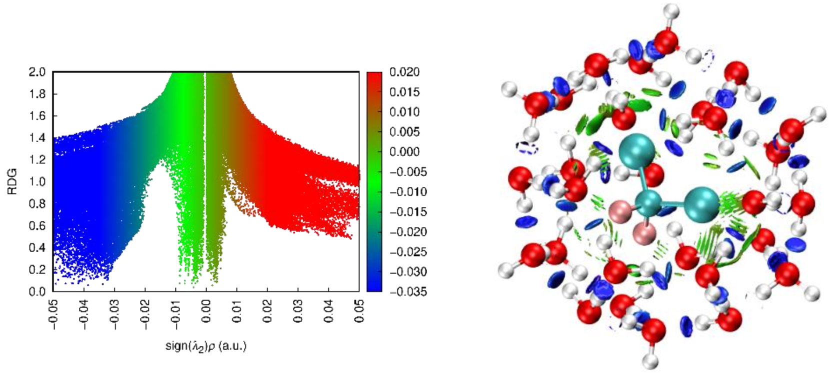
CH_3Br	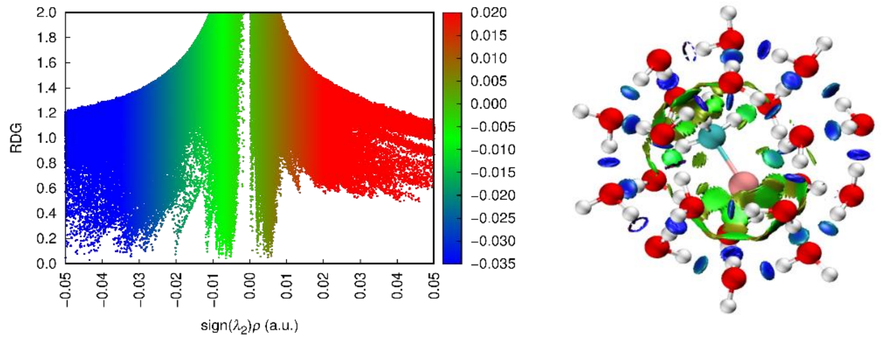	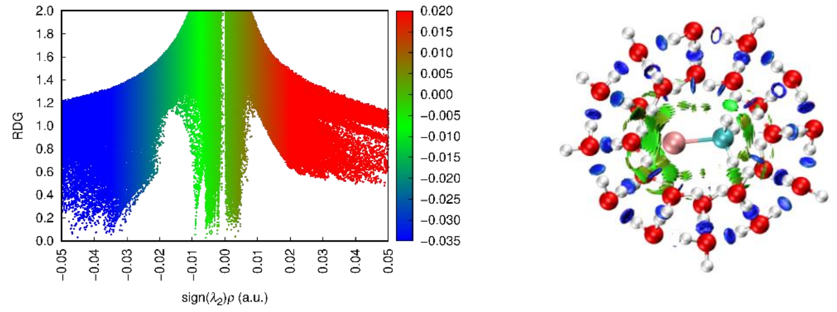	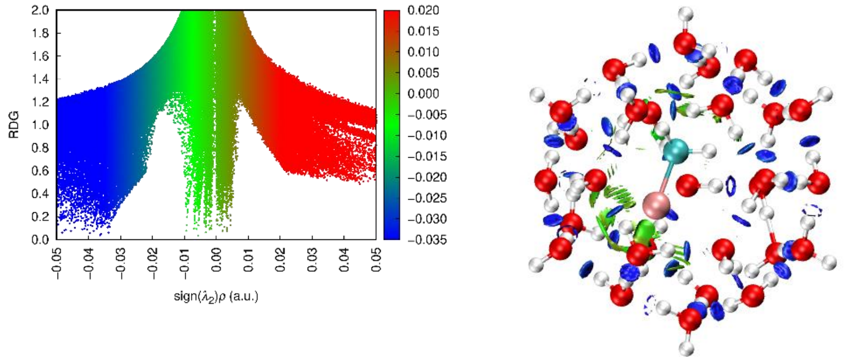

CH_3Cl

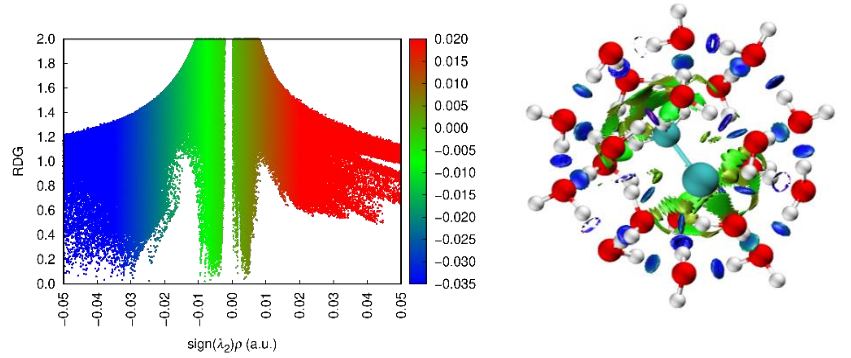

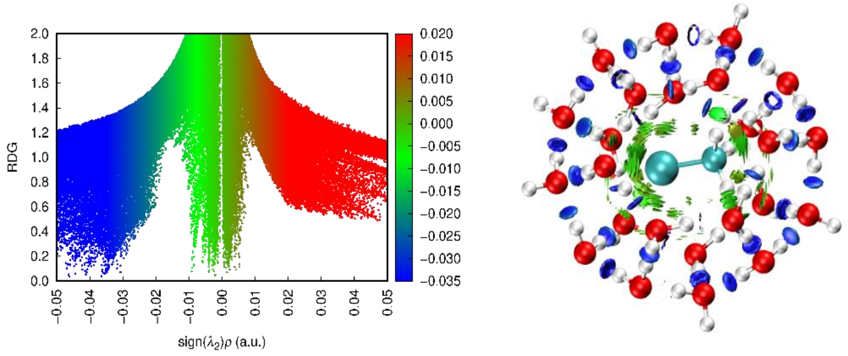

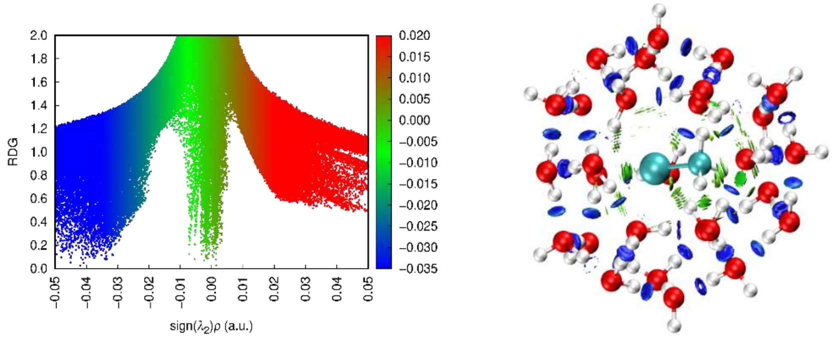

CH_4

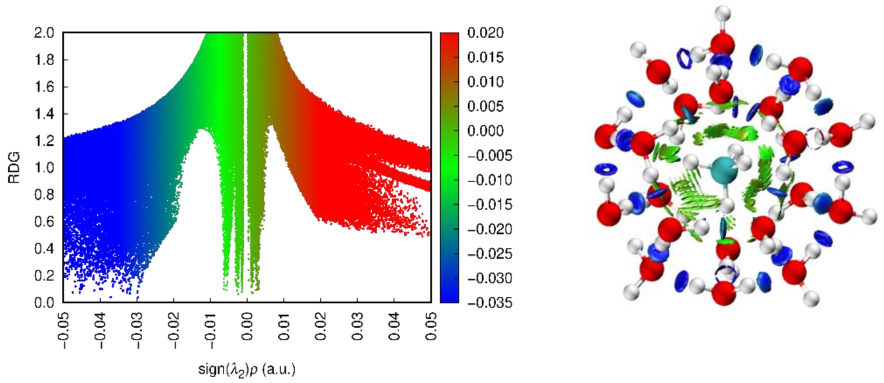

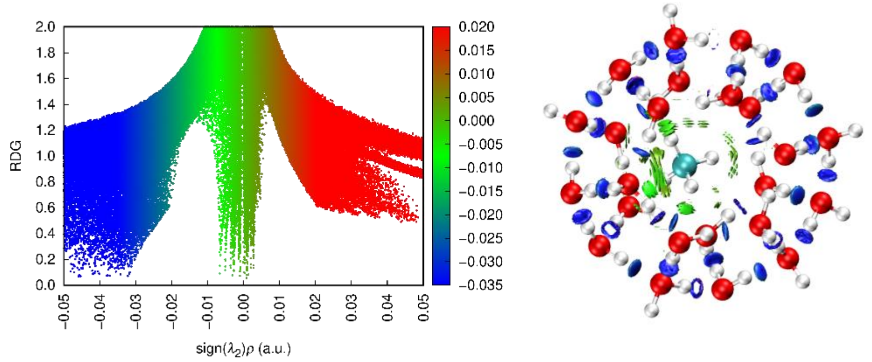

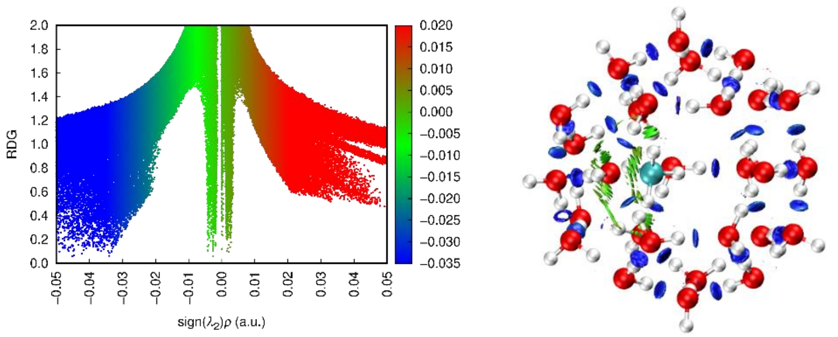

CO_2

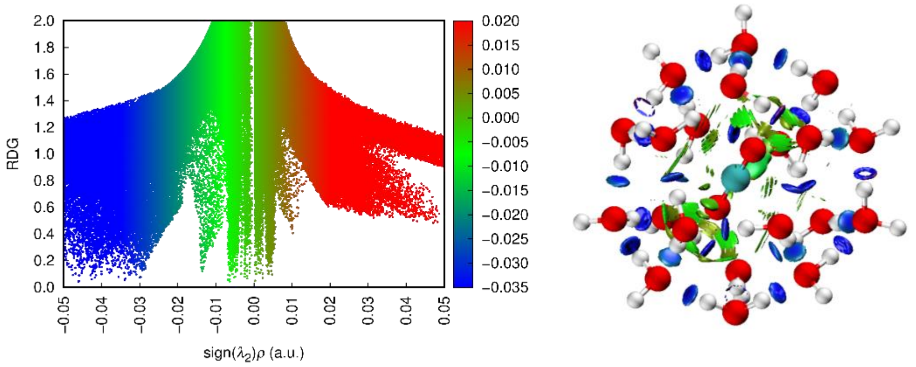

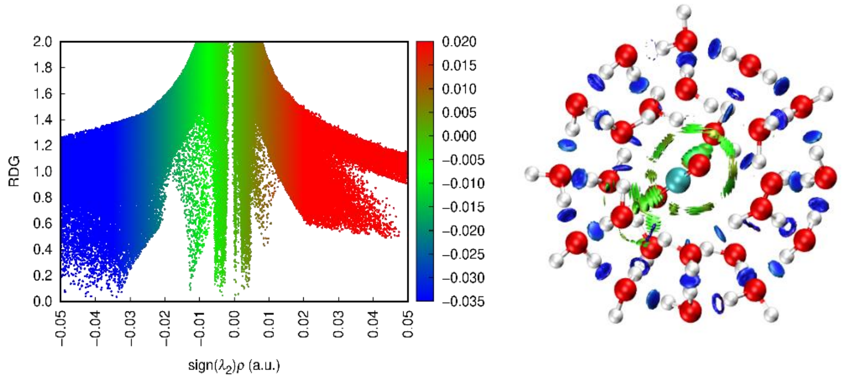

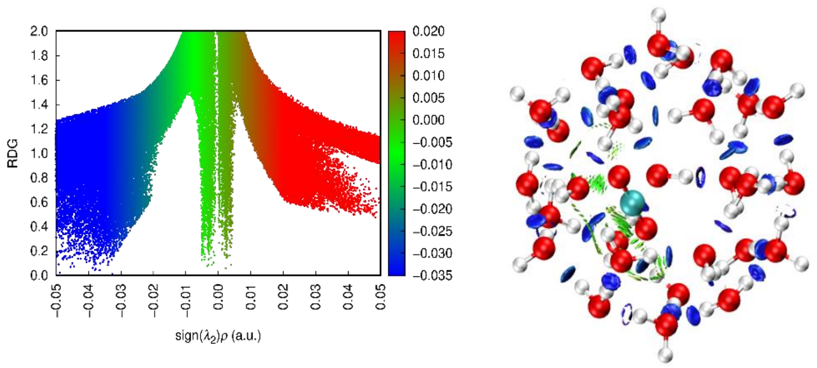

CO

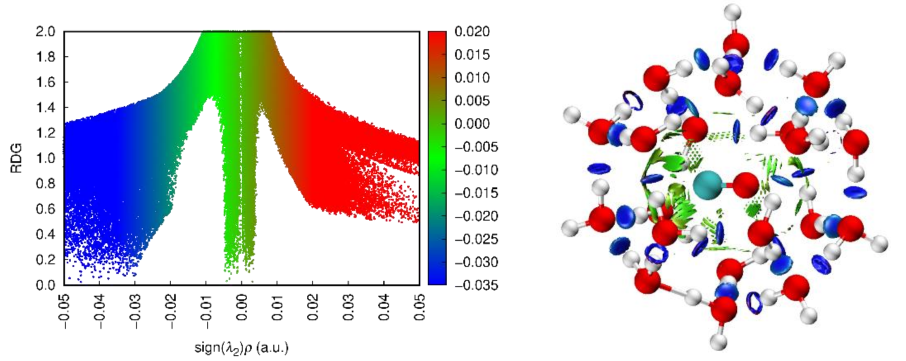

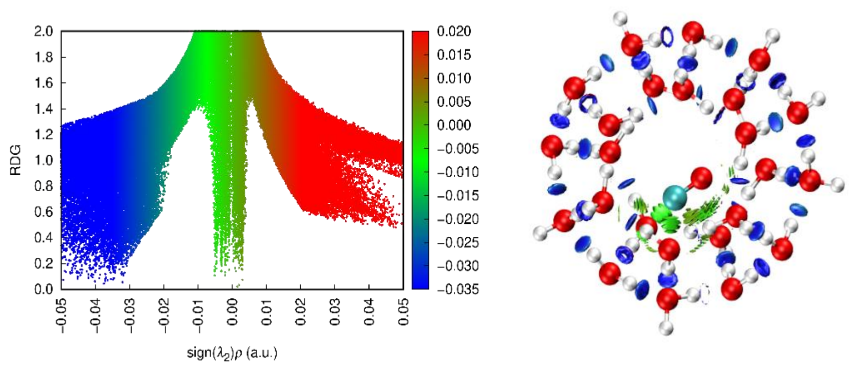

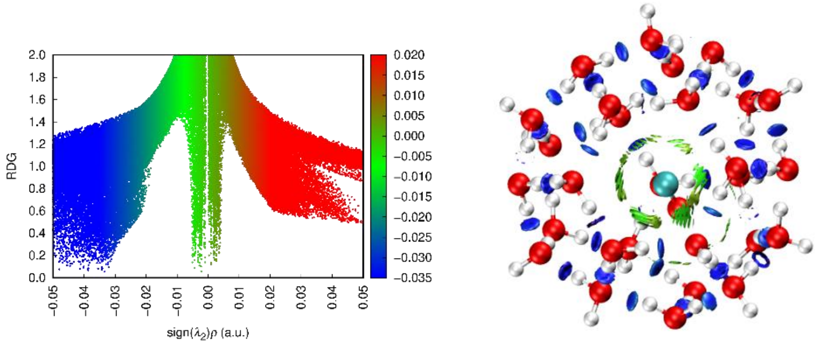

**H_2S**	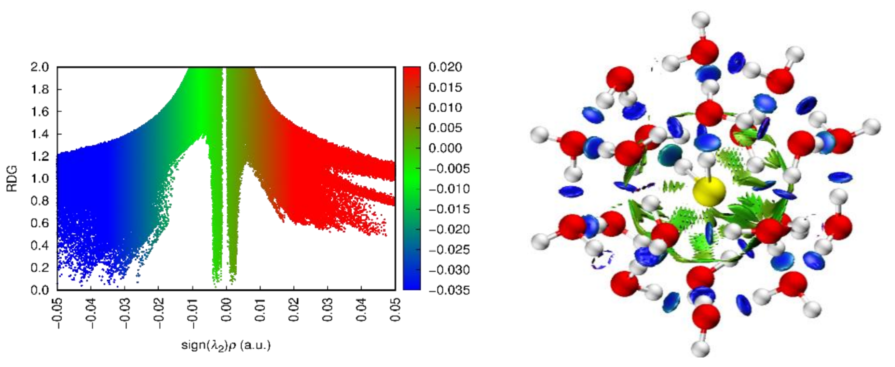	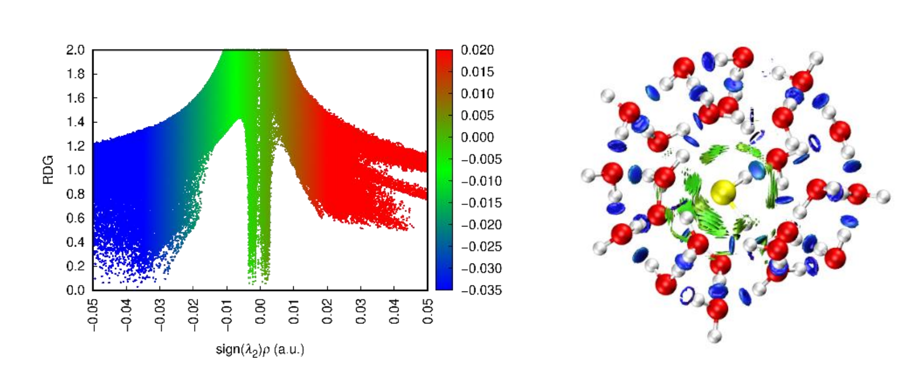	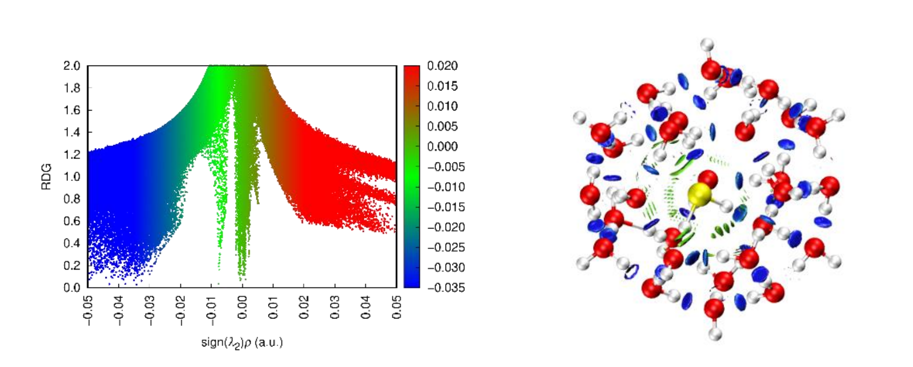
**CH_3F**	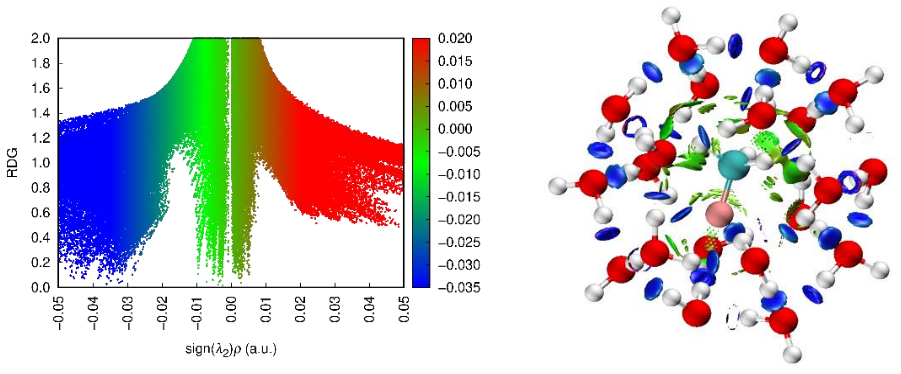	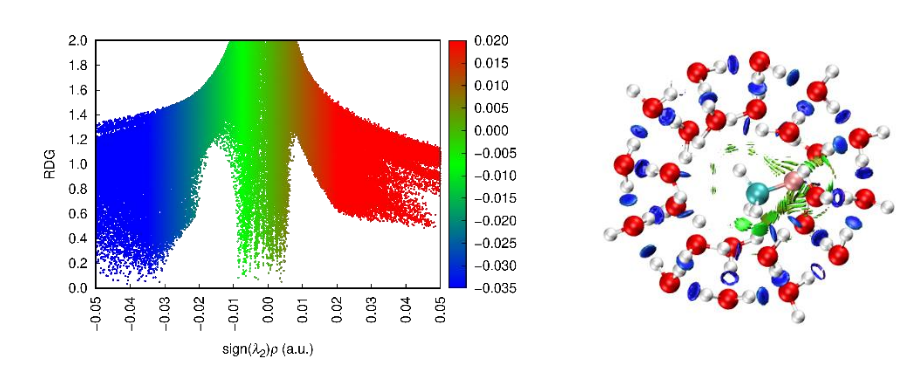	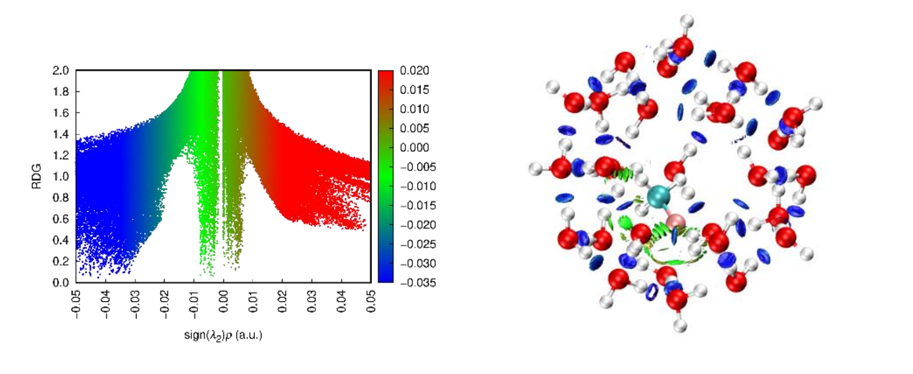
**N_2O**	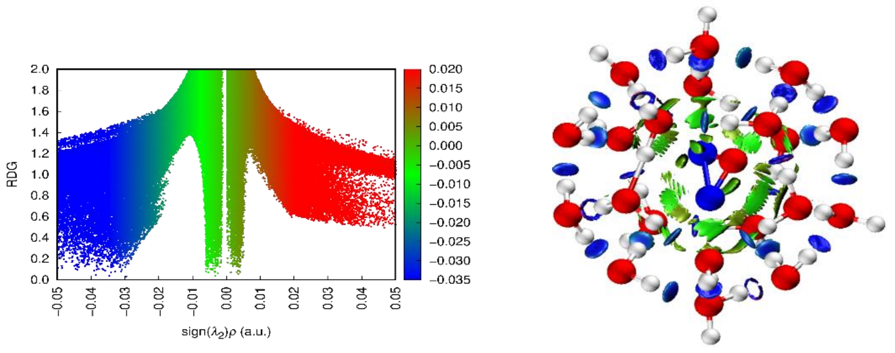	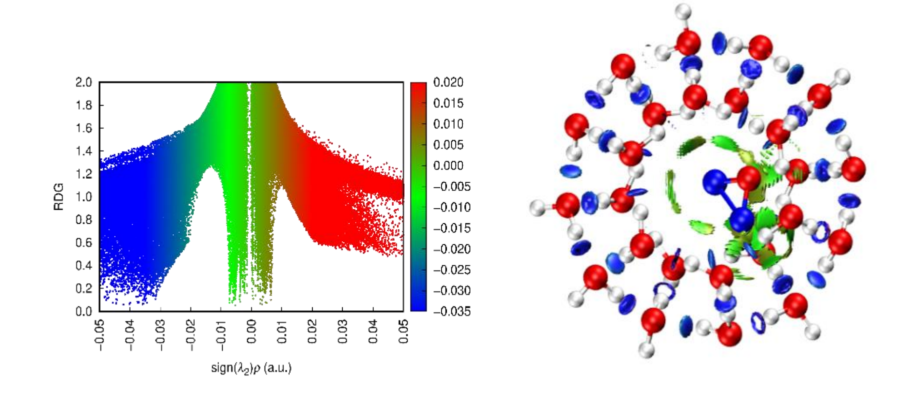	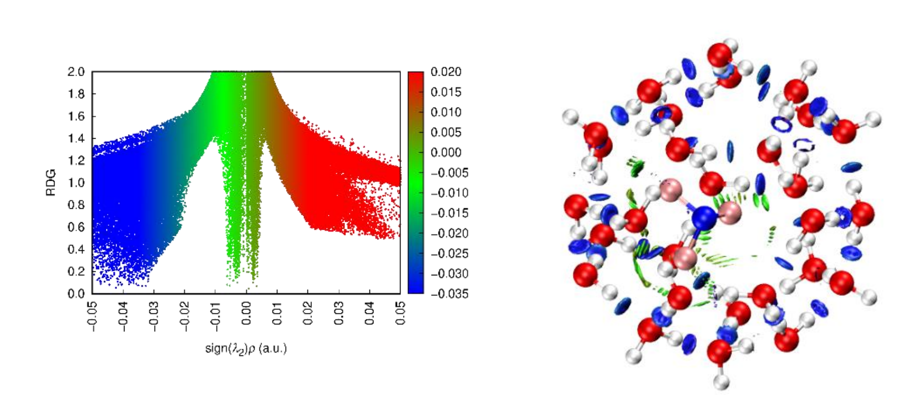
**NF_3**	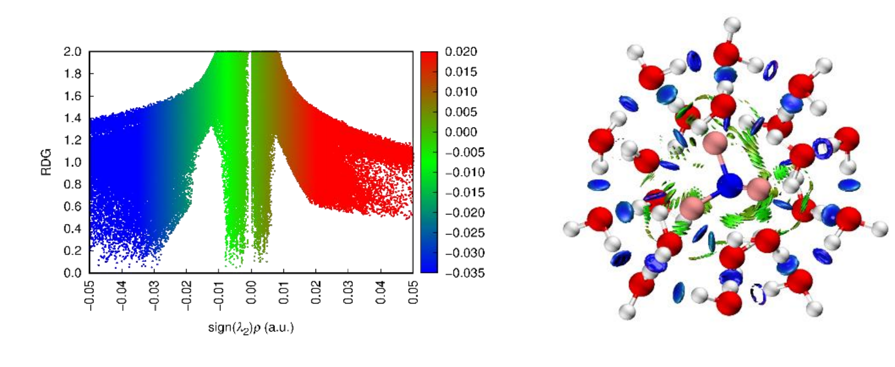	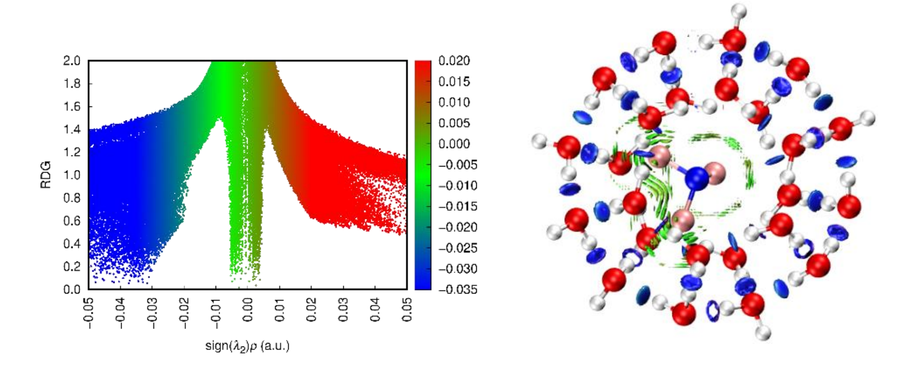	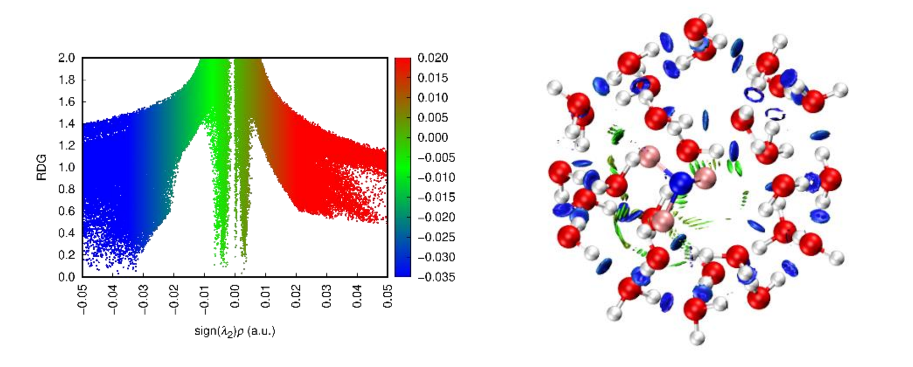

**O_3**	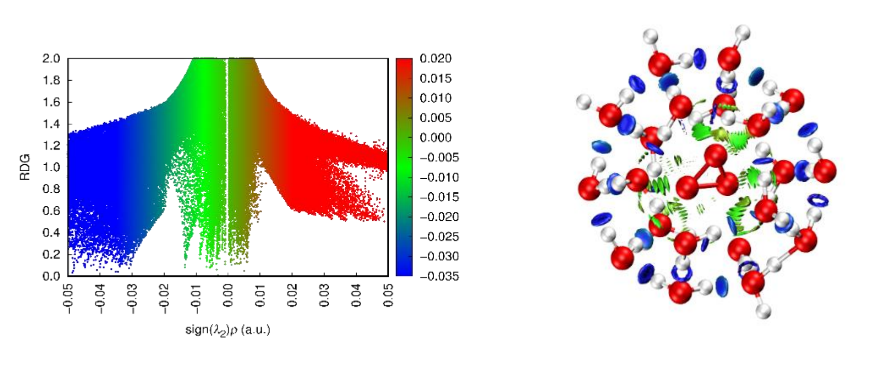	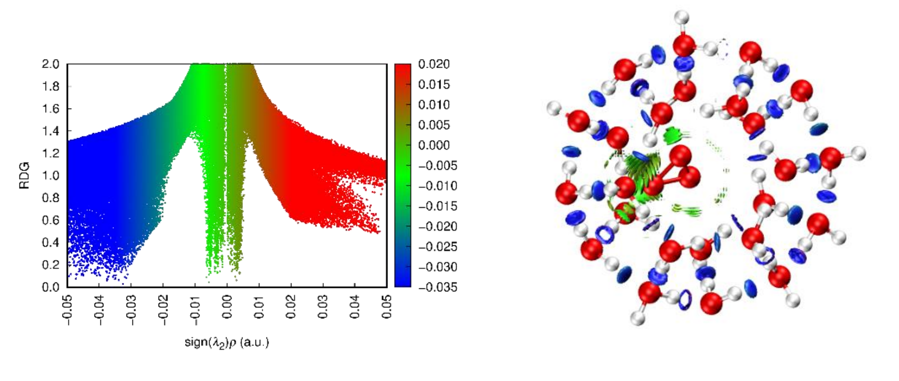	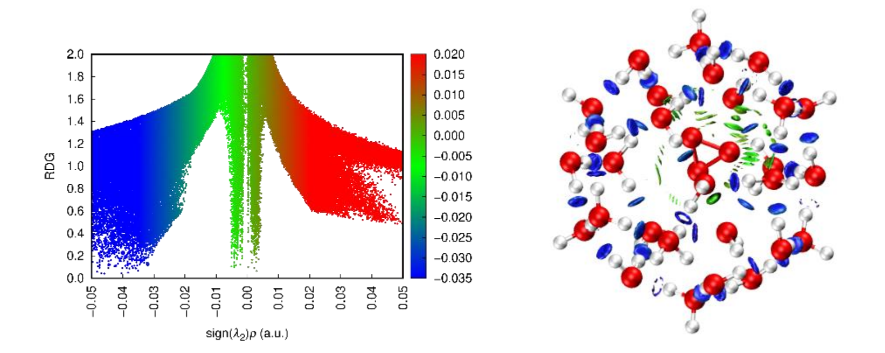
**CF_4**	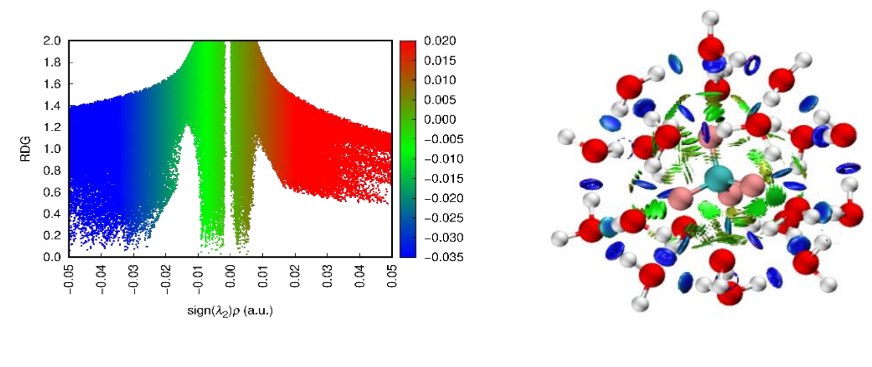	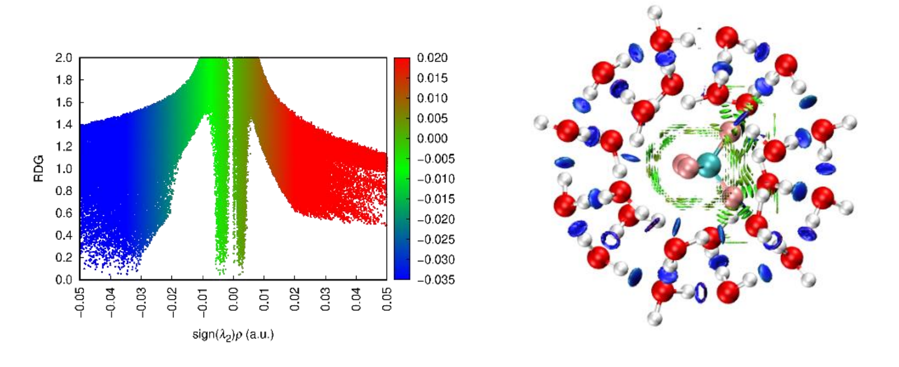	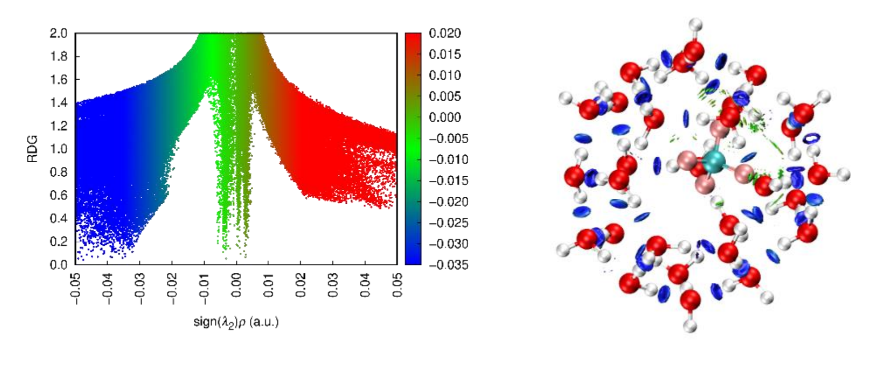
**SF_6**	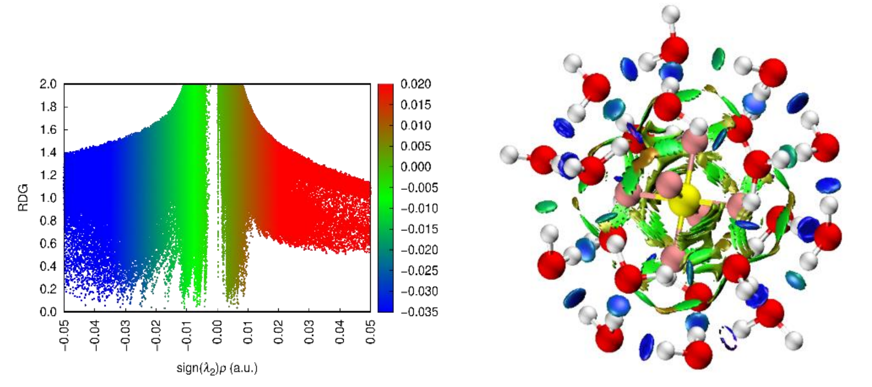	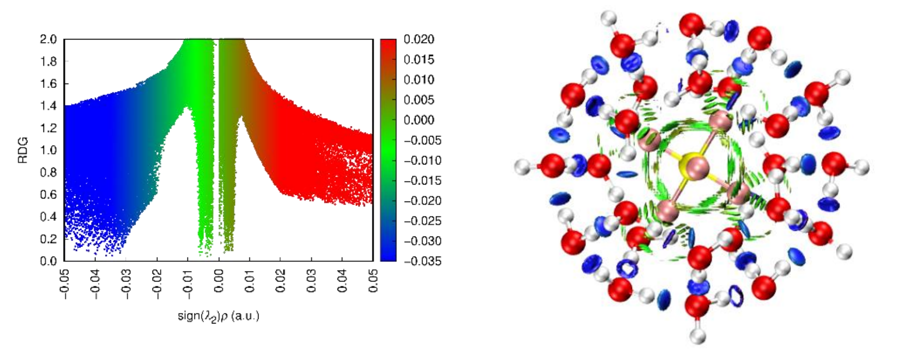	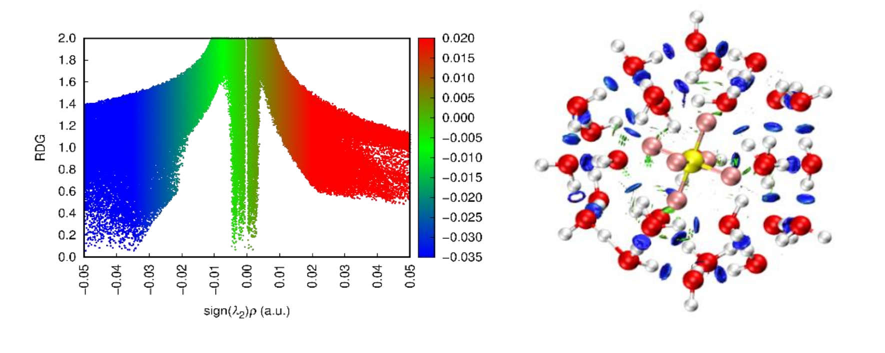
**SO_2**	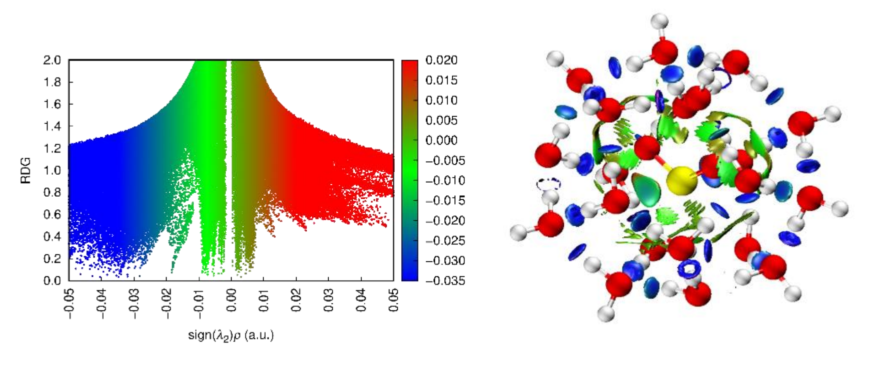	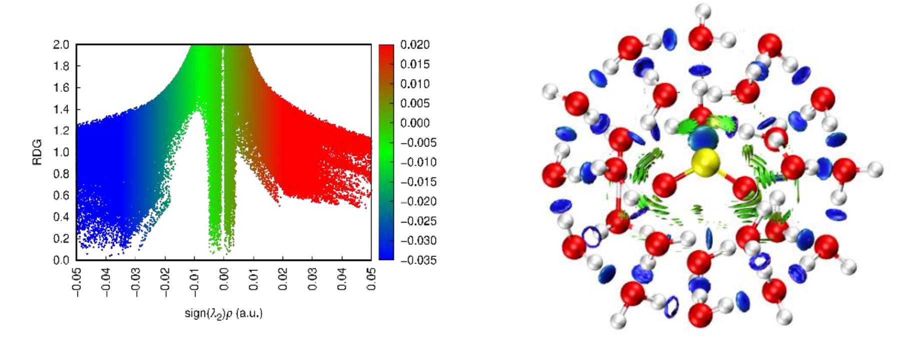	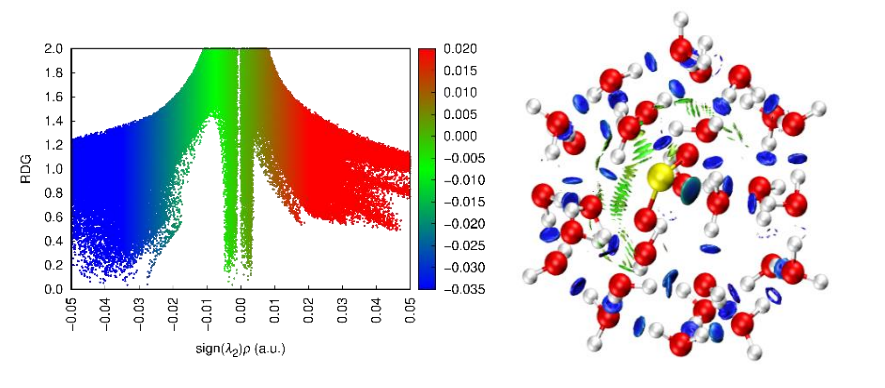

Electron localization function (ELF)

The electron localization function (ELF) has proven to be highly significant across a wide range of applications. This provides a chemically insightful measure of interactions between molecules and effectively identifies regions of electron delocalization.[1] It has been observed that ELF calculations produce distinct types of basins around attractors associated with either core or valence groups. The ELF determined through [2]

$$ELF(r) = \frac{1}{1 + [D(r)/D_0(r)]^2} \tag{6}$$

Where,

$$D(r) = \frac{1}{2}\sum_i \eta_i |\nabla\varphi_i|^2 - \frac{1}{8}\left[\frac{|\nabla\rho_\alpha(r)|^2}{\rho_\alpha(r)} + \frac{|\nabla\rho_\beta(r)|^2}{\rho_\beta(r)}\right] \tag{7}$$

$$D_0(r) = \frac{3}{10}(6\pi^2)^{2/3}\left[\rho_\alpha(r)^{5/3} + \rho_\beta(r)^{5/3}\right] \tag{8}$$

The initial term of *D(r)* represents the exact kinetic energy density of a non-interacting electron system, while the second term corresponds to the Weizsäcker kinetic energy density (T_w). Therefore, $D(r)= T_s(r) - T_w(r)$ highlights the additional kinetic energy density resulting from Pauli repulsion, known as the Pauli kinetic energy density. The $D_0(r)$ term can be understood as the Thomas-Fermi kinetic energy density (T_{TF}), which is the exact kinetic energy density of a uniform, non-interacting electron gas. Since $D_0(r)$ is used as the reference in ELF, the ELF essentially indicates the degree of relative electron localization.[3] Figure 8 illustrates the colored ELF map, which was generated using Multiwfn software. High and low ELF values correspond to regions of high and low electron density, respectively, and are indicative of covalent and non-covalent interactions.

These ELF changes have significant implications. Enhanced polarization or localization, as seen in polar molecules, often strengthens interactions with the clathrate cage, stabilizing the encapsulated molecule. Conversely, dispersed electron density, typical in weakly polarized molecules, correlates with bond weakening, as reflected in shifts in vibrational frequencies. ELF analysis aids in understanding the stability of within clathrate cages. Molecules such as

CO_2, CH_4, and SF_6 trapped in clathrates may exhibit reduced reactivity or mobility, contributing to greenhouse gas sequestration and mitigating environmental harm. Thus, ELF provides a comprehensive understanding of the electronic environment changes induced by clathrate encapsulation, essential for predicting guest molecule behaviour. The ELF analysis offers qualitative insights by graphically representing areas of electron localization.

The Pauli exclusion principle underpins many chemical concepts, including ELF, which provides insights based on this principle.[4] As Paulis reported, ELF is instrumental in understanding the exchange repulsive effect and chemical bonding between interacting molecules, addressing questions raised by Lewis.[5] Based on the ELF color scale and values, ELF values between 0.5 and 1 (Red/Blue) signify regions of localized bonding and non-bonding electrons, where the electron density distribution approaches homogeneity. Conversely, ELF values below 0.5 (Red) indicate delocalized electrons, often reflecting metallic characteristics.[6] Here, the chemical bonding nature was explored through the topological analysis of ELF. The results show minor changes in electron density following the formation of the complex. The electron density of both the guest and host molecules undergoes changes upon encapsulation within all the three types of hydrate clathrate cages. The Electron Localization Function (ELF) images aid in understanding the transitions occurring between the guest and host. These transitions result in alterations in the electron density of the atoms within the hydrate clathrate cages. According to Bader et al., ELF and the negative Laplacian of the electron density convey equivalent information. The Electron Localization Function (ELF) provides insights into the spatial distribution of electron density and bonding characteristics in molecules. When are encased within clathrate hydrate cages, their electronic structure, particularly bond electron density, is influenced by interactions with the hydrate framework. For weakly polarized molecules like CCl_4, CF_2Cl_2 and CF_4 ELF analysis shows that before encapsulation, electron density is highly localized around covalent bonds with minimal delocalization, maintaining symmetry. After encapsulation, the clathrate environment perturbs the outer electron cloud, slightly redistributing electron density. Highly electronegative atoms (e.g., F, Cl) experience enhanced polarization of electron density due to interactions with the polar water framework of the cage, weakening bond localization.

Polar molecules such as CH_3Br, CH_3Cl, H_2S and SO_2 display significant ELF asymmetry even before encapsulation due to their dipole moments. After encapsulation, the cage interactions amplify this polarization, with increased electron localization near electronegative atoms. This stabilization of high electron density regions can slightly shift bond lengths and angles, as seen

in SO_2, where sulphur shows increased electron localization, and in H_2S, where lone pair regions become more localized. Strongly bonded molecules like CO, CO_2, and N_2O) exhibit highly localized ELF distributions along their bonding axes (e.g., C≡O in CO, or C=O in CO_2). While these molecules maintain rigid bonding frameworks, the clathrate cage can distort ELF distributions slightly, particularly near lone pairs. For CO, the anisotropic interactions of its C≡O bond with the cage lead to inconsistent ELF changes, while for N_2O, ELF redistribution occurs near terminal atoms, reflecting weak stabilization. Highly polarizable molecules like SF_6 and CF_4 display highly localized ELF near electronegative atoms. Upon encapsulation, the hydrate cage induces stronger polarization in bonds due to its electric field, as evidenced by more localized electron density around F atoms and slightly reduced density near central atoms (S or C). In SF_6, subtle ELF changes around sulphur highlight these interactions.

Figure S35. Electron Localized function (ELF) Mapping of Greenhouse gases encased within 5^{12} hydrate clathrates.

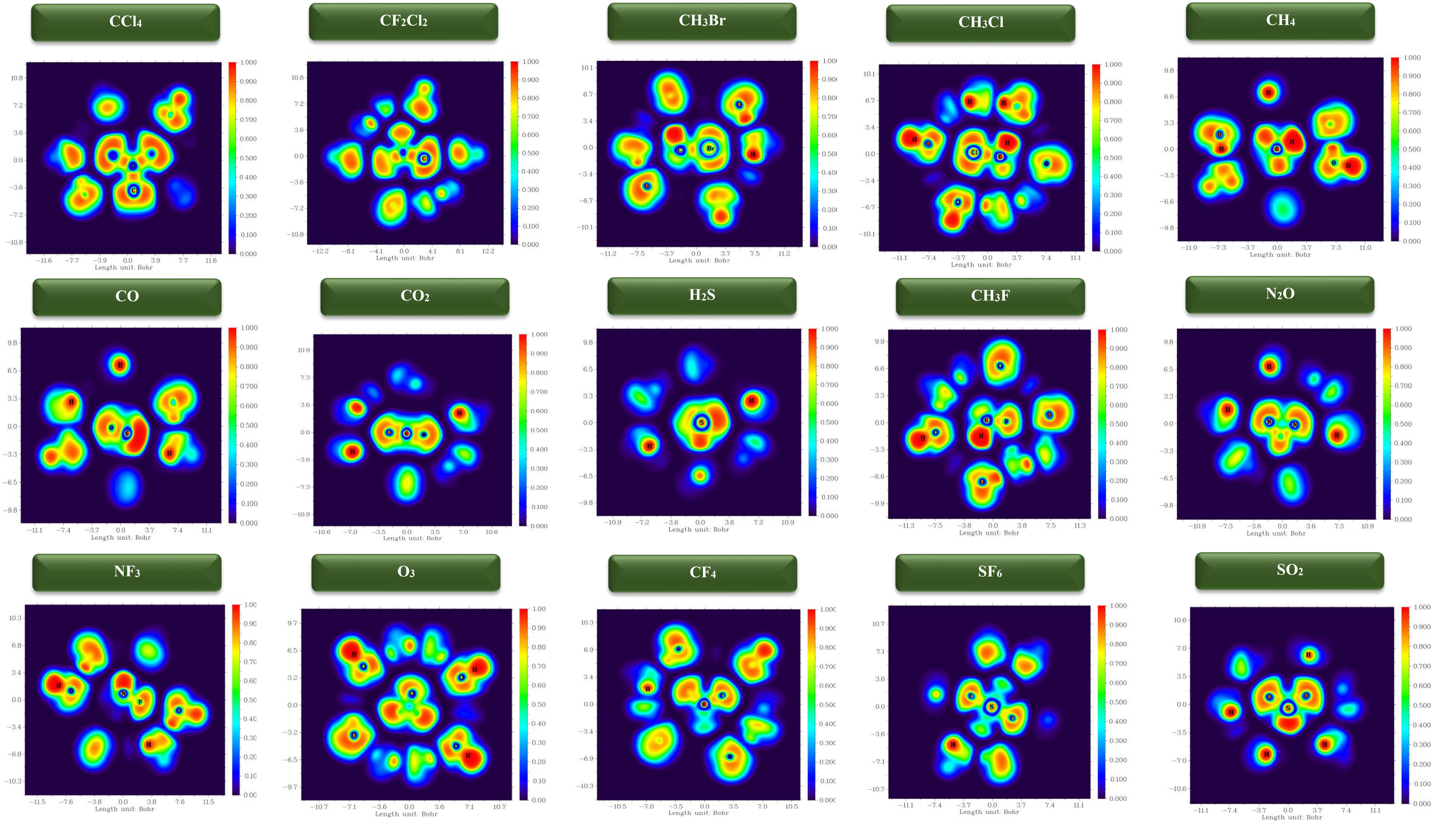

Figure S36. Electron Localized Function (ELF) Mapping of Greenhouse gases encased within $5^{12}6^{2}$ hydrate clathrates.

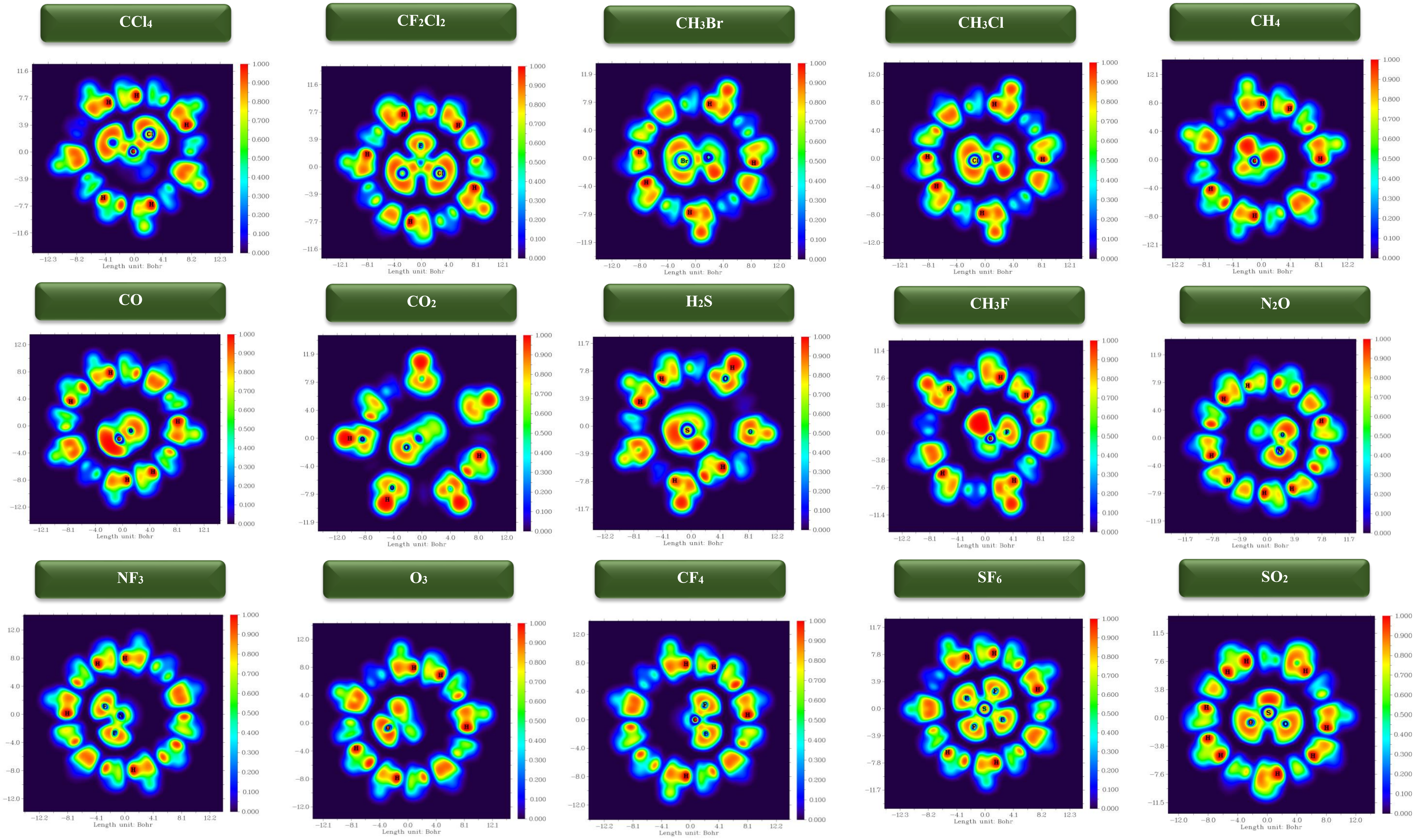

Figure S37. Electron Localized Function (ELF) Mapping of Greenhouse gases encased within $5^{12}6^4$ hydrate clathrates.

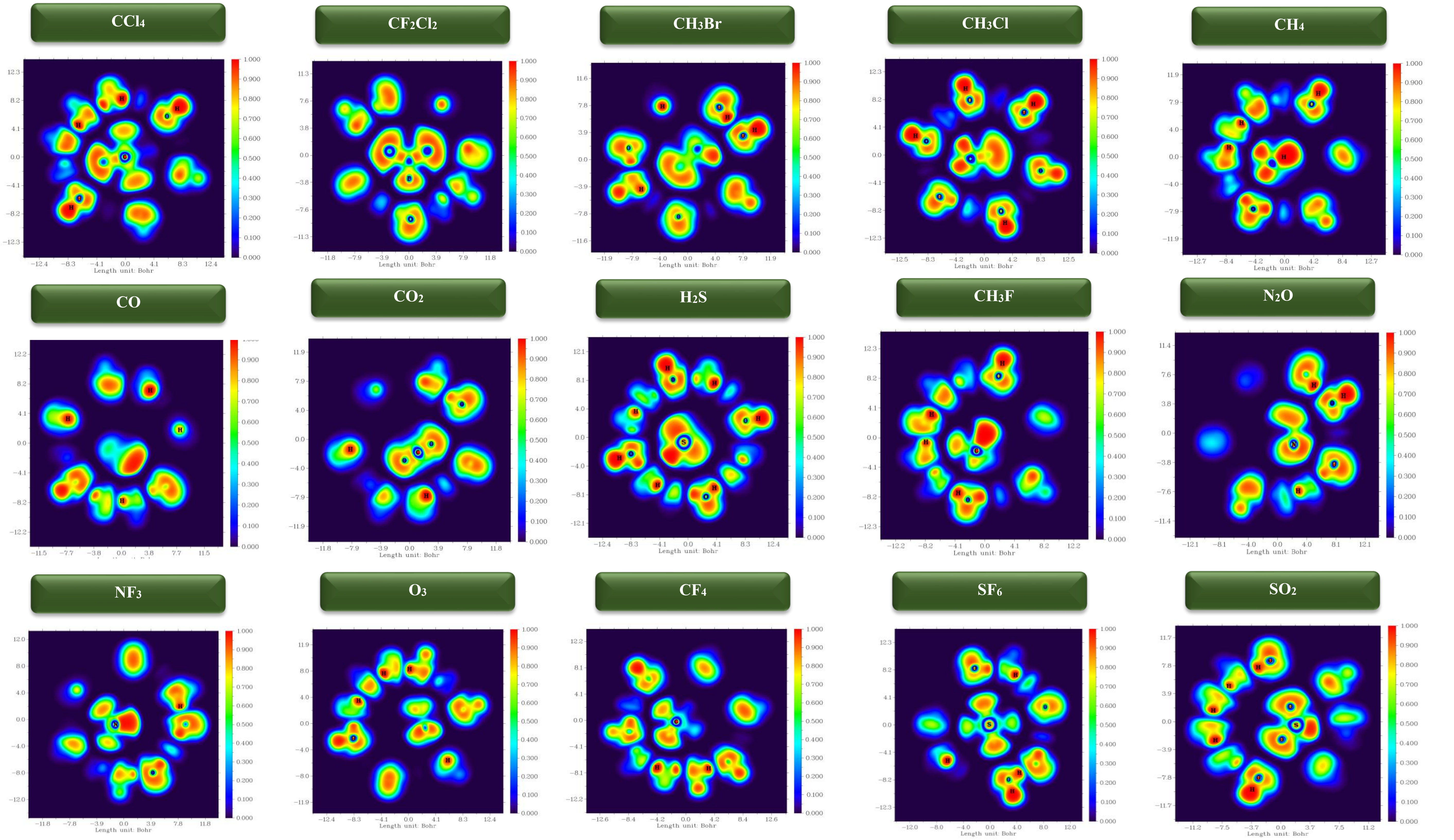

Figure S38. Bar plot for the energy gap among the Greenhouse gases encapsulation in 5^{12}, $5^{12}6^{2}$ and $5^{12}6^{4}$ clathrate hydrates.

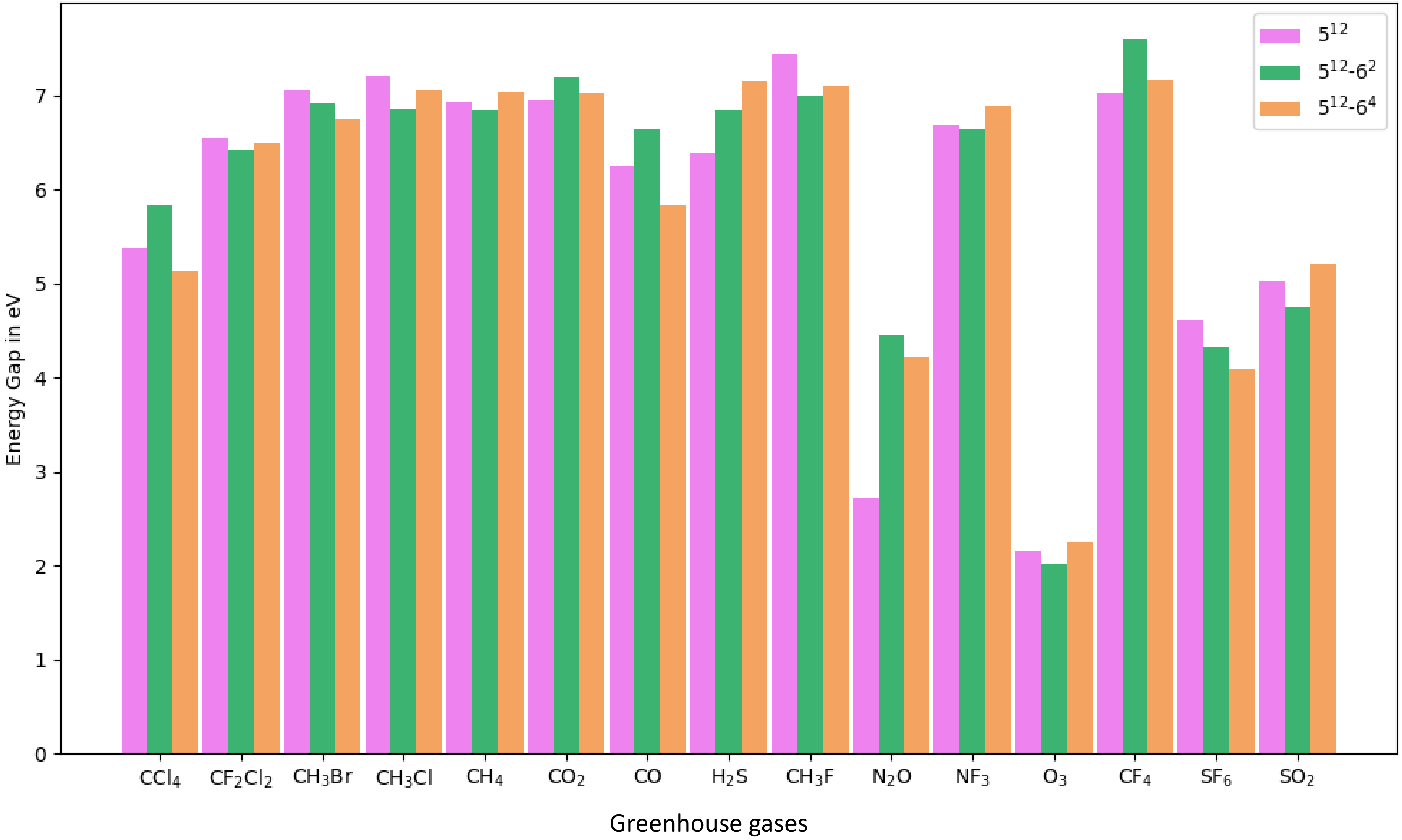

Figure S39. Bar plot for the chemical potential among the Greenhouse gases encapsulation in 5^{12}, $5^{12}6^2$ and $5^{12}6^4$ clathrate hydrates.

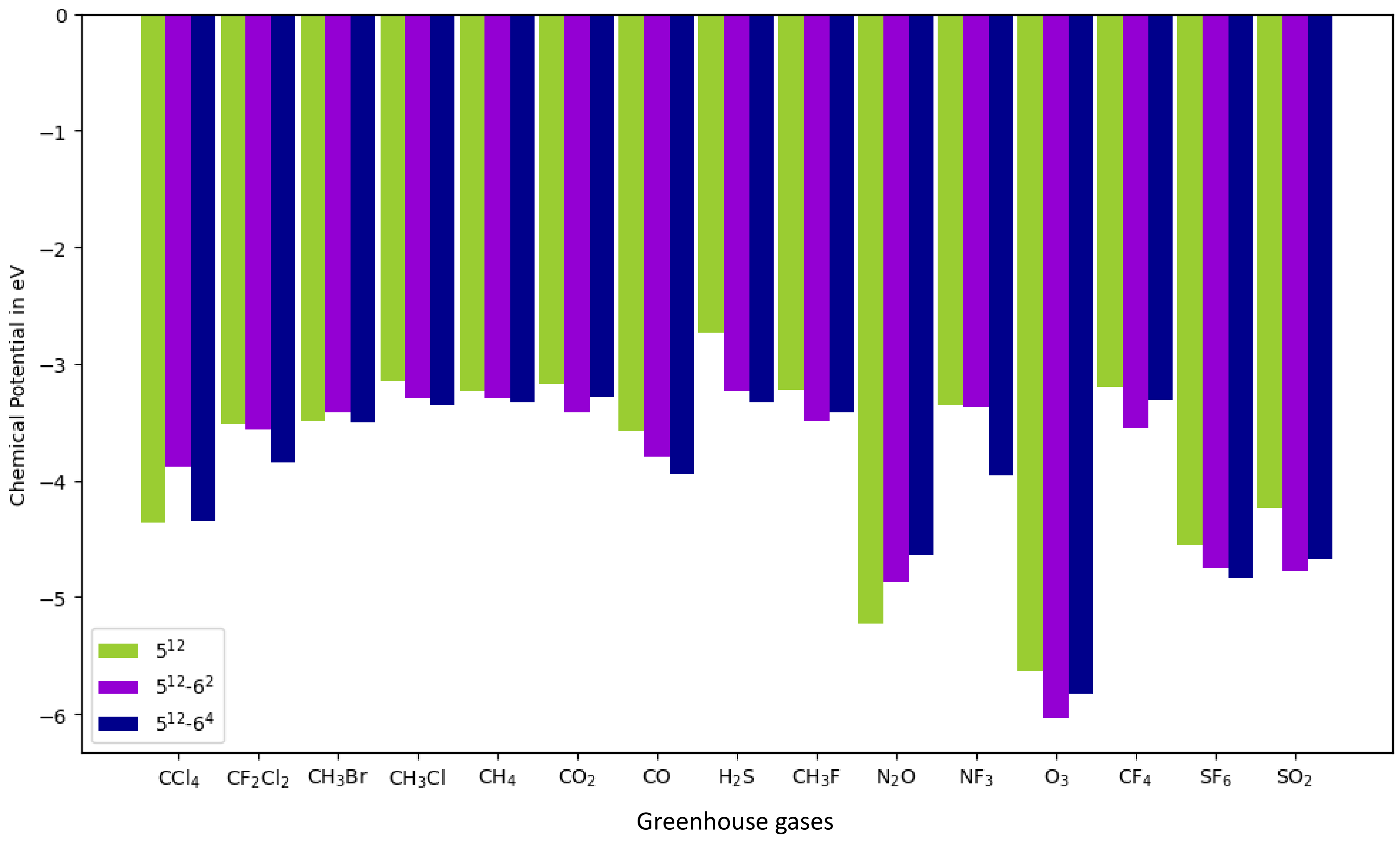

Figure S40. Bar plot for the chemical hardness among the Greenhouse gases encapsulation in 5^{12}, $5^{12}6^{2}$ and $5^{12}6^{4}$ clathrate hydrates.

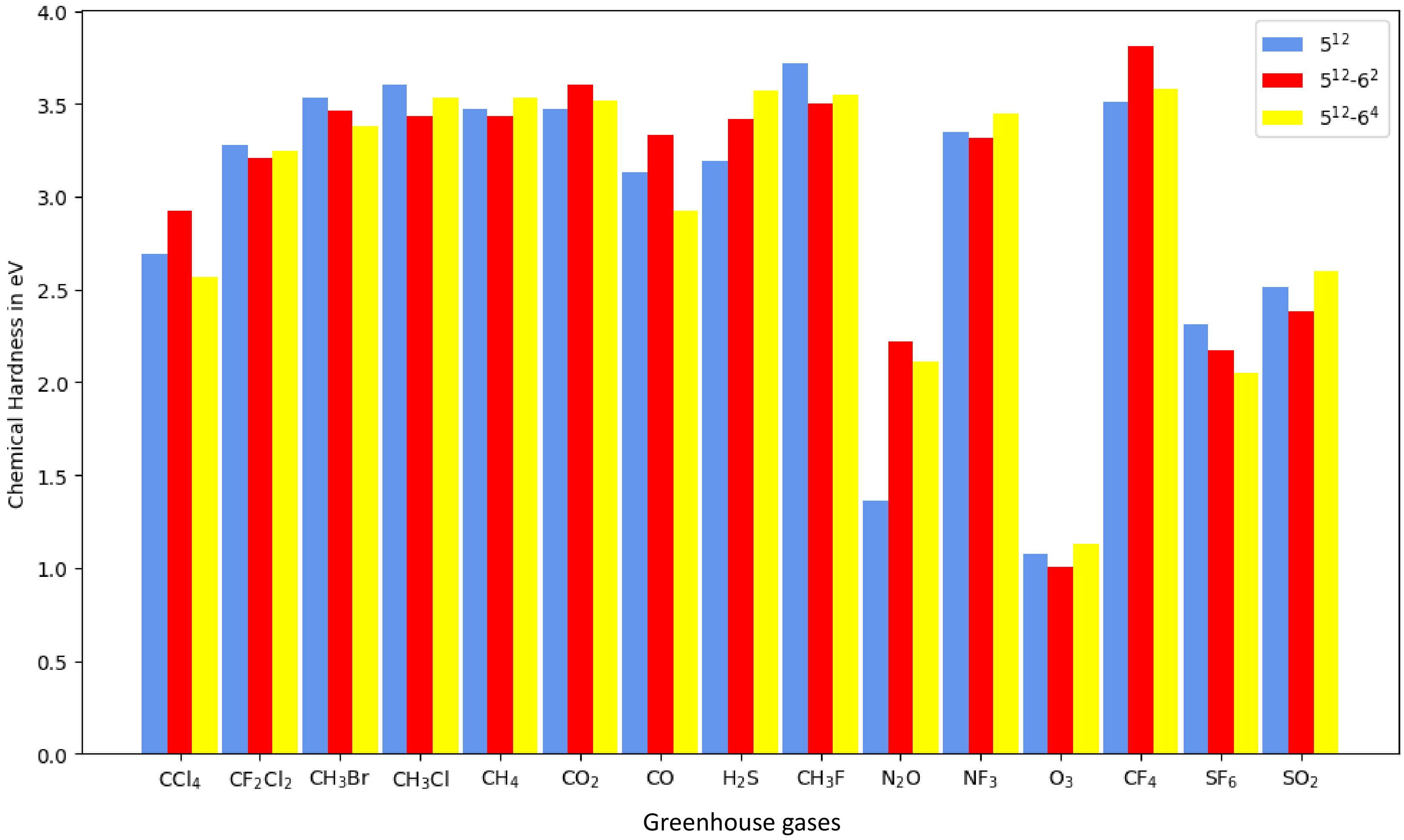

Figure S41. Bar plot for the electronegativity among the Greenhouse gases encapsulation in 5^{12}, $5^{12}6^2$ and $5^{12}6^4$ clathrate hydrates.

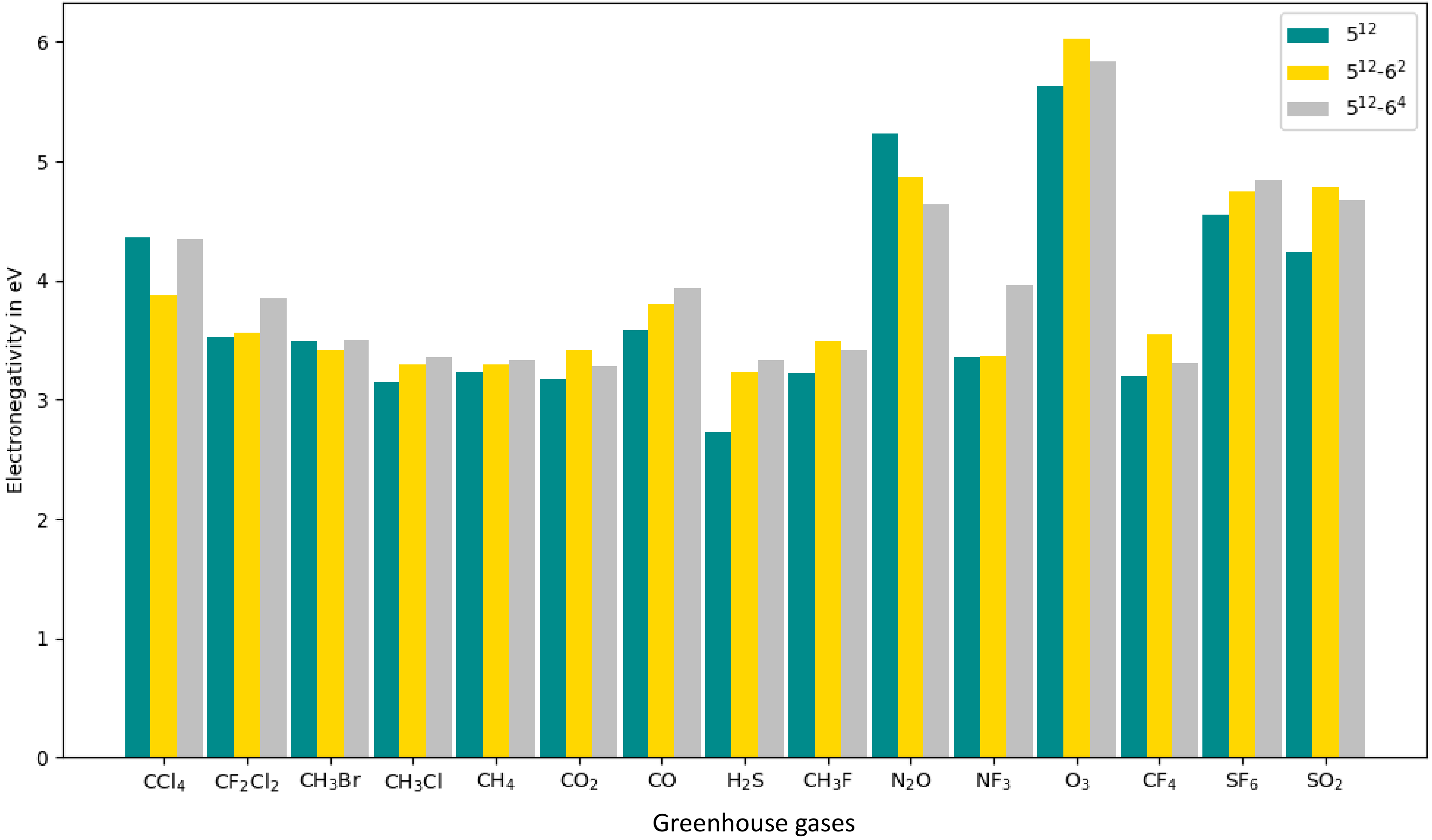

Figure S42. Bar plot for the electrophilicity index among the Greenhouse gases encapsulation in 5^{12}, $5^{12}6^{2}$ and $5^{12}6^{4}$ clathrate hydrates.

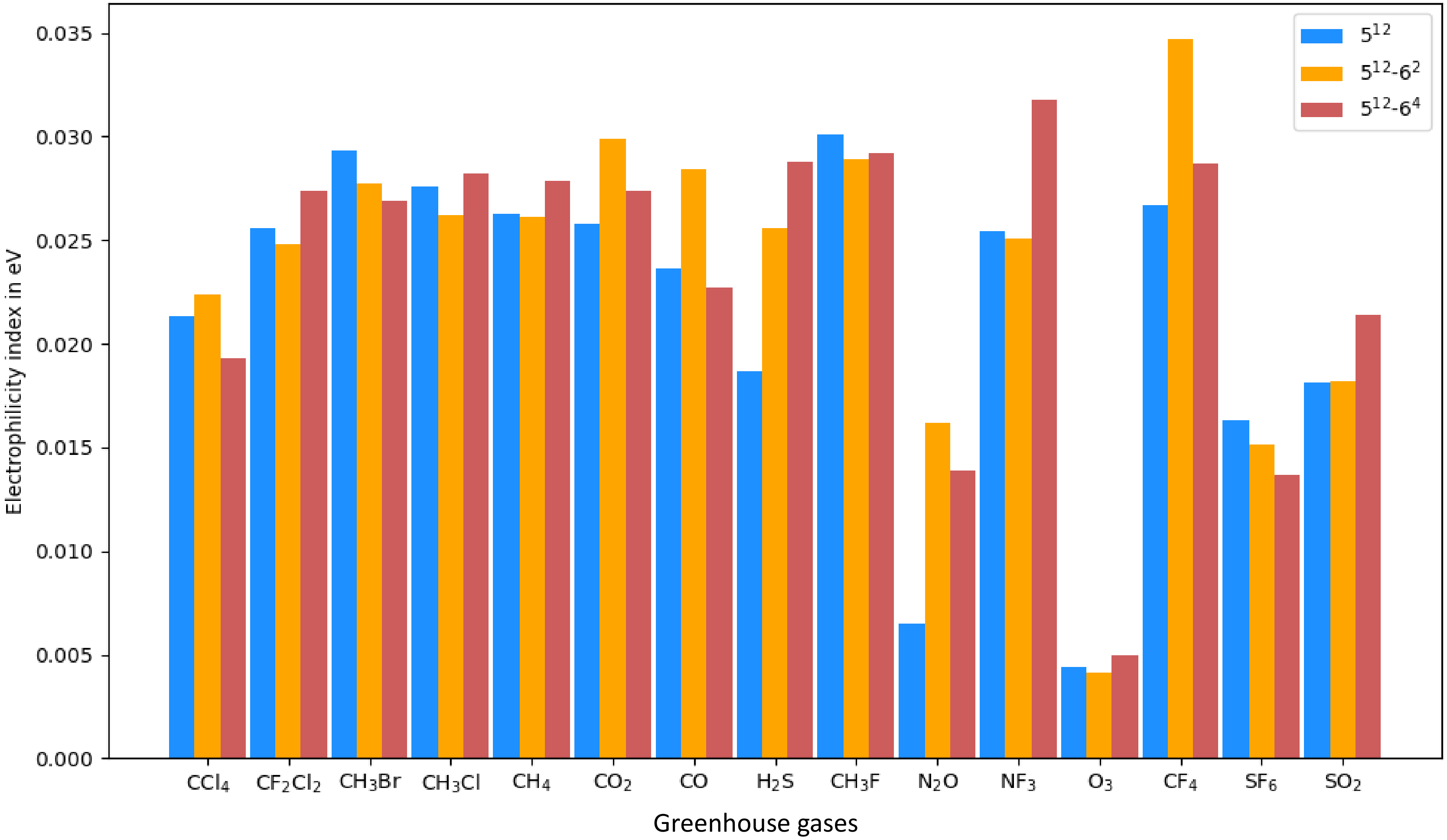

Figure S43. Plot for the relation between the $\Delta\nu$ (in cm^{-1}) and Δr (in Å) for GHGs such as CH_3Br, CH_3Cl, CF_2Cl_2 and CH_3F within the three hydrate clathrate cages of their bonds C-Br, C-Cl, C-F, and C-F respectively.

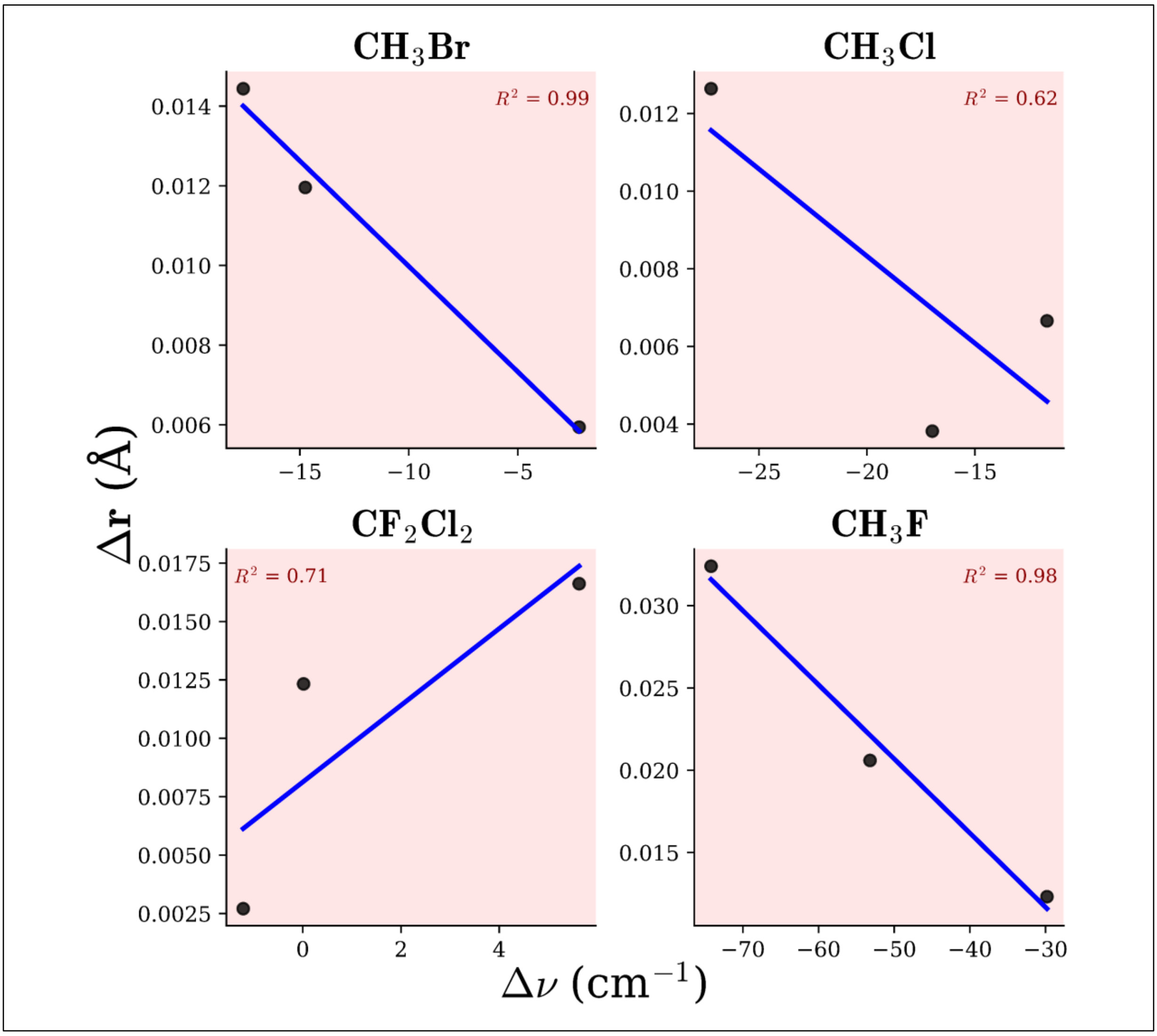

Figure S44. Plot for the relation between the $\Delta\nu$ (in cm^{-1}) and $\nabla^2 \rho(r)$ (in a.u.) for GHGs such as CH_3Br, CH_3Cl, CF_2Cl_2 and CH_3F within the three hydrate clathrate cages of their bonds C-Br, C-Cl, C-F, and C-F respectively.

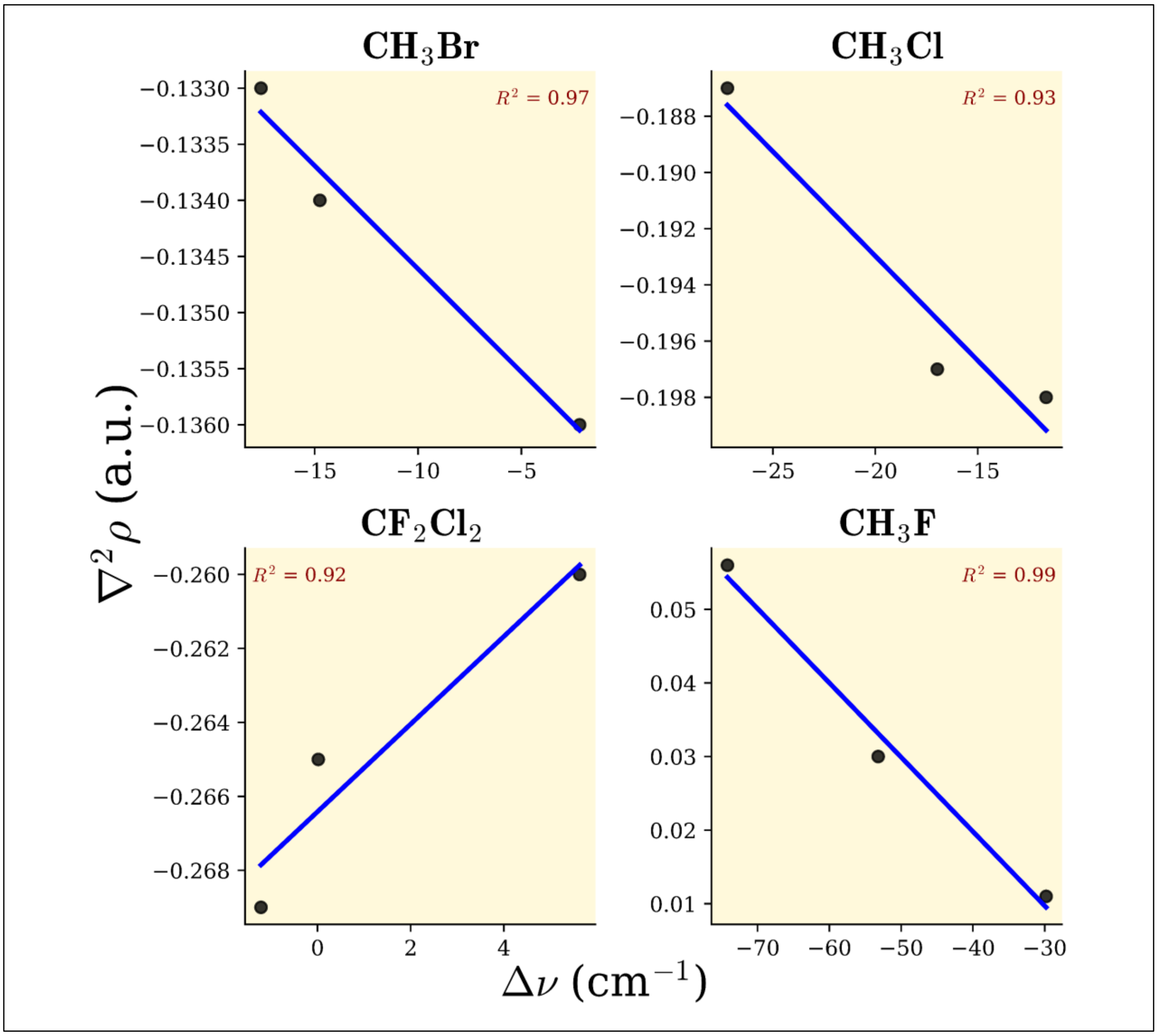

Figure S45. Plot for the relation between the Δr (in Å) and $\nabla^2 \rho(r)$ (in a.u.) for GHGs such as CH_3Br, CH_3Cl, CF_2Cl_2 and CH_3F within the three hydrate clathrate cages of their bonds C-Br, C-Cl, C-F, and C-F respectively.

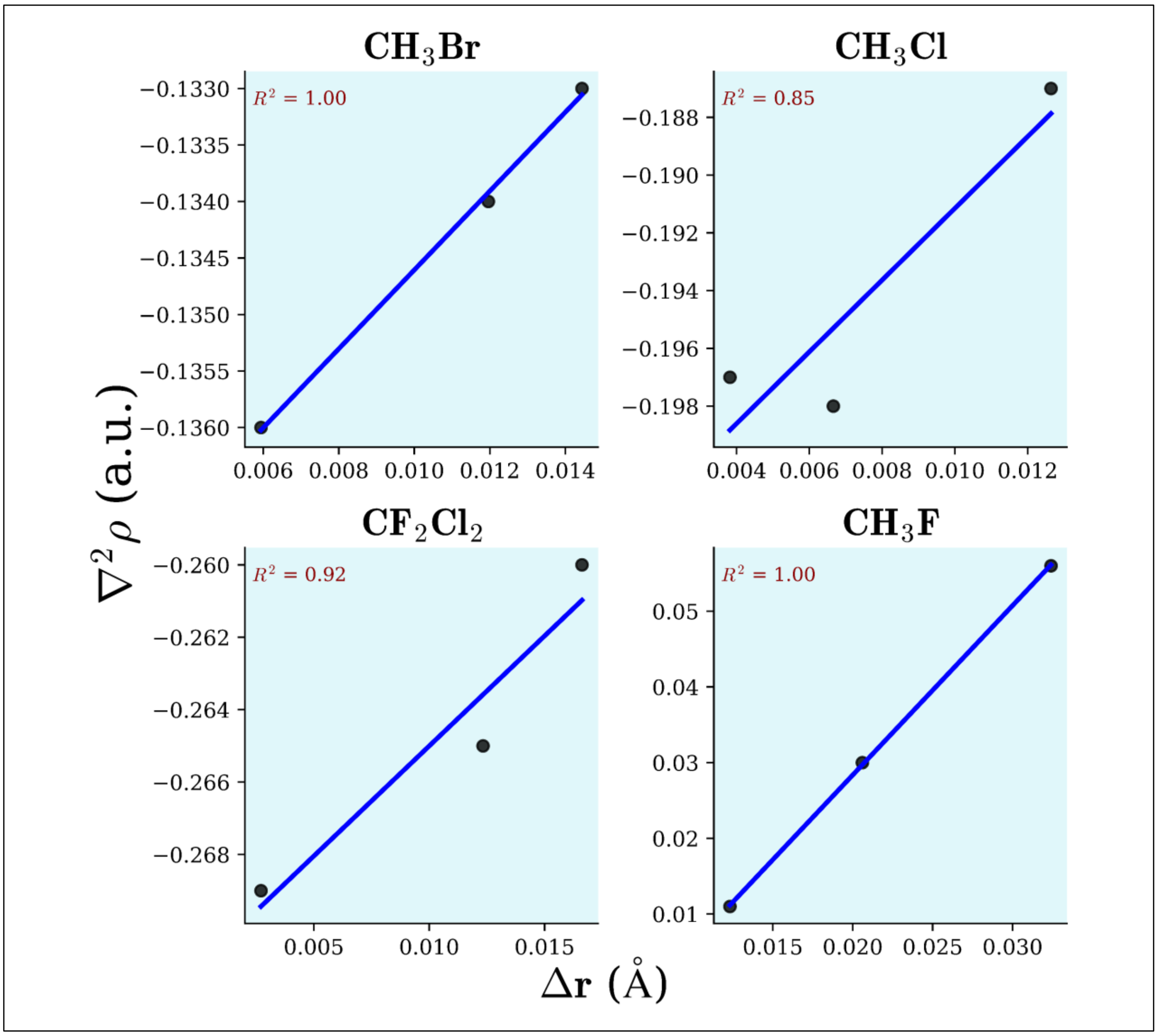

Table S17. Cartesian coordinates for optimized geometries of the guest molecule monomers computes at B3LYP/6-31G(d) level of theory and basis set.

CCl_4

C	0.000000000	0.000000000	0.000000000
Cl	1.034552000	1.034552000	1.034552000
Cl	-1.034552000	-1.034552000	1.034552000
Cl	1.034552000	-1.034552000	-1.034552000
Cl	-1.034552000	1.034552000	-1.034552000

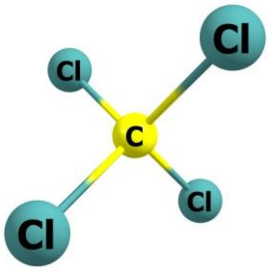

CF_2Cl_2

C	0.000000000	0.000000000	0.344814000
Cl	0.000000000	1.479072000	-0.655712000
Cl	0.000000000	-1.479072000	-0.655712000
F	-1.082744000	0.000063000	1.123629000
F	1.082744000	-0.000063000	1.123629000

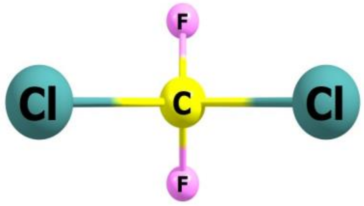

CH_3Br

C	0.000000000	0.000000000	-1.540232000
H	0.000000000	1.036877000	-1.871692000
H	0.897962000	-0.518439000	-1.871692000
H	-0.897962000	-0.518439000	-1.871692000
Br	0.000000000	0.000000000	0.424471000

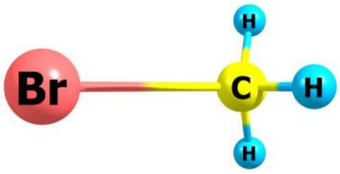

CH_3Cl

C	0.000000000	0.000000000	-1.139207000
H	0.000000000	1.033884000	-1.485431000
H	0.895369000	-0.516942000	-1.485431000
H	-0.895369000	-0.516942000	-1.485431000
Cl	0.000000000	0.000000000	0.664208000

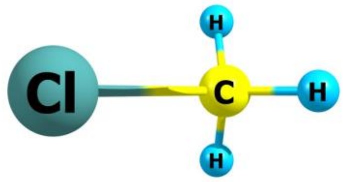

CH_4

C	0.000000000	0.000000000	0.000000000
H	0.631339000	0.631339000	0.631339000
H	-0.631339000	-0.631339000	0.631339000
H	-0.631339000	0.631339000	-0.631339000
H	0.631339000	-0.631339000	-0.631339000

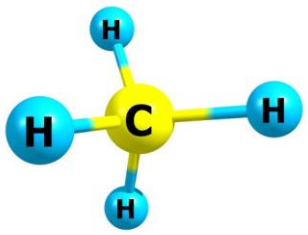

CO_2

C	0.000000000	0.000000000	-0.000083000
O	0.000000000	0.000000000	1.169178000
O	0.000000000	0.000000000	-1.169116000

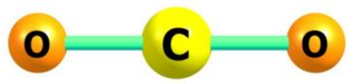

CO

C	0.000000000	0.000000000	-0.650252000
O	0.000000000	0.000000000	0.487689000

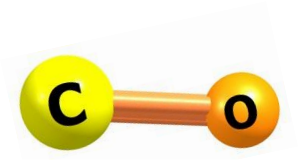

H_2S

S	0.000000000	0.000000000	0.103381000
H	0.000000000	0.977095000	-0.827050000
H	0.000000000	-0.977095000	-0.827050000

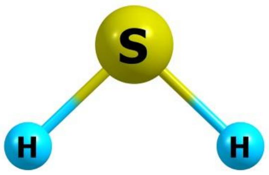

CH_3F

C	0.000000000	0.000000000	-0.630032000
H	0.000000000	1.032925000	-0.998065000
H	0.894540000	-0.516463000	-0.998065000
H	-0.894540000	-0.516463000	-0.998065000
F	0.000000000	0.000000000	0.752710000

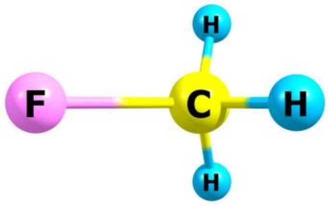

N_2O

O	0.000000000	0.000000000	0.599046000
N	0.000000000	-0.884567000	-0.342312000
N	0.000000000	0.884567000	-0.342312000

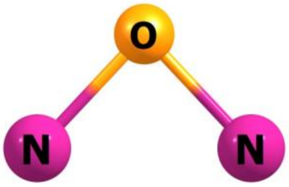

NF_3

N	0.000000000	0.000000000	0.487404000
F	0.000000000	1.240504000	-0.126364000
F	1.074308000	-0.620252000	-0.126364000
F	-1.074308000	-0.620252000	-0.126364000

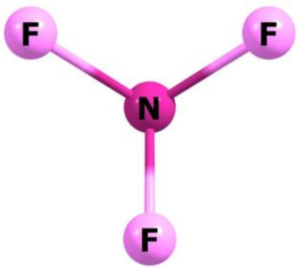

O_3

O	-0.719128000	-0.415187000	0.000000000
O	0.000000000	0.830376000	0.000000000
O	0.719128000	-0.415189000	0.000000000

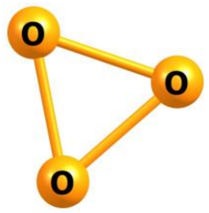

CF_4

C	0.000000000	0.000000000	0.000000000
F	-0.767523000	-0.767523000	0.767523000
F	0.767523000	0.767523000	0.767523000
F	-0.767523000	0.767523000	-0.767523000
F	0.767523000	-0.767523000	-0.767523000

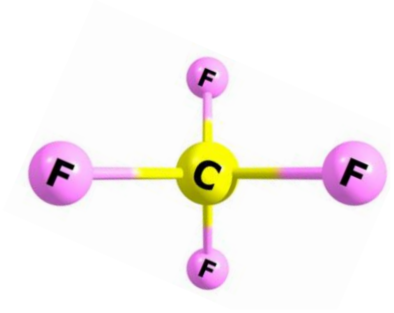

SF_6

S	0.000000000	0.000000000	0.000000000
F	0.000000000	0.000000000	1.600359000
F	0.000000000	1.600359000	0.000000000
F	1.600359000	0.000000000	0.000000000
F	0.000000000	0.000000000	-1.600359000
F	-1.600359000	0.000000000	0.000000000
F	0.000000000	-1.600359000	0.000000000

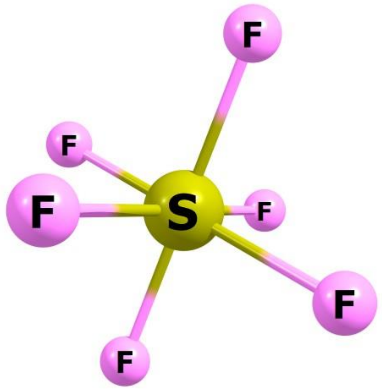

SO_2

S	0.000000000	0.000000000	0.370851000
O	0.000000000	-1.261655000	-0.370851000
O	0.000000000	1.261655000	-0.370851000

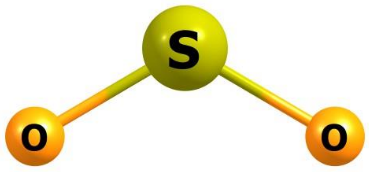

Table S18. Cartesian coordinates for optimized geometries of the empty 5^{12}, $5^{12}6^{2}$ and $5^{12}6^{4}$ clathrate hydrate monomers computes at B3LYP/6-31G(d) level of theory and basis set.

5^{12} Clathrate Hydate

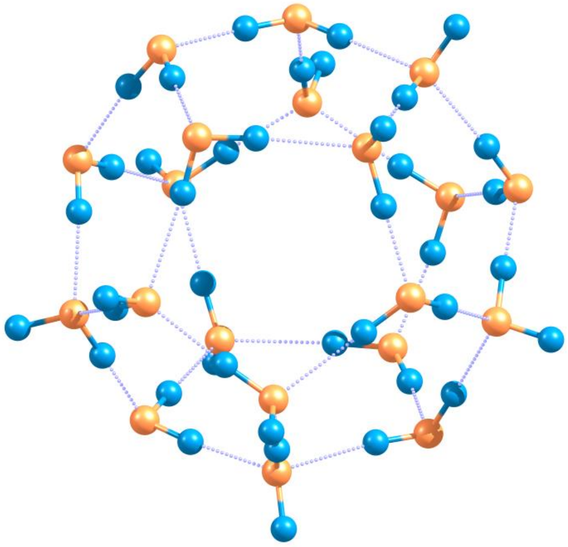

O	-0.807577000	3.763614000	-0.197525000
H	0.033759000	3.646888000	-0.739898000
H	-1.489303000	3.214763000	-0.641502000
O	-1.551322000	-3.222440000	1.739266000
H	-0.689809000	-3.295212000	1.284180000
H	-2.140813000	-2.797739000	1.075239000
O	1.413633000	3.377377000	-1.602252000
H	2.117106000	3.005161000	-1.018090000
H	1.275897000	2.699508000	-2.305179000
O	0.964264000	-3.657368000	0.371278000
H	0.821396000	-3.505735000	-0.596659000
H	1.034491000	-4.616986000	0.486410000
O	-2.569887000	0.928763000	2.787521000
H	-1.861318000	1.555542000	2.534554000
H	-2.085940000	0.129170000	3.126911000
O	3.749216000	-0.528969000	-0.549899000
H	3.468273000	-1.141628000	0.220329000
H	4.717658000	-0.566792000	-0.567274000
O	-3.871058000	0.315406000	0.542311000
H	-3.430683000	0.540243000	1.418013000
H	-3.530765000	0.983251000	-0.090431000
O	2.368082000	-1.079008000	-2.741412000
H	2.943499000	-0.882220000	-1.941778000

H	2.954137000	-1.057891000	-3.512858000
O	1.897263000	2.036300000	2.632716000
H	0.964568000	2.395613000	2.479076000
H	2.332087000	2.666011000	3.227134000
O	-1.780330000	0.621440000	-3.320016000
H	-1.856307000	-0.359643000	-3.057845000
H	-2.172595000	0.678524000	-4.204408000
O	1.709566000	-0.587100000	3.250137000
H	1.798972000	0.390552000	3.087626000
H	1.924094000	-0.724729000	4.185057000
O	-1.993027000	-1.894973000	-2.676399000
H	-2.464723000	-1.963222000	-1.804008000
H	-1.106040000	-2.276955000	-2.511180000
O	-0.547199000	2.868839000	2.169973000
H	-0.637031000	3.213235000	1.192339000
H	-0.875516000	3.597510000	2.718898000
O	-3.158561000	-2.025927000	-0.222592000
H	-3.997474000	-2.512237000	-0.220276000
H	-3.413782000	-1.077207000	0.095327000
O	0.962206000	1.340106000	-3.471994000
H	0.004323000	1.134069000	-3.425207000
H	1.396848000	0.541300000	-3.116549000
O	3.056606000	-2.107375000	1.381532000
H	2.344278000	-2.702614000	1.054572000
H	2.627175000	-1.575971000	2.094726000
O	0.601616000	-3.040756000	-2.275954000
H	0.688697000	-3.729709000	-2.952102000
H	1.280199000	-2.344464000	-2.502440000
O	3.265415000	2.218064000	0.129949000
H	3.383657000	1.280769000	-0.124790000

H	2.791634000	2.177104000	0.986113000
O	-1.097840000	-1.230126000	3.591618000
H	-1.315511000	-1.990870000	2.995632000
H	-0.174413000	-1.006531000	3.365918000
O	-2.819942000	2.147594000	-1.347338000
H	-2.474166000	1.614955000	-2.113274000
H	-3.515538000	2.716503000	-1.709670000

$5^{12}6^{2}$ Clathrate Hydate

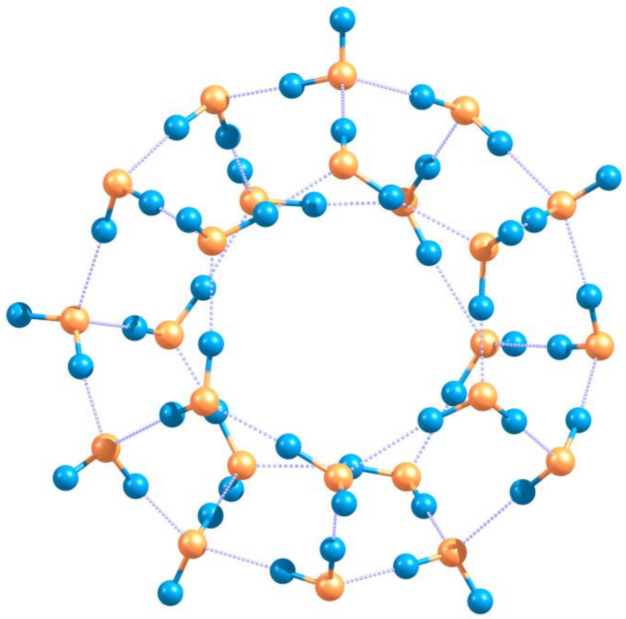

O	-0.345203000	2.481350000	-2.919374000
H	-1.096856000	1.809772000	-2.991477000
H	-0.321095000	2.922608000	-3.782394000
O	0.644304000	-2.757779000	-2.868436000
H	1.349687000	-2.073914000	-2.784163000
H	0.739074000	-3.325178000	-2.063525000
O	-0.817528000	4.308105000	-0.904330000
H	-0.656777000	3.683530000	-1.653866000
H	-1.566434000	3.917903000	-0.408712000
O	0.873225000	-4.244915000	-0.565567000
H	-0.008782000	-4.219572000	-0.048767000
H	1.038526000	-5.187042000	-0.722477000
O	-2.372808000	0.816525000	-3.124882000
H	-2.100343000	-0.134388000	-3.080825000
H	-2.961099000	0.945484000	-2.353360000
O	-1.783804000	-1.857683000	-2.943337000
H	-0.807581000	-2.154185000	-2.882220000
H	-2.109478000	-2.285936000	-3.750176000
O	-4.180995000	1.329050000	-0.954304000

H	-3.794204000	2.061844000	-0.399704000
H	-4.943414000	1.710572000	-1.415283000
O	-3.221704000	-3.086250000	-0.868769000
H	-2.702308000	-2.666710000	-1.591913000
H	-3.586242000	-2.334678000	-0.359936000
O	-3.012874000	3.214457000	0.574194000
H	-3.531310000	3.976918000	0.873336000
H	-2.631424000	2.793243000	1.407690000
O	-1.348417000	-4.205013000	0.767234000
H	-1.239652000	-3.541182000	1.486535000
H	-2.069582000	-3.848174000	0.183202000
O	2.314988000	1.535830000	-2.903515000
H	1.366880000	1.781941000	-2.843826000
H	2.768470000	2.051302000	-2.166945000
O	2.765969000	-0.966733000	-2.687684000
H	2.542147000	0.043656000	-2.732507000
H	3.260744000	-1.133769000	-3.504874000
O	3.429465000	2.874035000	-0.893150000
H	2.701398000	3.282784000	-0.380674000
H	3.848832000	2.236674000	-0.255026000
O	4.189227000	-1.426488000	-0.441844000
H	3.686385000	-1.267276000	-1.288637000
H	5.059819000	-1.757372000	-0.708255000
O	1.337992000	4.108803000	0.616923000
H	1.667923000	5.012160000	0.736538000
H	0.521521000	4.199255000	0.032977000
O	2.835198000	-3.294140000	1.204394000
H	2.149713000	-3.649729000	0.595013000
H	3.327205000	-2.642960000	0.659648000
O	-2.812927000	-0.359035000	2.903060000

H	-2.102181000	-1.054984000	2.913789000
H	-3.371893000	-0.581814000	2.126215000
O	2.489744000	0.703983000	2.924177000
H	1.822783000	1.459141000	2.927184000
H	2.980897000	0.803376000	3.753953000
O	-4.348306000	-0.899473000	0.616196000
H	-4.378049000	-0.106226000	0.025008000
H	-5.269562000	-1.147187000	0.785888000
O	4.419159000	1.060241000	0.889148000
H	4.389264000	0.194418000	0.429459000
H	3.743017000	0.970798000	1.596217000
O	-1.965176000	2.052194000	2.703428000
H	-2.251815000	1.063069000	2.758027000
H	-2.218160000	2.426989000	3.561596000
O	0.778503000	2.724046000	2.944513000
H	-0.130958000	2.391502000	2.791268000
H	0.990777000	3.260224000	2.142903000
O	-0.991221000	-2.369930000	2.858962000
H	0.011214000	-2.136992000	2.916461000
H	-1.145514000	-2.920341000	3.642525000
O	1.521136000	-1.916745000	3.120010000
H	1.807722000	-0.982781000	3.003007000
H	2.037147000	-2.442277000	2.444733000

$5^{12}6^4$ Clathrate Hydate

O	-1.008193000	-1.028248000	4.366167000
H	-0.064179000	-1.220972000	4.165992000
H	-1.521982000	-1.594714000	3.745554000
O	3.670766000	0.143197000	2.837518000
H	3.303834000	1.044057000	2.682586000
H	2.909471000	-0.396626000	3.134116000
O	2.724541000	2.731944000	2.389823000
H	3.240164000	3.299291000	2.983355000
H	1.751948000	2.936802000	2.625921000
O	0.298707000	3.391332000	3.021988000
H	-0.136079000	3.821393000	2.235958000
H	-0.293597000	2.647055000	3.266878000
O	3.228322000	3.469947000	-0.212173000
H	3.562578000	2.642155000	-0.636219000
H	3.060914000	3.222841000	0.731276000
O	4.121087000	1.135168000	-1.391287000
H	3.556967000	0.949849000	-2.218322000
H	5.013444000	1.292929000	-1.736141000
O	1.016926000	4.213470000	-1.301284000
H	1.170894000	5.155475000	-1.473753000
H	1.888055000	3.890255000	-0.860440000
O	-3.747874000	1.660469000	-1.947822000
H	-4.496074000	2.057309000	-2.418837000
H	-3.583106000	2.243697000	-1.154652000
O	-3.306617000	3.125338000	0.267466000
H	-3.897478000	3.883191000	0.398575000
H	-3.500682000	2.508167000	1.059876000
O	-0.853493000	4.447945000	0.801214000

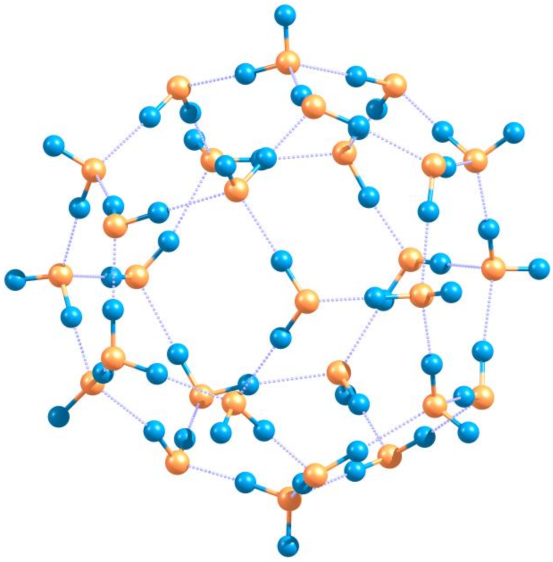

H	-0.233451000	4.296329000	0.054872000
H	-1.654692000	3.926704000	0.580879000
O	0.676480000	2.704828000	-3.504908000
H	0.771208000	3.287523000	-2.699294000
H	0.792590000	3.290877000	-4.267997000
O	-3.849721000	1.591069000	2.283364000
H	-4.003611000	0.666694000	1.977727000
H	-3.049597000	1.544279000	2.862899000
O	-4.279903000	-1.019844000	1.400415000
H	-4.267805000	-1.006395000	0.369648000
H	-5.196135000	-1.243763000	1.626011000
O	-1.588295000	1.478455000	3.856224000
H	-1.809642000	1.833214000	4.731089000
H	-1.326830000	0.514755000	4.028969000
O	1.737174000	-3.491705000	-2.304870000
H	2.171990000	-3.206501000	-1.474015000
H	0.850199000	-3.866981000	-2.026409000
O	3.222882000	-3.040166000	0.054609000
H	3.712871000	-2.141263000	0.213763000
H	3.920600000	-3.659257000	-0.210613000
O	4.515825000	-0.883281000	0.486222000
H	4.321156000	-0.190609000	-0.186920000
H	4.248698000	-0.488158000	1.363534000
O	2.742544000	0.806830000	-3.574225000
H	2.290467000	-0.067888000	-3.673891000
H	2.010987000	1.463732000	-3.560106000
O	1.830938000	-3.755448000	2.175368000
H	2.384379000	-3.507675000	1.368878000
H	2.206383000	-4.583893000	2.510000000
O	1.676858000	-1.624297000	3.837119000

H	1.747129000	-2.432867000	3.260810000
H	2.108223000	-1.862525000	4.672079000
O	-0.773570000	-4.465296000	1.221436000
H	0.122116000	-4.213649000	1.526033000
H	-1.349640000	-3.745812000	1.551792000
O	-2.449055000	-2.613185000	2.574819000
H	-3.131230000	-2.060991000	2.100310000
H	-2.949583000	-3.288976000	3.056851000
O	-0.672139000	-4.380658000	-1.546346000
H	-1.320907000	-3.685841000	-1.790181000
H	-0.734744000	-4.458770000	-0.562884000
O	-2.601760000	-2.521919000	-2.468960000
H	-3.111038000	-3.158089000	-2.995400000
H	-2.111406000	-1.960901000	-3.171706000
O	-4.393161000	-0.936851000	-1.159723000
H	-3.737536000	-1.527054000	-1.616774000
H	-4.172744000	-0.025176000	-1.459017000
O	-1.381083000	-1.179151000	-4.315951000
H	-1.554362000	-0.202004000	-4.262856000
H	-0.409855000	-1.282782000	-4.186173000
O	-1.832698000	1.502221000	-4.033734000
H	-2.411713000	1.577620000	-3.247099000
H	-0.979512000	1.908162000	-3.771103000
O	1.364328000	-1.539027000	-3.957665000
H	1.506339000	-2.284453000	-3.269764000
H	1.667072000	-1.924851000	-4.794110000

Table S19. Cartesian coordinates for optimized geometries of the Greenhouse gases encapsulated within 5^{12} clathrate hydrate computes at B3LYP/6-31G(d) level of theory and basis set.

<u>CCl_4 within 5^{12} clathrate hydrate</u>

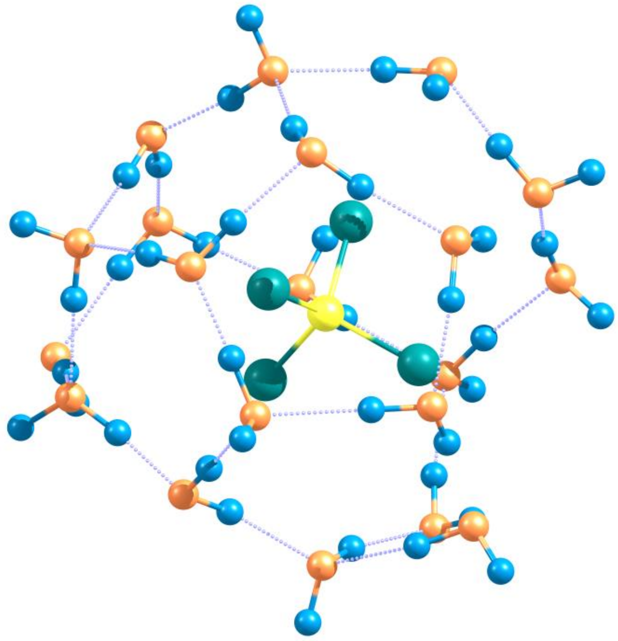

O	3.103269000	0.310240000	-2.405422000
H	2.480067000	1.044335000	-2.648012000
H	3.400336000	0.495119000	-1.491438000
O	-0.967257000	-2.351909000	3.212136000
H	-1.694701000	-1.705288000	3.130024000
H	-0.158906000	-1.808785000	3.342075000
O	1.530894000	2.498914000	-2.968483000
H	0.555389000	2.549604000	-3.028430000
H	1.778075000	3.161595000	-2.285891000
O	-3.176329000	-0.294228000	3.148679000
H	-2.628980000	0.511710000	3.306115000
H	-3.661314000	-0.443828000	3.974849000
O	2.317149000	-3.416945000	0.206486000
H	2.386051000	-2.925906000	-0.641269000
H	1.353483000	-3.425064000	0.436105000
O	-3.514751000	1.505088000	-1.646502000
H	-3.596064000	0.528458000	-1.834496000
H	-4.288361000	1.904628000	-2.073958000
O	3.301513000	-1.579292000	1.959671000
H	3.018749000	-2.317387000	1.349035000
H	3.691880000	-0.884223000	1.388245000
O	-3.837730000	1.993065000	1.191011000
H	-3.657533000	1.843286000	0.234062000
H	-3.997447000	1.105139000	1.558949000

O	0.309163000	-3.941098000	-2.321474000
H	0.974513000	-3.236087000	-2.510099000
H	0.644696000	-4.340582000	-1.500551000
O	2.768149000	2.726730000	1.450844000
H	2.030316000	2.391771000	2.062507000
H	3.313775000	3.293181000	2.017972000
O	-2.132141000	-3.206479000	-1.546127000
H	-1.226889000	-3.373315000	-1.946586000
H	-2.581179000	-4.061619000	-1.635060000
O	0.992762000	1.903809000	3.197552000
H	1.130773000	0.942427000	3.383701000
H	0.040540000	1.993292000	2.983771000
O	2.446568000	-2.227128000	-2.378383000
H	2.595590000	-1.225768000	-2.418896000
H	3.195325000	-2.608322000	-2.860899000
O	1.400029000	-0.804704000	3.622458000
H	1.746437000	-1.041842000	4.496357000
H	2.114063000	-1.105303000	2.972242000
O	2.192603000	4.244589000	-0.843720000
H	2.319838000	3.688869000	-0.041548000
H	1.417292000	4.790339000	-0.647744000
O	-3.929197000	-1.156457000	-2.124435000
H	-4.565094000	-1.442237000	-1.451384000
H	-3.185787000	-1.802495000	-2.021421000
O	-1.865181000	2.144940000	3.001434000
H	-2.113409000	2.858146000	3.609579000
H	-2.463009000	2.258049000	2.207387000
O	-1.410368000	2.773949000	-3.312121000
H	-2.017603000	2.323798000	-2.687979000
H	-1.517191000	2.284530000	-4.141465000

O	-0.381057000	-3.654875000	0.815400000
H	-0.625009000	-3.242905000	1.681684000
H	-1.066935000	-3.364222000	0.186627000
O	4.104628000	0.618918000	0.305826000
H	3.653921000	1.392617000	0.734790000
H	5.039460000	0.864624000	0.234240000
C	-0.400148000	0.235345000	-0.385797000
Cl	-1.797924000	-0.471637000	0.477382000
Cl	-0.459297000	-0.228966000	-2.115112000
Cl	-0.446156000	2.024947000	-0.223438000
Cl	1.088319000	-0.381956000	0.353693000

CF_2Cl_2 within 5^{12} clathrate hydrate

O	-1.98606200	-1.02345900	-3.29441500
H	-1.01790500	-1.30276900	-3.27917300
H	-2.30698200	-1.30278600	-4.16558100
O	2.40145300	2.19044500	-2.72047900
H	1.45100200	2.42566200	-2.62004000
H	2.82729100	2.44381100	-1.86643900
O	-3.39401200	-2.41353300	-1.31565900
H	-2.90459000	-1.90866100	-2.00856500
H	-2.71200200	-2.92943300	-0.83931500
O	3.55255300	2.79523200	-0.25052700
H	3.91777600	1.97310600	0.22550800
H	4.29645100	3.41542400	-0.28942900
O	0.48875000	-1.95434500	-3.32014500
H	1.26111000	-1.34202300	-3.25174800
H	0.58581500	-2.58488300	-2.57867500

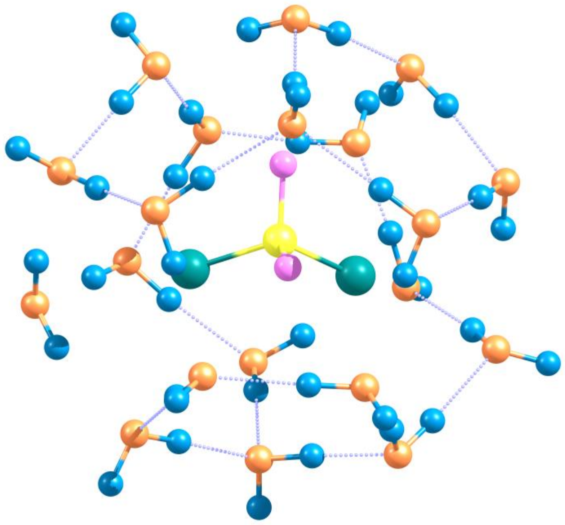

O	2.77054500	-0.38030600	-3.11845600
H	2.59949300	0.60757400	-2.96417900
H	3.27675300	-0.41844100	-3.94444800
O	0.68305900	-4.07957700	-1.39854900
H	-0.15244000	-4.15488100	-0.86273000
H	0.70190500	-4.84897700	-1.98720400
O	4.34232900	-1.31811100	-0.93557000
H	3.83510800	-0.97919800	-1.70613400
H	3.75779900	-1.99337300	-0.53530500
O	-1.57114600	-4.12871700	0.10099200
H	-2.08061900	-4.94072900	0.24295400
H	-1.39163700	-3.74661100	1.01315400
O	4.45078700	0.66277300	0.96420800
H	3.81434400	0.37194600	1.65652800
H	4.47730800	-0.07404300	0.29874300
O	-2.42449100	1.77229600	-3.09103600
H	-2.24415500	0.81012600	-3.17757800
H	-3.05791900	1.83504100	-2.32539200
O	-0.26949100	3.11863800	-2.55560700
H	-1.09343000	2.53656900	-2.73863100
H	-0.27199500	3.76305400	-3.28054700
O	-4.02587300	1.84246200	-0.88227200
H	-4.27954700	0.99422900	-0.46235700
H	-3.71874400	2.41705200	-0.12983100
O	-0.61583200	4.27028600	-0.11601400
H	-0.50028900	3.86318800	-1.01759900
H	-0.68442500	5.22528000	-0.26379400
O	-4.47036700	-0.69390200	0.40923900
H	-5.40228400	-0.94725400	0.49062400
H	-4.07722000	-1.33804100	-0.25567600

O	1.61674800	3.77954100	1.56803800
H	2.30470300	3.44395000	0.95240700
H	0.83300200	3.93361200	0.99884300
O	1.49308300	-2.66109700	2.70430200
H	1.82208700	-1.74182800	2.87387100
H	1.94263500	-2.92430900	1.87138900
O	-1.85989200	1.57917500	3.13112200
H	-2.25396900	0.66511200	3.00606900
H	-2.19754200	1.88190300	3.98782600
O	2.73100600	-3.38111600	0.27282400
H	2.03428300	-3.68747500	-0.35952000
H	3.34605500	-4.12330600	0.37340400
O	-2.97017700	3.31453700	1.15714200
H	-2.16201100	3.67775800	0.73641500
H	-2.62177100	2.70152400	1.84174300
O	-1.08062400	-3.03142200	2.48165000
H	-0.08602200	-2.81162100	2.55793500
H	-1.27714700	-3.55922500	3.27096300
O	-2.97784500	-0.83971700	2.79714900
H	-2.29069600	-1.51730600	2.63365900
H	-3.51771700	-0.80038500	1.97321600
O	2.61526700	-0.14644900	3.01153700
H	1.93866800	0.61133600	3.11743700
H	3.13539700	-0.11355200	3.82947500
O	0.98616900	1.85215500	3.37744400
H	0.02664800	1.68853500	3.25154600
H	1.21680400	2.58581400	2.74340000
C	-0.11628600	-0.37805300	0.38443100
Cl	-1.49472000	0.54596200	-0.20904300
Cl	1.43078900	0.28105000	-0.15815900

F	-0.21648200	-1.65668200	-0.04747000
F	-0.13933100	-0.40750500	1.72339200

<u>CH_3Br within 5^{12} clathrate hydrate</u>

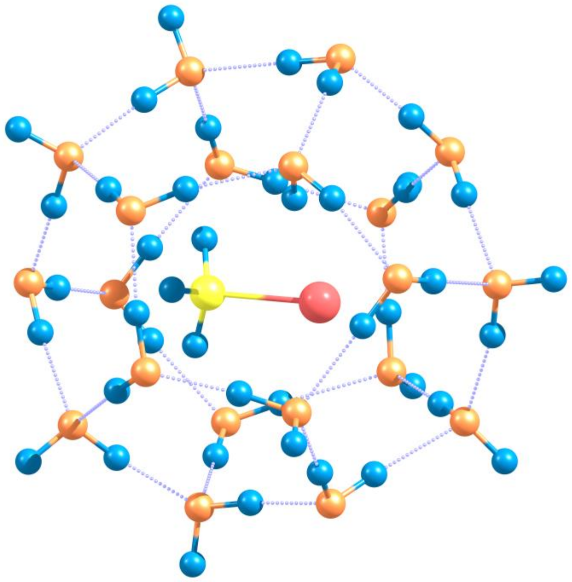

O	-4.110479000	-0.525289000	-0.063101000
H	-3.869064000	-0.368628000	-1.024868000
H	-3.665287000	-1.362099000	0.182354000
O	2.906064000	0.092000000	2.892245000
H	3.282394000	0.296777000	2.012870000
H	2.449260000	-0.770063000	2.757542000
O	-3.303471000	-0.062310000	-2.585361000
H	-2.865868000	0.821437000	-2.567858000
H	-2.593103000	-0.684827000	-2.860375000
O	3.888987000	0.723714000	0.189373000
H	3.816951000	-0.025525000	-0.450953000
H	4.825708000	0.971662000	0.210475000
O	-1.675172000	0.371724000	3.494764000
H	-2.304068000	0.771352000	2.856975000
H	-0.848555000	0.901057000	3.420545000
O	0.883778000	2.090918000	-2.754158000
H	1.434984000	2.408436000	-1.947374000
H	1.064356000	2.746993000	-3.445548000
O	-0.996997000	-2.177809000	2.921600000
H	-1.292194000	-1.264713000	3.190054000
H	-1.608818000	-2.454835000	2.207560000
O	1.473261000	-0.447058000	-3.339087000
H	1.261735000	0.510736000	-3.120221000
H	1.626691000	-0.466690000	-4.296611000

O	-2.044169000	3.400239000	0.301790000
H	-2.588879000	2.718613000	0.783326000
H	-2.540047000	4.229893000	0.372683000
O	-0.672551000	-3.632268000	-1.045127000
H	0.267206000	-3.469982000	-0.689523000
H	-0.663980000	-4.553655000	-1.346327000
O	0.675753000	3.901607000	1.119137000
H	-0.253166000	3.739443000	0.849701000
H	0.792826000	3.335757000	1.913117000
O	1.773588000	-3.344759000	-0.150207000
H	1.729237000	-3.008802000	0.781229000
H	2.271368000	-2.652068000	-0.632106000
O	-3.456822000	1.430554000	1.529690000
H	-3.702446000	0.672270000	0.888714000
H	-4.290547000	1.671642000	1.961702000
O	1.555411000	-2.341122000	2.404491000
H	1.806668000	-2.981840000	3.088177000
H	0.557414000	-2.211852000	2.548274000
O	-1.104897000	-1.785058000	-3.166480000
H	-0.977193000	-2.494159000	-2.501591000
H	-0.248696000	-1.311197000	-3.180737000
O	2.342071000	2.976301000	-0.815487000
H	2.842690000	2.237465000	-0.412929000
H	1.755068000	3.336302000	-0.091415000
O	3.357270000	-1.358267000	-1.592591000
H	4.041313000	-1.827303000	-2.094688000
H	2.704447000	-1.020718000	-2.265326000
O	-1.919748000	2.363117000	-2.334705000
H	-0.951944000	2.263595000	-2.460745000
H	-2.012229000	2.808485000	-1.466316000

O	0.722838000	1.682643000	2.817674000
H	1.563921000	1.215703000	3.085667000
H	0.613999000	1.311058000	1.922533000
O	-2.636244000	-2.907047000	0.717117000
H	-1.952782000	-3.220850000	0.067219000
H	-3.205709000	-3.670911000	0.893867000
C	-1.075465000	-0.168904000	-0.171174000
Br	0.886972000	-0.033836000	-0.052720000
H	-1.426027000	0.782695000	-0.555487000
H	-1.288275000	-0.961731000	-0.878027000
H	-1.418360000	-0.385860000	0.834053000

CH_3Cl within 5^{12} clathrate hydrate

O	-3.954632000	0.203912000	-0.636027000
H	-3.532072000	0.548838000	-1.479662000
H	-3.672031000	-0.731674000	-0.562713000
O	2.419150000	-1.169587000	2.975045000
H	2.866508000	-0.760982000	2.209300000
H	1.852671000	-1.868749000	2.578236000
O	-2.697172000	1.126391000	-2.815714000
H	-2.138334000	1.891978000	-2.546600000
H	-2.056441000	0.461693000	-3.161292000
O	3.952254000	0.010963000	0.699063000
H	3.805257000	-0.571812000	-0.085669000
H	4.872545000	-0.126106000	0.969106000
O	-1.984683000	-0.293661000	3.212141000
H	-2.445886000	0.370937000	2.663167000
H	-1.082429000	0.084374000	3.403184000

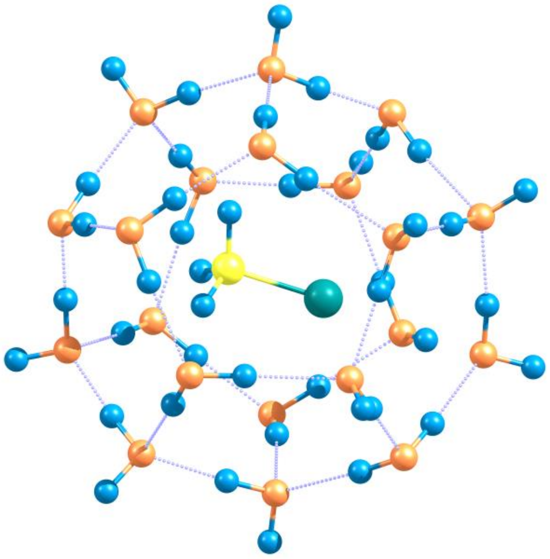

O	1.777211000	2.574154000	-2.087520000
H	2.255494000	2.582690000	-1.186624000
H	2.145946000	3.326342000	-2.575755000
O	-1.735495000	-2.755271000	2.168540000
H	-1.872156000	-1.865526000	2.602154000
H	-2.227791000	-2.701611000	1.323043000
O	1.961051000	0.140894000	-3.202646000
H	1.895157000	1.052386000	-2.791942000
H	2.247408000	0.285265000	-4.117478000
O	-1.549292000	3.511932000	0.931561000
H	-2.237918000	2.814622000	1.151214000
H	-1.990730000	4.366391000	1.049517000
O	-0.976747000	-3.112512000	-2.041558000
H	-0.089605000	-3.232165000	-1.551301000
H	-1.060069000	-3.898955000	-2.602051000
O	0.942411000	3.071811000	2.020187000
H	0.043138000	3.260107000	1.652006000
H	1.050901000	3.667628000	2.776322000
O	1.299987000	-3.483279000	-0.827292000
H	1.147179000	-3.421077000	0.151844000
H	1.947416000	-2.774249000	-1.024814000
O	-3.317116000	1.582927000	1.457260000
H	-3.559841000	1.039623000	0.619918000
H	-4.166201000	1.822192000	1.858794000
O	0.806780000	-3.209871000	1.843614000
H	0.936850000	-4.009659000	2.375785000
H	-0.182756000	-2.988330000	1.956080000
O	-0.765178000	-0.757975000	-3.618347000
H	-0.850587000	-1.602755000	-3.128094000
H	0.137910000	-0.443260000	-3.413556000

O	3.036477000	2.662530000	0.199973000
H	3.380682000	1.764197000	0.403383000
H	2.331884000	2.819422000	0.870491000
O	3.319421000	-1.527937000	-1.528793000
H	3.983002000	-1.991467000	-2.062014000
H	2.844180000	-0.918154000	-2.155470000
O	-1.017634000	3.164354000	-1.858355000
H	-0.065882000	2.933812000	-1.905378000
H	-1.192459000	3.334615000	-0.910463000
O	0.501786000	0.722292000	3.658340000
H	1.191726000	0.032751000	3.484565000
H	0.685153000	1.417925000	2.998003000
O	-3.038242000	-2.515304000	-0.358569000
H	-2.324146000	-2.783722000	-0.995794000
H	-3.757334000	-3.153470000	-0.479005000
C	-0.859126000	0.030936000	-0.174088000
H	-1.127987000	1.037281000	-0.481450000
H	-1.078437000	-0.661641000	-0.980823000
H	-1.332420000	-0.259780000	0.759989000
Cl	0.925563000	0.005315000	0.109361000

CH_4 within 5^{12} clathrate hydrate

O	-1.721426000	2.697216000	-2.144278000
H	-0.912505000	2.378137000	-2.647768000
H	-2.285592000	1.902117000	-2.030152000
O	-0.627081000	-1.628749000	3.516898000
H	0.186981000	-1.862906000	3.030284000
H	-1.353159000	-1.803479000	2.876198000

O	0.447373000	1.793087000	-3.390179000
H	1.277088000	1.975498000	-2.886485000
H	0.444743000	0.821151000	-3.559645000
O	1.814914000	-2.504504000	2.235067000
H	1.575745000	-3.001603000	1.413179000
H	2.104059000	-3.167670000	2.879609000
O	-2.468178000	2.042408000	2.108075000
H	-1.980317000	2.567958000	1.441226000
H	-1.776371000	1.728942000	2.750430000
O	3.751264000	-0.236567000	-0.898014000
H	3.649569000	-0.270352000	0.116693000
H	4.703058000	-0.154209000	-1.061956000
O	-3.797152000	0.013626000	0.984378000
H	-3.338620000	0.783705000	1.434532000
H	-3.680300000	0.179310000	0.024739000
O	2.375934000	-2.225051000	-1.998234000
H	2.939613000	-1.491772000	-1.609001000
H	2.878993000	-2.592808000	-2.740254000
O	1.539314000	3.493024000	0.522169000
H	0.543119000	3.566216000	0.374968000
H	1.878670000	4.400444000	0.503072000
O	-2.121152000	-1.814387000	-2.667429000
H	-1.937400000	-2.411650000	-1.867421000
H	-2.577335000	-2.378409000	-3.310064000
O	2.071938000	1.830008000	2.588082000
H	1.888359000	2.490517000	1.867305000
H	2.418057000	2.337045000	3.337643000
O	-1.659123000	-3.366415000	-0.615095000
H	-2.045415000	-2.971383000	0.211612000
H	-0.702770000	-3.470070000	-0.429570000

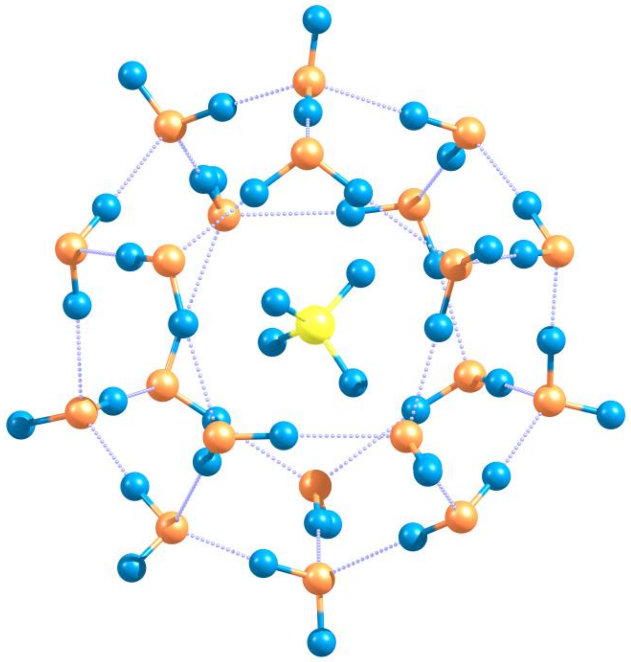

O	-1.067363000	3.592336000	0.153287000
H	-1.319437000	3.207348000	-0.776826000
H	-1.489442000	4.464532000	0.184191000
O	-2.630985000	-2.183526000	1.628504000
H	-3.329958000	-2.680710000	2.080480000
H	-3.074936000	-1.290169000	1.368923000
O	0.392220000	-0.979041000	-3.692958000
H	-0.493671000	-1.244824000	-3.366221000
H	1.012261000	-1.332846000	-3.026668000
O	3.496336000	-0.351939000	1.679976000
H	2.929984000	-1.117033000	1.929638000
H	3.029939000	0.443332000	2.033536000
O	1.142164000	-3.713675000	-0.135755000
H	1.350440000	-4.648755000	-0.283016000
H	1.623562000	-3.208967000	-0.849584000
O	2.670421000	2.265099000	-1.798616000
H	3.000578000	1.395164000	-1.495437000
H	2.270293000	2.668771000	-1.000881000
O	-0.494755000	1.115247000	3.738855000
H	-0.571531000	0.127262000	3.736302000
H	0.354886000	1.294489000	3.292084000
O	-3.366108000	0.426624000	-1.783361000
H	-2.942501000	-0.394856000	-2.149688000
H	-4.188104000	0.548389000	-2.281509000
C	-0.004472000	0.010745000	0.022634000
H	0.980282000	-0.094505000	0.486907000
H	0.087081000	0.573873000	-0.910145000
H	-0.415301000	-0.980410000	-0.188852000
H	-0.674316000	0.532143000	0.710804000

CO_2 within 5^{12} clathrate hydrate

O	-2.360130000	0.572211000	-3.037786000
H	-1.374549000	0.423275000	-3.071131000
H	-2.693648000	-0.232275000	-2.582517000
O	-0.422532000	-0.157880000	3.822404000
H	0.495277000	-0.206095000	3.489464000
H	-0.914347000	-0.848566000	3.323084000
O	0.267839000	0.185282000	-2.714625000
H	0.891419000	0.934806000	-2.883841000
H	0.720497000	-0.641317000	-3.012329000
O	2.348458000	-0.364991000	3.008987000
H	2.456406000	-1.175186000	2.451996000
H	2.794411000	-0.552203000	3.848654000
O	-3.598617000	1.503073000	1.126764000
H	-3.221053000	1.807166000	0.276945000
H	-2.896606000	1.732584000	1.790182000
O	3.714663000	1.069702000	-0.870939000
H	3.475716000	1.369559000	0.074687000
H	4.592228000	1.445674000	-1.040042000
O	-3.747512000	-1.175510000	1.010805000
H	-3.734429000	-0.172658000	1.070435000
H	-3.563802000	-1.371732000	0.068038000
O	3.473543000	-1.604691000	-1.028742000
H	3.537588000	-0.604785000	-0.997046000
H	4.305739000	-1.908099000	-1.422118000
O	-0.126653000	3.686383000	-1.182094000
H	-1.031408000	3.251059000	-1.286363000
H	-0.227133000	4.586876000	-1.525352000
O	-0.793017000	-3.311878000	-1.670877000

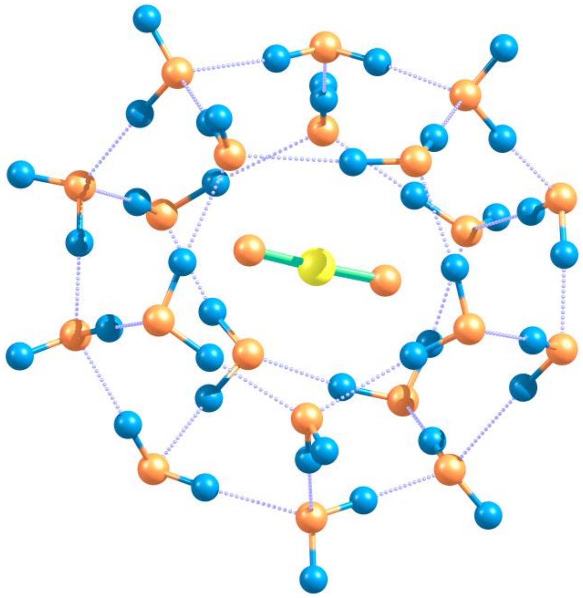

H	-0.502060000	-3.372523000	-0.699355000
H	-0.926488000	-4.232435000	-1.944856000
O	0.726622000	3.241047000	1.340715000
H	0.415899000	3.488757000	0.429386000
H	0.647594000	4.042256000	1.880397000
O	-0.052439000	-3.507995000	0.818931000
H	-0.694580000	-3.036621000	1.412675000
H	0.829836000	-3.127981000	1.013092000
O	-2.507138000	2.533411000	-1.369536000
H	-2.463888000	1.735013000	-2.019043000
H	-3.221212000	3.093600000	-1.710744000
O	-1.788082000	-2.121197000	2.375169000
H	-2.236962000	-2.672450000	3.034537000
H	-2.555769000	-1.709177000	1.818375000
O	1.475406000	-2.255411000	-3.027166000
H	0.711578000	-2.680373000	-2.579237000
H	2.115438000	-2.072817000	-2.310045000
O	3.150368000	1.886407000	1.537084000
H	2.899573000	1.108792000	2.085361000
H	2.318300000	2.410544000	1.468854000
O	2.582076000	-2.544365000	1.342968000
H	3.117237000	-3.319800000	1.570260000
H	2.939158000	-2.207643000	0.474769000
O	1.925970000	2.354574000	-2.694641000
H	2.533120000	1.957121000	-2.034372000
H	1.267055000	2.851078000	-2.166256000
O	-1.515900000	2.148769000	2.801773000
H	-1.170158000	1.344963000	3.265641000
H	-0.779123000	2.411719000	2.218466000
O	-3.098478000	-1.815101000	-1.702147000

H	-2.264318000	-2.353651000	-1.713825000
H	-3.759947000	-2.344499000	-2.172004000
C	-0.001388000	-0.075767000	0.017016000
O	1.152506000	-0.153913000	0.202897000
O	-1.159985000	-0.005096000	-0.089560000

CO within 5^{12} clathrate hydrate

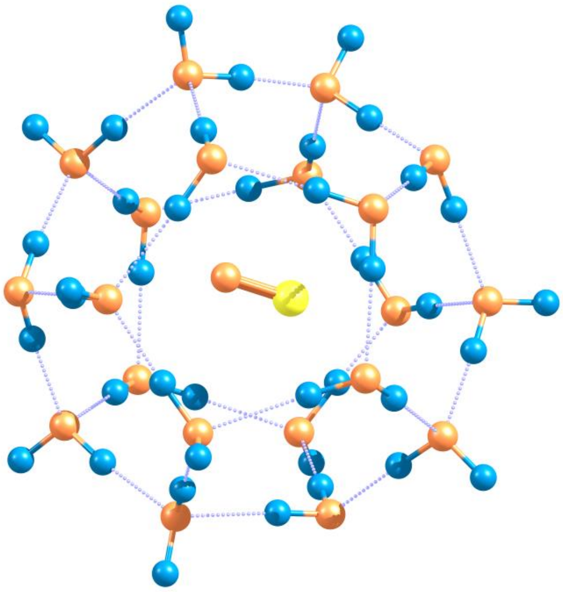

O	-1.856366000	2.748389000	-1.847231000
H	-1.165747000	2.374381000	-2.476751000
H	-2.479297000	2.012428000	-1.662666000
O	-0.174283000	-1.592024000	3.640379000
H	0.544057000	-1.876688000	3.042261000
H	-0.993255000	-1.705169000	3.106273000
O	0.011058000	1.723538000	-3.420397000
H	0.919727000	1.853147000	-3.055600000
H	-0.086817000	0.752055000	-3.557537000
O	1.965999000	-2.597612000	1.979714000
H	1.564945000	-3.084074000	1.216548000
H	2.330500000	-3.274415000	2.569675000
O	-1.980360000	2.206334000	2.462857000
H	-1.553331000	2.672999000	1.715799000
H	-1.227304000	1.829544000	2.991786000
O	3.511389000	-0.509155000	-1.431174000
H	3.584448000	-0.531286000	-0.409945000
H	4.425465000	-0.494138000	-1.753647000
O	-3.562455000	0.272514000	1.542739000
H	-2.999093000	1.018550000	1.911290000
H	-3.614122000	0.434989000	0.576834000
O	1.868159000	-2.399506000	-2.285578000

H	2.528016000	-1.699135000	-1.992991000
H	2.235249000	-2.803574000	-3.086170000
O	1.839624000	3.394059000	0.222915000
H	0.839332000	3.525861000	0.236182000
H	2.226419000	4.279865000	0.155862000
O	-2.688091000	-1.694061000	-2.267623000
H	-2.406673000	-2.285625000	-1.491067000
H	-3.271456000	-2.245782000	-2.810418000
O	2.539973000	1.667173000	2.185550000
H	2.309469000	2.346379000	1.497173000
H	2.991550000	2.146191000	2.896558000
O	-1.975615000	-3.256966000	-0.300210000
H	-2.191666000	-2.823465000	0.567719000
H	-1.006550000	-3.395699000	-0.275803000
O	-0.783244000	3.618658000	0.287288000
H	-1.216653000	3.250643000	-0.582078000
H	-1.155729000	4.506842000	0.397934000
O	-2.446826000	-1.974996000	2.044540000
H	-3.099644000	-2.417896000	2.608430000
H	-2.871589000	-1.057718000	1.831453000
O	-0.295977000	-1.034087000	-3.617135000
H	-1.144242000	-1.253574000	-3.174753000
H	0.393592000	-1.464855000	-3.076961000
O	3.727895000	-0.596340000	1.144539000
H	3.136892000	-1.308902000	1.477761000
H	3.357868000	0.234210000	1.529630000
O	0.843198000	-3.768167000	-0.229411000
H	0.927525000	-4.722558000	-0.375576000
H	1.255562000	-3.327179000	-1.024218000
O	2.487611000	2.049655000	-2.212971000

H	2.798143000	1.164487000	-1.933967000
H	2.263111000	2.506935000	-1.376604000
O	0.171058000	1.138472000	3.758593000
H	0.030226000	0.159232000	3.811074000
H	0.923578000	1.242236000	3.144669000
O	-3.642433000	0.644709000	-1.261162000
H	-3.318741000	-0.206048000	-1.659983000
H	-4.526900000	0.794414000	-1.626995000
C	-0.451616000	0.100220000	-0.420250000
O	0.621290000	0.243297000	-0.066182000

H_2S within 5^{12} clathrate hydrate

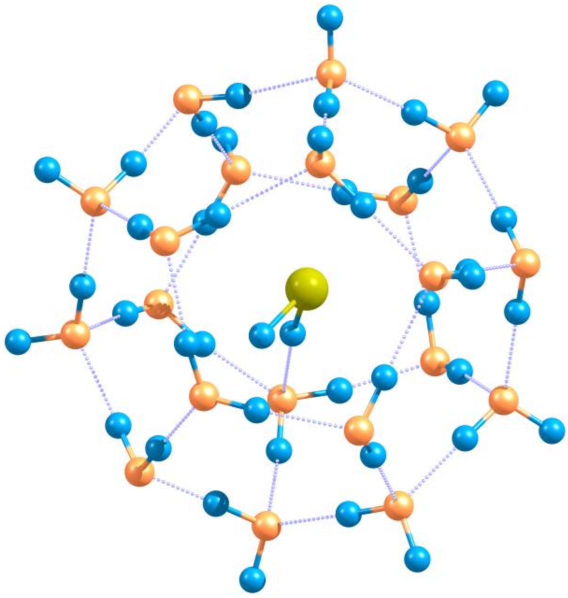

O	1.658986000	2.950816000	2.063290000
H	1.029459000	2.368540000	2.568120000
H	2.331286000	2.322753000	1.716143000
O	0.257562000	-1.063971000	-3.634706000
H	-0.369919000	-1.579111000	-3.089431000
H	1.119236000	-1.134733000	-3.166503000
O	-0.094243000	1.214560000	3.101167000
H	-1.078056000	1.301396000	3.020273000
H	0.105900000	0.301763000	3.429409000
O	-1.625644000	-2.670145000	-2.165994000
H	-1.129855000	-3.119093000	-1.436131000
H	-1.893644000	-3.370052000	-2.779931000
O	1.287519000	2.238784000	-1.847094000
H	0.964052000	2.981424000	-1.295873000
H	0.618783000	2.088892000	-2.578278000
O	-3.388507000	-1.123514000	1.438422000
H	-3.447829000	-1.057563000	0.417495000

H	-4.293775000	-1.311885000	1.730074000
O	3.658788000	0.972197000	-1.621218000
H	2.875865000	1.550903000	-1.808140000
H	3.716551000	0.990504000	-0.640947000
O	-1.474525000	-2.838788000	2.142889000
H	-2.209331000	-2.201904000	1.892211000
H	-1.844159000	-3.417050000	2.827048000
O	-2.285636000	3.157966000	0.206941000
H	-1.329099000	3.458670000	0.200533000
H	-2.808317000	3.956398000	0.375326000
O	2.883989000	-1.451157000	2.002703000
H	2.683295000	-2.005488000	1.170515000
H	3.581243000	-1.944238000	2.461974000
O	-2.915559000	1.576146000	-1.948068000
H	-2.672447000	2.149338000	-1.176352000
H	-3.608026000	2.057552000	-2.425286000
O	2.449703000	-2.957374000	-0.085656000
H	2.582268000	-2.400431000	-0.894812000
H	1.492913000	-3.171061000	-0.094267000
O	0.274104000	3.937515000	0.130914000
H	0.829542000	3.533042000	0.900111000
H	0.466341000	4.887086000	0.154298000
O	2.740026000	-1.373047000	-2.311732000
H	3.377847000	-1.692469000	-2.968085000
H	3.095345000	-0.460714000	-2.028081000
O	0.550467000	-1.397081000	3.614788000
H	1.366134000	-1.424723000	3.066949000
H	-0.118039000	-1.866547000	3.076729000
O	-3.630105000	-0.999295000	-1.139684000
H	-2.936165000	-1.580510000	-1.526667000

H	-3.372221000	-0.089642000	-1.420519000
O	-0.282345000	-3.837573000	-0.072450000
H	-0.242106000	-4.800768000	0.026314000
H	-0.715131000	-3.494613000	0.756072000
O	-2.744808000	1.457534000	2.472798000
H	-2.951796000	0.573558000	2.101338000
H	-2.589916000	2.019521000	1.684930000
O	-0.568944000	1.553983000	-3.636414000
H	-0.283762000	0.607031000	-3.727094000
H	-1.376102000	1.509411000	-3.084721000
O	3.574018000	1.048238000	1.203319000
H	3.308888000	0.140452000	1.518863000
H	4.408552000	1.247174000	1.653848000
S	-0.112730000	-0.149640000	-0.022286000
H	-0.005423000	0.484021000	1.177921000
H	0.526526000	0.821061000	-0.742506000

CH_3F within 5^{12} clathrate hydrate

O	-0.949519000	2.464052000	2.666180000
H	-0.895165000	1.591117000	3.158880000
H	-0.045476000	2.618128000	2.316988000
O	0.513924000	0.135019000	-3.677919000
H	0.554052000	-0.756022000	-3.276515000
H	1.220391000	0.643028000	-3.218886000
O	-0.728605000	0.075608000	3.815906000
H	-1.420543000	-0.528954000	3.445601000
H	0.124084000	-0.312699000	3.528628000
O	0.675704000	-2.501169000	-2.592211000
H	1.432335000	-2.533037000	-1.943051000

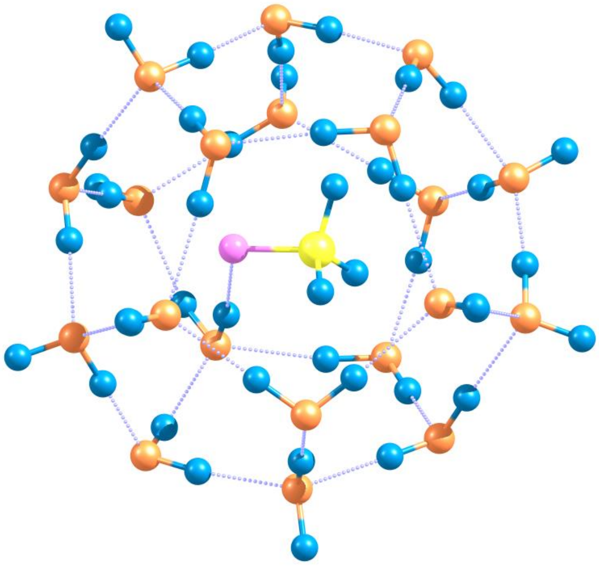

H	0.951047000	-3.042019000	-3.347527000
O	-1.357777000	3.288883000	-1.506948000
H	-1.875553000	3.059452000	-0.709088000
H	-1.608218000	2.603440000	-2.183529000
O	-1.121981000	-3.482394000	1.108081000
H	-1.380153000	-3.361217000	0.138904000
H	-1.504445000	-4.334323000	1.369126000
O	1.300502000	3.444154000	-1.081589000
H	0.318262000	3.432058000	-1.260718000
H	1.392920000	3.304512000	-0.115715000
O	1.644248000	-3.443950000	1.441151000
H	0.666519000	-3.527672000	1.329928000
H	1.747309000	-2.673121000	2.046181000
O	-3.916921000	0.138818000	0.623452000
H	-3.540371000	1.065648000	0.734121000
H	-4.813130000	0.167307000	0.991261000
O	3.506940000	0.941461000	1.867967000
H	3.662793000	0.563786000	0.926515000
H	4.388683000	1.130860000	2.222332000
O	-3.363060000	-0.882632000	-1.834039000
H	-3.665509000	-0.501975000	-0.968809000
H	-4.158448000	-0.989150000	-2.377242000
O	3.887495000	-0.038037000	-0.496792000
H	3.415927000	0.532060000	-1.157215000
H	3.450154000	-0.915710000	-0.572618000
O	-2.775711000	2.518648000	0.870713000
H	-2.034210000	2.487860000	1.587870000
H	-3.332078000	3.273849000	1.115994000
O	2.518735000	1.513805000	-2.296162000
H	3.086235000	1.973418000	-2.933818000

H	2.018414000	2.261593000	-1.809325000
O	1.610471000	-0.911185000	2.521589000
H	2.411629000	-0.340987000	2.584923000
H	1.289689000	-0.688890000	1.625533000
O	-1.754795000	-3.145255000	-1.422016000
H	-0.921256000	-2.942178000	-1.906407000
H	-2.359321000	-2.390217000	-1.601008000
O	2.688403000	-2.593754000	-0.787067000
H	3.399995000	-3.225614000	-0.973683000
H	2.268483000	-2.923354000	0.083054000
O	-2.560208000	-1.520630000	2.545376000
H	-2.058901000	-2.207878000	2.054226000
H	-3.002779000	-0.981087000	1.860051000
O	-1.937220000	1.262404000	-3.221732000
H	-1.056214000	0.874396000	-3.463671000
H	-2.402347000	0.538821000	-2.760426000
O	1.659127000	2.929254000	1.674370000
H	2.353074000	2.222694000	1.769988000
H	2.016120000	3.711078000	2.121759000
C	-0.436664000	0.112305000	-0.162556000
H	-0.927832000	-0.740943000	-0.631297000
H	-0.556690000	1.014107000	-0.762307000
H	-0.810385000	0.263418000	0.852677000
F	0.944766000	-0.181578000	-0.073647000

N_2O within 5^{12} clathrate hydrate

O	-2.035317000	2.549271000	-1.930079000
H	-1.535128000	2.018568000	-2.628162000
H	-2.644782000	1.918237000	-1.491321000

O	0.599131000	-0.927662000	3.694303000
H	1.154254000	-1.429817000	3.065657000
H	-0.326043000	-1.104808000	3.412637000
O	-0.677616000	1.145688000	-3.714093000
H	0.287606000	1.216083000	-3.516195000
H	-0.890064000	0.187133000	-3.618968000
O	2.235941000	-2.469229000	1.894702000
H	1.624345000	-2.980079000	1.306177000
H	2.667806000	-3.122205000	2.465944000
O	-1.231082000	2.781310000	2.339112000
H	-0.964237000	3.099787000	1.452058000
H	-0.391832000	2.447611000	2.753498000
O	3.184010000	-1.065446000	-2.064726000
H	3.469353000	-0.939397000	-1.093677000
H	4.003114000	-1.206309000	-2.562980000
O	-3.021044000	0.824337000	2.104270000
H	-2.379849000	1.588810000	2.217633000
H	-3.345661000	0.867526000	1.180043000
O	1.164801000	-2.782692000	-2.198014000
H	1.956685000	-2.168313000	-2.191841000
H	1.178876000	-3.255492000	-3.043434000
O	2.067663000	3.139272000	-0.769218000
H	1.103290000	3.371000000	-0.584907000
H	2.471871000	3.941617000	-1.132080000
O	-3.302295000	-1.752280000	-1.350041000
H	-2.876000000	-2.249215000	-0.574083000
H	-4.009322000	-2.333631000	-1.668385000
O	3.163221000	1.752664000	1.282225000
H	2.798280000	2.304366000	0.539696000
H	3.849851000	2.291658000	1.703287000

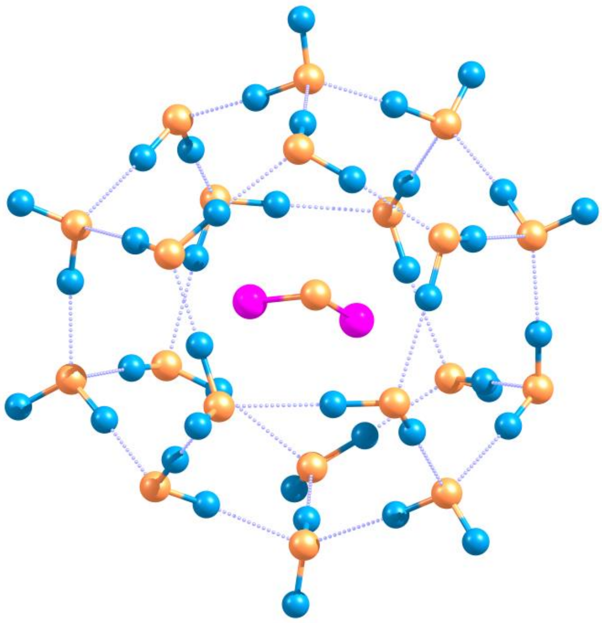

O	-2.221208000	-3.084707000	0.628376000
H	-2.202107000	-2.520800000	1.444042000
H	-1.278821000	-3.287796000	0.451755000
O	-0.468458000	3.642914000	-0.260707000
H	-1.097770000	3.163602000	-0.939267000
H	-0.737090000	4.573918000	-0.293793000
O	-2.016903000	-1.435277000	2.793083000
H	-2.542190000	-1.700160000	3.563832000
H	-2.410019000	-0.529338000	2.509005000
O	-1.197907000	-1.557408000	-3.245681000
H	-1.955278000	-1.596194000	-2.623263000
H	-0.422773000	-1.804577000	-2.706304000
O	3.920046000	-0.780884000	0.413637000
H	3.359132000	-1.375296000	0.961163000
H	3.694423000	0.128773000	0.721015000
O	0.523380000	-3.772416000	0.189972000
H	0.541990000	-4.741159000	0.161857000
H	0.772240000	-3.466734000	-0.727923000
O	2.007288000	1.369230000	-3.011257000
H	2.369876000	0.520400000	-2.686307000
H	2.007274000	1.950396000	-2.223652000
O	1.099552000	1.754373000	3.326588000
H	0.930981000	0.804026000	3.546695000
H	1.789981000	1.727976000	2.636381000
O	-3.829741000	0.799757000	-0.600685000
H	-3.700412000	-0.139347000	-0.900168000
H	-4.750122000	1.025380000	-0.801792000
O	-0.118598000	0.319007000	0.806972000
N	0.853069000	-0.088880000	0.046332000
N	-0.900304000	-0.024661000	-0.148300000

NF$_3$ within 5^{12} clathrate hydrate

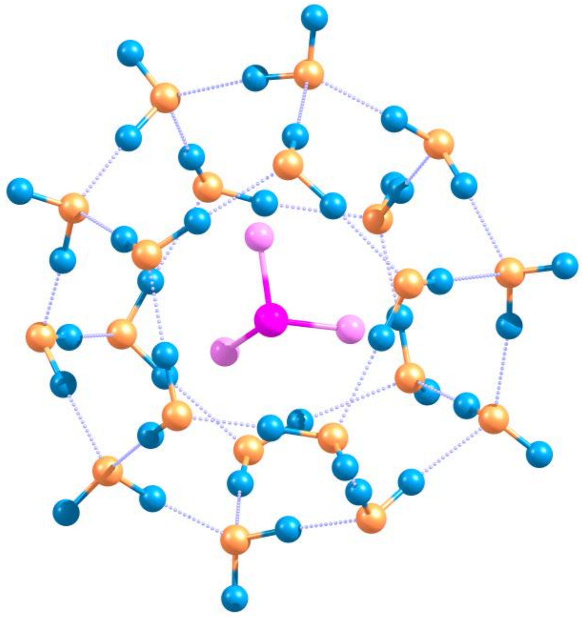

O	-3.255362000	-2.160379000	-0.345498000
H	-2.862167000	-2.128945000	-1.271768000
H	-2.576564000	-2.601480000	0.208325000
O	1.833461000	1.868965000	2.756606000
H	2.258869000	1.972784000	1.883757000
H	1.822978000	0.899288000	2.921596000
O	-2.143804000	-1.970596000	-2.752925000
H	-2.096293000	-1.007636000	-2.965088000
H	-1.210005000	-2.285641000	-2.773889000
O	3.195363000	2.314241000	0.257203000
H	3.556411000	1.457472000	-0.080275000
H	3.959414000	2.858275000	0.499234000
O	-2.199395000	0.453618000	3.045084000
H	-2.662579000	0.364481000	2.187575000
H	-1.698679000	1.310401000	2.982017000
O	0.803871000	1.545473000	-3.115758000
H	0.951104000	2.269199000	-2.408876000
H	0.784677000	2.018005000	-3.962084000
O	-0.609381000	-1.645929000	3.451690000
H	-1.216690000	-0.852203000	3.349199000
H	-0.875317000	-2.260744000	2.735456000
O	2.414124000	-0.543605000	-2.850369000
H	1.829718000	0.260561000	-2.988652000
H	2.749797000	-0.787105000	-3.726423000
O	-3.135351000	2.189812000	-1.009616000
H	-3.378337000	1.435327000	-0.386101000
H	-3.974229000	2.539917000	-1.345153000
O	1.052650000	-3.711765000	-0.031520000

H	1.761985000	-3.098604000	0.359809000
H	1.456193000	-4.592930000	-0.028364000
O	-1.169411000	3.766753000	0.031524000
H	-1.934156000	3.257875000	-0.345617000
H	-1.483454000	4.673961000	0.160621000
O	2.915237000	-2.210321000	1.019981000
H	2.559914000	-1.760341000	1.830809000
H	3.236032000	-1.485539000	0.445036000
O	-3.662062000	0.173139000	0.605848000
H	-3.464722000	-0.758111000	0.192744000
H	-4.562490000	0.099181000	0.957368000
O	1.826038000	-0.903670000	3.138810000
H	2.231117000	-1.093583000	3.998911000
H	0.840850000	-1.190440000	3.245434000
O	0.553399000	-2.762608000	-2.661239000
H	0.687520000	-3.159111000	-1.774780000
H	1.113669000	-1.963099000	-2.644226000
O	1.206065000	3.420534000	-1.380373000
H	1.885604000	3.094219000	-0.749459000
H	0.384634000	3.565168000	-0.854133000
O	3.975813000	-0.151837000	-0.700795000
H	4.897153000	-0.386576000	-0.888362000
H	3.469835000	-0.307998000	-1.546211000
O	-1.924466000	0.771864000	-3.182107000
H	-0.973882000	0.970330000	-3.060252000
H	-2.363693000	1.239142000	-2.442266000
O	-0.771650000	2.758736000	2.740553000
H	0.178053000	2.484408000	2.794325000
H	-0.886721000	3.065613000	1.821237000
O	-1.284878000	-3.368505000	1.304869000

H	-0.440117000	-3.544907000	0.811113000
H	-1.594506000	-4.230668000	1.620506000
N	0.028176000	-0.090435000	0.440267000
F	-0.446813000	-1.056777000	-0.424252000
F	1.244769000	0.237706000	-0.159412000
F	-0.744653000	0.999265000	0.092475000

O_3 within 5^{12} clathrate hydrate

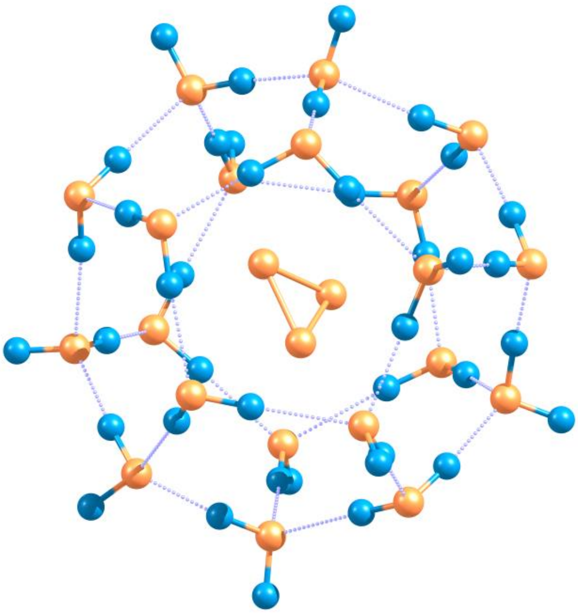

O	0.502192000	3.203562000	-2.088923000
H	0.882755000	2.435626000	-2.616387000
H	-0.438895000	2.967971000	-1.938923000
O	-1.409510000	-0.902108000	3.553055000
H	-0.998637000	-1.626413000	3.041675000
H	-2.087011000	-0.524142000	2.947424000
O	1.465895000	1.110375000	-3.405786000
H	2.218439000	0.677912000	-2.935894000
H	0.782921000	0.408550000	-3.516628000
O	-0.275064000	-3.152765000	2.160276000
H	-0.821496000	-3.319497000	1.351574000
H	-0.470117000	-3.877885000	2.772632000
O	-0.354679000	3.186470000	2.238931000
H	0.301201000	3.201252000	1.511815000
H	-0.055381000	2.439224000	2.820854000
O	2.505120000	-2.650942000	-1.035646000
H	2.461910000	-2.654922000	-0.013112000
H	3.259354000	-3.217027000	-1.260520000
O	-2.708342000	2.541414000	1.165187000
H	-1.843559000	2.812100000	1.602126000
H	-2.531860000	2.594627000	0.201607000

O	0.140376000	-3.224743000	-2.087700000
H	1.052808000	-3.034811000	-1.711023000
H	0.270961000	-3.857624000	-2.809681000
O	3.541812000	1.553463000	0.441516000
H	2.842769000	2.277205000	0.335520000
H	4.400278000	2.002183000	0.418883000
O	-2.943719000	0.150217000	-2.560362000
H	-3.183663000	-0.430959000	-1.762432000
H	-3.693384000	0.079228000	-3.170235000
O	2.858879000	-0.122874000	2.444420000
H	3.165441000	0.516377000	1.746374000
H	3.461154000	-0.010708000	3.195094000
O	-3.550311000	-1.348553000	-0.513364000
H	-3.508558000	-0.809655000	0.320864000
H	-2.904149000	-2.073018000	-0.382302000
O	1.636318000	3.337525000	0.183543000
H	1.172183000	3.258391000	-0.745132000
H	1.886068000	4.271298000	0.259402000
O	-3.289429000	0.121712000	1.753399000
H	-4.121564000	0.215781000	2.242373000
H	-3.020538000	1.093075000	1.516223000
O	-0.506432000	-0.849792000	-3.581510000
H	-1.368288000	-0.500685000	-3.270471000
H	-0.320057000	-1.624564000	-3.017603000
O	2.411337000	-2.701570000	1.549472000
H	1.479192000	-2.872604000	1.814563000
H	2.609968000	-1.796411000	1.889061000
O	-1.697500000	-3.498051000	-0.157180000
H	-2.189216000	-4.321553000	-0.297087000
H	-1.031480000	-3.445418000	-0.898875000

O	3.454070000	-0.092934000	-1.890914000
H	3.075736000	-0.940774000	-1.580638000
H	3.488704000	0.471310000	-1.091465000
O	0.523178000	1.043187000	3.686434000
H	-0.186207000	0.352825000	3.723786000
H	1.262958000	0.614705000	3.215321000
O	-2.216290000	2.591322000	-1.620119000
H	-2.498010000	1.717650000	-2.000968000
H	-2.754193000	3.261925000	-2.066935000
O	-0.174695000	0.104861000	0.748514000
O	0.685566000	0.307239000	-0.394014000
O	-0.604839000	-0.298335000	-0.569338000

CF_4 within 5^{12} clathrate hydrate

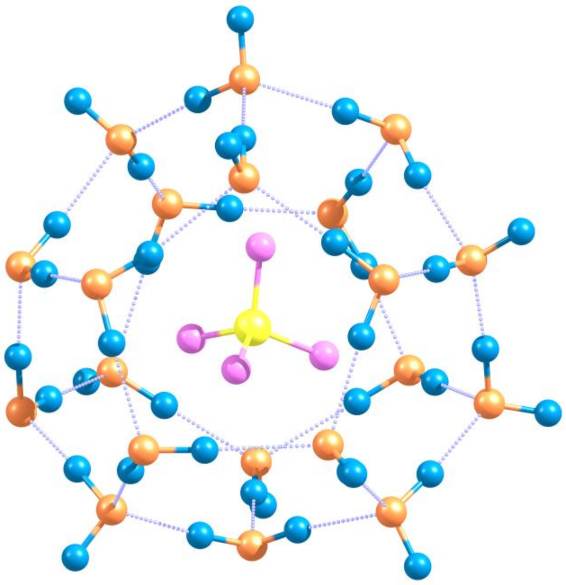

O	2.058661000	2.320708000	2.256780000
H	1.096492000	2.279448000	2.533009000
H	2.406635000	1.412472000	2.383083000
O	0.846166000	-2.438451000	-3.244719000
H	-0.104717000	-2.412711000	-3.026077000
H	1.285156000	-2.646236000	-2.389818000
O	-0.534905000	2.219427000	2.845382000
H	-1.131551000	2.632849000	2.177998000
H	-0.989704000	1.414897000	3.185288000
O	-2.000580000	-2.272104000	-2.493785000
H	-2.130453000	-2.579938000	-1.563428000
H	-2.580809000	-2.823486000	-3.039901000
O	3.431717000	0.788775000	-1.527601000
H	2.979165000	1.525774000	-1.069536000
H	2.833824000	0.558588000	-2.286897000

O	-3.639723000	1.032986000	-0.068007000
H	-3.310111000	0.767604000	-0.995664000
H	-4.491138000	1.471786000	-0.218186000
O	3.791021000	-1.340536000	0.052722000
H	3.709321000	-0.533871000	-0.538714000
H	3.586520000	-1.014930000	0.953767000
O	-3.555320000	-1.055834000	1.601811000
H	-3.625001000	-0.251713000	1.008109000
H	-4.425836000	-1.161919000	2.014009000
O	-0.114111000	3.826390000	-1.252421000
H	0.766143000	3.570067000	-0.836475000
H	-0.061540000	4.777898000	-1.426885000
O	0.734720000	-1.740099000	3.265914000
H	0.545285000	-2.348947000	2.477486000
H	0.803714000	-2.337059000	4.026820000
O	-0.667520000	1.960011000	-3.163764000
H	-0.450550000	2.687720000	-2.522896000
H	-0.635783000	2.357502000	-4.046644000
O	0.230067000	-3.371815000	1.278783000
H	0.900806000	-3.299732000	0.549489000
H	-0.633354000	-3.178563000	0.858630000
O	2.189869000	3.020445000	-0.205683000
H	2.113217000	2.719811000	0.780131000
H	2.926068000	3.651357000	-0.211494000
O	2.029107000	-3.052856000	-0.738851000
H	2.569909000	-3.842830000	-0.893586000
H	2.706240000	-2.342499000	-0.430321000
O	-1.649468000	-0.210192000	3.710018000
H	-0.834703000	-0.733499000	3.553933000
H	-2.266894000	-0.503098000	3.013596000

O	-2.836195000	0.394734000	-2.451475000
H	-2.509116000	-0.530933000	-2.520454000
H	-2.084949000	0.967774000	-2.735200000
O	-2.370021000	-3.000225000	0.144929000
H	-2.771426000	-3.853175000	0.370027000
H	-2.850924000	-2.316111000	0.688989000
O	-2.095340000	3.292664000	0.805015000
H	-2.562551000	2.495837000	0.481805000
H	-1.434901000	3.493901000	0.111378000
O	1.631667000	0.213886000	-3.500255000
H	1.361201000	-0.737866000	-3.482401000
H	0.820360000	0.719319000	-3.302730000
O	3.000527000	-0.350016000	2.632001000
H	2.196562000	-0.858355000	2.918845000
H	3.627429000	-0.399237000	3.368832000
C	-0.021657000	-0.013464000	0.013051000
F	1.150964000	-0.101706000	0.617085000
F	-0.050651000	-0.836880000	-1.029911000
F	-0.990363000	-0.360038000	0.861355000
F	-0.227820000	1.229704000	-0.400815000

SF_6 within 5^{12} clathrate hydrate

O	2.744978000	2.753857000	-1.260277000
H	1.914364000	3.276929000	-1.077409000
H	2.999292000	2.349227000	-0.403881000
O	-0.026437000	-4.130897000	0.558705000
H	-0.895872000	-3.706636000	0.677163000
H	0.583332000	-3.610691000	1.126950000
O	0.391066000	4.079959000	-0.749473000

H	-0.348818000	3.702889000	-1.276945000
H	0.097182000	3.971369000	0.183593000
O	-2.808430000	-2.716129000	0.989237000
H	-2.678831000	-2.053434000	1.709126000
H	-3.449499000	-3.356026000	1.333689000
O	3.137645000	-1.876085000	-1.770910000
H	2.854875000	-1.013112000	-2.131671000
H	2.356014000	-2.471938000	-1.889726000
O	-3.833334000	1.288973000	-0.692404000
H	-3.804390000	0.307669000	-0.933226000
H	-4.690496000	1.608385000	-1.012127000
O	3.740926000	-1.539718000	0.860430000
H	3.563962000	-1.691434000	-0.111646000
H	3.760518000	-0.566848000	0.961332000
O	-3.110889000	1.728817000	1.899241000
H	-3.387509000	1.592952000	0.950690000
H	-3.819920000	2.253211000	2.300867000
O	-0.265826000	0.649242000	-3.656587000
H	0.703883000	0.696336000	-3.403567000
H	-0.314313000	0.998297000	-4.559414000
O	1.523025000	1.772175000	3.040340000
H	1.125583000	0.872476000	3.264034000
H	1.770520000	2.153484000	3.896569000
O	-1.507257000	-1.762884000	-3.043012000
H	-1.056624000	-0.920472000	-3.303429000
H	-1.703709000	-2.219731000	-3.874890000
O	0.475147000	-0.596606000	3.679967000
H	0.942402000	-1.327197000	3.202202000
H	-0.458595000	-0.659399000	3.395380000
O	2.317471000	0.768258000	-2.904481000

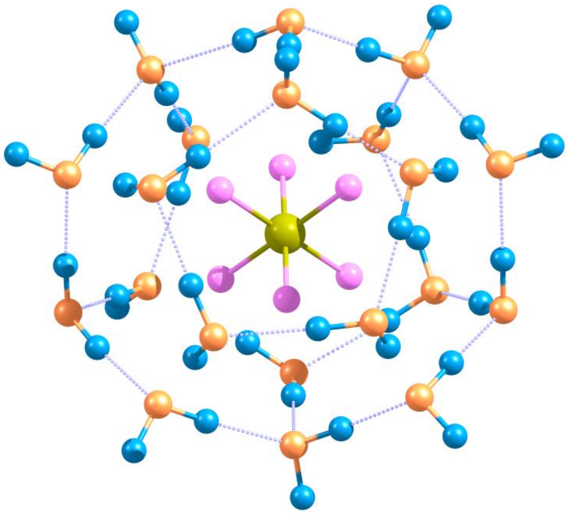

H	2.464388000	1.544173000	-2.254187000
H	2.979275000	0.903560000	-3.599842000
O	1.762364000	-2.602332000	2.233986000
H	2.212176000	-3.256008000	2.791322000
H	2.510778000	-2.182533000	1.686110000
O	-0.508132000	3.552370000	1.919598000
H	0.167605000	2.946309000	2.288724000
H	-1.314948000	3.009274000	1.866952000
O	-3.743059000	-1.303183000	-1.316271000
H	-3.453680000	-1.814862000	-0.527408000
H	-3.020265000	-1.452642000	-1.967799000
O	-2.405209000	-0.724481000	2.946468000
H	-2.860656000	-0.813313000	3.797485000
H	-2.672087000	0.167025000	2.595218000
O	-1.697641000	2.753791000	-2.213964000
H	-2.376672000	2.297165000	-1.680497000
H	-1.219669000	2.031131000	-2.667554000
O	0.826941000	-3.468685000	-2.041004000
H	0.553595000	-3.779258000	-1.143681000
H	0.071074000	-2.930550000	-2.341024000
O	3.563780000	1.420922000	1.186732000
H	2.872021000	1.571257000	1.880190000
H	4.363345000	1.859524000	1.514573000
S	-0.020791000	-0.008991000	0.015336000
F	-0.474310000	-1.373759000	0.715266000
F	-0.641491000	0.807887000	1.249327000
F	-1.436893000	0.068901000	-0.729297000
F	0.416490000	1.359598000	-0.666554000
F	1.380009000	-0.090512000	0.770472000
F	0.575751000	-0.830073000	-1.210123000

SO_2 within 5^{12} clathrate hydrate

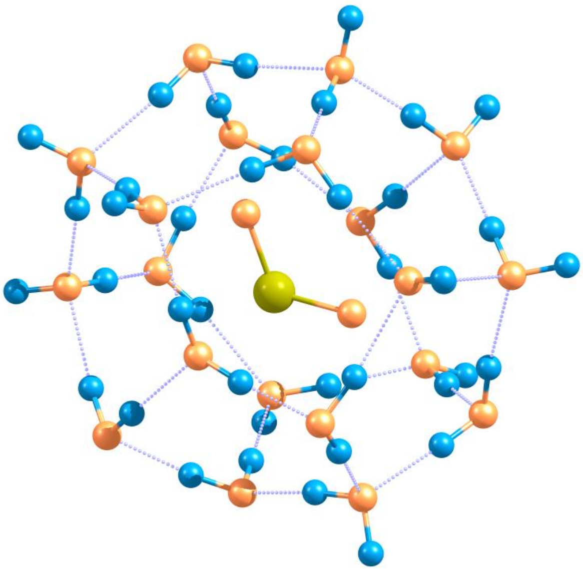

O	-2.002336000	2.897931000	1.490533000
H	-2.276927000	2.133932000	2.084783000
H	-1.027703000	2.962475000	1.562701000
O	2.300907000	-0.673339000	-3.091097000
H	2.218935000	-1.479931000	-2.539522000
H	2.787140000	-0.029171000	-2.524811000
O	-2.581422000	0.795572000	3.027315000
H	-3.030372000	0.092840000	2.492081000
H	-1.650768000	0.492593000	3.087434000
O	1.836663000	-3.018308000	-1.522709000
H	2.221061000	-2.811403000	-0.634832000
H	2.308913000	-3.803813000	-1.837708000
O	-0.521023000	3.058493000	-2.568468000
H	-1.301572000	2.993798000	-1.977563000
H	-0.387066000	2.120461000	-2.833055000
O	-1.611811000	-3.280422000	1.209332000
H	-1.368093000	-3.303849000	0.222304000
H	-2.026206000	-4.139919000	1.381322000
O	1.761751000	3.209456000	-1.102113000
H	0.935605000	3.217207000	-1.661465000
H	1.442932000	3.299404000	-0.179315000
O	0.620537000	-2.674878000	2.505036000
H	-0.218278000	-2.944293000	2.016939000
H	0.549120000	-3.062875000	3.389854000
O	-3.892993000	0.072035000	-1.169238000
H	-3.478966000	0.977589000	-1.064417000
H	-4.823501000	0.231560000	-1.386581000
O	2.487634000	1.487893000	2.696098000

H	3.063705000	0.972828000	2.026902000
H	3.070083000	1.691771000	3.442429000
O	-2.245249000	-1.442646000	-2.733759000
H	-2.865246000	-0.925992000	-2.151782000
H	-2.740702000	-1.643490000	-3.541574000
O	3.972521000	0.132423000	1.040949000
H	3.828206000	0.530010000	0.144938000
H	3.564469000	-0.756847000	0.977945000
O	-2.807445000	2.531711000	-0.916366000
H	-2.466944000	2.654290000	0.048319000
H	-3.442082000	3.254504000	-1.040090000
O	3.479095000	1.262364000	-1.433683000
H	4.222305000	1.734565000	-1.838994000
H	2.765110000	1.974720000	-1.308757000
O	0.099403000	0.097854000	2.703279000
H	0.952351000	0.553856000	2.873430000
H	0.310620000	-0.858124000	2.651773000
O	-0.949265000	-3.509674000	-1.316116000
H	0.005670000	-3.289077000	-1.399026000
H	-1.418989000	-2.803726000	-1.814634000
O	2.907130000	-2.578487000	1.004379000
H	3.504541000	-3.236067000	1.391548000
H	2.088974000	-2.596895000	1.564825000
O	-3.692604000	-1.214434000	1.443236000
H	-2.949967000	-1.847108000	1.366346000
H	-3.711012000	-0.778027000	0.567518000
O	-0.109618000	0.354764000	-2.651518000
H	0.770641000	-0.048727000	-2.923270000
H	-0.814800000	-0.305280000	-2.835736000
O	0.842671000	3.333633000	1.562841000

H	1.469821000	2.718012000	2.024631000
H	0.982321000	4.206465000	1.959545000
S	-0.021079000	0.238154000	-0.127200000
O	-1.236453000	-0.585012000	-0.065862000
O	1.251151000	-0.501767000	-0.080724000

Table S20. Cartesian coordinates for optimized geometries of the Greenhouse gases encapsulated within $5^{12}6^2$ clathrate hydrate computes at B3LYP/6-31G(d) level of theory and basis set.

CCl$_4$ within $5^{12}6^2$ clathrate hydrate

O	1.418021000	-2.280373000	-3.498446000
H	1.758496000	-1.338747000	-3.478324000
H	1.843261000	-2.683824000	-4.270496000
O	-1.825271000	2.158483000	-2.800106000
H	-2.178932000	1.243893000	-2.777066000
H	-2.185707000	2.608270000	-2.000260000
O	2.426094000	-3.374842000	-1.066894000
H	2.023664000	-3.018875000	-1.893839000
H	2.824252000	-2.607154000	-0.610329000
O	-2.777043000	3.523051000	-0.505328000
H	-1.927224000	3.791401000	-0.021887000
H	-3.209239000	4.362957000	-0.723623000
O	2.465989000	0.231243000	-3.399197000
H	1.818616000	0.973431000	-3.351014000
H	3.019629000	0.352170000	-2.602309000
O	0.794299000	2.527608000	-3.152439000
H	-0.175281000	2.329862000	-2.962577000
H	0.771500000	3.018791000	-3.988345000
O	4.318152000	0.631221000	-1.113185000
H	4.226057000	-0.156707000	-0.515016000
H	5.188715000	0.543519000	-1.530581000
O	1.595003000	4.310523000	-1.009331000
H	1.355507000	3.716828000	-1.755735000
H	2.334997000	3.862090000	-0.555938000

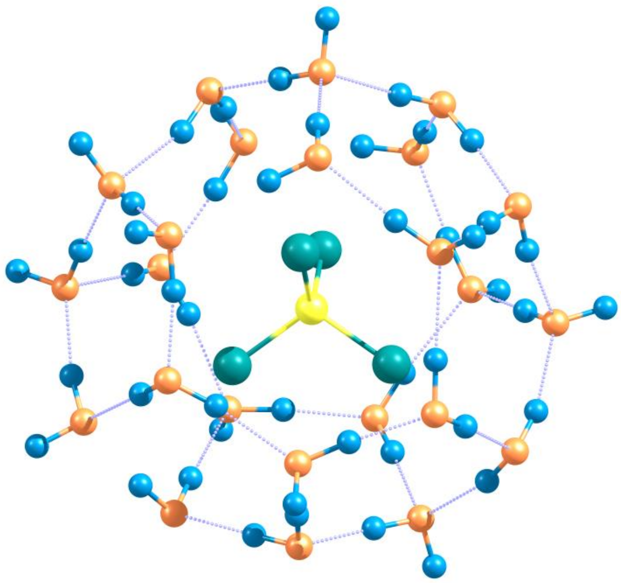

O	3.989014000	-1.545867000	0.497525000
H	4.695565000	-2.203424000	0.586088000
H	3.632699000	-1.403920000	1.423228000
O	-0.584925000	4.263412000	0.760764000
H	-0.367434000	3.617229000	1.467369000
H	0.198561000	4.289469000	0.157046000
O	-1.665255000	-2.692022000	-3.289480000
H	-0.716814000	-2.594358000	-3.500027000
H	-1.682093000	-3.187898000	-2.418401000
O	-3.102654000	-0.464578000	-2.869400000
H	-2.519514000	-1.277263000	-3.009096000
H	-3.671708000	-0.436072000	-3.653760000
O	-1.750459000	-3.923126000	-0.874195000
H	-0.913767000	-4.015775000	-0.375813000
H	-2.344014000	-3.453763000	-0.233775000
O	-4.496997000	-0.437920000	-0.467890000
H	-3.994250000	-0.447703000	-1.323454000
H	-5.352806000	-0.847028000	-0.665264000
O	0.664321000	-4.301289000	0.737900000
H	0.749466000	-5.266260000	0.702586000
H	1.316989000	-3.958351000	0.058733000
O	-4.047297000	1.775407000	1.356339000
H	-3.709455000	2.475987000	0.753877000
H	-4.340913000	1.060539000	0.754337000
O	2.538993000	1.458149000	2.944064000
H	1.593376000	1.745335000	2.982027000
H	2.894712000	1.890859000	2.140405000
O	-1.823463000	-1.793626000	3.383357000
H	-0.916908000	-2.198872000	3.376564000
H	-2.299578000	-2.257316000	4.088840000

O	3.668974000	2.773374000	0.525142000
H	3.991742000	2.063095000	-0.082406000
H	4.434424000	3.338964000	0.708437000
O	-3.276613000	-2.579962000	0.992212000
H	-3.634448000	-1.767240000	0.579096000
H	-2.737499000	-2.277475000	1.752712000
O	3.027346000	-1.143788000	2.994382000
H	2.748799000	-0.168744000	2.966990000
H	3.679813000	-1.183023000	3.711205000
O	0.675482000	-3.066231000	3.314298000
H	1.467032000	-2.492357000	3.322581000
H	0.715073000	-3.549802000	2.455602000
O	-0.064642000	2.507294000	3.005353000
H	-0.879581000	1.932940000	3.172449000
H	-0.079094000	3.149304000	3.732177000
O	-2.274137000	1.106441000	3.541624000
H	-2.107222000	0.142218000	3.470441000
H	-2.931411000	1.311266000	2.835903000
C	-0.049454000	0.054876000	0.006312000
Cl	0.464348000	-1.065738000	1.292534000
Cl	-0.178992000	-0.842121000	-1.515873000
Cl	1.150864000	1.364613000	-0.154780000
Cl	-1.627679000	0.739811000	0.437308000

CF_2Cl_2 within $5^{12}6^2$ clathrate hydrate

O	2.628862000	0.019175000	-3.050321000
H	2.113319000	0.882144000	-3.039847000
H	3.060450000	-0.003841000	-3.918005000
O	-2.974594000	0.285794000	-2.848806000

H	-2.463064000	-0.554543000	-2.842584000
H	-3.549999000	0.247101000	-2.047849000
O	4.522666000	-0.190835000	-0.976682000
H	3.869535000	-0.141261000	-1.715670000
H	4.305828000	0.574575000	-0.406199000
O	-4.559931000	0.179514000	-0.528345000
H	-4.269535000	0.952008000	0.070081000
H	-5.505470000	0.327057000	-0.683016000
O	1.356678000	2.372210000	-3.015073000
H	0.371729000	2.318323000	-3.015064000
H	1.582088000	2.792515000	-2.160643000
O	-1.454623000	2.435677000	-2.854687000
H	-1.996225000	1.578724000	-2.840407000
H	-1.847718000	2.957590000	-3.570948000
O	2.289868000	3.693318000	-0.637153000
H	2.907181000	3.094657000	-0.134804000
H	2.847128000	4.377686000	-1.037741000
O	-2.243908000	3.791203000	-0.503872000
H	-1.963942000	3.300977000	-1.309211000
H	-1.419350000	3.925341000	0.004208000
O	3.912359000	2.032663000	0.766647000
H	4.762174000	2.383122000	1.074522000
H	3.440410000	1.700543000	1.589918000
O	-3.821947000	2.152627000	1.012703000
H	-3.229493000	1.825586000	1.725564000
H	-3.268412000	2.778281000	0.473833000
O	0.964189000	-2.329405000	-3.078354000
H	1.464406000	-1.487390000	-3.044044000
H	1.367576000	-2.902237000	-2.360243000
O	-1.609676000	-2.199719000	-2.898074000

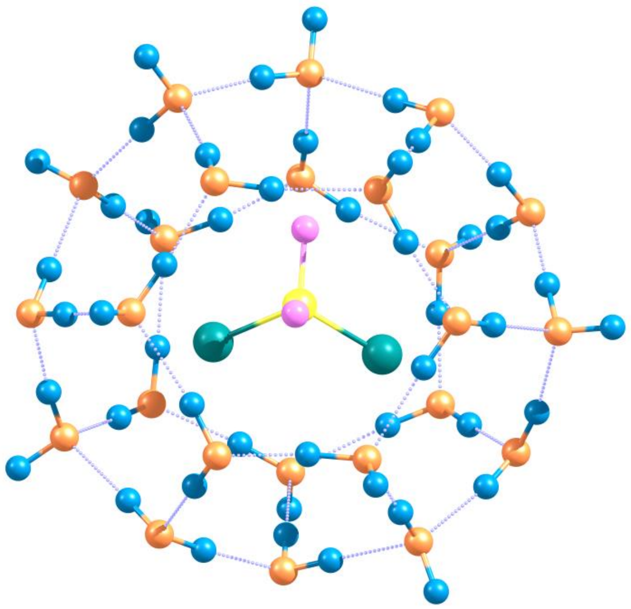

H	-0.582195000	-2.191781000	-2.916706000
H	-1.854795000	-2.602154000	-3.745558000
O	2.010869000	-3.812015000	-1.103709000
H	2.636627000	-3.287932000	-0.564515000
H	1.277732000	-4.046197000	-0.475254000
O	-2.439483000	-3.610752000	-0.731903000
H	-2.151670000	-3.106605000	-1.542596000
H	-2.829779000	-4.435992000	-1.056192000
O	3.903404000	-2.314788000	0.518495000
H	4.664978000	-2.912985000	0.551472000
H	4.186623000	-1.542636000	-0.059310000
O	-3.869542000	-2.002291000	1.115944000
H	-4.217928000	-1.266741000	0.562617000
H	-3.440408000	-2.613292000	0.479048000
O	0.353474000	2.389144000	3.090963000
H	-0.508732000	1.899894000	3.117194000
H	0.255553000	3.040817000	2.362761000
O	0.137354000	-2.690267000	2.927293000
H	1.052290000	-2.277575000	2.927118000
H	0.168670000	-3.350312000	3.636722000
O	0.168765000	4.255337000	0.997384000
H	0.939607000	4.139674000	0.388200000
H	0.172301000	5.184943000	1.270137000
O	-0.043133000	-4.334651000	0.625084000
H	-0.875662000	-4.075343000	0.177140000
H	0.019850000	-3.733141000	1.398638000
O	2.619483000	1.160392000	2.938203000
H	1.679106000	1.568403000	2.953238000
H	3.000392000	1.415000000	3.793238000
O	2.632020000	-1.699248000	2.977988000

H	2.601706000	-0.718974000	2.960909000
H	3.114777000	-1.950271000	2.158014000
O	-2.144298000	1.257136000	3.141752000
H	-2.195654000	0.240241000	3.200149000
H	-2.588354000	1.569852000	3.945115000
O	-2.337092000	-1.340141000	3.323448000
H	-1.464449000	-1.762853000	3.160265000
H	-2.916322000	-1.631702000	2.574006000
C	-0.062651000	0.281962000	-0.422501000
Cl	1.427305000	-0.474689000	0.176894000
Cl	-1.493687000	-0.469813000	0.276447000
F	-0.036371000	1.593942000	-0.122572000
F	-0.100584000	0.171044000	-1.756416000

CH_3Br within $5^{12}6^2$ clathrate hydrate

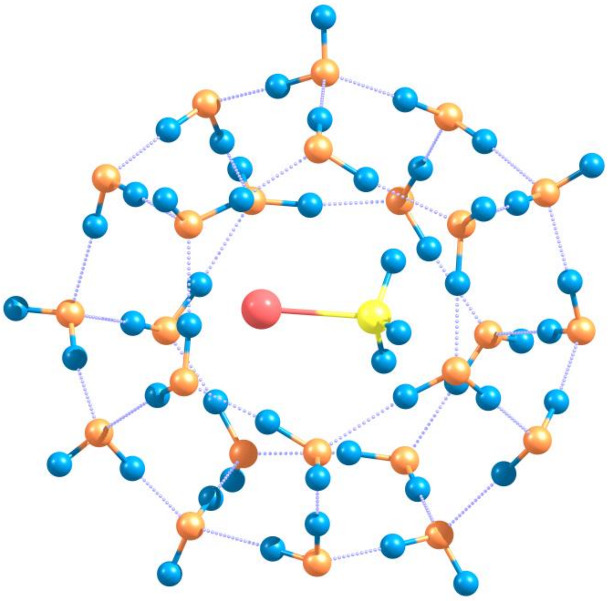

O	-0.114399000	2.334670000	-3.067015000
H	-0.944257000	1.752021000	-3.058589000
H	-0.128930000	2.766309000	-3.935218000
O	0.214942000	-2.931393000	-2.819908000
H	1.011419000	-2.354196000	-2.816953000
H	0.268844000	-3.432189000	-1.968779000
O	-0.238805000	4.273111000	-1.104382000
H	-0.192267000	3.591936000	-1.820170000
H	-0.996576000	3.999576000	-0.546125000
O	0.403845000	-4.226881000	-0.403024000
H	-0.432237000	-4.034440000	0.159402000
H	0.434360000	-5.193159000	-0.473258000
O	-2.349334000	0.969477000	-3.140290000
H	-2.203407000	-0.008681000	-3.089754000

H	-2.829918000	1.185980000	-2.315398000
O	-2.109388000	-1.756087000	-2.909104000
H	-1.169081000	-2.135049000	-2.827323000
H	-2.484937000	-2.217368000	-3.674955000
O	-3.887542000	1.798667000	-0.866993000
H	-3.349794000	2.460005000	-0.351050000
H	-4.604665000	2.299793000	-1.284235000
O	-3.500458000	-2.616486000	-0.623539000
H	-3.005779000	-2.316839000	-1.419925000
H	-3.747462000	-1.795516000	-0.153142000
O	-2.405368000	3.535369000	0.568909000
H	-2.819923000	4.352877000	0.883974000
H	-2.025469000	3.085677000	1.387374000
O	-1.692428000	-3.794888000	1.034875000
H	-1.463804000	-3.108501000	1.702153000
H	-2.381068000	-3.384253000	0.445892000
O	2.373967000	1.062808000	-3.031053000
H	1.471080000	1.451635000	-3.025816000
H	2.903606000	1.575078000	-2.345654000
O	2.616143000	-1.478785000	-2.856799000
H	2.489399000	-0.459915000	-2.919142000
H	3.019233000	-1.730880000	-3.701860000
O	3.710332000	2.375041000	-1.151538000
H	3.090508000	2.929966000	-0.633416000
H	4.081055000	1.734679000	-0.488747000
O	4.107235000	-1.913926000	-0.652471000
H	3.598483000	-1.772365000	-1.498821000
H	4.938194000	-2.342952000	-0.904386000
O	1.906143000	3.944026000	0.380704000
H	2.320318000	4.817096000	0.453627000

H	1.087496000	4.082693000	-0.196638000
O	2.496163000	-3.313904000	1.178363000
H	1.763997000	-3.663536000	0.619608000
H	3.098912000	-2.856156000	0.555369000
O	-2.635136000	0.143259000	3.091647000
H	-2.008305000	-0.627663000	3.117555000
H	-3.207918000	-0.031784000	2.312709000
O	2.966232000	0.542343000	2.999280000
H	2.363122000	1.343885000	2.937074000
H	3.531480000	0.700279000	3.770369000
O	-4.276574000	-0.282028000	0.838745000
H	-4.239392000	0.482995000	0.211064000
H	-5.213333000	-0.425741000	1.041160000
O	4.581270000	0.542209000	0.667645000
H	4.438465000	-0.329848000	0.243826000
H	4.033708000	0.527554000	1.482092000
O	-1.418980000	2.365774000	2.730046000
H	-1.849679000	1.431143000	2.828843000
H	-1.641644000	2.818370000	3.558893000
O	1.380303000	2.647876000	2.758336000
H	0.417285000	2.456841000	2.729563000
H	1.575633000	3.150199000	1.930745000
O	-1.015365000	-2.035902000	3.114238000
H	-0.004445000	-1.897570000	3.130605000
H	-1.190210000	-2.591694000	3.889315000
O	1.566038000	-1.880416000	3.268136000
H	2.020731000	-1.011477000	3.208726000
H	1.955478000	-2.430407000	2.535570000
C	0.999900000	0.079920000	0.201421000
H	1.123059000	0.870132000	0.937120000

H	1.393843000	-0.868244000	0.561171000
H	1.415839000	0.364451000	-0.762244000
Br	-0.946890000	-0.174907000	-0.047692000

CH_3Cl within $5^{12}6^2$ clathrate hydrate

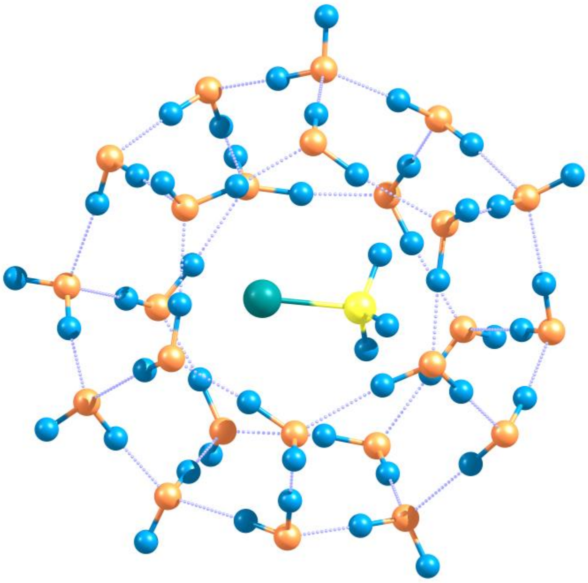

O	-0.352822000	2.317722000	-3.092224000
H	-1.141077000	1.681196000	-3.095834000
H	-0.390880000	2.761704000	-3.953395000
O	0.350203000	-2.936500000	-2.843443000
H	1.097656000	-2.297139000	-2.833861000
H	0.436800000	-3.431196000	-1.991742000
O	-0.621562000	4.198801000	-1.085832000
H	-0.525322000	3.535048000	-1.812987000
H	-1.359546000	3.866182000	-0.533430000
O	0.626319000	-4.219384000	-0.420270000
H	-0.221057000	-4.105586000	0.145777000
H	0.740695000	-5.178785000	-0.498270000
O	-2.489433000	0.789624000	-3.159886000
H	-2.271116000	-0.174137000	-3.097993000
H	-2.991550000	0.980204000	-2.341522000
O	-2.052512000	-1.915971000	-2.909241000
H	-1.090371000	-2.239764000	-2.851800000
H	-2.423226000	-2.401068000	-3.662463000
O	-4.089647000	1.508244000	-0.877572000
H	-3.614800000	2.208735000	-0.352652000
H	-4.851164000	1.946606000	-1.286499000
O	-3.367496000	-2.878010000	-0.612343000
H	-2.888459000	-2.542828000	-1.404033000
H	-3.655311000	-2.074921000	-0.133711000

O	-2.748625000	3.339687000	0.587349000
H	-3.204269000	4.143472000	0.879882000
H	-2.345824000	2.932169000	1.416415000
O	-1.496752000	-3.977362000	1.028471000
H	-1.318978000	-3.293229000	1.713149000
H	-2.211353000	-3.600909000	0.446670000
O	2.229783000	1.227652000	-3.061896000
H	1.299582000	1.545440000	-3.048268000
H	2.717225000	1.767704000	-2.368421000
O	2.633781000	-1.295986000	-2.863387000
H	2.439846000	-0.288568000	-2.932805000
H	3.058719000	-1.524114000	-3.704572000
O	3.452422000	2.606192000	-1.142864000
H	2.794306000	3.116206000	-0.625726000
H	3.861129000	1.991655000	-0.478580000
O	4.145055000	-1.638713000	-0.653360000
H	3.627631000	-1.526470000	-1.498941000
H	5.007842000	-1.996407000	-0.909582000
O	1.540911000	4.030516000	0.402919000
H	1.883855000	4.934151000	0.473567000
H	0.713820000	4.103081000	-0.174235000
O	2.642453000	-3.162362000	1.173882000
H	1.936394000	-3.557989000	0.612132000
H	3.211744000	-2.660675000	0.553368000
O	-2.701420000	-0.070220000	3.078600000
H	-2.026186000	-0.798589000	3.104773000
H	-3.265963000	-0.280979000	2.302029000
O	2.824465000	0.713331000	3.031383000
H	2.171970000	1.475463000	2.976250000
H	3.381491000	0.901318000	3.801704000

O	-4.318773000	-0.598752000	0.839016000
H	-4.327963000	0.158049000	0.201072000
H	-5.245565000	-0.807537000	1.029409000
O	4.428246000	0.834407000	0.688427000
H	4.351912000	-0.045651000	0.263888000
H	3.887409000	0.778633000	1.505451000
O	-1.676790000	2.255234000	2.758143000
H	-2.029531000	1.288176000	2.849855000
H	-1.933253000	2.682436000	3.590585000
O	1.106169000	2.718709000	2.795337000
H	0.157372000	2.466837000	2.764153000
H	1.270517000	3.225026000	1.963457000
O	-0.953403000	-2.155697000	3.109743000
H	0.046791000	-1.952955000	3.134797000
H	-1.103242000	-2.702332000	3.896470000
O	1.611491000	-1.817385000	3.278055000
H	1.994472000	-0.914386000	3.223186000
H	2.040570000	-2.329379000	2.540068000
C	0.979390000	0.156893000	0.210114000
H	1.041318000	0.926254000	0.976795000
H	1.409772000	-0.779563000	0.563189000
H	1.450451000	0.495258000	-0.711055000
Cl	-0.779301000	-0.137495000	-0.13402600

CH_4 within $5^{12}6^2$ clathrate hydrate

O	0.235327000	-2.460719000	-2.995965000
H	0.979437000	-1.779252000	-3.039355000
H	0.246221000	-2.897790000	-3.861221000
O	-0.694844000	2.808926000	-2.850593000

H	-1.409039000	2.134331000	-2.771783000
H	-0.762288000	3.358492000	-2.030864000
O	0.791173000	-4.287285000	-0.997761000
H	0.591345000	-3.657593000	-1.733406000
H	1.547382000	-3.888559000	-0.520502000
O	-0.822151000	4.256961000	-0.518134000
H	0.066197000	4.215325000	-0.013722000
H	-0.992892000	5.202943000	-0.641155000
O	2.254555000	-0.783050000	-3.145755000
H	2.001525000	0.172800000	-3.095606000
H	2.867705000	-0.924865000	-2.396305000
O	1.725647000	1.896240000	-2.955176000
H	0.751007000	2.196547000	-2.883972000
H	2.045165000	2.324465000	-3.764488000
O	4.155653000	-1.335095000	-1.076407000
H	3.784546000	-2.072802000	-0.517944000
H	4.894768000	-1.715232000	-1.574672000
O	3.235785000	3.069964000	-0.900231000
H	2.686195000	2.670030000	-1.611969000
H	3.600583000	2.305708000	-0.410881000
O	3.020667000	-3.232153000	0.457418000
H	3.524359000	-4.017049000	0.721468000
H	2.675485000	-2.823314000	1.312083000
O	1.412389000	4.181341000	0.792146000
H	1.310594000	3.503277000	1.499273000
H	2.119627000	3.828523000	0.188803000
O	-2.406955000	-1.472364000	-2.876985000
H	-1.464307000	-1.745574000	-2.889502000
H	-2.820160000	-1.985976000	-2.115489000
O	-2.848065000	1.042683000	-2.695878000

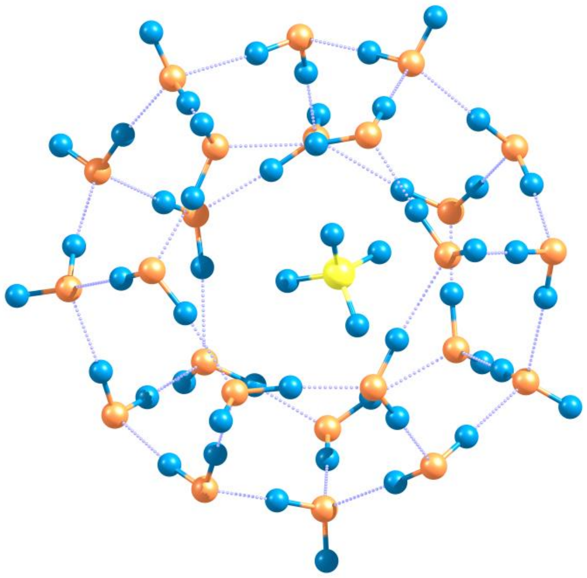

H	-2.627589000	0.035863000	-2.745804000
H	-3.359722000	1.218194000	-3.500732000
O	-3.406445000	-2.804138000	-0.810745000
H	-2.668688000	-3.234070000	-0.330409000
H	-3.802539000	-2.178499000	-0.146299000
O	-4.187624000	1.432983000	-0.385811000
H	-3.727431000	1.294323000	-1.259593000
H	-5.063793000	1.787961000	-0.596435000
O	-1.297753000	-4.096528000	0.611116000
H	-1.625296000	-4.998430000	0.746219000
H	-0.508218000	-4.188041000	-0.008717000
O	-2.702148000	3.186954000	1.248096000
H	-2.049932000	3.582632000	0.627125000
H	-3.233426000	2.572202000	0.698472000
O	2.922734000	0.307714000	2.843963000
H	2.210930000	1.002915000	2.876157000
H	3.463758000	0.537924000	2.056838000
O	-2.427940000	-0.706702000	3.071157000
H	-1.747458000	-1.444243000	3.008499000
H	-2.904383000	-0.870322000	3.899112000
O	4.396827000	0.863068000	0.523556000
H	4.403186000	0.079545000	-0.080784000
H	5.322484000	1.122828000	0.643583000
O	-4.353650000	-1.019666000	1.015077000
H	-4.330065000	-0.161923000	0.540560000
H	-3.676150000	-0.923206000	1.719509000
O	2.074074000	-2.101780000	2.654339000
H	2.360640000	-1.111582000	2.701396000
H	2.378404000	-2.483468000	3.492449000
O	-0.681740000	-2.693109000	2.908542000

CO_2 within $5^{12}6^2$ clathrate hydrate

O	-0.648289000	1.883401000	3.173777000
H	0.212260000	1.363351000	3.257544000
H	-0.874979000	2.152181000	4.077303000
O	-0.456814000	-3.414516000	2.249336000
H	-1.277604000	-2.873449000	2.175760000
H	-0.317101000	-3.800513000	1.350758000
O	-0.272802000	4.087638000	1.557458000
H	-0.398939000	3.325105000	2.173961000
H	0.581051000	3.915420000	1.108382000
O	0.009879000	-4.445708000	-0.276710000
H	0.898679000	-4.128210000	-0.666565000
H	0.070187000	-5.412852000	-0.290016000
O	1.642329000	0.612319000	3.360015000
H	1.608165000	-0.357205000	3.164139000
H	2.300106000	0.986886000	2.738580000
O	1.693998000	-2.054996000	2.744480000
H	0.812797000	-2.531322000	2.536304000
H	2.054210000	-2.540062000	3.503045000
O	3.564287000	1.862305000	1.665926000
H	3.080500000	2.561110000	1.144220000
H	4.157316000	2.337249000	2.267686000
O	3.626493000	-2.520901000	0.755513000
H	2.929689000	-2.362087000	1.431452000
H	3.858427000	-1.631721000	0.417729000
O	2.217495000	3.704341000	0.230236000
H	2.592187000	4.593666000	0.139103000
H	2.054551000	3.376131000	-0.708930000
O	2.292180000	-3.715691000	-1.289490000

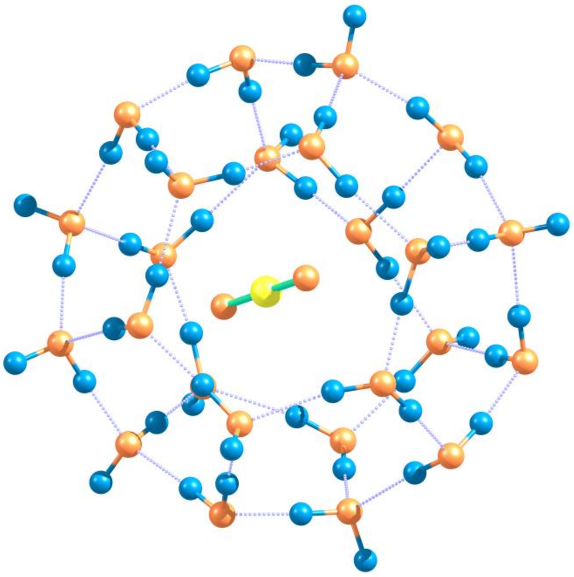

H	2.182502000	-2.983018000	-1.936402000
H	2.843807000	-3.329717000	-0.557136000
O	-3.026370000	0.422876000	2.665350000
H	-2.142818000	0.835047000	2.768414000
H	-3.465361000	0.950957000	1.932124000
O	-2.914348000	-2.072749000	2.078046000
H	-2.903862000	-1.061218000	2.282626000
H	-3.474426000	-2.452034000	2.772758000
O	-4.085403000	1.872184000	0.681874000
H	-3.389423000	2.484774000	0.366475000
H	-4.280071000	1.300588000	-0.107254000
O	-3.874660000	-2.309963000	-0.422677000
H	-3.542696000	-2.228015000	0.516122000
H	-4.730650000	-2.759419000	-0.364740000
O	-2.142071000	3.757537000	-0.275456000
H	-2.676552000	4.566095000	-0.278603000
H	-1.442688000	3.903687000	0.436703000
O	-1.811911000	-3.295074000	-2.029523000
H	-1.226788000	-3.802487000	-1.420673000
H	-2.590656000	-3.049817000	-1.482326000
O	3.264806000	0.757431000	-2.638680000
H	2.765588000	-0.065127000	-2.877041000
H	3.708565000	0.523313000	-1.793400000
O	-2.221965000	0.854321000	-3.202839000
H	-1.781615000	1.747566000	-3.033121000
H	-2.482678000	0.862127000	-4.135539000
O	4.444642000	0.055570000	-0.184367000
H	4.194833000	0.707876000	0.516769000
H	5.412246000	0.003906000	-0.174312000
O	-4.423864000	0.261135000	-1.500981000

H	-4.198874000	-0.628544000	-1.154780000
H	-3.681154000	0.489629000	-2.100100000
O	1.782907000	2.833380000	-2.235111000
H	2.297646000	1.954999000	-2.372769000
H	2.099077000	3.413340000	-2.945407000
O	-0.995744000	3.129676000	-2.705994000
H	-0.063958000	2.904115000	-2.495134000
H	-1.394353000	3.418222000	-1.849000000
O	1.865026000	-1.553194000	-3.067834000
H	0.918247000	-1.309133000	-2.774005000
H	1.783729000	-1.892878000	-3.971790000
O	-0.471400000	-0.984783000	-2.147307000
H	-1.057229000	-0.349699000	-2.622735000
H	-0.994284000	-1.839422000	-2.095577000
C	0.287501000	0.031095000	0.325245000
O	1.152700000	0.678790000	-0.127296000
O	-0.555176000	-0.589825000	0.839496000

CO within $5^{12}6^{2}$ clathrate hydrate

O	-0.033193000	-2.562081000	2.900325000
H	-0.844406000	-1.965442000	2.990134000
H	0.048325000	-3.003076000	3.759949000
O	0.454284000	2.719193000	2.806273000
H	1.234915000	2.123750000	2.714705000
H	0.493687000	3.333836000	2.032421000
O	-0.366379000	-4.418018000	0.885401000
H	-0.252774000	-3.783467000	1.635254000
H	-1.155543000	-4.096369000	0.402858000
O	0.508239000	4.350514000	0.595765000

H	-0.366070000	4.217084000	0.087059000
H	0.555758000	5.302856000	0.768957000
O	-2.204182000	-1.102661000	3.158173000
H	-2.026912000	-0.129721000	3.118127000
H	-2.790616000	-1.282726000	2.395199000
O	-1.880370000	1.617856000	2.996947000
H	-0.939891000	2.000999000	2.895693000
H	-2.212234000	2.011601000	3.818583000
O	-3.994672000	-1.776203000	1.027631000
H	-3.543339000	-2.456778000	0.455407000
H	-4.704384000	-2.241855000	1.495314000
O	-3.452218000	2.713057000	0.947707000
H	-2.895196000	2.340108000	1.667992000
H	-3.757419000	1.932171000	0.443572000
O	-2.673183000	-3.515593000	-0.547001000
H	-3.126720000	-4.313735000	-0.858171000
H	-2.347985000	-3.040248000	-1.375415000
O	-1.717365000	4.019013000	-0.693855000
H	-1.591254000	3.408439000	-1.455107000
H	-2.390094000	3.582430000	-0.105907000
O	2.522817000	-1.371934000	2.847110000
H	1.601150000	-1.705670000	2.800826000
H	3.012141000	-1.841142000	2.102653000
O	2.747110000	1.163153000	2.653651000
H	2.615693000	0.137156000	2.690366000
H	3.225899000	1.369907000	3.471182000
O	3.728945000	-2.592747000	0.816068000
H	3.034925000	-3.065460000	0.311442000
H	4.074227000	-1.911957000	0.178226000
O	4.075464000	1.771908000	0.386883000

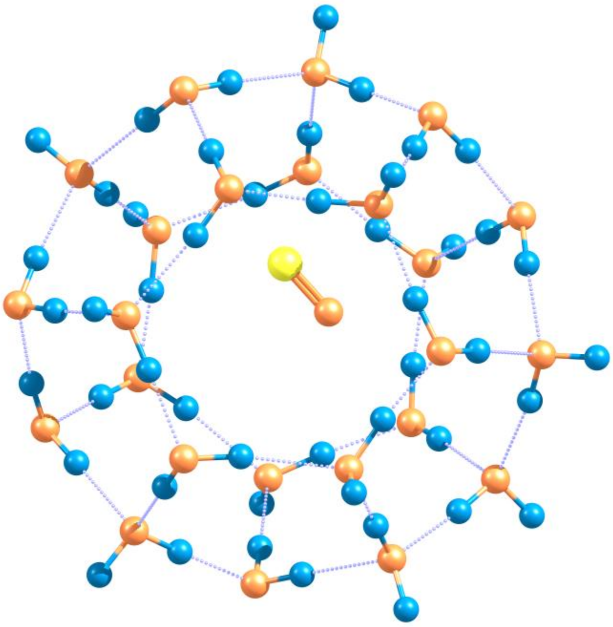

H	3.605594000	1.558134000	1.240146000
H	4.912448000	2.188037000	0.641244000
O	1.733041000	-4.006561000	-0.668157000
H	2.141639000	-4.874546000	-0.805428000
H	0.939349000	-4.177842000	-0.070356000
O	2.503136000	3.513747000	-1.193855000
H	1.815838000	3.834137000	-0.567664000
H	3.054177000	2.902765000	-0.658653000
O	-2.898223000	0.082427000	-2.835030000
H	-2.279252000	0.858563000	-2.877684000
H	-3.447057000	0.241465000	-2.035510000
O	2.492288000	-0.489413000	-2.937479000
H	1.908030000	-1.311228000	-2.946788000
H	2.973400000	-0.515806000	-3.778598000
O	-4.417568000	0.442221000	-0.508155000
H	-4.347111000	-0.354842000	0.074278000
H	-5.364124000	0.595092000	-0.647403000
O	4.504987000	-0.675161000	-0.961933000
H	4.408118000	0.181889000	-0.494626000
H	3.802717000	-0.644954000	-1.648021000
O	-1.785692000	-2.220524000	-2.671163000
H	-2.170035000	-1.264246000	-2.703229000
H	-2.025003000	-2.605996000	-3.528564000
O	0.990757000	-2.662812000	-2.965974000
H	0.060291000	-2.408645000	-2.788335000
H	1.267870000	-3.185861000	-2.175362000
O	-1.312049000	2.289992000	-2.868245000
H	-0.298002000	2.129515000	-2.909446000
H	-1.504697000	2.823711000	-3.654590000
O	1.232214000	2.000725000	-3.018849000

H	1.628253000	1.103249000	-2.945657000
H	1.724593000	2.576768000	-2.365981000
C	0.254830000	1.019380000	-0.048405000
O	-0.669175000	0.373862000	-0.215320000

H_2S within $5^{12}6^2$ clathrate hydrate

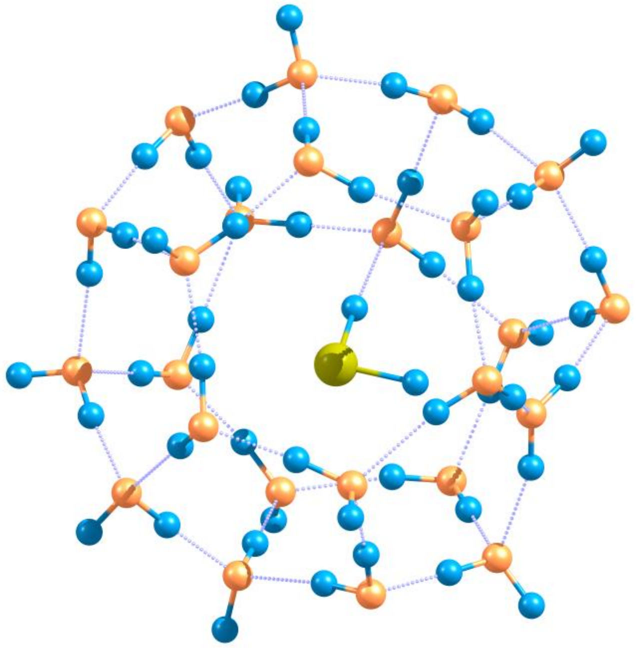

O	1.093489000	-1.934500000	-3.297341000
H	1.483055000	-0.999506000	-3.274301000
H	1.377110000	-2.299339000	-4.149679000
O	-1.943329000	2.448042000	-2.583997000
H	-2.258238000	1.517081000	-2.529184000
H	-2.175564000	2.848794000	-1.711734000
O	2.306697000	-3.456736000	-1.300281000
H	1.842876000	-2.923910000	-1.991658000
H	2.813612000	-2.806023000	-0.770529000
O	-2.582665000	3.571551000	-0.132107000
H	-1.731395000	3.854681000	0.358086000
H	-3.079768000	4.395702000	-0.246715000
O	2.235220000	0.431845000	-3.283820000
H	1.591354000	1.169897000	-3.139914000
H	2.814618000	0.462018000	-2.495111000
O	0.643567000	2.637778000	-2.858670000
H	-0.352935000	2.491089000	-2.716261000
H	0.690310000	3.219455000	-3.632980000
O	4.144360000	0.495364000	-1.145852000
H	4.073510000	-0.344002000	-0.613050000
H	4.985038000	0.441654000	-1.624776000
O	1.576091000	4.143742000	-0.688898000
H	1.229726000	3.611314000	-1.442018000

H	2.221615000	3.554761000	-0.248304000
O	3.891680000	-1.750443000	0.320479000
H	4.681854000	-2.256311000	0.563264000
H	3.412744000	-1.557296000	1.184383000
O	-0.447957000	4.380836000	1.103703000
H	-0.199687000	3.753408000	1.817894000
H	0.305782000	4.348460000	0.452463000
O	-1.704766000	-2.179999000	-3.168674000
H	-0.745278000	-1.985453000	-3.270363000
H	-1.737175000	-2.669572000	-2.304963000
O	-3.116010000	-0.093687000	-2.580948000
H	-2.500210000	-0.885099000	-2.801282000
H	-3.698863000	-0.021150000	-3.352472000
O	-1.600101000	-3.077626000	-0.648888000
H	-0.950345000	-3.721717000	-0.293882000
H	-2.366997000	-3.053064000	-0.011314000
O	-4.448154000	-0.416331000	-0.247516000
H	-3.947928000	-0.329752000	-1.107255000
H	-5.379962000	-0.507458000	-0.496077000
O	0.556095000	-4.437678000	0.404865000
H	0.675341000	-5.398730000	0.406308000
H	1.229548000	-4.076773000	-0.257483000
O	-3.830053000	1.700932000	1.501204000
H	-3.458441000	2.410595000	0.925582000
H	-4.111600000	0.999690000	0.873502000
O	2.645148000	1.345380000	2.953700000
H	1.753088000	1.752049000	3.088716000
H	2.987633000	1.776767000	2.139175000
O	-1.870211000	-1.920511000	3.177434000
H	-0.969185000	-2.345377000	2.998286000

H	-2.118733000	-2.219222000	4.064808000
O	3.563617000	2.519431000	0.581154000
H	3.824562000	1.825596000	-0.076519000
H	4.324436000	3.113737000	0.662920000
O	-3.601993000	-2.750413000	1.117121000
H	-3.940004000	-1.920241000	0.716094000
H	-3.066935000	-2.457220000	1.888260000
O	2.680646000	-1.222852000	2.629319000
H	2.574014000	-0.208897000	2.717661000
H	3.169168000	-1.484286000	3.425340000
O	0.499361000	-3.006490000	2.799952000
H	1.159391000	-2.292245000	2.662955000
H	0.526565000	-3.539880000	1.971587000
O	0.169548000	2.516799000	3.153675000
H	-0.520697000	1.799723000	2.967381000
H	-0.074593000	2.906591000	4.006406000
O	-1.549740000	0.711253000	2.453774000
H	-1.698773000	-0.177811000	2.851747000
H	-2.439467000	1.060966000	2.145665000
S	0.267842000	-0.302514000	-0.180961000
H	-0.512886000	-1.406294000	-0.366191000
H	-0.481078000	0.167323000	0.855801000

CH_3F within $5^{12}6^2$ clathrate hydrate

O	-1.809249000	1.413152000	-3.096664000
H	-1.953561000	0.409960000	-3.063959000
H	-2.149731000	1.688685000	-3.961444000
O	2.401596000	-2.006985000	-2.762821000
H	2.531016000	-1.030386000	-2.766412000

H	2.824649000	-2.314584000	-1.923264000
O	-3.267796000	2.618893000	-1.082520000
H	-2.749689000	2.188510000	-1.805678000
H	-3.599042000	1.878847000	-0.532274000
O	3.497282000	-2.766454000	-0.356323000
H	2.786437000	-3.255774000	0.194259000
H	4.221905000	-3.404442000	-0.443549000
O	-2.284691000	-1.159562000	-3.042407000
H	-1.453948000	-1.685531000	-2.938269000
H	-2.785022000	-1.307123000	-2.213347000
O	-0.077057000	-2.787978000	-2.771014000
H	0.873814000	-2.421742000	-2.746334000
H	-0.085836000	-3.374829000	-3.542845000
O	-4.043022000	-1.651585000	-0.845128000
H	-4.202847000	-0.813588000	-0.332696000
H	-4.884378000	-1.882304000	-1.266282000
O	-0.416757000	-4.468102000	-0.554846000
H	-0.277526000	-3.888127000	-1.337761000
H	-1.185064000	-4.073397000	-0.094358000
O	-4.389995000	0.614800000	0.587161000
H	-5.256624000	0.929469000	0.884952000
H	-3.798950000	0.608647000	1.398544000
O	1.721559000	-4.007016000	1.056054000
H	1.382696000	-3.372381000	1.727993000
H	0.939403000	-4.234404000	0.483464000
O	0.847305000	2.260972000	-2.978398000
H	-0.070280000	1.913718000	-3.030939000
H	0.827856000	3.019990000	-2.317333000
O	2.884400000	0.737245000	-2.773560000
H	2.029785000	1.309921000	-2.826627000

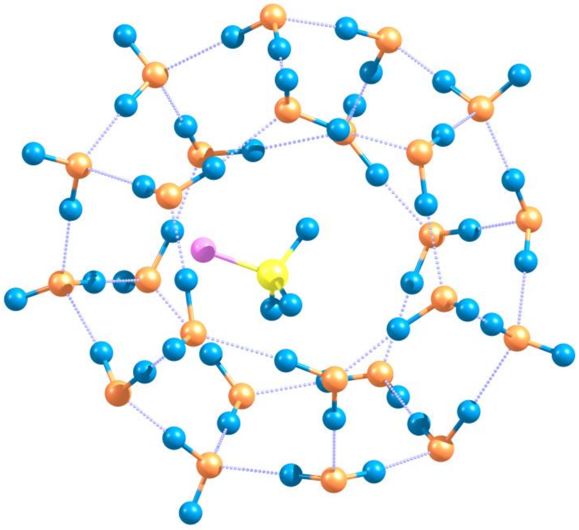

H	3.343531000	0.915025000	-3.608988000
O	0.802237000	4.209208000	-1.182324000
H	-0.014657000	4.145522000	-0.644389000
H	1.542037000	4.073835000	-0.532218000
O	4.213076000	1.538943000	-0.563035000
H	3.756194000	1.253824000	-1.402172000
H	5.094088000	1.841264000	-0.829330000
O	-1.572833000	4.009073000	0.366944000
H	-1.924371000	4.911548000	0.399000000
H	-2.230739000	3.483921000	-0.193964000
O	4.389076000	-0.637799000	1.239135000
H	4.095105000	-1.400292000	0.690950000
H	4.348658000	0.130175000	0.629953000
O	-1.844471000	-1.758864000	2.954166000
H	-0.856970000	-1.848053000	2.999979000
H	-2.112752000	-2.312332000	2.186406000
O	1.573645000	2.345274000	2.819896000
H	0.577845000	2.487906000	2.822328000
H	1.898337000	2.802853000	3.610392000
O	-2.658715000	-3.321630000	0.792270000
H	-3.189379000	-2.760561000	0.170792000
H	-3.242420000	-4.044765000	1.066418000
O	2.772710000	3.670261000	0.626327000
H	3.301711000	2.954712000	0.214240000
H	2.340076000	3.233707000	1.392171000
O	-2.819839000	0.601989000	2.744006000
H	-2.363324000	-0.319807000	2.784120000
H	-3.286642000	0.672186000	3.591461000
O	-1.035461000	2.782881000	2.773674000
H	-1.620515000	1.994033000	2.774013000

H	-1.255174000	3.268071000	1.941483000
O	0.837164000	-2.204180000	3.011450000
H	1.482860000	-1.403536000	3.033033000
H	1.031949000	-2.686972000	3.829818000
O	2.522367000	-0.272994000	3.151015000
H	2.155816000	0.626208000	2.997884000
H	3.246961000	-0.394464000	2.473619000
C	-0.405188000	0.402006000	0.066107000
H	-0.434328000	1.076356000	0.924863000
H	0.238701000	-0.455963000	0.282444000
H	-0.042537000	0.939579000	-0.814107000
F	-1.706029000	-0.062388000	-0.181904000

N_2O within $5^{12}6^2$ clathrate hydrate

O	0.333767000	2.674366000	-2.917917000
H	-0.592116000	2.272414000	-2.946703000
H	0.426670000	3.150668000	-3.757038000
O	-0.482957000	-2.657014000	-3.057447000
H	0.405747000	-2.240253000	-2.968644000
H	-0.559636000	-3.265284000	-2.280876000
O	0.595387000	4.349677000	-0.731913000
H	0.526784000	3.769510000	-1.529008000
H	-0.216039000	4.163602000	-0.216482000
O	-0.681303000	-4.255155000	-0.827616000
H	-1.485687000	-3.968706000	-0.262217000
H	-0.849432000	-5.186730000	-1.036352000
O	-2.127248000	1.739465000	-3.031794000
H	-2.178849000	0.751371000	-3.035548000
H	-2.602799000	2.009789000	-2.219947000

O	-2.469510000	-0.989377000	-2.981317000
H	-1.653249000	-1.602069000	-2.982203000
H	-2.949441000	-1.234189000	-3.787598000
O	-3.572697000	2.674397000	-0.739004000
H	-2.935565000	3.191922000	-0.172118000
H	-4.179193000	3.323049000	-1.127110000
O	-4.154984000	-1.806970000	-0.886839000
H	-3.552015000	-1.537058000	-1.616234000
H	-4.237432000	-1.006829000	-0.329919000
O	-1.780135000	3.949102000	0.808230000
H	-1.990484000	4.826395000	1.162401000
H	-1.545918000	3.376249000	1.604676000
O	-2.717150000	-3.570024000	0.613387000
H	-2.382807000	-3.033329000	1.368519000
H	-3.290815000	-2.956391000	0.080683000
O	2.493528000	0.878334000	-2.930631000
H	1.697338000	1.451777000	-2.884595000
H	3.091074000	1.181594000	-2.181293000
O	2.101314000	-1.636405000	-2.871724000
H	2.212950000	-0.605815000	-2.854724000
H	2.489588000	-1.899599000	-3.720598000
O	4.020699000	1.633083000	-0.891036000
H	3.571941000	2.298687000	-0.329186000
H	4.212432000	0.869602000	-0.285637000
O	3.375531000	-2.687051000	-0.741330000
H	2.928339000	-2.314733000	-1.552106000
H	4.080431000	-3.265875000	-1.067753000
O	2.631622000	3.507795000	0.728485000
H	3.163043000	4.289870000	0.939131000
H	1.871896000	3.838030000	0.154443000

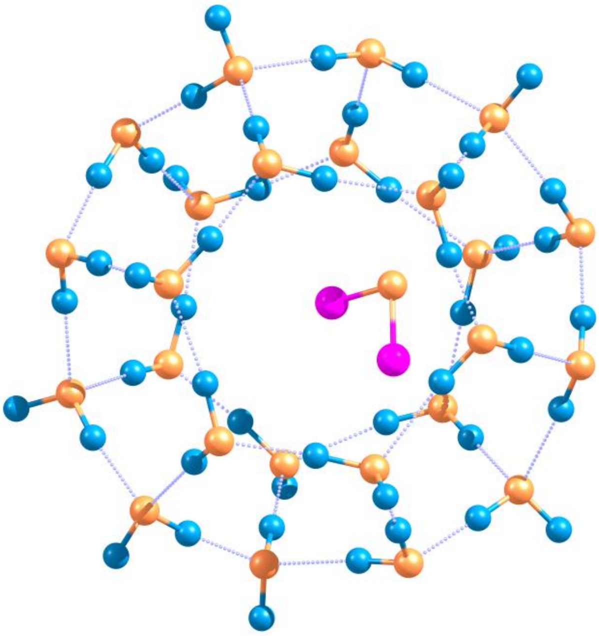

O	1.542152000	-4.119944000	0.872935000
H	0.755946000	-4.195494000	0.286041000
H	2.201107000	-3.633357000	0.333473000
O	-2.745507000	0.392268000	2.973703000
H	-2.315905000	-0.503144000	2.941619000
H	-3.351168000	0.409378000	2.200271000
O	2.675779000	-0.464375000	2.937743000
H	2.259323000	0.450172000	2.890477000
H	3.180691000	-0.475229000	3.764796000
O	-4.431380000	0.540641000	0.733693000
H	-4.201509000	1.328733000	0.180461000
H	-5.372442000	0.624588000	0.948311000
O	4.432082000	-0.522020000	0.732101000
H	4.113904000	-1.300781000	0.228587000
H	3.864676000	-0.504356000	1.533095000
O	-1.145475000	2.392286000	2.842626000
H	-1.740934000	1.551546000	2.864900000
H	-1.215219000	2.769170000	3.733358000
O	1.631644000	1.952270000	2.777707000
H	0.655333000	2.011265000	2.704138000
H	2.000383000	2.516268000	2.056442000
O	-1.739030000	-2.128544000	2.812172000
H	-0.720705000	-2.237606000	2.829151000
H	-2.045807000	-2.646628000	3.572415000
O	0.800050000	-2.562066000	2.949706000
H	1.421847000	-1.801434000	2.955001000
H	1.103775000	-3.160040000	2.210051000
O	1.184346000	-0.248826000	0.245636000
N	0.973627000	0.975263000	-0.101486000
N	0.135764000	-0.137835000	0.993628000

NF$_3$ within $5^{12}6^2$ clathrate hydrate

O	-0.249001000	-2.542443000	2.895920000
H	-0.988920000	-1.856310000	2.922409000
H	-0.258308000	-2.958111000	3.771777000
O	0.652943000	2.748980000	2.917421000
H	1.360015000	2.067858000	2.825490000
H	0.756154000	3.337584000	2.128778000
O	-0.692367000	-4.357437000	0.863806000
H	-0.555455000	-3.741505000	1.624714000
H	-1.458317000	-3.992817000	0.375481000
O	0.896557000	4.283267000	0.654291000
H	0.015664000	4.249569000	0.134039000
H	1.042614000	5.225272000	0.829921000
O	-2.253119000	-0.844810000	2.980200000
H	-2.025032000	0.118203000	3.007856000
H	-2.818073000	-0.954086000	2.188697000
O	-1.771311000	1.846352000	2.975927000
H	-0.798135000	2.153607000	2.919078000
H	-2.096140000	2.236664000	3.801992000
O	-4.088989000	-1.373444000	0.868744000
H	-3.695300000	-2.108581000	0.321835000
H	-4.795281000	-1.774175000	1.397593000
O	-3.212641000	3.087158000	0.913807000
H	-2.702495000	2.654405000	1.635305000
H	-3.605228000	2.348451000	0.407751000
O	-2.903166000	-3.276811000	-0.619753000
H	-3.435200000	-4.009219000	-0.966515000
H	-2.505058000	-2.816742000	-1.423962000
O	-1.315375000	4.213200000	-0.685316000

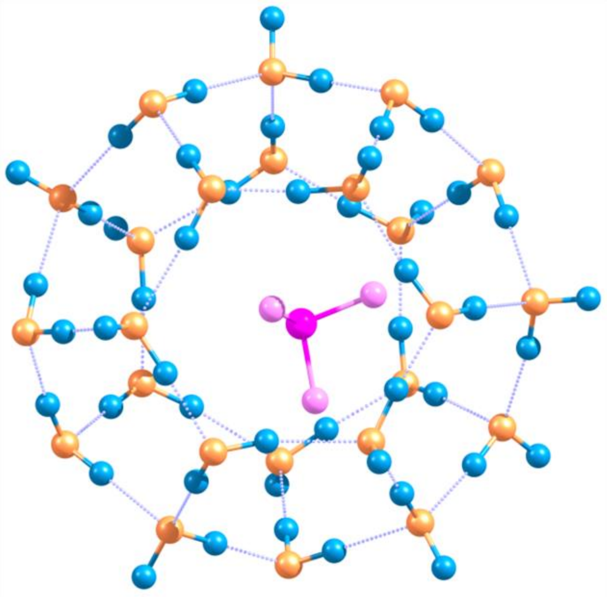

H	-1.186467000	3.558433000	-1.409674000
H	-2.042728000	3.843897000	-0.116631000
O	2.383820000	-1.534025000	2.908396000
H	1.446038000	-1.816260000	2.843144000
H	2.863184000	-2.033028000	2.176831000
O	2.784446000	0.977548000	2.733656000
H	2.575498000	-0.036914000	2.758465000
H	3.259513000	1.138677000	3.563656000
O	3.559036000	-2.827248000	0.906361000
H	2.840505000	-3.245936000	0.388364000
H	3.967395000	-2.173221000	0.278261000
O	4.249831000	1.500193000	0.530381000
H	3.733134000	1.317074000	1.363856000
H	5.110961000	1.836407000	0.819859000
O	1.480489000	-4.061313000	-0.614635000
H	1.810424000	-4.959113000	-0.770254000
H	0.654073000	-4.177368000	-0.049272000
O	2.899057000	3.385635000	-1.098116000
H	2.200306000	3.725380000	-0.494848000
H	3.390114000	2.731491000	-0.556152000
O	-2.708764000	0.408494000	-2.827880000
H	-1.999368000	1.105041000	-2.822326000
H	-3.292933000	0.630702000	-2.069873000
O	2.632188000	-0.585947000	-2.891426000
H	1.959185000	-1.336398000	-2.880092000
H	3.120739000	-0.701967000	-3.720611000
O	-4.338585000	0.913186000	-0.599642000
H	-4.339956000	0.092409000	-0.047292000
H	-5.265998000	1.108777000	-0.800781000
O	4.530534000	-0.963566000	-0.835859000

H	4.487563000	-0.106307000	-0.361666000
H	3.868983000	-0.863322000	-1.555322000
O	-1.832734000	-1.992847000	-2.663739000
H	-2.136996000	-1.009081000	-2.686451000
H	-2.030180000	-2.330832000	-3.551194000
O	0.909113000	-2.589171000	-2.879025000
H	0.004566000	-2.258711000	-2.695312000
H	1.134392000	-3.152577000	-2.099961000
O	-0.895721000	2.423111000	-2.791327000
H	0.109384000	2.207856000	-2.851108000
H	-1.062962000	2.975801000	-3.570546000
O	1.626504000	2.024492000	-3.052350000
H	1.941777000	1.096764000	-2.966381000
H	2.128241000	2.543855000	-2.361983000
N	-0.281703000	-0.082823000	0.190240000
F	-0.728338000	-1.399939000	0.124353000
F	-1.473834000	0.619114000	0.083324000
F	0.262622000	0.090573000	-1.064050000

O_3 within $5^{12}6^2$ clathrate hydrate

O	0.136376000	2.543332000	2.922541000
H	0.937180000	1.929515000	2.987471000
H	0.100657000	2.999186000	3.777594000
O	-0.396500000	-2.797325000	2.853791000
H	-1.157799000	-2.177798000	2.759467000
H	-0.424072000	-3.366960000	2.045546000
O	0.491494000	4.373792000	0.883693000
H	0.363451000	3.742119000	1.633455000
H	1.278543000	4.040980000	0.405400000

O	-0.426314000	-4.303234000	0.553447000
H	0.459914000	-4.200912000	0.055209000
H	-0.512287000	-5.256088000	0.708687000
O	2.275790000	1.033508000	3.109487000
H	2.084926000	0.062579000	3.067775000
H	2.863362000	1.209174000	2.346683000
O	1.931640000	-1.676739000	2.943551000
H	0.985774000	-2.061153000	2.873068000
H	2.280072000	-2.060475000	3.763242000
O	4.093896000	1.697354000	0.997746000
H	3.655831000	2.391106000	0.430728000
H	4.796325000	2.150327000	1.488338000
O	3.542359000	-2.786139000	0.933199000
H	2.962218000	-2.409132000	1.633416000
H	3.853465000	-2.009464000	0.426557000
O	2.791321000	3.466145000	-0.555826000
H	3.246330000	4.266559000	-0.858835000
H	2.458637000	3.003062000	-1.388392000
O	1.813770000	-4.076915000	-0.733144000
H	1.668234000	-3.427611000	-1.458806000
H	2.489927000	-3.658331000	-0.136302000
O	-2.412299000	1.333585000	2.876816000
H	-1.496570000	1.685576000	2.873815000
H	-2.878222000	1.806500000	2.122375000
O	-2.671623000	-1.207323000	2.673647000
H	-2.518016000	-0.189075000	2.716715000
H	-3.163538000	-1.409327000	3.484470000
O	-3.546555000	2.548356000	0.800462000
H	-2.868502000	3.037520000	0.289614000
H	-3.916692000	1.888151000	0.156468000

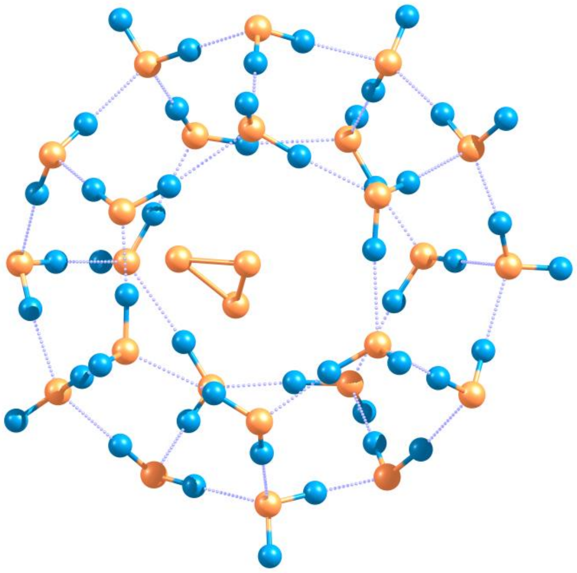

O	-3.991012000	-1.769315000	0.383939000
H	-3.521876000	-1.562811000	1.239498000
H	-4.821875000	-2.200096000	0.633882000
O	-1.600352000	3.996540000	-0.688374000
H	-1.994403000	4.871360000	-0.823991000
H	-0.806873000	4.151818000	-0.086295000
O	-2.433182000	-3.494461000	-1.227505000
H	-1.728049000	-3.795988000	-0.611077000
H	-2.982876000	-2.886853000	-0.687323000
O	2.997720000	-0.116040000	-2.868140000
H	2.355077000	-0.874718000	-2.886831000
H	3.559047000	-0.283895000	-2.079255000
O	-2.438672000	0.467931000	-3.005693000
H	-1.833939000	1.275469000	-2.980205000
H	-2.907662000	0.534440000	-3.851509000
O	4.513973000	-0.517443000	-0.540879000
H	4.453905000	0.280151000	0.042127000
H	5.458602000	-0.683142000	-0.678991000
O	-4.392519000	0.667857000	-0.979179000
H	-4.304920000	-0.192405000	-0.515963000
H	-3.731355000	0.619420000	-1.703662000
O	1.889068000	2.189690000	-2.683297000
H	2.282541000	1.238246000	-2.729427000
H	2.104777000	2.585381000	-3.542206000
O	-0.893654000	2.608052000	-2.971442000
H	0.031850000	2.341375000	-2.787724000
H	-1.165413000	3.135061000	-2.181704000
O	1.359903000	-2.278960000	-2.839302000
H	0.346090000	-2.120489000	-2.902698000
H	1.560872000	-2.820668000	-3.618345000

O	-1.180795000	-2.025746000	-3.101270000
H	-1.579777000	-1.128244000	-3.051063000
H	-1.676451000	-2.585735000	-2.437313000
O	-0.250001000	0.114501000	0.726578000
O	-1.485116000	0.368868000	0.032230000
O	-0.549525000	-0.595335000	-0.492539000

CF_4 within $5^{12}6^2$ clathrate hydrate

O	0.460127000	-2.413094000	-2.925318000
H	1.161152000	-1.688100000	-2.963324000
H	0.500061000	-2.847078000	-3.791400000
O	-0.822735000	2.709283000	-2.838574000
H	-1.487371000	1.983480000	-2.783444000
H	-0.972516000	3.259180000	-2.029927000
O	1.045461000	-4.218151000	-0.921829000
H	0.855051000	-3.590218000	-1.661490000
H	1.772920000	-3.802693000	-0.415234000
O	-1.191202000	4.156788000	-0.533468000
H	-0.315155000	4.184858000	-0.005443000
H	-1.409599000	5.087292000	-0.694963000
O	2.380895000	-0.619930000	-3.041109000
H	2.084872000	0.324526000	-3.019772000
H	2.967058000	-0.726952000	-2.264999000
O	1.672823000	2.024158000	-2.913810000
H	0.674401000	2.230766000	-2.848858000
H	1.951743000	2.462438000	-3.732658000
O	4.218168000	-1.076536000	-0.889224000
H	3.881307000	-1.839616000	-0.343920000
H	5.002177000	-1.401272000	-1.357278000

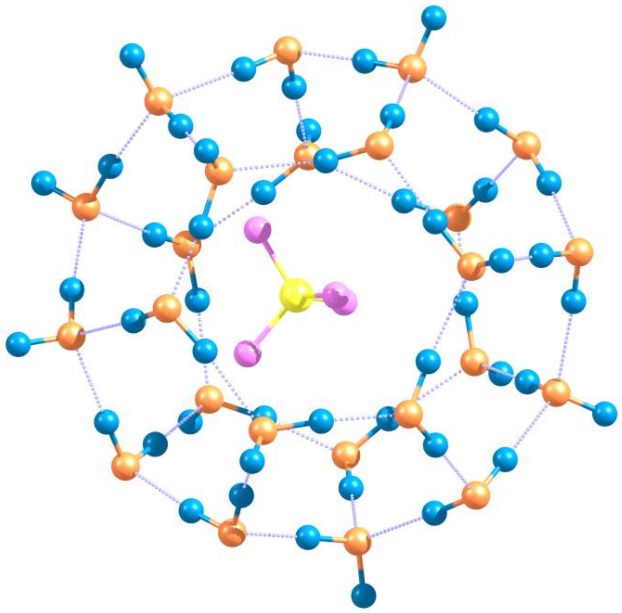

O	2.991750000	3.340863000	-0.806415000
H	2.520499000	2.903018000	-1.550630000
H	3.415956000	2.605452000	-0.321228000
O	3.184819000	-3.073311000	0.604287000
H	3.739658000	-3.809627000	0.903238000
H	2.753781000	-2.693131000	1.431302000
O	1.006865000	4.254736000	0.827787000
H	0.930644000	3.578231000	1.539071000
H	1.764303000	3.963672000	0.253350000
O	-2.259688000	-1.655057000	-2.962795000
H	-1.297883000	-1.834106000	-2.887590000
H	-2.685860000	-2.197654000	-2.229666000
O	-2.860520000	0.815335000	-2.742352000
H	-2.584103000	-0.181413000	-2.796665000
H	-3.351080000	0.972686000	-3.563912000
O	-3.297920000	-3.065225000	-0.960770000
H	-2.547236000	-3.432512000	-0.449636000
H	-3.760402000	-2.463987000	-0.317614000
O	-4.326227000	1.157653000	-0.504305000
H	-3.806739000	1.039816000	-1.348007000
H	-5.215379000	1.429593000	-0.775984000
O	-1.143858000	-4.169005000	0.556772000
H	-1.412100000	-5.094159000	0.663034000
H	-0.308046000	-4.197844000	-0.006619000
O	-3.135371000	3.091097000	1.187323000
H	-2.456995000	3.492873000	0.599200000
H	-3.571523000	2.415604000	0.624951000
O	2.612610000	0.482628000	2.842029000
H	1.868058000	1.139836000	2.891430000
H	3.166280000	0.771086000	2.083394000

O	-2.569990000	-0.878992000	2.922039000
H	-1.845980000	-1.579260000	2.910111000
H	-3.069511000	-1.051336000	3.734711000
O	4.207925000	1.179797000	0.647776000
H	4.291991000	0.382912000	0.066870000
H	5.110367000	1.432406000	0.894062000
O	-4.415541000	-1.335720000	0.829448000
H	-4.428997000	-0.468831000	0.371148000
H	-3.764818000	-1.207213000	1.554194000
O	1.997017000	-2.005438000	2.717303000
H	2.192245000	-0.996901000	2.744164000
H	2.262854000	-2.332770000	3.590754000
O	-0.723308000	-2.776710000	2.906372000
H	0.174322000	-2.405552000	2.774884000
H	-0.891908000	-3.316197000	2.096909000
O	0.699412000	2.397823000	2.905361000
H	-0.289057000	2.111325000	2.954694000
H	0.825477000	2.940343000	3.699263000
O	-1.786704000	1.806181000	3.142652000
H	-2.015649000	0.855879000	3.033658000
H	-2.321011000	2.292410000	2.452717000
C	0.384864000	0.010760000	-0.001985000
F	1.113679000	1.121877000	0.000121000
F	-0.455605000	0.029812000	-1.027610000
F	-0.292320000	-0.084030000	1.134452000
F	1.198034000	-1.045348000	-0.116782000

SF_6 within $5^{12}6^2$ clathrate hydrate

O	-1.128534000	-2.269838000	3.038584000

H	-1.601344000	-1.382313000	3.111541000
H	-1.306455000	-2.722330000	3.877238000
O	1.513003000	2.426681000	2.900630000
H	1.958550000	1.550064000	2.851466000
H	1.782679000	2.900328000	2.075369000
O	-2.146506000	-3.757486000	0.931547000
H	-1.797775000	-3.231931000	1.692386000
H	-2.726561000	-3.144833000	0.435562000
O	2.216515000	3.709402000	0.559920000
H	1.371344000	3.944026000	0.035875000
H	2.644317000	4.563220000	0.726686000
O	-2.485195000	-0.004293000	3.201652000
H	-1.933391000	0.816068000	3.187948000
H	-3.041346000	0.052964000	2.398564000
O	-1.089728000	2.380503000	3.059884000
H	-0.075463000	2.343984000	2.979302000
H	-1.249482000	2.912476000	3.854643000
O	-4.312417000	0.066503000	0.984281000
H	-4.204042000	-0.742092000	0.412253000
H	-5.158017000	-0.039254000	1.445994000
O	-2.019297000	3.946153000	0.905929000
H	-1.682967000	3.402257000	1.653465000
H	-2.622593000	3.352161000	0.416258000
O	-3.896076000	-2.078582000	-0.598113000
H	-4.633812000	-2.646408000	-0.867528000
H	-3.424802000	-1.810410000	-1.446552000
O	0.090459000	4.348770000	-0.777236000
H	-0.036847000	3.708677000	-1.513862000
H	-0.699756000	4.246419000	-0.183237000
O	1.723183000	-2.233866000	2.961567000

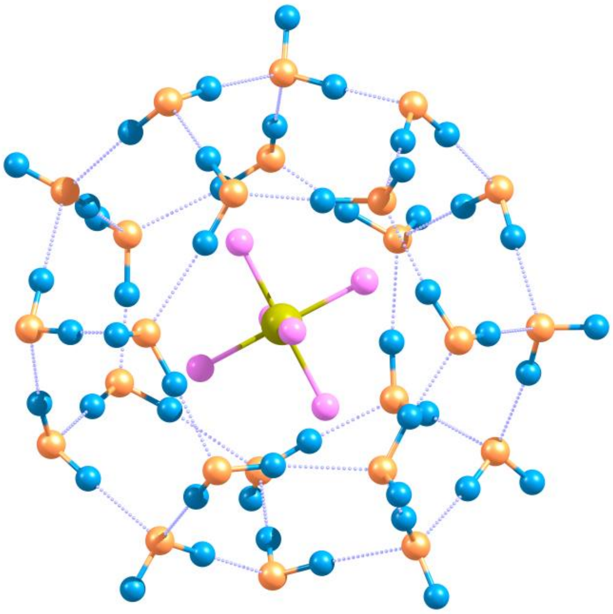

H	0.744427000	-2.177165000	2.959742000
H	1.955052000	-2.845448000	2.198120000
O	2.943888000	0.014609000	2.768173000
H	2.414202000	-0.869759000	2.814741000
H	3.496047000	0.004670000	3.565215000
O	2.289565000	-3.812906000	0.884583000
H	1.468872000	-3.997410000	0.382838000
H	2.865201000	-3.332025000	0.232610000
O	4.371824000	0.013465000	0.466644000
H	3.864872000	0.024079000	1.324946000
H	5.307965000	-0.028166000	0.712214000
O	-0.083952000	-4.295126000	-0.636136000
H	-0.110147000	-5.253871000	-0.775527000
H	-0.864982000	-4.094916000	-0.032012000
O	3.745379000	2.192618000	-1.243298000
H	3.235273000	2.771153000	-0.633273000
H	3.999134000	1.428245000	-0.684151000
O	-2.560010000	1.242432000	-2.916059000
H	-1.668439000	1.674296000	-2.977663000
H	-2.970920000	1.626489000	-2.110558000
O	2.104319000	-1.449768000	-3.053057000
H	1.228547000	-1.944684000	-3.038650000
H	2.560101000	-1.766671000	-3.847583000
O	-3.774469000	2.236496000	-0.582137000
H	-4.038086000	1.485265000	0.006383000
H	-4.583124000	2.741452000	-0.755706000
O	3.735401000	-2.365044000	-0.923033000
H	3.971816000	-1.536309000	-0.455214000
H	3.144425000	-2.070328000	-1.649801000
O	-2.602314000	-1.334364000	-2.794872000

H	-2.522823000	-0.311339000	-2.823474000
H	-3.012102000	-1.572840000	-3.640921000
O	-0.168699000	-2.827342000	-2.992407000
H	-0.950092000	-2.246163000	-2.885278000
H	-0.138466000	-3.371586000	-2.169548000
O	-0.182766000	2.594922000	-2.963429000
H	0.695466000	2.069576000	-3.039518000
H	-0.160709000	3.207379000	-3.714953000
O	2.078130000	1.373125000	-3.221631000
H	2.045259000	0.393701000	-3.149913000
H	2.720464000	1.673360000	-2.519771000
S	0.020916000	-0.008742000	-0.002567000
F	-0.108058000	-0.067306000	-1.596794000
F	0.829651000	-1.385152000	-0.013147000
F	1.383991000	0.810229000	-0.129806000
F	0.124262000	0.045105000	1.593415000
F	-1.363476000	-0.827987000	0.126357000
F	-0.809453000	1.363922000	0.009808000

SO_2 within $5^{12}6^2$ clathrate hydrate

O	-0.088941000	2.675191000	2.709914000
H	0.812268000	2.230840000	2.804833000
H	-0.168351000	3.240770000	3.493253000
O	-0.022935000	-1.711470000	2.135011000
H	-0.924282000	-1.485225000	2.494458000
H	-0.062169000	-2.658505000	1.830186000
O	-0.046935000	4.293459000	0.475923000
H	-0.071065000	3.717528000	1.279425000
H	0.740879000	3.992259000	-0.022351000

O	-0.039806000	-4.172565000	1.061171000
H	0.813929000	-4.119524000	0.489237000
H	0.036425000	-4.993863000	1.569208000
O	2.342407000	1.687807000	3.022073000
H	2.340198000	0.709801000	3.141505000
H	2.813788000	1.822324000	2.173153000
O	2.305918000	-1.085373000	3.187973000
H	1.382643000	-1.324068000	2.877459000
H	2.413729000	-1.495445000	4.059008000
O	3.867239000	2.198955000	0.663548000
H	3.276407000	2.710420000	0.045997000
H	4.560937000	2.814736000	0.944083000
O	3.760061000	-2.313448000	1.085547000
H	3.295154000	-1.904496000	1.846958000
H	3.972270000	-1.560502000	0.495335000
O	2.248725000	3.540871000	-1.028063000
H	2.564120000	4.350639000	-1.456908000
H	1.942778000	2.934598000	-1.769818000
O	2.111210000	-3.949768000	-0.322820000
H	1.902603000	-3.458937000	-1.151031000
H	2.742493000	-3.372159000	0.187218000
O	-2.611216000	1.431690000	2.822627000
H	-1.702627000	1.776932000	2.679066000
H	-3.147771000	1.735449000	2.019357000
O	-2.520409000	-1.077147000	2.910746000
H	-2.566774000	-0.024278000	2.840948000
H	-2.802522000	-1.281753000	3.815193000
O	-3.970031000	2.209506000	0.696835000
H	-3.326233000	2.673027000	0.120304000
H	-4.241752000	1.415384000	0.163220000

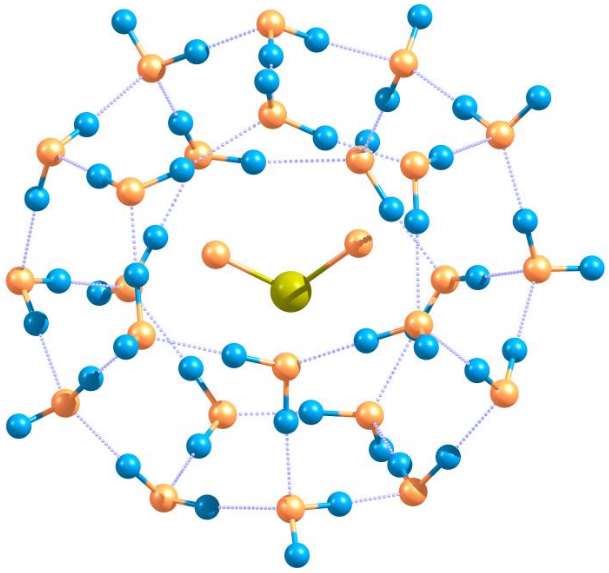

O	-3.885880000	-2.177114000	0.862601000
H	-3.427657000	-1.774202000	1.649697000
H	-4.716374000	-2.548688000	1.194522000
O	-2.153033000	3.627837000	-0.967028000
H	-2.599471000	4.465839000	-1.160105000
H	-1.354288000	3.876051000	-0.402272000
O	-2.235431000	-3.880505000	-0.668289000
H	-1.490237000	-4.075577000	-0.059936000
H	-2.837589000	-3.306378000	-0.147481000
O	2.830209000	-0.233000000	-2.914026000
H	2.298909000	-1.067522000	-2.852981000
H	3.397493000	-0.230196000	-2.110000000
O	-2.704379000	-0.161026000	-2.913726000
H	-2.194424000	0.706675000	-2.982764000
H	-3.216901000	-0.218490000	-3.734256000
O	4.431249000	-0.147505000	-0.623318000
H	4.250660000	0.690762000	-0.126474000
H	5.386690000	-0.163930000	-0.783623000
O	-4.588744000	0.011251000	-0.807060000
H	-4.341744000	-0.751098000	-0.243089000
H	-3.921429000	-0.008441000	-1.527439000
O	1.429821000	1.921820000	-2.963275000
H	1.943656000	1.033251000	-2.912432000
H	1.576454000	2.239813000	-3.867840000
O	-1.386937000	2.113197000	-3.132886000
H	-0.433042000	1.952633000	-2.976607000
H	-1.679350000	2.679060000	-2.376510000
O	1.418085000	-2.556935000	-2.646899000
H	0.397238000	-2.461869000	-2.642674000
H	1.603946000	-3.152050000	-3.389606000

O	-1.146755000	-2.440495000	-2.670488000
H	-1.668322000	-1.608744000	-2.754582000
H	-1.586109000	-2.981222000	-1.955813000
S	0.119314000	-0.377399000	-0.124396000
O	-1.162983000	0.317925000	0.026225000
O	1.332715000	0.413937000	0.145597000

Table S21. Cartesian coordinates for optimized geometries of the Greenhouse gases encapsulated within $5^{12}6^4$ clathrate hydrate computes at B3LYP/6-31G(d) level of theory and basis set.

CCl_4 within $5^{12}6^4$ clathrate hydrate

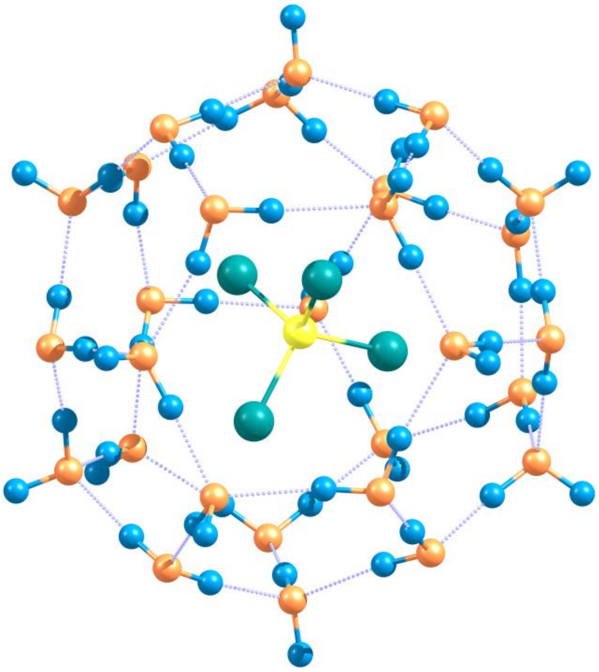

O	2.397120000	-2.199202000	-3.195348000
H	1.429646000	-2.334345000	-3.305665000
H	2.603065000	-2.517130000	-2.286901000
O	-2.468923000	-0.942612000	-3.897123000
H	-2.098115000	-0.037821000	-4.019918000
H	-1.699302000	-1.548359000	-3.871743000
O	-1.485214000	1.673547000	-4.048812000
H	-1.678759000	2.116629000	-4.889079000
H	-0.486529000	1.815616000	-3.911346000
O	1.046008000	2.235090000	-3.777356000
H	1.159905000	2.875925000	-3.026093000
H	1.699899000	1.521979000	-3.614958000
O	-2.798968000	3.082888000	-2.034800000
H	-3.328633000	2.412920000	-1.540246000
H	-2.321817000	2.576854000	-2.736092000
O	-4.324342000	1.255487000	-0.595475000
H	-4.120205000	1.390777000	0.387631000
H	-5.251803000	1.522437000	-0.688360000
O	-1.216383000	4.397785000	-0.453531000
H	-1.343458000	5.308624000	-0.762013000
H	-1.820311000	3.840089000	-1.062597000
O	2.789317000	2.411417000	2.510386000
H	3.380995000	2.888872000	3.111726000
H	3.028765000	2.724004000	1.596572000

O	3.498218000	3.149325000	-0.011801000
H	4.138761000	3.866122000	-0.136605000
H	3.912046000	2.341787000	-0.459361000
O	1.322841000	3.973869000	-1.685736000
H	0.490377000	4.026883000	-1.169584000
H	2.004406000	3.647455000	-1.062094000
O	-1.899597000	3.806422000	2.100531000
H	-1.654807000	4.094866000	1.176706000
H	-2.279073000	4.585409000	2.534496000
O	4.594178000	1.047886000	-1.163114000
H	4.606714000	0.251623000	-0.583109000
H	4.131064000	0.776735000	-1.993477000
O	4.515173000	-1.171589000	0.562603000
H	4.062125000	-0.839295000	1.420203000
H	5.407358000	-1.423564000	0.847455000
O	3.140136000	0.327177000	-3.413285000
H	3.642156000	0.351475000	-4.242161000
H	2.820875000	-0.627196000	-3.326887000
O	-2.725026000	-2.772978000	2.548620000
H	-2.829411000	-2.793861000	1.573818000
H	-1.813411000	-3.130123000	2.717539000
O	-3.212195000	-3.129254000	-0.245457000
H	-3.564691000	-2.360732000	-0.828561000
H	-3.973153000	-3.720909000	-0.138203000
O	-4.192374000	-1.286752000	-1.771203000
H	-4.206920000	-0.412451000	-1.317080000
H	-3.614874000	-1.155236000	-2.566569000
O	-3.901806000	1.755742000	1.952318000
H	-3.531882000	0.987661000	2.452761000
H	-3.219480000	2.457420000	2.024010000

O	-1.086311000	-4.268054000	-1.385657000
H	-1.884492000	-3.838225000	-0.950833000
H	-1.353561000	-5.184024000	-1.557887000
O	-0.333879000	-2.835591000	-3.596127000
H	-0.616798000	-3.357115000	-2.798062000
H	-0.317814000	-3.473071000	-4.326466000
O	0.983856000	-4.634341000	0.612658000
H	0.265469000	-4.477516000	-0.034143000
H	1.703438000	-4.048325000	0.298447000
O	3.094589000	-3.154045000	-0.641444000
H	3.595647000	-2.441921000	-0.157412000
H	3.738469000	-3.862610000	-0.793762000
O	-0.160215000	-3.663091000	2.953649000
H	0.450999000	-3.000580000	3.343757000
H	0.314402000	-4.068003000	2.186543000
O	1.433160000	-1.559846000	4.039419000
H	1.727434000	-1.791798000	4.934063000
H	0.740729000	-0.827668000	4.192328000
O	3.536349000	-0.283315000	2.785261000
H	2.780583000	-0.780753000	3.189356000
H	3.218262000	0.641818000	2.679027000
O	-0.337258000	0.284024000	4.570862000
H	-0.119989000	1.216067000	4.313736000
H	-1.202914000	0.086352000	4.146228000
O	0.281742000	2.871926000	3.795192000
H	1.068756000	2.747260000	3.225150000
H	-0.418501000	3.210958000	3.201423000
O	-2.846420000	-0.321965000	3.463778000
H	-2.785247000	-1.254582000	3.062365000
H	-3.416353000	-0.433713000	4.240247000

C	0.021430000	-0.017958000	0.019533000
Cl	-0.989821000	-0.291770000	-1.427122000
Cl	1.696147000	0.369298000	-0.487509000
Cl	-0.633002000	1.354078000	0.958099000
Cl	0.017922000	-1.480756000	1.036086000

CF_2Cl_2 within $5^{12}6^4$ clathrate hydrate

O	0.866520000	0.182238000	-4.493298000
H	-0.095727000	0.119882000	-4.299355000
H	1.269235000	-0.612465000	-4.073204000
O	-3.541646000	1.590982000	-2.421390000
H	-3.030567000	2.373579000	-2.110017000
H	-2.926630000	1.082639000	-2.989506000
O	-2.137583000	3.786448000	-1.454305000
H	-2.497815000	4.605559000	-1.827097000
H	-1.132583000	3.848551000	-1.626791000
O	0.397464000	4.116568000	-1.893159000
H	0.891777000	4.201462000	-1.033416000
H	0.857596000	3.399212000	-2.381257000
O	-2.595829000	3.737697000	1.260119000
H	-3.110318000	2.912436000	1.433466000
H	-2.454312000	3.761346000	0.281622000
O	-4.024465000	1.419543000	1.803030000
H	-3.469519000	0.900086000	2.478784000
H	-4.823448000	1.674762000	2.289472000
O	-0.300498000	3.734707000	2.432838000
H	-0.292742000	4.603006000	2.864857000
H	-1.206518000	3.700138000	1.950153000
O	4.063633000	0.351352000	2.205794000

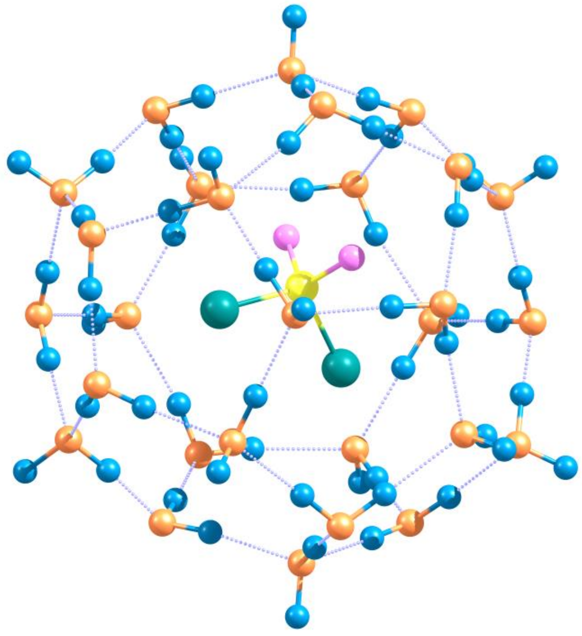

H	4.826996000	0.435023000	2.796957000
H	4.045553000	1.183858000	1.659038000
O	3.958595000	2.525260000	0.596378000
H	4.677748000	3.174060000	0.642412000
H	4.013052000	2.135190000	-0.340545000
O	1.683783000	4.254301000	0.498768000
H	1.020610000	3.971775000	1.165361000
H	2.411748000	3.600750000	0.559061000
O	-0.249994000	1.625633000	4.095247000
H	-0.232538000	2.424489000	3.496547000
H	-0.277039000	1.974051000	4.999201000
O	4.102026000	1.515521000	-1.799724000
H	4.155812000	0.532411000	-1.841408000
H	3.340403000	1.776373000	-2.374514000
O	4.183008000	-1.267549000	-1.842615000
H	4.154541000	-1.558266000	-0.855436000
H	5.037051000	-1.588649000	-2.170825000
O	1.952706000	2.272974000	-3.342039000
H	2.252259000	2.814809000	-4.088129000
H	1.498134000	1.473020000	-3.766320000
O	-2.370444000	-3.641909000	1.185123000
H	-2.753051000	-3.063722000	0.493289000
H	-1.553934000	-4.060614000	0.781868000
O	-3.846193000	-2.280006000	-0.813391000
H	-4.201672000	-1.324877000	-0.667167000
H	-4.618530000	-2.857818000	-0.714686000
O	-4.802811000	0.093066000	-0.529270000
H	-4.532958000	0.513618000	0.320456000
H	-4.405551000	0.670523000	-1.233770000
O	-2.641007000	0.219424000	3.658438000

H	-2.355567000	-0.710570000	3.479822000
H	-1.800487000	0.708486000	3.802476000
O	-2.484092000	-2.484893000	-3.069379000
H	-3.031309000	-2.413964000	-2.226557000
H	-2.940975000	-3.131660000	-3.628107000
O	-1.909568000	-0.039043000	-4.086367000
H	-2.136669000	-0.945726000	-3.745144000
H	-2.312393000	0.014390000	-4.966639000
O	-0.026409000	-3.822247000	-2.479589000
H	-0.863285000	-3.337160000	-2.630252000
H	0.668837000	-3.135782000	-2.539926000
O	2.013443000	-2.040255000	-3.260960000
H	2.812653000	-1.797891000	-2.716988000
H	2.324360000	-2.685517000	-3.914051000
O	-0.134591000	-4.691252000	0.140867000
H	0.636041000	-4.246493000	0.554923000
H	-0.076901000	-4.450252000	-0.816042000
O	2.115843000	-3.519876000	1.436811000
H	2.521226000	-4.333150000	1.777069000
H	1.697483000	-3.093807000	2.268410000
O	4.221883000	-1.964744000	0.631744000
H	3.442343000	-2.520288000	0.895915000
H	4.170262000	-1.159344000	1.194831000
O	1.078001000	-2.551630000	3.599683000
H	1.429081000	-1.653858000	3.841611000
H	0.105942000	-2.437701000	3.488205000
O	2.027015000	-0.051164000	4.139954000
H	2.634672000	0.149338000	3.398287000
H	1.261634000	0.552049000	4.030423000
O	-1.688438000	-2.332565000	3.305004000

H	-1.950442000	-2.818181000	2.443229000
H	-2.044602000	-2.890618000	4.013389000
C	-0.021244000	-0.391213000	0.350645000
Cl	-1.451137000	0.344463000	-0.410729000
Cl	1.488705000	0.304650000	-0.303972000
F	-0.060448000	-0.187175000	1.670856000
F	-0.030990000	-1.707804000	0.127761000

CH_3Br within $5^{12}6^4$ clathrate hydrate

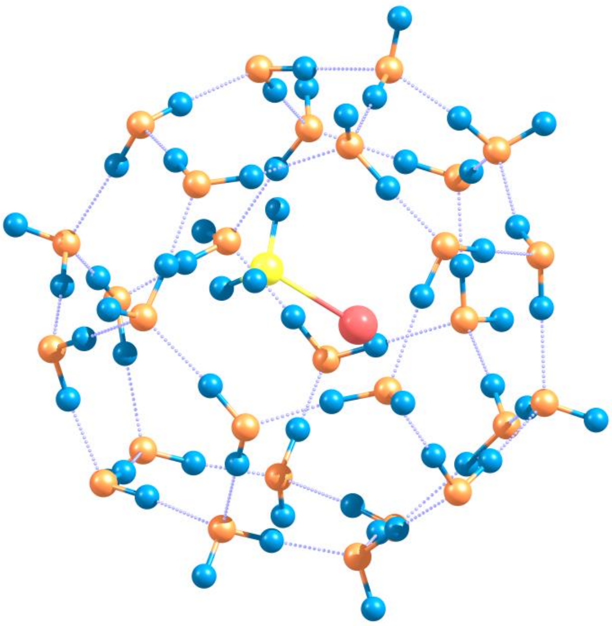

O	4.106623000	-1.945974000	0.694377000
H	3.354883000	-2.429247000	1.106836000
H	4.081151000	-1.052146000	1.102788000
O	-0.263541000	-4.444810000	0.879835000
H	-0.352404000	-4.303623000	-0.091472000
H	0.510586000	-3.904988000	1.141722000
O	-0.513353000	-4.062292000	-1.855660000
H	-0.437583000	-4.918033000	-2.304534000
H	0.246625000	-3.504907000	-2.239232000
O	1.382408000	-2.735961000	-3.018247000
H	1.005550000	-1.975369000	-3.540555000
H	2.182103000	-2.389546000	-2.565121000
O	-2.931619000	-2.875986000	-2.407951000
H	-3.305407000	-2.675633000	-1.515596000
H	-2.065939000	-3.318919000	-2.225675000
O	-3.971001000	-2.276868000	0.084720000
H	-4.235751000	-1.295363000	0.080567000
H	-4.809988000	-2.754233000	0.175290000
O	-2.479453000	-0.681133000	-3.676878000
H	-2.816867000	-0.881138000	-4.563838000

H	-2.640916000	-1.543979000	-3.141851000
O	0.437067000	3.709724000	-2.493303000
H	0.514647000	4.494617000	-3.056422000
H	0.866629000	2.963768000	-3.002553000
O	1.666109000	1.697087000	-3.751512000
H	2.004265000	1.861992000	-4.645115000
H	2.487102000	1.417991000	-3.209174000
O	0.256510000	-0.663852000	-4.330942000
H	-0.667195000	-0.620320000	-4.000303000
H	0.684284000	0.159276000	-4.011714000
O	-3.729156000	1.312282000	-2.391030000
H	-3.251680000	0.594721000	-2.898166000
H	-4.463643000	1.589754000	-2.959130000
O	3.804728000	1.030373000	-2.454320000
H	3.807086000	1.484752000	-1.581399000
H	3.763039000	0.063699000	-2.247053000
O	3.856289000	2.431298000	-0.031442000
H	3.029884000	3.034421000	-0.026443000
H	4.603432000	3.044295000	-0.109442000
O	3.709971000	-1.663444000	-1.885229000
H	4.478883000	-2.115720000	-2.264984000
H	3.808244000	-1.778825000	-0.884022000
O	-2.268014000	0.950675000	3.653712000
H	-1.972916000	0.018071000	3.582562000
H	-1.441743000	1.494317000	3.809574000
O	-1.488194000	-1.738870000	3.815995000
H	-1.855386000	-2.385400000	3.087929000
H	-2.012817000	-1.934745000	4.607751000
O	-2.403376000	-3.381279000	2.106244000
H	-2.918032000	-2.927504000	1.399065000

H	-1.634980000	-3.821257000	1.639650000
O	-4.777035000	0.200768000	-0.043182000
H	-4.428203000	0.792727000	0.667600000
H	-4.426581000	0.591364000	-0.875350000
O	1.123760000	-1.961248000	4.126871000
H	0.121665000	-1.891902000	4.034086000
H	1.285220000	-2.286900000	5.025168000
O	2.074674000	-3.347382000	1.996664000
H	1.760481000	-2.872775000	2.810295000
H	2.389210000	-4.212404000	2.300610000
O	2.112718000	0.695563000	3.950770000
H	1.790150000	-0.227269000	4.013283000
H	2.781642000	0.688838000	3.234598000
O	4.069385000	0.584391000	1.915591000
H	3.978626000	1.301696000	1.228636000
H	4.926039000	0.737197000	2.342292000
O	-0.058352000	2.417076000	3.975159000
H	0.014926000	3.023117000	3.206473000
H	0.757663000	1.863550000	3.965953000
O	-0.018225000	4.215250000	1.804568000
H	0.003442000	5.082152000	2.238672000
H	-0.952952000	4.165513000	1.383356000
O	1.865451000	4.061501000	-0.149292000
H	1.201098000	4.127777000	0.584879000
H	1.343592000	3.957468000	-0.978602000
O	-2.390312000	4.152716000	0.787555000
H	-2.392958000	4.039739000	-0.201214000
H	-2.887564000	3.378124000	1.138988000
O	-2.329316000	3.722332000	-1.900930000
H	-1.382444000	3.598968000	-2.120348000

H	-2.752193000	2.851390000	-2.060542000
O	-3.773346000	1.959365000	1.819702000
H	-3.167181000	1.527918000	2.519472000
H	-4.507710000	2.334381000	2.329778000
C	0.316265000	0.755432000	0.838479000
H	0.917764000	0.521680000	1.714358000
H	0.583996000	1.728140000	0.431154000
H	-0.744829000	0.701168000	1.073771000
Br	0.707805000	-0.589584000	-0.556084000

CH_3Cl within $5^{12}6^4$ clathrate hydrate

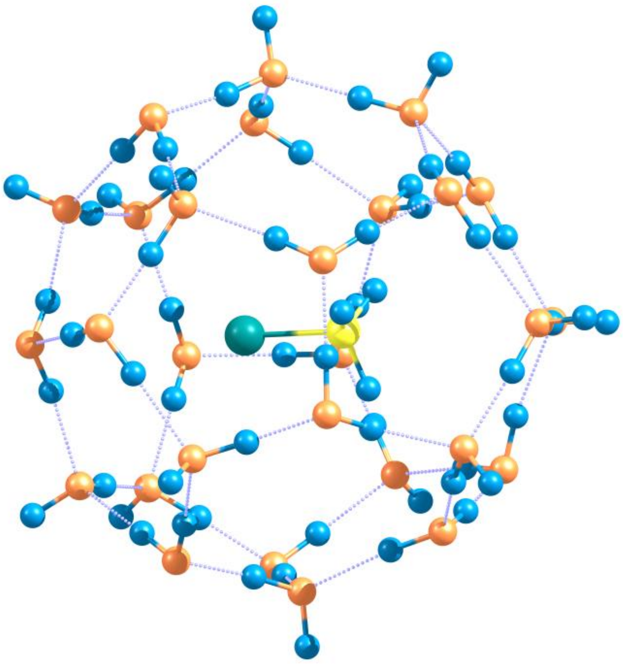

O	0.587330000	2.575380000	-3.529876000
H	1.308103000	1.908407000	-3.569082000
H	0.774536000	3.139084000	-2.744795000
O	1.961206000	-2.077583000	-3.557180000
H	0.988631000	-2.239917000	-3.543982000
H	2.072852000	-1.105887000	-3.587362000
O	-0.738203000	-2.721456000	-3.537114000
H	-0.879803000	-3.242107000	-4.342652000
H	-1.457474000	-1.994152000	-3.575847000
O	-2.631233000	-0.968279000	-3.716349000
H	-3.259728000	-1.085297000	-2.950769000
H	-2.310493000	-0.043156000	-3.641164000
O	-1.235539000	-4.368885000	-1.384648000
H	-0.397647000	-4.351725000	-0.861407000
H	-1.059630000	-3.786578000	-2.164570000
O	1.104070000	-4.294092000	0.088087000
H	0.879749000	-4.047680000	1.053413000
H	1.450961000	-5.197921000	0.140590000

O	-3.143631000	-3.335424000	0.011507000
H	-3.851179000	-3.992156000	-0.081815000
H	-2.367771000	-3.717833000	-0.541223000
O	-3.559691000	1.593574000	2.309138000
H	-4.323955000	1.849386000	2.847017000
H	-3.913106000	1.425350000	1.391395000
O	-4.437822000	1.231838000	-0.209833000
H	-5.381719000	1.392678000	-0.363396000
H	-3.961700000	1.942467000	-0.766537000
O	-4.234676000	-1.257976000	-1.551889000
H	-3.805124000	-1.919092000	-0.965876000
H	-4.211374000	-0.415594000	-1.049555000
O	-2.208273000	-3.021803000	2.517916000
H	-2.582918000	-3.121295000	1.597878000
H	-2.713476000	-3.632026000	3.075879000
O	-3.284650000	3.076605000	-1.631657000
H	-2.588166000	3.524148000	-1.097003000
H	-2.796357000	2.617416000	-2.357690000
O	-1.330239000	4.380482000	-0.116284000
H	-1.409025000	4.021810000	0.850993000
H	-1.632912000	5.299688000	-0.051372000
O	-1.903584000	1.747757000	-3.618832000
H	-2.194360000	2.073866000	-4.484223000
H	-0.929889000	2.004528000	-3.561516000
O	3.835119000	-0.524882000	2.544478000
H	4.002693000	-0.762785000	1.608776000
H	3.622135000	0.449329000	2.519462000
O	4.504796000	-1.083046000	-0.156901000
H	3.896283000	-1.805835000	-0.572347000
H	5.371318000	-1.506956000	-0.058914000

O	3.057207000	-2.873955000	-1.243047000
H	2.353589000	-3.326027000	-0.719919000
H	2.648807000	-2.623202000	-2.119507000
O	0.480533000	-3.793414000	2.553613000
H	0.988888000	-3.032900000	2.930643000
H	-0.457941000	-3.497281000	2.557171000
O	4.410683000	1.078105000	-1.681157000
H	4.488659000	0.259377000	-1.098619000
H	5.319525000	1.349162000	-1.881109000
O	2.630379000	0.681779000	-3.679962000
H	3.334012000	0.819132000	-2.990347000
H	3.083605000	0.731139000	-4.535313000
O	3.467783000	3.254943000	-0.061423000
H	3.755666000	2.506088000	-0.621686000
H	2.587940000	3.507791000	-0.410313000
O	1.069415000	4.133777000	-1.286610000
H	0.205446000	4.230390000	-0.791067000
H	1.328397000	5.033618000	-1.536982000
O	3.128796000	2.056627000	2.393022000
H	2.215489000	2.257379000	2.695568000
H	3.266201000	2.554361000	1.551133000
O	0.585401000	2.550281000	3.452996000
H	0.798234000	3.091283000	4.229599000
H	0.343167000	1.634839000	3.851446000
O	-1.643303000	3.611052000	2.299608000
H	-0.822050000	3.220589000	2.699778000
H	-2.310771000	2.886545000	2.317733000
O	0.023148000	0.289172000	4.561990000
H	-0.891269000	-0.020319000	4.321725000
H	0.633147000	-0.401430000	4.214491000

O	-2.468175000	-0.531092000	3.824097000
H	-2.809242000	0.147470000	3.205383000
H	-2.371698000	-1.350085000	3.293510000
O	1.813628000	-1.681996000	3.697649000
H	2.580560000	-1.248059000	3.185873000
H	2.218314000	-1.985119000	4.524791000
C	1.075457000	0.297625000	-0.209757000
H	0.850462000	0.947501000	-1.056852000
H	1.742298000	0.792742000	0.497399000
H	1.503047000	-0.644674000	-0.555335000
Cl	-0.476381000	-0.066245000	0.648020000

CH_4 within $5^{12}6^4$ clathrate hydrate

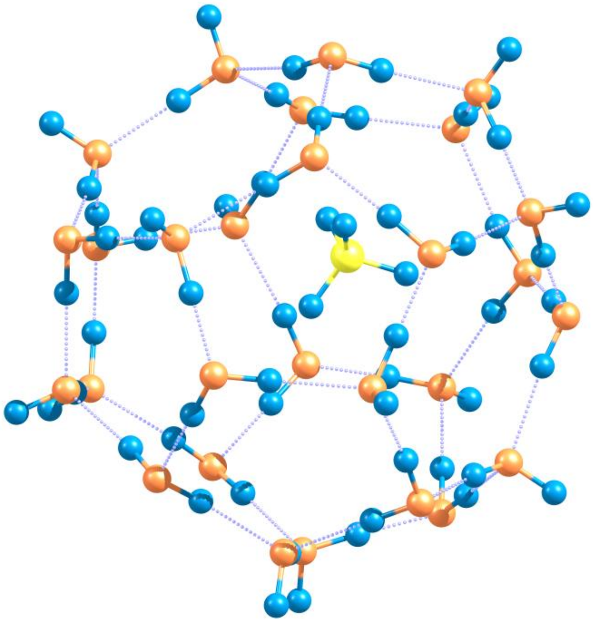

O	0.294231000	2.572265000	-3.607449000
H	1.155565000	2.107339000	-3.509434000
H	0.216888000	3.165562000	-2.825519000
O	2.888780000	-1.548134000	-3.128669000
H	2.002845000	-1.972631000	-3.201311000
H	2.731295000	-0.584684000	-3.204014000
O	0.479210000	-2.925740000	-3.372475000
H	0.600225000	-3.472735000	-4.163761000
H	-0.390184000	-2.417085000	-3.545615000
O	-1.766167000	-1.740118000	-3.886905000
H	-2.440055000	-2.005427000	-3.203218000
H	-1.716677000	-0.760740000	-3.829061000
O	0.159032000	-4.580909000	-1.200033000
H	0.900185000	-4.332223000	-0.595115000
H	0.275195000	-4.003208000	-1.995181000
O	2.210274000	-3.848445000	0.498923000

H	1.816064000	-3.638803000	1.414422000
H	2.772504000	-4.624444000	0.646449000
O	-2.109002000	-4.058018000	-0.100720000
H	-2.602854000	-4.882444000	-0.232082000
H	-1.194579000	-4.238002000	-0.536489000
O	-4.147121000	0.600996000	1.847423000
H	-5.013596000	0.649591000	2.278699000
H	-4.325035000	0.319993000	0.906227000
O	-4.551589000	-0.051839000	-0.732089000
H	-5.476461000	-0.165967000	-1.000761000
H	-4.222039000	0.747073000	-1.278987000
O	-3.511087000	-2.412712000	-1.916877000
H	-3.003593000	-2.919057000	-1.245598000
H	-3.795076000	-1.592029000	-1.460919000
O	-1.618520000	-3.440187000	2.472818000
H	-1.838705000	-3.670026000	1.526038000
H	-1.990801000	-4.155496000	3.010343000
O	-3.774020000	1.987569000	-2.130203000
H	-3.317127000	2.645222000	-1.555312000
H	-3.084331000	1.675022000	-2.766280000
O	-2.494659000	3.850299000	-0.493308000
H	-2.601293000	3.522720000	0.478577000
H	-3.033417000	4.655798000	-0.532387000
O	-1.838936000	1.069959000	-3.864649000
H	-2.116078000	1.262803000	-4.773525000
H	-0.986488000	1.599116000	-3.738696000
O	3.358224000	0.624227000	2.992129000
H	3.681733000	0.408149000	2.092124000
H	2.918808000	1.518419000	2.902331000
O	4.541088000	0.149294000	0.465453000

H	4.265887000	-0.736313000	0.011891000
H	5.461186000	0.016719000	0.741003000
O	3.943989000	-2.040869000	-0.704065000
H	3.295487000	-2.618627000	-0.238958000
H	3.573058000	-1.892716000	-1.619118000
O	1.163302000	-3.453618000	2.846663000
H	1.380527000	-2.569961000	3.236427000
H	0.186867000	-3.440769000	2.731084000
O	4.090802000	2.165431000	-1.190735000
H	4.289150000	1.407046000	-0.557882000
H	4.923429000	2.650898000	-1.291971000
O	2.758025000	1.278327000	-3.375912000
H	3.283232000	1.612435000	-2.599801000
H	3.310426000	1.438160000	-4.156151000
O	2.389882000	4.017549000	0.178806000
H	2.942839000	3.359782000	-0.290281000
H	1.525995000	3.990122000	-0.281587000
O	0.026883000	4.212099000	-1.379209000
H	-0.884714000	4.089313000	-0.992394000
H	0.091192000	5.152158000	-1.605940000
O	2.084215000	2.959346000	2.704335000
H	1.117959000	2.849734000	2.836137000
H	2.192277000	3.392874000	1.822650000
O	-0.642305000	2.682494000	3.376782000
H	-0.643939000	3.296120000	4.128122000
H	-0.683702000	1.757770000	3.816318000
O	-2.906818000	3.099645000	1.923213000
H	-2.072440000	2.962930000	2.445245000
H	-3.350909000	2.220959000	1.914704000
O	-0.716866000	0.393731000	4.580697000

H	-1.476474000	-0.173348000	4.282048000
H	0.097529000	-0.111701000	4.351867000
O	-2.775428000	-1.128997000	3.627348000
H	-3.191362000	-0.578417000	2.931914000
H	-2.374176000	-1.889336000	3.155535000
O	1.628118000	-1.008712000	4.001804000
H	2.294373000	-0.381912000	3.541765000
H	2.014191000	-1.161823000	4.877989000
C	0.921246000	0.512606000	-0.271699000
H	0.050049000	0.005062000	0.152411000
H	0.613394000	1.083439000	-1.152830000
H	1.664736000	-0.234403000	-0.564377000
H	1.345239000	1.186902000	0.477675000

CO_2 within $5^{12}6^4$ clathrate hydrate

O	-1.434119000	-2.422328000	-3.534817000
H	-0.449937000	-2.394517000	-3.518677000
H	-1.717912000	-1.483195000	-3.622811000
O	3.084999000	-3.089743000	-1.419638000
H	2.617975000	-3.446069000	-0.629149000
H	2.374918000	-2.787640000	-2.021878000
O	1.821895000	-4.129239000	0.844009000
H	2.128830000	-5.038609000	0.980390000
H	0.811300000	-4.208665000	0.754855000
O	-0.765213000	-4.434874000	0.739226000
H	-1.136337000	-3.891155000	1.482803000
H	-1.215791000	-4.080884000	-0.057473000
O	2.499296000	-2.582437000	3.017248000
H	3.062610000	-1.861015000	2.644071000

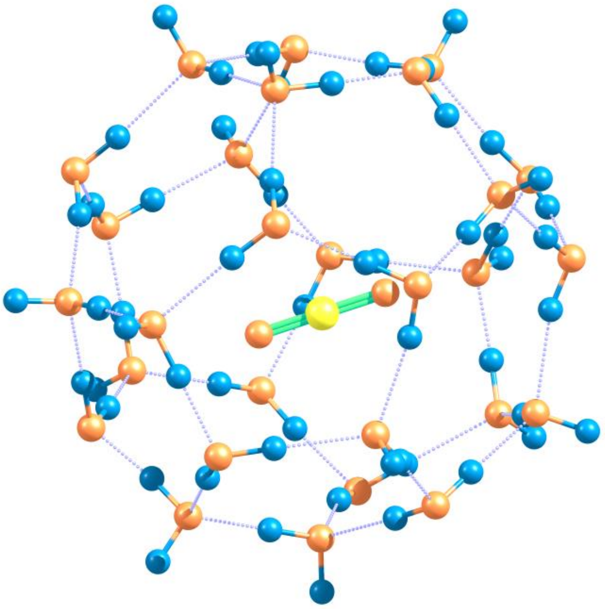

H	2.252031000	-3.146493000	2.242400000
O	4.031914000	-0.527881000	1.991295000
H	3.671594000	0.361859000	2.328681000
H	4.921448000	-0.590321000	2.371861000
O	0.400390000	-1.571825000	4.100325000
H	0.326381000	-2.068384000	4.930210000
H	1.235098000	-1.965370000	3.641784000
O	-3.578631000	1.475450000	2.332427000
H	-4.309706000	1.813450000	2.871355000
H	-3.703171000	0.487132000	2.292891000
O	-3.933017000	-1.190238000	2.112886000
H	-4.672807000	-1.601582000	2.585574000
H	-4.105786000	-1.389887000	1.126084000
O	-1.688804000	-2.790227000	2.681179000
H	-0.999737000	-2.264569000	3.140760000
H	-2.436377000	-2.182649000	2.496121000
O	0.778426000	1.075793000	4.355761000
H	0.618506000	0.091666000	4.298920000
H	0.891524000	1.271805000	5.298057000
O	-4.407521000	-1.726662000	-0.384344000
H	-4.310939000	-0.900678000	-0.913792000
H	-3.690308000	-2.322223000	-0.709642000
O	-4.127277000	0.612010000	-1.881172000
H	-3.932741000	1.379956000	-1.220734000
H	-4.996107000	0.830392000	-2.252683000
O	-2.403801000	-3.416440000	-1.320185000
H	-2.872769000	-4.218980000	-1.596853000
H	-2.004451000	-3.041015000	-2.173554000
O	2.792888000	3.486039000	-1.205114000
H	3.023859000	2.571549000	-1.472222000

H	1.965635000	3.714913000	-1.723446000
O	3.747463000	1.032108000	-2.226142000
H	4.012291000	0.254426000	-1.592938000
H	4.590820000	1.418747000	-2.508509000
O	4.489839000	-0.894776000	-0.731064000
H	4.275112000	-0.729167000	0.215880000
H	4.002315000	-1.732955000	-0.973699000
O	3.157805000	1.705243000	3.008740000
H	2.901460000	2.404474000	2.356944000
H	2.326082000	1.504105000	3.492898000
O	2.202001000	0.171958000	-4.179732000
H	2.817824000	0.507474000	-3.454086000
H	2.681307000	0.297063000	-5.012716000
O	1.350114000	-2.316735000	-3.530948000
H	1.676953000	-1.418782000	-3.807560000
H	1.720463000	-2.938894000	-4.175587000
O	-0.021609000	1.967881000	-4.280983000
H	0.748928000	1.365275000	-4.237335000
H	-0.763648000	1.433825000	-3.931053000
O	-2.207969000	0.244442000	-3.725126000
H	-2.913834000	0.434487000	-3.044084000
H	-2.625308000	0.404873000	-4.585271000
O	0.518932000	4.027869000	-2.509056000
H	-0.206845000	3.964362000	-1.851097000
H	0.315630000	3.333332000	-3.182360000
O	-1.568069000	4.074184000	-0.596101000
H	-1.883741000	4.970659000	-0.790917000
H	-1.093753000	4.174001000	0.305904000
O	-3.800915000	2.543434000	-0.229911000
H	-2.978563000	3.077165000	-0.386591000

H	-3.703085000	2.176372000	0.678757000
O	-0.365456000	4.427086000	1.670074000
H	-0.736837000	3.854040000	2.392293000
H	0.575185000	4.151958000	1.569778000
O	-1.372832000	2.745938000	3.574645000
H	-2.057297000	2.209964000	3.122747000
H	-0.651691000	2.120129000	3.797477000
O	2.312491000	3.704260000	1.324768000
H	2.486829000	3.577843000	0.323197000
H	2.803954000	4.508483000	1.552600000
C	-0.629078000	-0.942454000	0.012295000
O	0.349921000	-1.530577000	-0.233379000
O	-1.599753000	-0.333134000	0.244837000

CO within $5^{12}6^4$ clathrate hydrate

O	0.754672000	1.217387000	-4.244087000
H	1.636467000	1.037590000	-3.848593000
H	0.376825000	1.964837000	-3.725933000
O	3.813870000	-1.704968000	-1.730855000
H	3.081295000	-2.359262000	-1.811389000
H	3.543186000	-0.920390000	-2.248420000
O	1.863664000	-3.678093000	-1.829458000
H	2.245004000	-4.423826000	-2.317873000
H	0.991659000	-3.468952000	-2.320398000
O	-0.349980000	-3.289810000	-3.113235000
H	-1.120672000	-3.476802000	-2.511332000
H	-0.482469000	-2.365782000	-3.418033000
O	1.269823000	-4.499983000	0.726920000
H	1.775175000	-3.880975000	1.308086000

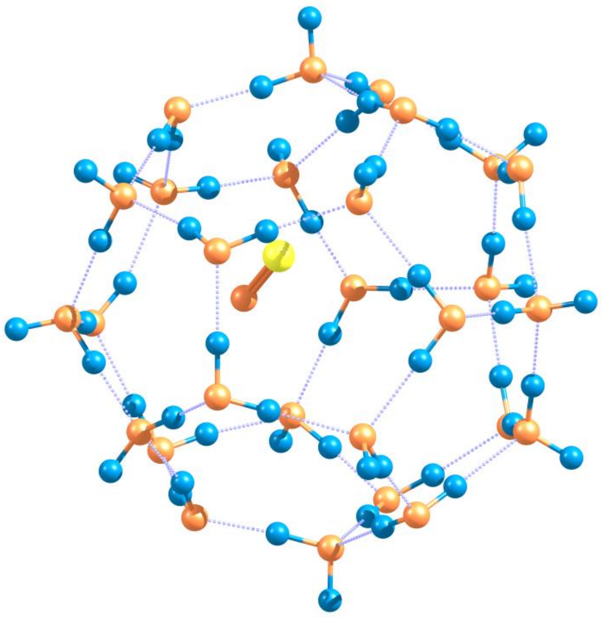

H	1.491366000	-4.222604000	-0.197157000
O	2.648413000	-2.734614000	2.346611000
H	2.002519000	-2.320225000	3.015931000
H	3.268270000	-3.245240000	2.889826000
O	-1.258122000	-4.236307000	1.135417000
H	-1.551670000	-5.154154000	1.245795000
H	-0.246880000	-4.312251000	0.963425000
O	-4.544096000	0.099966000	0.751680000
H	-5.488777000	0.066126000	0.965163000
H	-4.416793000	-0.513551000	-0.025537000
O	-4.134647000	-1.459290000	-1.403420000
H	-4.919160000	-1.902644000	-1.762027000
H	-3.824320000	-0.838628000	-2.154935000
O	-2.408085000	-3.712562000	-1.388458000
H	-2.012316000	-3.815753000	-0.495698000
H	-2.945202000	-2.893423000	-1.339444000
O	-1.569450000	-2.681459000	3.310213000
H	-1.491622000	-3.267230000	2.504872000
H	-1.927359000	-3.245915000	4.012009000
O	-3.403133000	0.111467000	-3.329880000
H	-3.244079000	1.020280000	-2.983278000
H	-2.526340000	-0.195780000	-3.669128000
O	-2.950560000	2.673768000	-2.324789000
H	-3.243855000	2.682435000	-1.336308000
H	-3.578024000	3.266537000	-2.766783000
O	-0.952001000	-0.777814000	-4.222182000
H	-1.025104000	-1.016399000	-5.159099000
H	-0.262247000	-0.039613000	-4.195552000
O	2.221047000	2.476859000	3.066010000
H	2.709551000	2.028619000	2.343859000

H	1.681047000	3.191600000	2.615104000
O	3.994424000	1.421125000	1.142834000
H	4.080026000	0.396264000	1.031815000
H	4.773916000	1.681022000	1.657986000
O	4.320477000	-1.092925000	0.848506000
H	3.686432000	-1.629968000	1.377865000
H	4.161466000	-1.347497000	-0.102347000
O	0.976611000	-1.824746000	4.120279000
H	0.914419000	-0.838836000	4.182423000
H	0.076770000	-2.113759000	3.848801000
O	3.754030000	2.589394000	-1.208531000
H	3.875939000	2.148488000	-0.309312000
H	4.497987000	3.202961000	-1.305571000
O	3.276873000	0.730782000	-3.116382000
H	3.498209000	1.427471000	-2.440093000
H	3.973766000	0.783667000	-3.787988000
O	1.471156000	4.285279000	-0.858186000
H	2.241499000	3.688129000	-0.948953000
H	0.766093000	3.844755000	-1.374860000
O	-0.364543000	3.289849000	-2.764503000
H	-1.322998000	3.098087000	-2.564541000
H	-0.361987000	4.117920000	-3.268253000
O	0.714248000	4.294503000	1.805919000
H	-0.198765000	3.934969000	1.789624000
H	0.988068000	4.343147000	0.857073000
O	-1.980179000	3.422312000	1.973849000
H	-2.312461000	4.222855000	2.409716000
H	-1.982016000	2.720387000	2.719652000
O	-3.815625000	2.709708000	0.088065000
H	-3.133498000	2.979229000	0.758499000

H	-4.083188000	1.797215000	0.343296000
O	-1.992578000	1.726387000	3.930642000
H	-2.527873000	0.912108000	3.736049000
H	-1.070865000	1.408603000	4.073893000
O	-3.402338000	-0.521831000	3.272556000
H	-3.716495000	-0.363612000	2.358252000
H	-2.755408000	-1.255618000	3.204613000
O	0.637306000	0.890889000	4.350590000
H	1.258923000	1.494039000	3.802661000
H	0.821189000	1.132739000	5.271391000
C	0.904497000	-0.324411000	-1.160278000
O	1.149771000	0.622610000	-0.578271000

H_2S within $5^{12}6^4$ clathrate hydrate

O	1.462620000	-4.434160000	-0.818654000
H	0.545481000	-4.382171000	-0.464778000
H	2.028798000	-4.060153000	-0.107031000
O	-3.355567000	-2.970623000	-0.943272000
H	-3.145309000	-2.426525000	-1.738259000
H	-2.487544000	-3.276832000	-0.610117000
O	-2.825552000	-1.457395000	-3.221094000
H	-3.323822000	-1.854071000	-3.951946000
H	-1.853366000	-1.476448000	-3.537004000
O	-0.418148000	-1.502684000	-4.176119000
H	-0.092905000	-0.565116000	-4.267912000
H	0.243911000	-1.945774000	-3.602287000
O	-3.609592000	1.140205000	-2.830723000
H	-3.900223000	1.158730000	-1.887879000
H	-3.335567000	0.202206000	-2.992310000

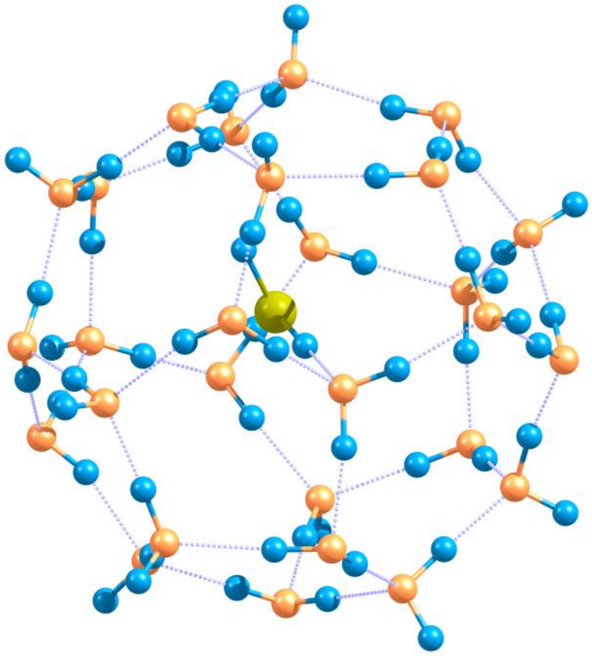

O	-4.367367000	1.206634000	-0.164544000
H	-3.802161000	1.922913000	0.270879000
H	-5.279458000	1.526364000	-0.091960000
O	-1.584697000	2.688151000	-3.216344000
H	-1.828697000	3.163536000	-4.025535000
H	-2.377671000	2.060882000	-3.036330000
O	3.447144000	2.788966000	-0.924963000
H	4.086064000	3.463704000	-1.199775000
H	3.245918000	2.255556000	-1.744903000
O	2.985758000	1.263096000	-3.090661000
H	3.499682000	1.479321000	-3.883975000
H	3.283355000	0.319458000	-2.840165000
O	0.443787000	1.065321000	-4.317977000
H	-0.218271000	1.606045000	-3.835405000
H	1.267031000	1.134471000	-3.788849000
O	-1.182445000	4.181146000	-1.025760000
H	-1.295757000	3.648464000	-1.864334000
H	-1.402006000	5.095050000	-1.261466000
O	3.810545000	-1.124933000	-2.486734000
H	3.996428000	-1.167292000	-1.520685000
H	3.061204000	-1.753760000	-2.635454000
O	4.432898000	-1.252906000	0.241482000
H	4.355135000	-0.301832000	0.625407000
H	5.388058000	-1.420897000	0.233847000
O	1.700085000	-2.843069000	-2.899518000
H	1.922140000	-3.451981000	-3.620506000
H	1.566317000	-3.437140000	-2.091983000
O	-1.445788000	0.545695000	4.167305000
H	-2.054526000	-0.094528000	3.736667000
H	-0.550825000	0.255341000	3.839659000

O	-2.952186000	-1.576212000	3.077841000
H	-3.473242000	-1.450227000	2.187690000
H	-3.642074000	-1.760354000	3.734054000
O	-4.346680000	-1.344400000	0.960107000
H	-4.299062000	-0.438188000	0.577739000
H	-4.010407000	-1.952944000	0.240693000
O	-2.958000000	3.158324000	0.848716000
H	-2.451596000	3.062967000	1.692501000
H	-2.329734000	3.551973000	0.201299000
O	-1.225416000	-3.581093000	2.821148000
H	-1.862584000	-2.807121000	2.927863000
H	-1.518443000	-4.246859000	3.461541000
O	-1.135229000	-4.311938000	0.200680000
H	-1.175039000	-4.072375000	1.163775000
H	-1.577802000	-5.171226000	0.126749000
O	1.428380000	-2.752679000	3.474231000
H	0.529000000	-3.082532000	3.263972000
H	1.970532000	-2.984199000	2.689332000
O	3.038108000	-3.339647000	1.229680000
H	3.533582000	-2.531421000	0.916950000
H	3.720276000	-3.966064000	1.514920000
O	0.830543000	-0.169602000	2.941670000
H	1.584553000	0.443528000	3.110310000
H	1.129711000	-1.086298000	3.187484000
O	2.770199000	1.755432000	3.170170000
H	3.181786000	1.944603000	4.026637000
H	2.164794000	2.563497000	2.979534000
O	4.462650000	1.173229000	1.094008000
H	3.886830000	1.380466000	1.872244000
H	4.122621000	1.752905000	0.372852000

O	1.198387000	3.759360000	2.752808000
H	1.265862000	4.054471000	1.802989000
H	0.272109000	3.445300000	2.862417000
O	1.392613000	4.423860000	0.127548000
H	2.016465000	3.770535000	-0.253793000
H	0.528756000	4.237870000	-0.297407000
O	-1.470960000	2.928604000	3.135012000
H	-1.510402000	1.986600000	3.526886000
H	-1.801998000	3.507328000	3.838630000
S	-0.312608000	0.342476000	-0.140819000
H	0.199999000	0.102744000	1.104107000
H	-1.202898000	1.254439000	0.307171000

CH_3F within $5^{12}6^4$ clathrate hydrate

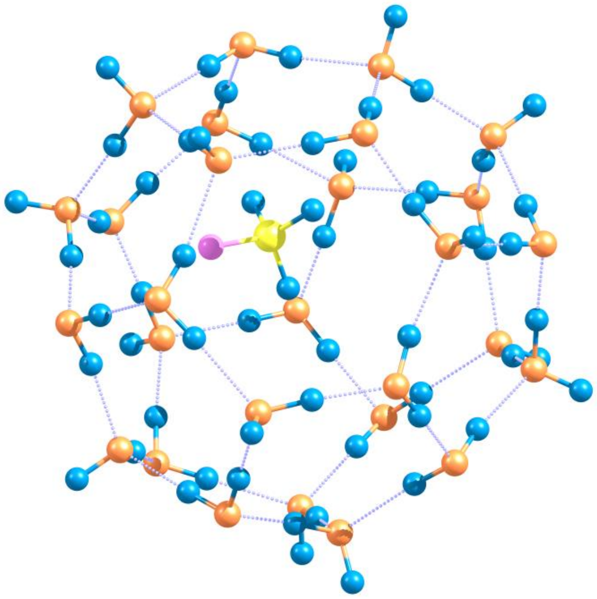

O	0.303185000	-2.533139000	-3.608221000
H	-0.640189000	-2.291840000	-3.453930000
H	0.560847000	-3.113130000	-2.857189000
O	-3.329344000	0.797790000	-2.982462000
H	-2.613291000	1.468993000	-3.048924000
H	-2.863866000	-0.063295000	-2.963613000
O	-1.369916000	2.792700000	-3.266131000
H	-1.657828000	3.285341000	-4.049850000
H	-0.417512000	2.503194000	-3.484544000
O	1.077192000	2.177423000	-3.895651000
H	1.698989000	2.607315000	-3.248375000
H	1.286798000	1.219389000	-3.848757000
O	-1.354433000	4.441768000	-1.072540000
H	-1.978940000	4.000008000	-0.445454000
H	-1.366410000	3.872550000	-1.882487000

O	-3.060286000	3.166302000	0.677307000
H	-2.586327000	3.064420000	1.570001000
H	-3.809016000	3.753763000	0.862370000
O	1.023744000	4.491011000	-0.102512000
H	1.283750000	5.415905000	-0.236202000
H	0.069082000	4.438630000	-0.488018000
O	4.297768000	0.479666000	1.661189000
H	5.164747000	0.653276000	2.057986000
H	4.354589000	0.811670000	0.720999000
O	4.383588000	1.248783000	-0.912007000
H	5.225003000	1.618255000	-1.222149000
H	4.259386000	0.394927000	-1.460499000
O	2.705547000	3.267746000	-2.008944000
H	2.120289000	3.629789000	-1.307974000
H	3.207337000	2.543629000	-1.578858000
O	0.853062000	3.739418000	2.473154000
H	0.953521000	4.031509000	1.522729000
H	1.050667000	4.521346000	3.010563000
O	4.118175000	-0.913877000	-2.317203000
H	3.873471000	-1.679139000	-1.747118000
H	3.345911000	-0.782528000	-2.921923000
O	3.439542000	-3.082300000	-0.682311000
H	3.504246000	-2.743428000	0.288694000
H	4.177298000	-3.706454000	-0.765828000
O	1.927840000	-0.507493000	-3.927157000
H	2.188901000	-0.588807000	-4.857442000
H	1.265555000	-1.258700000	-3.779056000
O	-2.900269000	-1.417191000	3.086697000
H	-3.323153000	-1.278443000	2.210827000
H	-2.267509000	-2.177433000	2.962356000

O	-4.293190000	-1.233543000	0.658216000
H	-4.283416000	-0.313942000	0.186698000
H	-5.210292000	-1.352168000	0.949762000
O	-4.430028000	1.038017000	-0.518554000
H	-3.866640000	1.708104000	-0.069447000
H	-4.055944000	0.965462000	-1.441444000
O	-1.827486000	3.055228000	2.975357000
H	-1.789979000	2.144821000	3.360790000
H	-0.887698000	3.291928000	2.811039000
O	-3.339748000	-3.086974000	-0.983164000
H	-3.685897000	-2.378862000	-0.355886000
H	-4.067302000	-3.718596000	-1.088884000
O	-2.352615000	-1.865884000	-3.199591000
H	-2.729044000	-2.302422000	-2.389719000
H	-2.913333000	-2.150840000	-3.937024000
O	-1.210323000	-4.530180000	0.236914000
H	-1.935539000	-4.028122000	-0.191253000
H	-0.408549000	-4.306948000	-0.277878000
O	1.061884000	-4.111546000	-1.435817000
H	1.935485000	-3.766875000	-1.099038000
H	1.225047000	-5.029194000	-1.701864000
O	-1.089871000	-3.374839000	2.726879000
H	-0.166605000	-3.065823000	2.849804000
H	-1.108184000	-3.858686000	1.864702000
O	1.517678000	-2.450045000	3.286382000
H	1.735342000	-3.039038000	4.026008000
H	1.337377000	-1.546782000	3.739562000
O	3.744254000	-2.253258000	1.725896000
H	2.929699000	-2.339921000	2.287619000
H	3.952646000	-1.290838000	1.719782000

O	1.056900000	-0.229148000	4.518871000
H	1.630686000	0.520178000	4.206360000
H	0.128880000	0.059081000	4.352579000
O	2.605981000	1.785446000	3.525943000
H	3.122782000	1.372929000	2.803301000
H	2.007624000	2.428797000	3.089692000
O	-1.583285000	0.558484000	4.094496000
H	-2.099642000	-0.199102000	3.637236000
H	-1.959726000	0.596399000	4.987318000
C	-0.586634000	-0.954992000	-0.072358000
H	-0.092197000	-0.060846000	0.321863000
H	0.023982000	-1.397088000	-0.866802000
H	-0.746757000	-1.677663000	0.732452000
F	-1.819302000	-0.587516000	-0.612424000

N_2O within $5^{12}6^4$ clathrate hydrate

O	-0.576429000	1.035499000	-4.376992000
H	0.368614000	0.841168000	-4.185002000
H	-0.802701000	1.802318000	-3.800630000
O	3.345657000	-1.762541000	-2.808495000
H	2.645988000	-2.417315000	-2.583034000
H	2.863150000	-0.986646000	-3.161772000
O	1.419873000	-3.681539000	-2.156814000
H	1.637571000	-4.454114000	-2.700681000
H	0.442958000	-3.485799000	-2.375042000
O	-1.087379000	-3.348694000	-2.715971000
H	-1.645995000	-3.506930000	-1.908898000
H	-1.387429000	-2.486877000	-3.080550000
O	1.633630000	-4.378143000	0.496563000

H	2.288187000	-3.731728000	0.859207000
H	1.561449000	-4.154842000	-0.464441000
O	3.430828000	-2.531549000	1.483229000
H	3.019667000	-2.072138000	2.294956000
H	4.194532000	-3.012368000	1.837926000
O	-0.655144000	-4.114377000	1.643483000
H	-0.876220000	-5.024887000	1.894751000
H	0.256981000	-4.195111000	1.174969000
O	-4.013105000	0.194859000	2.087219000
H	-4.843978000	0.167977000	2.585063000
H	-4.113135000	-0.463528000	1.346192000
O	-4.258606000	-1.507339000	0.000051000
H	-5.118072000	-1.951506000	-0.069934000
H	-4.199422000	-0.927515000	-0.841685000
O	-2.514267000	-3.687982000	-0.432704000
H	-1.895376000	-3.776767000	0.324211000
H	-3.085630000	-2.917543000	-0.225929000
O	-0.263503000	-2.436320000	3.720340000
H	-0.443947000	-3.072231000	2.973871000
H	-0.391141000	-2.941527000	4.537454000
O	-4.170504000	-0.043461000	-2.131973000
H	-3.917498000	0.879812000	-1.894147000
H	-3.445378000	-0.368335000	-2.719703000
O	-3.486745000	2.569488000	-1.460755000
H	-3.443321000	2.634220000	-0.432134000
H	-4.238248000	3.132171000	-1.704084000
O	-2.138001000	-0.971549000	-3.759655000
H	-2.515071000	-1.231649000	-4.614297000
H	-1.504372000	-0.209622000	-3.976161000
O	3.150933000	2.730302000	2.015687000

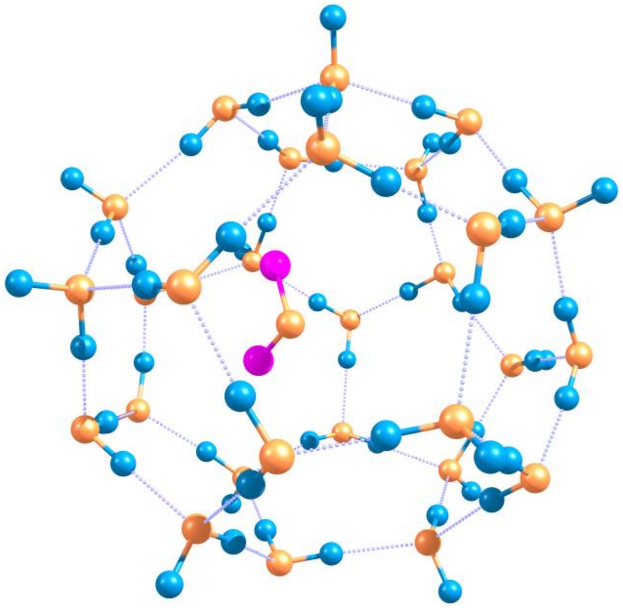

H	3.406721000	2.233908000	1.210474000
H	2.476009000	3.406106000	1.709148000
O	4.260488000	1.540492000	-0.295949000
H	4.349901000	0.512533000	-0.373547000
H	5.152474000	1.852111000	-0.077768000
O	4.576034000	-0.983373000	-0.532203000
H	4.129981000	-1.485893000	0.188244000
H	4.159718000	-1.304398000	-1.380812000
O	2.382616000	-1.513448000	3.631547000
H	2.322667000	-0.525553000	3.656332000
H	1.449716000	-1.821318000	3.674607000
O	3.193561000	2.579627000	-2.471416000
H	3.631800000	2.197921000	-1.646985000
H	3.854046000	3.160984000	-2.877620000
O	2.150539000	0.558580000	-3.935641000
H	2.551854000	1.318517000	-3.432607000
H	2.573836000	0.573480000	-4.807787000
O	1.116078000	4.340525000	-1.576051000
H	1.826825000	3.729619000	-1.858069000
H	0.290125000	3.878670000	-1.828088000
O	-1.202697000	3.196256000	-2.740790000
H	-2.032907000	2.998445000	-2.223198000
H	-1.411519000	3.972534000	-3.282454000
O	1.273930000	4.448853000	1.188719000
H	0.408392000	4.096014000	1.489230000
H	1.215463000	4.465976000	0.202075000
O	-1.207268000	3.590507000	2.247473000
H	-1.414725000	4.406409000	2.729626000
H	-0.955892000	2.927917000	2.987294000
O	-3.534483000	2.756564000	1.093899000

H	-2.674143000	3.058292000	1.488688000
H	-3.692846000	1.860124000	1.468607000
O	-0.567662000	1.999486000	4.186207000
H	-1.119737000	1.172794000	4.187392000
H	0.359397000	1.696056000	4.048739000
O	-2.062375000	-0.281091000	4.083484000
H	-2.668965000	-0.182332000	3.320789000
H	-1.457302000	-1.015808000	3.850384000
O	2.086526000	1.208620000	3.814949000
H	2.491165000	1.785516000	3.071946000
H	2.537890000	1.504201000	4.620521000
O	-0.746344000	-1.169279000	-0.299957000
N	-1.197678000	-0.050050000	-0.758207000
N	-1.011117000	-0.700420000	0.874561000

NF_3 within $5^{12}6^4$ clathrate hydrate

O	-0.275813000	-1.888463000	4.186900000
H	0.670357000	-1.767778000	3.945570000
H	-0.641131000	-2.485812000	3.494324000
O	3.784791000	0.768636000	2.760869000
H	3.162015000	1.531575000	2.740911000
H	3.233118000	-0.005647000	2.995734000
O	2.096736000	2.989757000	2.700359000
H	2.454961000	3.599521000	3.363635000
H	1.119126000	2.867746000	2.972308000
O	-0.378082000	2.831860000	3.443364000
H	-0.961026000	3.209399000	2.729368000
H	-0.726192000	1.929335000	3.611983000
O	2.229999000	4.198684000	0.232615000

H	2.761806000	3.569419000	-0.313034000
H	2.190431000	3.785540000	1.130787000
O	3.681876000	2.402462000	-1.286585000
H	3.147271000	2.175721000	-2.122704000
H	4.471720000	2.856450000	-1.618481000
O	-0.156067000	4.430979000	-0.711762000
H	-0.280795000	5.392332000	-0.743351000
H	0.791055000	4.309526000	-0.332343000
O	-4.071643000	0.672362000	-1.619548000
H	-4.908215000	0.945545000	-2.025332000
H	-4.017314000	1.158386000	-0.749823000
O	-3.915157000	1.893307000	0.777022000
H	-4.687594000	2.423106000	1.028400000
H	-3.868677000	1.155567000	1.482796000
O	-1.904886000	3.786351000	1.413224000
H	-1.312053000	3.933932000	0.644834000
H	-2.525367000	3.081403000	1.131163000
O	-0.181310000	3.176355000	-3.093861000
H	-0.204603000	3.659210000	-2.220252000
H	-0.297619000	3.856537000	-3.774393000
O	-3.871570000	0.023895000	2.572456000
H	-3.747892000	-0.846291000	2.126475000
H	-3.071855000	0.141663000	3.141240000
O	-3.573838000	-2.438037000	1.301640000
H	-3.653818000	-2.290925000	0.284981000
H	-4.367829000	-2.944705000	1.532693000
O	-1.593907000	0.378142000	4.093690000
H	-1.836829000	0.528083000	5.020288000
H	-1.051664000	-0.477154000	4.103714000
O	2.657729000	-2.555820000	-2.805023000

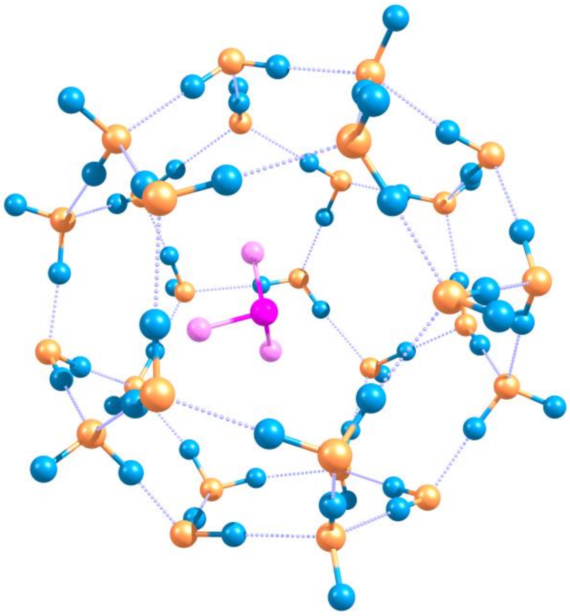

H	3.046885000	-2.272756000	-1.951059000
H	1.927463000	-3.199429000	-2.565145000
O	4.097327000	-2.012058000	-0.436440000
H	4.318924000	-1.037841000	-0.161441000
H	4.925285000	-2.360568000	-0.801823000
O	4.743561000	0.352190000	0.271007000
H	4.320291000	1.041509000	-0.291583000
H	4.428822000	0.533588000	1.201229000
O	2.329596000	1.985282000	-3.470896000
H	2.136899000	1.038088000	-3.682633000
H	1.444876000	2.398162000	-3.352788000
O	3.104015000	-3.372818000	1.590789000
H	3.512673000	-2.870676000	0.816862000
H	3.724067000	-4.087795000	1.799647000
O	2.436771000	-1.614170000	3.537750000
H	2.702885000	-2.286062000	2.853142000
H	2.956321000	-1.829004000	4.327633000
O	0.758763000	-4.676482000	0.591108000
H	1.564713000	-4.223219000	0.912652000
H	0.024359000	-4.196361000	1.025783000
O	-1.295101000	-3.558472000	2.197379000
H	-2.129120000	-3.169947000	1.810958000
H	-1.559241000	-4.406627000	2.585361000
O	0.640606000	-4.181379000	-2.129347000
H	-0.194015000	-3.677511000	-2.241467000
H	0.674654000	-4.415299000	-1.169334000
O	-1.801189000	-2.871964000	-2.723069000
H	-2.102866000	-3.557280000	-3.340227000
H	-1.523449000	-2.100193000	-3.336320000
O	-3.932111000	-2.083694000	-1.213274000

H	-3.164069000	-2.389489000	-1.763852000
H	-4.007193000	-1.117938000	-1.389193000
O	-1.104931000	-1.003190000	-4.372155000
H	-1.543242000	-0.132245000	-4.178332000
H	-0.137755000	-0.837651000	-4.283937000
O	-2.275427000	1.364881000	-3.693791000
H	-2.797533000	1.155209000	-2.891899000
H	-1.559226000	1.963642000	-3.394505000
O	1.647283000	-0.590233000	-4.144350000
H	2.040004000	-1.347782000	-3.577612000
H	1.986067000	-0.759057000	-5.037051000
N	-0.634339000	0.379210000	-0.081661000
F	-1.118068000	0.528020000	1.208505000
F	-1.464680000	-0.606468000	-0.582846000
F	-1.133104000	1.515278000	-0.702715000

O_3 within $5^{12}6^4$ clathrate hydrate

O	-0.396074000	2.184130000	-4.034729000
H	0.528866000	1.872031000	-3.909166000
H	-0.558076000	2.808791000	-3.290803000
O	3.196150000	-1.273931000	-3.199274000
H	2.444390000	-1.906748000	-3.132188000
H	2.780260000	-0.396823000	-3.328674000
O	1.126279000	-3.135764000	-3.016938000
H	1.263790000	-3.751267000	-3.753278000
H	0.169767000	-2.803006000	-3.142792000
O	-1.337657000	-2.449381000	-3.415879000
H	-1.927606000	-2.763604000	-2.677567000
H	-1.537313000	-1.495117000	-3.532244000

O	1.275496000	-4.501389000	-0.638477000
H	1.980922000	-4.025046000	-0.135883000
H	1.229115000	-4.036400000	-1.510966000
O	3.225810000	-3.125134000	0.753247000
H	2.845929000	-2.844905000	1.654650000
H	3.940981000	-3.742165000	0.972032000
O	-0.993250000	-4.317098000	0.560195000
H	-1.303947000	-5.235966000	0.570046000
H	-0.086013000	-4.360310000	0.076329000
O	-3.977415000	-0.017812000	2.078857000
H	-4.801885000	-0.099178000	2.581572000
H	-4.144301000	-0.461951000	1.200378000
O	-4.398023000	-1.111966000	-0.347284000
H	-5.301714000	-1.415785000	-0.524631000
H	-4.246788000	-0.353228000	-1.013350000
O	-2.833642000	-3.268864000	-1.311143000
H	-2.202717000	-3.556745000	-0.616465000
H	-3.321916000	-2.509658000	-0.925799000
O	-0.489450000	-3.245187000	2.970596000
H	-0.712456000	-3.666605000	2.092731000
H	-0.646971000	-3.927897000	3.640301000
O	-4.108879000	0.829191000	-2.047338000
H	-3.790315000	1.646581000	-1.598691000
H	-3.407577000	0.608303000	-2.707678000
O	-3.190663000	3.138138000	-0.781324000
H	-3.161508000	2.934261000	0.229341000
H	-3.896860000	3.796345000	-0.874310000
O	-2.112591000	0.212522000	-3.851057000
H	-2.498202000	0.176147000	-4.739885000
H	-1.419848000	0.951096000	-3.900620000

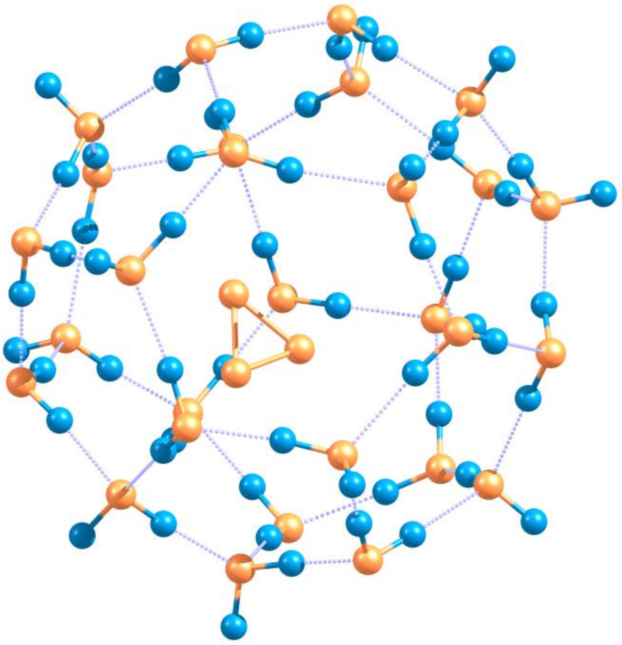

O	3.366780000	1.835515000	2.593509000
H	3.596417000	1.536614000	1.688635000
H	2.760227000	2.623659000	2.467796000
O	4.402038000	1.180027000	0.048430000
H	4.395759000	0.201436000	-0.290789000
H	5.319900000	1.342301000	0.315964000
O	4.492997000	-1.214048000	-0.831412000
H	4.006658000	-1.845942000	-0.252709000
H	4.046040000	-1.268535000	-1.722783000
O	2.235733000	-2.583679000	3.100306000
H	2.260086000	-1.633370000	3.375652000
H	1.278493000	-2.803763000	3.059276000
O	3.463351000	2.830172000	-1.783239000
H	3.849667000	2.211089000	-1.086834000
H	4.180887000	3.434106000	-2.027366000
O	2.261958000	1.362637000	-3.715750000
H	2.723537000	1.924396000	-3.035888000
H	2.738510000	1.516963000	-4.545809000
O	1.533807000	4.485180000	-0.464882000
H	2.194471000	3.904862000	-0.894919000
H	0.677308000	4.182822000	-0.829892000
O	-0.855947000	3.903436000	-1.887807000
H	-1.706501000	3.649522000	-1.432627000
H	-0.983059000	4.814926000	-2.191944000
O	1.654651000	3.863680000	2.233444000
H	0.761471000	3.510104000	2.434352000
H	1.614071000	4.139276000	1.285046000
O	-0.896307000	2.963037000	3.051615000
H	-1.037127000	3.661309000	3.710314000
H	-0.712121000	2.127386000	3.616423000

O	-3.255204000	2.650261000	1.729715000
H	-2.385702000	2.768673000	2.196158000
H	-3.509238000	1.709925000	1.874110000
O	-0.423453000	0.909920000	4.549978000
H	-1.045697000	0.158815000	4.358494000
H	0.477428000	0.569167000	4.341283000
O	-2.113901000	-1.130245000	3.894615000
H	-2.702126000	-0.800253000	3.184172000
H	-1.576412000	-1.842928000	3.488680000
O	2.151178000	0.019881000	3.972870000
H	2.613509000	0.723857000	3.389324000
H	2.625246000	0.060490000	4.817678000
O	-0.996299000	-1.039051000	0.481021000
O	-1.249883000	-0.548955000	-0.847361000
O	-1.342553000	0.342517000	0.281223000

CF_4 within $5^{12}6^4$ clathrate hydrate

O	0.559574000	-3.286571000	-3.173920000
H	-0.358552000	-2.933732000	-3.153428000
H	0.703127000	-3.665004000	-2.275966000
O	-2.776264000	0.386365000	-3.404448000
H	-1.997270000	0.976092000	-3.530735000
H	-2.409969000	-0.508553000	-3.251207000
O	-0.675918000	2.146676000	-3.839467000
H	-0.828858000	2.498359000	-4.729901000
H	0.273617000	1.768691000	-3.873821000
O	1.753848000	1.284336000	-4.026533000
H	2.328770000	1.791723000	-3.390671000
H	1.870963000	0.339177000	-3.785372000

O	-0.826129000	4.181926000	-2.007326000
H	-1.561818000	3.909174000	-1.405959000
H	-0.776603000	3.466064000	-2.688986000
O	-2.863302000	3.394416000	-0.316273000
H	-2.499573000	3.413378000	0.634090000
H	-3.545870000	4.082847000	-0.332349000
O	1.419741000	4.275853000	-0.757675000
H	1.774377000	5.137580000	-1.027013000
H	0.516595000	4.209048000	-1.246715000
O	4.143709000	0.537043000	2.060388000
H	4.968889000	0.731965000	2.529656000
H	4.341376000	0.668872000	1.090951000
O	4.616528000	0.771501000	-0.578069000
H	5.521431000	1.011424000	-0.831318000
H	4.473718000	-0.156974000	-0.983763000
O	3.218836000	2.637287000	-2.188614000
H	2.579407000	3.137717000	-1.636243000
H	3.633160000	1.986653000	-1.582504000
O	0.850438000	4.045655000	1.857251000
H	1.101899000	4.149483000	0.895829000
H	1.032589000	4.902659000	2.271550000
O	4.319191000	-1.585471000	-1.610670000
H	3.966668000	-2.213101000	-0.937112000
H	3.622057000	-1.539922000	-2.310772000
O	3.294095000	-3.332589000	0.301614000
H	3.271827000	-2.822298000	1.197844000
H	3.951550000	-4.032173000	0.438644000
O	2.352220000	-1.409660000	-3.532768000
H	2.738873000	-1.644612000	-4.390361000
H	1.624104000	-2.098952000	-3.382494000

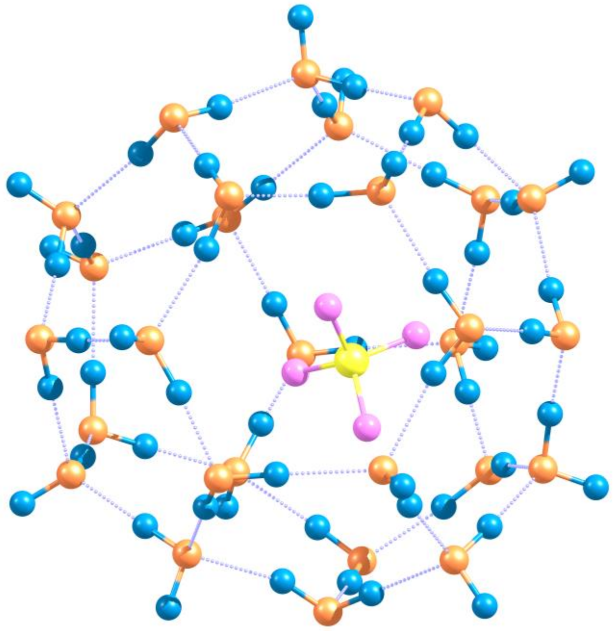

O	-3.294163000	-0.744771000	2.880869000
H	-3.574758000	-0.752726000	1.941749000
H	-2.716115000	-1.555259000	2.995579000
O	-4.376820000	-0.922179000	0.276367000
H	-4.261229000	-0.082133000	-0.316159000
H	-5.313128000	-0.922245000	0.528392000
O	-4.189736000	1.124179000	-1.238176000
H	-3.691391000	1.880564000	-0.851465000
H	-3.698762000	0.882023000	-2.073065000
O	-1.910308000	3.590712000	2.096301000
H	-1.993242000	2.764722000	2.635111000
H	-0.941656000	3.741667000	2.022351000
O	-3.501186000	-3.087683000	-0.966110000
H	-3.867826000	-2.274718000	-0.497520000
H	-4.239159000	-3.710827000	-1.042587000
O	-2.072394000	-2.336364000	-3.141794000
H	-2.616383000	-2.647284000	-2.369461000
H	-2.540369000	-2.646094000	-3.932303000
O	-1.556206000	-4.256464000	0.774009000
H	-2.223186000	-3.843963000	0.188028000
H	-0.695391000	-4.050486000	0.356095000
O	0.915810000	-4.235957000	-0.587649000
H	1.777096000	-3.912124000	-0.202478000
H	0.949086000	-5.203077000	-0.532887000
O	-1.648782000	-2.833364000	3.136385000
H	-0.738371000	-2.479397000	3.231319000
H	-1.627547000	-3.392609000	2.321300000
O	0.932905000	-1.872959000	3.740183000
H	0.984298000	-2.347702000	4.584887000
H	0.783277000	-0.898042000	4.018679000

O	3.365779000	-2.096575000	2.544167000
H	2.471398000	-2.028969000	2.972313000
H	3.648932000	-1.165608000	2.393636000
O	0.528121000	0.546359000	4.552555000
H	1.195612000	1.189460000	4.193703000
H	-0.345957000	0.848475000	4.212381000
O	2.350983000	2.247560000	3.440383000
H	2.907659000	1.678407000	2.869550000
H	1.849120000	2.825821000	2.827499000
O	-1.992749000	1.343334000	3.664183000
H	-2.489142000	0.527227000	3.295996000
H	-2.460241000	1.555551000	4.486734000
C	-0.722083000	-0.109013000	0.044658000
F	-1.596485000	0.726372000	0.594204000
F	0.212201000	-0.425418000	0.933841000
F	-1.349876000	-1.216551000	-0.348574000
F	-0.156860000	0.471938000	-1.010420000

SF_6 within $5^{12}6^4$ clathrate hydrate

O	0.755495000	-2.080137000	-4.005901000
H	-0.137830000	-2.314477000	-3.666195000
H	1.385742000	-2.398440000	-3.319281000
O	-3.871620000	-1.013561000	-2.355048000
H	-3.610366000	-0.071643000	-2.478532000
H	-3.074729000	-1.535639000	-2.580914000
O	-3.249556000	1.676352000	-2.688418000
H	-3.865867000	2.000054000	-3.363271000
H	-2.323945000	1.901965000	-3.060377000
O	-0.976562000	2.366029000	-3.701096000

H	-0.538729000	3.038688000	-3.111000000
H	-0.317346000	1.645152000	-3.805237000
O	-3.644425000	3.062493000	-0.353358000
H	-3.834780000	2.354625000	0.309757000
H	-3.520444000	2.580070000	-1.208663000
O	-4.127142000	1.059808000	1.480773000
H	-3.465634000	1.168886000	2.246971000
H	-4.996052000	1.210778000	1.883649000
O	-1.486437000	4.325840000	0.241986000
H	-1.763689000	5.253524000	0.185378000
H	-2.332754000	3.791020000	0.003329000
O	3.673083000	2.510155000	1.016367000
H	4.406039000	3.090653000	1.270886000
H	3.363014000	2.830965000	0.122751000
O	2.827141000	3.237884000	-1.420558000
H	3.280191000	3.991076000	-1.830565000
H	3.030202000	2.447182000	-2.038121000
O	0.202915000	4.110916000	-1.998818000
H	-0.345534000	4.146040000	-1.184987000
H	1.065174000	3.737539000	-1.717772000
O	-0.730822000	3.496661000	2.680503000
H	-0.981617000	3.836568000	1.775203000
H	-0.843568000	4.245929000	3.284749000
O	3.392674000	1.280388000	-3.011911000
H	3.706731000	0.494639000	-2.506624000
H	2.559246000	0.988780000	-3.457844000
O	4.249759000	-0.929582000	-1.554221000
H	4.308739000	-0.630849000	-0.569023000
H	5.167295000	-1.119951000	-1.803925000
O	1.048850000	0.508602000	-4.222710000

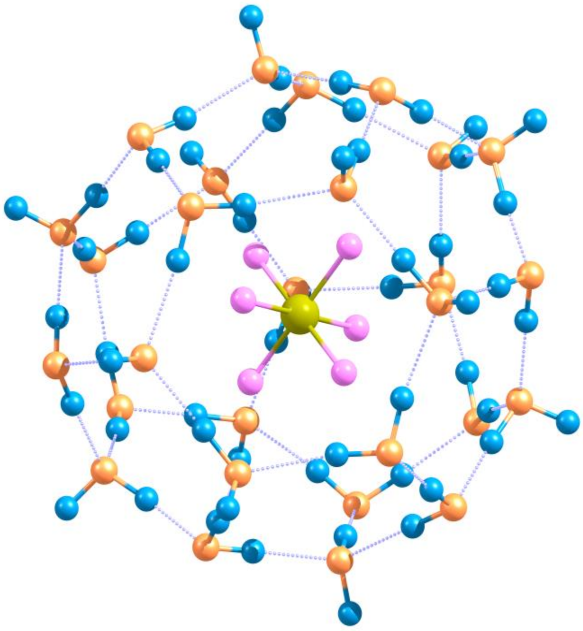

H	1.144656000	0.634216000	-5.179405000
H	0.896442000	-0.487241000	-4.107333000
O	-1.078892000	-2.818437000	3.261371000
H	-1.599640000	-2.812382000	2.431074000
H	-0.176488000	-3.178640000	3.011564000
O	-2.795034000	-3.217135000	1.079621000
H	-3.418398000	-2.457015000	0.748588000
H	-3.364066000	-3.796254000	1.609910000
O	-4.398224000	-1.420830000	0.254377000
H	-4.243952000	-0.552411000	0.693473000
H	-4.251659000	-1.260310000	-0.720838000
O	-2.516935000	1.494035000	3.471485000
H	-1.949897000	0.735481000	3.758781000
H	-1.880516000	2.193318000	3.201518000
O	-1.522235000	-4.380318000	-0.906364000
H	-2.031907000	-3.964732000	-0.140670000
H	-1.817251000	-5.302229000	-0.953542000
O	-1.780182000	-2.793004000	-3.078014000
H	-1.701124000	-3.414163000	-2.304219000
H	-2.246435000	-3.291116000	-3.766805000
O	1.213384000	-4.491689000	-0.085274000
H	0.272459000	-4.437682000	-0.349878000
H	1.663094000	-3.834863000	-0.655175000
O	2.534530000	-2.942527000	-2.046457000
H	3.178963000	-2.215696000	-1.817603000
H	3.073753000	-3.675910000	-2.379491000
O	1.345556000	-3.677128000	2.549783000
H	1.928312000	-2.887577000	2.529009000
H	1.334857000	-4.016643000	1.621351000
O	3.088060000	-1.463428000	2.735971000

H	3.719083000	-1.864554000	3.354137000
H	2.582027000	-0.780948000	3.309449000
O	4.536954000	-0.129798000	0.859990000
H	4.002382000	-0.633411000	1.529073000
H	4.234390000	0.804234000	0.934259000
O	1.854364000	0.210544000	4.268361000
H	1.896594000	1.146906000	3.937651000
H	0.895995000	-0.017748000	4.291253000
O	1.928930000	2.727254000	3.226590000
H	2.450952000	2.650109000	2.401097000
H	1.018022000	2.954678000	2.944145000
O	-0.838410000	-0.489779000	4.337331000
H	-0.935499000	-1.398260000	3.872857000
H	-1.037203000	-0.671234000	5.268777000
S	0.041472000	-0.037920000	0.020898000
F	0.457754000	-1.524385000	0.443818000
F	-1.344000000	-0.198105000	0.807372000
F	0.732916000	0.537800000	1.344864000
F	-0.373485000	1.449151000	-0.410399000
F	-0.646291000	-0.611250000	-1.310181000
F	1.428359000	0.124434000	-0.773094000

SO_2 within $5^{12}6^4$ clathrate hydrate

O	-0.542452000	4.181657000	-1.944977000
H	-0.199395000	3.588352000	-2.646772000
H	0.157787000	4.168054000	-1.249343000
O	-0.724567000	0.071497000	-2.837634000
H	-1.660995000	-0.096170000	-3.115124000
H	-0.440158000	0.930612000	-3.226663000

O	-3.311111000	-0.595732000	-3.134532000
H	-3.860198000	-0.598273000	-3.932444000
H	-3.758876000	0.055625000	-2.482017000
O	-4.354759000	1.065251000	-1.460226000
H	-4.412178000	0.601200000	-0.580311000
H	-3.751361000	1.826242000	-1.308911000
O	-2.951912000	-3.114420000	-1.965192000
H	-2.000881000	-3.348168000	-2.101176000
H	-3.084622000	-2.244804000	-2.408018000
O	-0.318337000	-3.901595000	-2.322038000
H	0.104589000	-4.075838000	-1.407747000
H	-0.343447000	-4.772138000	-2.747678000
O	-3.435760000	-2.884548000	0.570027000
H	-4.227816000	-3.435335000	0.669919000
H	-3.208607000	-2.946794000	-0.425419000
O	-0.953307000	0.243796000	4.376260000
H	-1.204094000	0.195274000	5.310866000
H	-1.768399000	0.541584000	3.887001000
O	-3.090328000	1.130965000	2.979245000
H	-3.856455000	1.386513000	3.516474000
H	-2.785633000	2.001907000	2.532124000
O	-4.371791000	-0.268130000	0.891007000
H	-4.002693000	-1.176568000	0.814923000
H	-3.885382000	0.161161000	1.626828000
O	-1.248502000	-3.815853000	1.880932000
H	-2.044580000	-3.430204000	1.424356000
H	-1.558579000	-4.628887000	2.307144000
O	-2.339537000	3.340487000	1.881669000
H	-1.373369000	3.484306000	2.028075000
H	-2.462035000	3.313863000	0.901878000

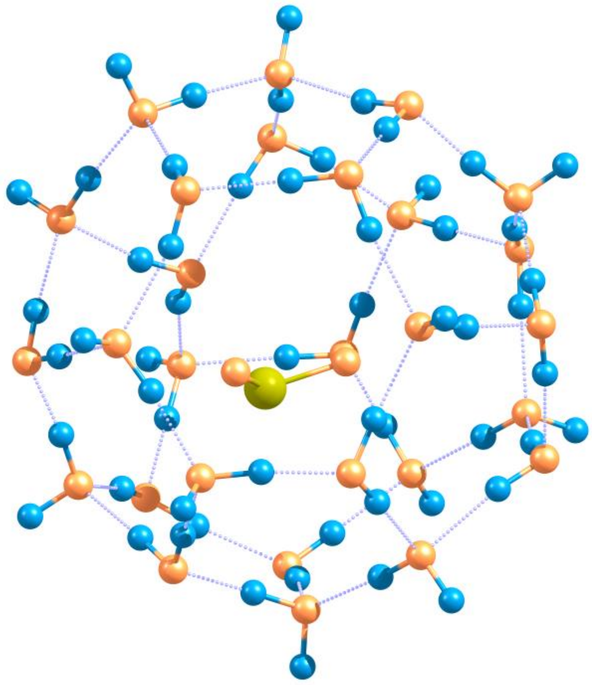

O	0.342085000	3.789639000	2.374636000
H	0.710750000	3.022644000	2.954627000
H	0.402204000	4.570210000	2.946744000
O	-2.761719000	3.310732000	-0.854980000
H	-3.355613000	4.060368000	-1.015929000
H	-1.889266000	3.579333000	-1.298325000
O	4.371943000	-1.698131000	-0.671794000
H	3.870095000	-1.440954000	-1.472377000
H	4.540920000	-0.840121000	-0.179926000
O	3.191226000	-1.039183000	-3.173217000
H	2.274885000	-1.451592000	-3.388711000
H	3.821868000	-1.493760000	-3.752261000
O	0.865918000	-1.930368000	-3.821631000
H	0.548612000	-2.704951000	-3.295582000
H	0.271307000	-1.199796000	-3.517063000
O	0.682592000	-4.509595000	-0.013486000
H	1.512812000	-4.026088000	0.223912000
H	0.026554000	-4.227594000	0.663612000
O	2.969282000	1.585779000	-3.443425000
H	3.083659000	0.588325000	-3.375999000
H	3.716104000	1.911838000	-3.967291000
O	0.426254000	2.360627000	-3.872510000
H	1.384890000	2.115507000	-3.752243000
H	0.316333000	2.555288000	-4.814950000
O	3.736849000	2.670288000	-0.894665000
H	3.432404000	2.243656000	-1.719832000
H	2.918256000	3.044909000	-0.506867000
O	1.478914000	4.142980000	-0.043879000
H	1.097055000	3.953591000	0.857296000
H	1.918876000	5.002925000	0.035795000

O	4.716813000	0.596023000	0.666537000
H	4.119724000	0.557848000	1.445144000
H	4.394738000	1.363074000	0.132671000
O	3.223013000	0.413967000	3.061393000
H	3.969453000	0.506069000	3.673865000
H	2.952794000	-0.569588000	3.150331000
O	1.196731000	1.983763000	3.983448000
H	1.937645000	1.429647000	3.620355000
H	0.457744000	1.353731000	4.139867000
O	2.640271000	-2.095751000	3.335337000
H	1.692447000	-2.237468000	3.598514000
H	2.731936000	-2.516479000	2.448863000
O	-0.001490000	-2.397902000	3.978803000
H	-0.348020000	-1.482484000	4.007410000
H	-0.435129000	-2.811352000	3.202115000
O	2.992445000	-3.289423000	0.838556000
H	3.491649000	-2.634390000	0.232204000
H	3.634790000	-3.995595000	1.007349000
S	-1.062237000	0.250808000	-0.226313000
O	-0.666153000	-1.127883000	0.083181000
O	-0.085026000	1.313416000	0.046871000

References:

[1]. Steinmann, Stephan N., Yirong Mo, and Clemence Corminboeuf. "How do electron localization functions describe π-electron delocalization?." *Phys. Chem. Chem. Phys.* **2011**, 13. 46, 20584-20592.

[2]. Lu, T., & CHEN, F. W. Meaning and functional form of the electron localization function. *Acta Physico-Chimica Sinica*, **2011**, 27(12), 2786-2792.

[3]. Savin, A., Jepsen, O., Flad, J., Andersen, O. K., Preuss, H., & von Schnering, H. G. Electron localization in solid-state structures of the elements: the diamond structure. *Angewandte Chemie International Edition in English*, **1992**, 31(2), 187-188.

[4]. A.S. Kazachenko, F. Akman, Y.N. Malyar, N. Issaoui, N.Y. Vasilieva, Synthesis optimization, DFT and physicochemical study of chitosan sulfates, *J. Mol. Struct.* **2021**, 1245, 131083.

[5]. N. Rekik, N. Issaoui, B. Oujia, M.J. W´ojcik, Theoretical IR spectral density of H-bond in liquid phase: Combined effects of anharmonicities, Fermi resonances, direct and indirect relaxations, *J. Mol. Liq*. **2008**, 141 (3), 104–109.

[6]. R. Trivedi, A. Banerjee, D. Bandyopadhyay, *Phys. E* **2021**, *131*, 114725.
